# Supplementary material for: Synergistic catalysis for cascade allylation and 2-aza-cope rearrangement of azomethine ylides
Source: Nat Commun. 2019 Apr 8;10:1594. doi: 10.1038/s41467-019-09563-6 (PMC6453969; doi:10.1038/s41467-019-09563-6)
Supplement: Supplementary file 1 — Supplementary Information [file 41467_2019_9563_MOESM1_ESM.pdf]

Supplementary Information for

Synergistic Catalysis for Cascade Allylation and 2-aza-Cope Rearrangement of Azomethine Ylides

Liang *et al*.

Supplementary Methods

General information

^{1}H NMR spectra were recorded on a Bruker Mercury 400 MHz spectrometer in $CDCl_3$, CD_2Cl_2 and Toluene-D_8. Chemical shifts are reported in ppm with the internal TMS signal at 0.0 ppm as a standard. The data are reported as (s = single, d = double, t = triple, q = quartet, m = multiple or unresolved, and brs = broad single). ^{13}C NMR spectra were recorded on a Bruker 100 MHz spectrometer in $CDCl_3$. ^{19}F NMR spectra were recorded on a Bruker 376 MHz spectrometer in $CDCl_3$. Chemical shifts are reported in ppm. Commercially available reagents were used without further purification. All reactions were monitored by TLC with silica gel-coated plates. Enantiomeric ratios were determined by HPLC, using a chiralpak AD-H, OD-H, OJ-H and IA column with hexane and *i*-PrOH as solvents. Aldimino ester[1], allyl carbonates[2], **L1**[3] and **L2**[4] were prepared according to the literature procedure. The absolute configuration of the known product **5** was assigned by optical rotation comparison with the reported data,[5] the absolute configuration of the **3Q** was assigned by optical rotation comparisons with the reported data after deprotection,[6] and those of other products were deduced on the basis of those results.

General Procedure for Synergistic Cu/Ir Catalyzed Cascade Allylation/2-*aza*-Cope Rearrangement Reaction

General Reaction Procedure A:

A flame dried Schlenk tube I was cooled to rt and filled with N_2. To this flask were added $[Ir(COD)Cl]_2$ (0.003 mmol, 1.5 mol %), phosphoramidite ligand (*S,S,S*)-**L2** (0.006 mmol, 3 mol %), degassed THF (0.5 mL) and degassed *n*-propylamine (0.5 mL). The reaction mixture was heated at 50 °C for 30 min and then the volatile solvents were removed under vacuum to give the iridium complex as a pale yellow solid. Meanwhile, in a separated Schlenk tube II, DPEPhos (0.011 mmol, 5.5 mol %) and $Cu(MeCN)_4BF_4$ (0.01 mmol, 5 mol %) were dissolved in 0.5 mL of DCM, and stirred at room temperature for about 0.5 h. The Cu/DPEphos complex solution was then transferred to the Schlenk tube I containing iridium complex via syringe. Allylic carbonate (0.20 mmol), leucine derived aldimine ester (0.30 mmol), Cs_2CO_3 (0.30 mmol) and DCM

(0.5 mL) were then added. The cascade reaction was finished smoothly in 12-24 h (monitored by ^{1}H NMR spectroscopy).

General Reaction Procedure B:

A flame dried Schlenk tube I was cooled to rt and filled with N_2. To this flask were added $[Ir(COD)Cl]_2$ (0.003 mmol, 1.5 mol %), phosphoramidite ligand (*S,S,S*)-**L2** (0.006 mmol, 3 mol %), degassed THF (0.5 mL) and degassed *n*-propylamine (0.5 mL). The reaction mixture was heated at 50 °C for 30 min and then the volatile solvents were removed under vacuum to give a pale yellow solid. Meanwhile, in a separated Schlenk tube II, DPEPhos (0.011 mmol, 5.5 mol %) and $Cu(MeCN)_4BF_4$ (0.01 mmol, 5 mol %) were dissolved in 0.5 mL of DCM, and stirred at room temperature for about 0.5 h. The Cu/DPEphos complex solution was then transferred to the Schlenk tube I containing iridium complex via syringe. Allylic carbonate (0.20 mmol), leucine derived aldimine ester (0.30 mmol), Cs_2CO_3 (0.30 mmol) and DCM (0.5 mL) were then added. Once starting material was consumed (monitored by TLC), 1 mL of DCE was added and the reaction was heated to 50 °C for 3 h to accelerate the 2-aza-Cope rearrangement process.

General Reaction Procedure C:

A flame dried Schlenk tube I was cooled to rt and filled with N_2. To this flask were added $[Ir(COD)Cl]_2$ (0.003 mmol, 1.5 mol %), phosphoramidite ligand (*S,S,S*)-**L2** (0.006 mmol, 3 mol %), degassed THF (0.5 mL) and degassed *n*-propylamine (0.5 mL). The reaction mixture was heated at 50 °C for 30 min and then the volatile solvents were removed under vacuum to give a pale yellow solid. Meanwhile, in a separated Schlenk tube II, (S,S_p)-**L1** (0.011 mmol, 5.5 mol %) and $Cu(MeCN)_4BF_4$ (0.01 mmol, 5 mol %) were dissolved in 0.5 mL of DCM, and stirred at room temperature for about 0.5 h. The Cu/(S,S_p)-**L1** complex solution was then transferred to the Schlenk tube I containing iridium complex via syringe. Allylic carbonate (0.20 mmol), phenylglycine derived aldimine ester (0.30 mmol), Cs_2CO_3 (0.30 mmol) and DCM (0.5 mL) were then added. Once starting material was consumed (monitored by TLC), 1 mL of toluene was added and the reaction was heated to 100 °C for 8 h to accelerate the 2-aza-Cope rearrangement process.

General Reaction Procedure D:

For *ortho*-substituted cinnamyl carbonates, a flame dried Schlenk tube I was cooled to rt and filled with N_2. To this flask were added $[Ir(COD)Cl]_2$ (0.003 mmol, 1.5 mol %), phosphoramidite ligand (S,S_a)-**L4** (0.006 mmol, 3 mol %), degassed THF (0.5 mL) and degassed *n*-propylamine (0.5 mL). The reaction mixture was heated at 50 °C for 30 min and then the volatile solvents were removed under vacuum to give a pale yellow solid. Meanwhile, in a separated Schlenk tube II, DPEPhos (0.011 mmol, 5.5 mol %) and $Cu(MeCN)_4BF_4$ (0.01 mmol, 5 mol %) were dissolved in 0.5 mL of DCM, and stirred at room temperature for about 0.5 h. The Cu/DPEphos complex solution was then transferred to the Schlenk tube I containing iridium complex via syringe. Allylic carbonate (0.20 mmol), leucine derived aldimine ester (0.30 mmol), Cs_2CO_3 (0.30 mmol) and DCM (0.5 mL) were then added. The cascade reaction was finished smoothly in 24 h (monitored by ^1H NMR spectroscopy).

General Reaction Procedure E:

A flame dried Schlenk tube I was cooled to rt and filled with N_2. To this flask were added $[Ir(COD)Cl]_2$ (0.003 mmol, 1.5 mol %), phosphoramidite ligand (*rac*)-**L2** (0.006 mmol, 3 mol %), degassed THF (0.5 mL) and degassed *n*-propylamine (0.5 mL). The reaction mixture was heated at 50 °C for 30 min and then the volatile solvents were removed under vacuum to give a pale yellow solid. Meanwhile, in a separated Schlenk tube II, (S,S_p)-**L1** (0.011 mmol, 5.5 mol %) and $Cu(MeCN)_4BF_4$ (0.01 mmol, 5 mol %) were dissolved in 0.5 mL of DCM, and stirred at room temperature for about 0.5 h. The Cu/(S,S_p)-**L1** complex solution was then transferred to the Schlenk tube I containing iridium complex via syringe. Allylic carbonate (0.20 mmol), phenylglycine derived aldimine ester (0.30 mmol), Cs_2CO_3 (0.30 mmol) and DCM (0.5 mL) were then added and the reaction was heated to 35 °C. Once starting material was consumed (monitored by TLC), 1 mL of toluene was added and the reaction was heated to 100 °C for 8 h to accelerate the 2-aza-Cope rearrangement process.

General Workup Procedure:

The reaction was cooled to rt and 2 N HCl (0.5 mL) was added to the mixture. After

stirring for 0.5 h, 2 N NaOH (1 mL) and Boc_2O (88 mg, 0.4 mmol) was then added and stirring at rt for 3 h. The layers were separated, and the aqueous layer was extracted with DCM (5 mL x 2). The combined organic components were washed with saturated brine (10 mL), dried over anhydrous Na_2SO_4, filtration and evaporated in vacuum. The residue was purified by column chromatography to give the desired product, which was then directly analyzed by HPLC to determine the enantiomeric excess.

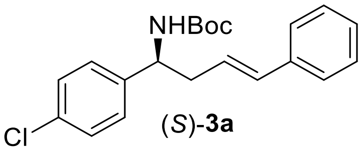

(*S*)-**3a**

***tert*-butyl (*S*,*E*)-(1-(4-chlorophenyl)-4-phenylbut-3-en-1-yl)carbamate (Table 1)**
Following **General Reaction Procedure A:** Yield (94%); white solid, m.p. 118-120 °C; $[\alpha]^{30}_D$ = -26.7 (*c* 0.46, CH_2Cl_2); ^1H NMR (400 MHz, Chloroform-*d*) δ 7.36 – 7.28 (m, 6H), 7.25 – 7.20 (m, 3H), 6.45 (d, *J* = 15.6 Hz, 1H), 6.02 (dt, *J* = 15.6, 7.2 Hz, 1H), 4.90 (s, 1H), 4.79 (s, 1H), 2.65 (m, 2H), 1.39 (s, 9H). ^{13}C NMR (101 MHz, $CDCl_3$) δ 155.1, 136.9, 133.5, 132.8, 128.7, 128.6, 127.6, 127.5, 126.2, 124.8, 79.8, 53.8, 40.3, 28.3. HRMS (ESI+) Calcd. For $C_{21}H_{24}ClNO_2Na$ ($[M+Na]^+$): 380.1388, found: 380.1397. The product was analyzed by HPLC to determine the enantiomeric excess: 96% ee (Chiralpak OD-H, *i*-propanol /hexane = 10/90, flow rate 1.0 mL/min, λ = 254 nm); t_r = 6.69 and 7.42 min.

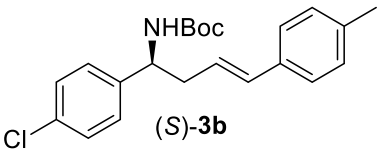

(*S*)-**3b**

***tert*-butyl (*S*,*E*)-(1-(4-chlorophenyl)-4-(*p*-tolyl)but-3-en-1-yl)carbamate (Figure 5)**
Following **General Reaction Procedure A:** Yield (86%); white solid, m.p. 142-144 °C; $[\alpha]^{30}_D$ = -35.0 (*c* 0.14, CH_2Cl_2); ^1H NMR (400 MHz, Chloroform-*d*) δ 7.31 (d, *J* = 8.4 Hz, 2H), 7.25 – 7.15 (m, 4H), 7.10 (d, *J* = 8.0 Hz, 2H), 6.42 (d, *J* = 16.0 Hz, 1H), 5.99 – 5,91 (m, 1H), 4.91 (brs, 1H), 4.78 (brs, 1H), 2.63 - 2.60 (m, 2H), 2.33 (s, 3H), 1.39 (s, 9H). ^{13}C NMR (101 MHz, $CDCl_3$) δ 155.1, 141.0, 137.3, 134.1, 133.4, 132.7, 129.22, 128.6, 127.5, 126.0, 123.7, 79.8, 53.8, 40.3, 28.3, 21.2. HRMS (ESI+) Calcd. For

$C_{22}H_{26}ClNO_2Na$ ([M+Na]$^+$): 394.1544, found: 394.1548. The product was analyzed by HPLC to determine the enantiomeric excess: 97% ee (Chiralpak AD-H, *i*-propanol /hexane = 10/90, flow rate 1.0 mL/min, λ = 254 nm); t_r = 7.65 and 9.63 min.

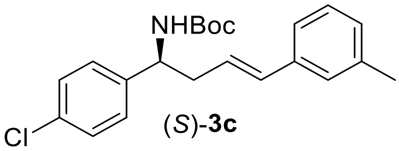

(*S*)-**3c**

***tert*-butyl (*S*,*E*)-(1-(4-chlorophenyl)-4-(*m*-tolyl)but-3-en-1-yl)carbamate (Figure 5)** Following **General Reaction Procedure A:** Yield (79%); white solid, m.p. 123-125 °C; $[\alpha]^{30}_D$ = -28.1 (*c* 0.31, CH_2Cl_2); ^{1}H NMR (400 MHz, Chloroform-*d*) δ 7.33 – 7.30 (m, 2H), 7.25 – 7.20 (m, 3H), 7.14 – 7.09 (m, 2H), 7.04 (d, *J* = 7.6 Hz, 2H), 6.42 (d, *J* = 16.0 Hz, 1H), 6.04 – 5.96 (m, 1H), 4.91 (brs, 1H), 4.79 (brs, 1H), 2.65 – 2,60 (m, 2H), 2.33 (s, 3H), 1.39 (s, 9H). ^{13}C NMR (101 MHz, $CDCl_3$) δ 155.1, 141.0, 138.1, 136.8, 133.6, 132.8, 128.6, 128.4, 128.3, 127.5, 126.9, 124.6, 123.3, 79.8, 53.7, 40.3, 28.3, 21.4. HRMS (ESI+) Calcd. For $C_{22}H_{26}ClNO_2Na$ ([M+Na]$^+$): 394.1544, found: 394.1551. The product was analyzed by HPLC to determine the enantiomeric excess: 97% ee (Chiralpak OD-H, *i*-propanol /hexane = 10/90, flow rate 1.0 mL/min, λ = 254 nm); t_r = 6.62 and 7.85 min.

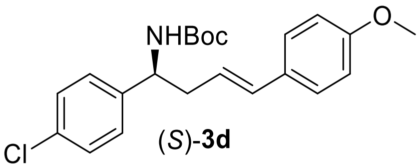

(*S*)-**3d**

***tert*-butyl (*S*,*E*)-(1-(4-chlorophenyl)-4-(4-methoxyphenyl)but-3-en-1-yl) carbamate (Figure 5)** Following **General Reaction Procedure A:** Yield (89%); white solid, m.p. 140-142 °C; $[\alpha]^{30}_D$ = -23.3 (*c* 0.70, CH_2Cl_2); ^{1}H NMR (400 MHz, $CDCl_3$) δ 7.33 – 7.28 (m, 2H), 7.26 – 7.20 (m, 4H), 6.83 (d, *J* = 8.8 Hz, 2H), 6.39 (d, *J* = 16.0 Hz, 1H), 5.96 – 5.78 (m, 1H), 4.94 (s, 1H), 4.87 – 4.67 (m, 1H), 3.84 – 3.76 (m, 3H), 2.60 (m, 2H), 1.39 (s, 9H). ^{13}C NMR (101 MHz, $CDCl_3$) δ 159.0, 155.1, 141.0, 132.9, 132.7, 129.7, 128.6, 127.5, 127.3, 122.5, 113.9, 79.7, 55.2, 53.8, 40.3, 28.3. HRMS (ESI+) Calcd. For $C_{22}H_{26}ClNO_3Na$ ([M+Na]$^+$): 410.1493, found: 410.1497. The product was

analyzed by HPLC to determine the enantiomeric excess: 95% ee (Chiralpak OD-H, *i*-propanol /hexane = 10/90, flow rate 1.0 mL/min, λ = 254 nm); t_r = 7.21 and 8.95 min.

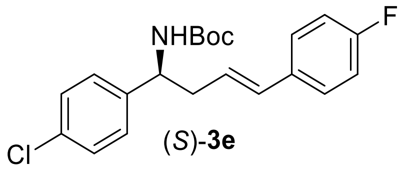

(*S*)-**3e**

***tert*-butyl (*S,E*)-(1-(4-chlorophenyl)-4-(4-fluorophenyl)but-3-en-1-yl)carbamate (Figure 5)** Following **General Reaction Procedure A:** Yield (81%); white solid, m.p. 125-127 °C; $[\alpha]^{30}_{D}$ = -26.4 (*c* 0.81, CH_2Cl_2); ^{1}H NMR (400 MHz, CDC13) δ 7.37 – 7.21 (m, 6H), 7.00 – 6.95 (m, 2H), 6.40 (d, *J* = 16.0 Hz, 1H), 6.06 – 5.86 (m, 1H), 4.92 – 4.90 (m, 1H), 4.79 (brs, 1H), 2.62 (m, 2H), 1.39 (s, 9H). ^{13}C NMR (101 MHz, $CDCl_3$) δ 162.2 (d, *J* = 245 Hz), 155.1, 140.8, 133.1, 132.8, 132.2, 128.7, 127.6 (d, *J* = 8.0 Hz), 127.5, 124.6, 115.4 (d, *J* = 21.4 Hz), 79.8, 53.8, 40.3, 28.3. ^{19}F NMR (376 MHz, $CDCl_3$) δ -114.64. HRMS (ESI+) Calcd. For $C_{21}H_{23}ClFNO_2Na$ ($[M+Na]^+$): 398.1299, found: 398.1302. The product was analyzed by HPLC to determine the enantiomeric excess: 95% ee (Chiralpak OD-H, *i*-propanol /hexane = 10/90, flow rate 1.0 mL/min, λ = 254 nm); t_r = 5.54 and 6.33 min.

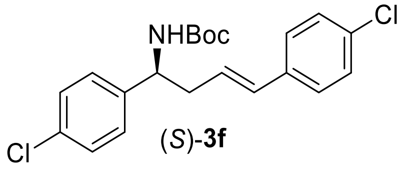

(*S*)-**3f**

***tert*-butyl (*S,E*)-(1,4-bis(4-chlorophenyl)but-3-en-1-yl)carbamate (Figure 5)** Following **General Reaction Procedure A:** Yield (81%); white solid, m.p. 128-130 °C; $[\alpha]^{30}_{D}$ = -19.5 (*c* 1.10, CH_2Cl_2); ^{1}H NMR (400 MHz, $CDCl_3$) δ 7.35 – 7.20 (m, 8H), 6.39 (d, *J* = 16.0 Hz, 1H), 6.09 – 5.93 (m, 1H), 4.94 – 4.92 (m, 1H), 4.79 (brs, 1H), 2.63 (m, 2H), 1.39 (s, 9H). ^{13}C NMR (101 MHz, $CDCl_3$) δ 155.0, 140.7, 135.4, 133.0, 132.9, 132.2, 128.7, 128.6, 127.5, 127.3, 125.7, 79.8, 53.7, 40.3, 27.4. HRMS (ESI+) Calcd. For $C_{21}H_{23}Cl_2NO_2Na$ ($[M+Na]^+$): 414.0998, found: 414.1004. The product was analyzed by HPLC to determine the enantiomeric excess: 95% ee (Chiralpak OD-H, *i*-

propanol /hexane = 10/90, flow rate 1.0 mL/min, λ = 254 nm); t_r = 5.75 and 6.73 min.

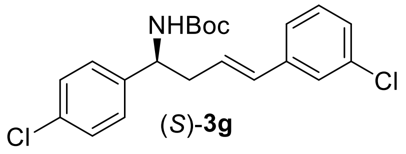

(*S*)-**3g**

***tert*-butyl (*S,E*)-(4-(3-chlorophenyl)-1-(4-chlorophenyl)but-3-en-1-yl)carbamate (Figure 5)** Following **General Reaction Procedure A:** Yield (78%); white solid, m.p. 118-120 °C; $[\alpha]^{30}_D$ = -21.7 (*c* 0.94, CH_2Cl_2); ^{1}H NMR (400 MHz, $CDCl_3$) δ 7.34 – 7.26 (m, 3H), 7.25 – 7.12 (m, 5H), 6.37 (d, *J* = 16.0 Hz, 1H), 6.14 – 5.95 (m, 1H), 4.93 (m, 1H), 4.79 (brs, 1H), 2.63 (m, 2H), 1.40 (s, 9H). ^{13}C NMR (101 MHz, $CDCl_3$) δ 155.0, 140.6, 138.8, 134.4, 132.9, 132.1, 129.7, 128.7, 127.5, 127.3, 126.6, 126.0, 124.3, 79.8, 53.7, 40.2, 28.2. HRMS (ESI+) Calcd. For $C_{21}H_{23}Cl_2NO_2Na$ ($[M+Na]^+$): 414.0998, found: 414. 0999. The product was analyzed by HPLC to determine the enantiomeric excess: 96% ee (Chiralpak OD-H, *i*-propanol /hexane = 10/90, flow rate 1.0 mL/min, λ = 254 nm); t_r = 6.69 and 7.34 min.

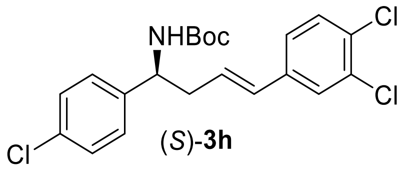

(*S*)-**3h**

***tert*-butyl (*S,E*)- (1-(4-chlorophenyl)-4-(3,4-dichlorophenyl)but-3-en-1-yl)carbamate (Figure 5)** Following **General Reaction Procedure A:** Yield (79%); white solid, m.p. 85-87 °C; $[\alpha]^{30}_D$ = -24.4 (*c* 0.35, CH_2Cl_2); ^{1}H NMR (400 MHz, $CDCl_3$) δ 7.39 – 7.30 (m, 4H), 7.22 (d, *J* = 8.4 Hz, 2H), 7.11 (dd, *J* = 8.5, 2.0 Hz, 1H), 6.34 (d, *J* = 16.0 Hz, 1H), 6.12 – 5.98 (m, 1H), 4.85 – 4.80 (m, 2H), 2.64 (m, 2H), 1.40 (s, 9H). ^{13}C NMR (101 MHz, $CDCl_3$) δ 155.0, 140.5, 137.0, 133.1, 132.6, 131.1, 131.0, 130.4, 128.8, 127.8, 127.6, 127.3, 125.3, 79.9, 53.7, 40.3, 28.3. HRMS (ESI+) Calcd. For $C_{21}H_{23}Cl_3NO_2Na$ ($[M+Na]^+$): 448.0608, found: 448.0611. The product was analyzed by HPLC to determine the enantiomeric excess: 95% ee (Chiralpak OD-H, *i*-propanol /hexane = 10/90, flow rate 1.0 mL/min, λ = 254 nm); t_r = 6.49 and 8.21 min.

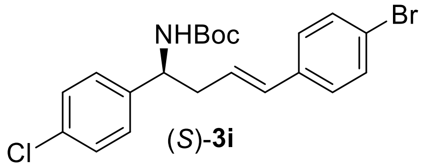
(*S*)-**3i**

***tert*-butyl (*S,E*)- (4-(4-bromophenyl)-1-(4-chlorophenyl)but-3-en-1-yl)carbamate (Figure 5)** Following **General Reaction Procedure A:** Yield (90%); white solid, m.p. 148-150 °C; $[\alpha]^{30}_{D}$ = -19.7 (*c* 1.10, CH_2Cl_2); ^{1}H NMR (400 MHz, $CDCl_3$) δ 7.40 (d, *J* = 8.4 Hz, 2H), 7.31 (d, *J* = 8.4 Hz, 2H), 7.22 (d, *J* = 8.4 Hz, 2H), 7.15 (d, *J* = 8.4 Hz, 2H), 6.37 (d, *J* = 16.0 Hz, 1H), 6.18 – 5.92 (m, 1H), 4.93 – 4.90 (m, 1H), 4.78 (brs, 1H), 2.62 (m, 2H), 1.39 (s, 9H). ^{13}C NMR (101 MHz, $CDCl_3$) δ 155.0, 140.7, 135.8, 132.9, 132.2, 131.6, 128.7, 127.6, 127.5, 125.9, 121.1, 79.8, 53.7, 40.3, 28.3. HRMS (ESI+) Calcd. For $C_{21}H_{23}ClBrNO_2Na$ ($[M+Na]^+$): 458.0493, found: 458.0501. The product was analyzed by HPLC to determine the enantiomeric excess: 94% ee (Chiralpak OD-H, *i*-propanol /hexane = 10/90, flow rate 1.0 mL/min, λ = 254 nm); t_r = 6.29 and 7.52 min.

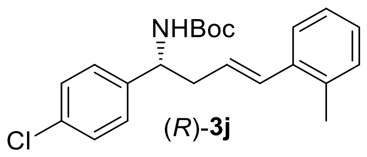
(*R*)-**3j**

***tert*-butyl (*R,E*)-(1-(4-chlorophenyl)-4-(*o*-tolyl)but-3-en-1-yl)carbamate (Figure 5)** Following **General Reaction Procedure D:** Yield (89%); white solid, m.p. 123-125 °C; $[\alpha]^{30}_{D}$ = 25.9 (*c* 0.84, CH_2Cl_2); ^{1}H NMR (400 MHz, Chloroform-*d*) δ 7.36 – 7.27 (m, 3H), 7.24 – 7.21 (m, 2H), 7.15 – 7.11 (m, 3H), 6.62 (d, *J* = 15.6 Hz, 1H), 5.92 – 5.84 (m, 1H), 4.93 (m, 1H), 4.79 (brs, 1H), 2.68 – 2.63 (m, 2H), 2.29 (s, 3H), 1.40 (s, 3H). ^{13}C NMR (101 MHz, $CDCl_3$) δ 155.0, 140.9, 136.1, 135.1, 132.8, 131.5, 130.2, 128.6, 127.6, 127.4, 126.2, 126.0, 125.6, 79.7, 53.7, 40.5, 28.3, 19.8. HRMS (ESI+) Calcd. For $C_{22}H_{26}ClNO_2Na$ ($[M+Na]^+$): 394.1544, found: 394.1551. The product was analyzed by HPLC to determine the enantiomeric excess: 94% ee (Chiralpak OD-H, *i*-propanol /hexane = 10/90, flow rate 1.0 mL/min, λ = 254 nm); t_r = 6.09 and 6.91 min.

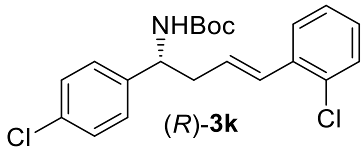

(*R*)-**3k**

***tert*-butyl (*R,E*)-(4-(2-chlorophenyl)-1-(4-chlorophenyl)but-3-en-1-yl)carbamate (Figure 5)** Following **General Reaction Procedure D:** Yield (81%); white solid, m.p. 100-102 °C; $[\alpha]^{30}_{D} = 31.2$ (*c* 0.24, CH_2Cl_2); ^1H NMR (400 MHz, Chloroform-*d*) δ 7.46 – 7.37 (m, 1H), 7.37 – 7.29 (m, 3H), 7.25 – 7.20 (m, 2H), 7.19 - 7.14 (m, 2H), 6.82 (d, *J* = 16.0 Hz, 1H), 6.05 – 5.96 (m, 1H), 4.92 (m, 1H), 4.82 (brs, 1H), 2.70 – 2.86 (m, 2H), 1.40 (s, 9H). ^{13}C NMR (101 MHz, $CDCl_3$) δ 155.1, 140.7, 135.1, 132.9, 132.7, 129.7, 129.6, 128.7, 128.5, 128.0, 127.8, 127.6, 126.8, 79.8, 53.6, 40.4, 28.3. HRMS (ESI+) Calcd. For $C_{21}H_{23}Cl_2NO_2Na$ ($[M+Na]^+$): 414.0998, found: 414.1003. The product was analyzed by HPLC to determine the enantiomeric excess: 95% ee (Chiralpak IE-H, *i*-propanol /hexane = 5/95, flow rate 1.0 mL/min, λ = 254 nm); t_r = 7.85 and 8.34 min.

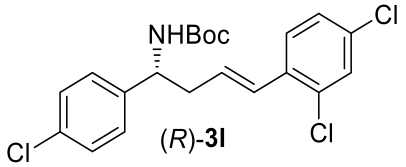

(*R*)-**3l**

***tert*-butyl (*R,E*)-(1-(4-chlorophenyl)-4-(2,4-dichlorophenyl)but-3-en-1-yl) carbamate (Figure 5)** Following **General Reaction Procedure D:** Yield (85%); white solid, m.p. 104-106 °C; $[\alpha]^{30}_{D} = 16.1$ (*c* 0.44, CH_2Cl_2); ^1H NMR (400 MHz, Chloroform-*d*) δ 7.39 – 7.29 (m, 4H), 7.23 (d, *J* = 8.4 Hz, 2H), 7.16 (dd, *J* = 8.4, 2.0 Hz, 1H), 6.74 (d, *J* = 15.6 Hz, 1H), 6.05 – 5.96 (m, 1H), 4.89 – 4.81 (m, 2H), 2.70 – 2.65 (m, 2H), 1.39 (s, 9H). ^{13}C NMR (101 MHz, $CDCl_3$) δ 155.1, 140.6, 133.7, 133.4, 133.1, 133.0, 129.3, 129.0, 128.8, 128.6, 127.6, 127.5, 127.2, 79.9, 53.6, 40.5, 28.3. HRMS (ESI+) Calcd. For $C_{21}H_{23}Cl_3NO_2Na$ ($[M+Na]^+$): 448.0608, found: 448.0616. The product was analyzed by HPLC to determine the enantiomeric excess: 88% ee (Chiralpak OD-H, *i*-propanol /hexane = 10/90, flow rate 1.0 mL/min, λ = 254 nm); t_r = 5.27 and 6.04 min.

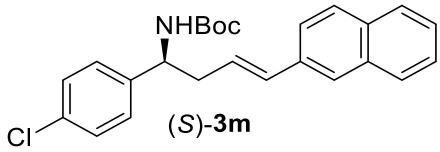

(*S*)-**3m**

***tert*-butyl (*S,E*)-(1-(4-chlorophenyl)-4-(naphthalen-2-yl)but-3-en-1-yl)carbamate (Figure 5)** Following **General Reaction Procedure A:** Yield (77%); white solid, m.p. 145-147 °C; $[\alpha]^{30}_{D}$ = -24.3 (*c* 0.42, CH_2Cl_2); ^{1}H NMR (400 MHz, Chloroform-*d*) δ 7.88 – 7.71 (m, 3H), 7.65 (s, 1H), 7.52 – 7.48 (m, 1H), 7.46 – 7.42 (m, 2H), 7.34 – 7.31 (m, 2H), 7.26 – 7.24 (m, 2H), 6.61 (d, *J* = 16.0 Hz, 1H), 6.19 – 6.11 (m, 1H), 4.94 – 4.83 (m, 2H), 2.72 – 2.68 (m, 2H), 1.39 (s, 9H). ^{13}C NMR (101 MHz, $CDCl_3$) δ 155.1, 140.9, 134.3, 133.6, 133.5, 132.8, 128.7, 128.2, 127.9, 127.6, 127.6, 126.3, 125.9, 125.8, 125.3, 125.2, 123.4, 79.8, 53.8, 40.5, 28.3. HRMS (ESI+) Calcd. For $C_{25}H_{26}ClNO_2Na$ ($[M+Na]^+$): 430.1544, found: 430.1552. The product was analyzed by HPLC to determine the enantiomeric excess: 96% ee (Chiralpak AD-H, *i*-propanol /hexane = 10/90, flow rate 1.0 mL/min, λ = 254 nm); t_r = 12.99 and 18.27 min.

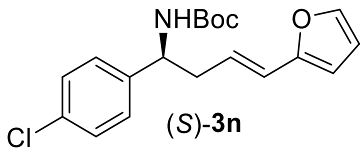

(*S*)-**3n**

***tert*-butyl (*S,E*)-(1-(4-chlorophenyl)-4-(furan-2-yl)but-3-en-1-yl)carbamate (Figure 5)** Following **General Reaction Procedure A:** Yield (82%); white solid, m.p. 116-118 °C; $[\alpha]^{30}_{D}$ = -22.9 (*c* 0.70, CH_2Cl_2); ^{1}H NMR (400 MHz, $CDCl_3$) δ 7.35 – 7.27 (m, 3H), 7.22 (d, *J* = 8.4 Hz, 2H), 6.34 (dd, *J* = 3.2, 1.6 Hz, 1H), 6.26 (d, *J* = 16.0 Hz, 1H), 6.16 (d, *J* = 3.2 Hz, 1H), 6.01 – 5.89 (m, 1H), 4.91 (m, 1H), 4.77 (brs, 1H), 2.60 (m, 2H), 1.40 (s, 9H). ^{13}C NMR (101 MHz, $CDCl_3$) δ 155.0, 152.4, 141.7, 140.8, 132.8, 128.6, 127.5, 123.5, 121.9, 111.2, 107.2, 79.8, 53.7, 40.1, 28.3. HRMS (ESI+) Calcd. For $C_{19}H_{22}ClNO_3Na$ ($[M+Na]^+$): 370.1180, found: 370.1186. The product was analyzed by HPLC to determine the enantiomeric excess: 91% ee (Chiralpak OD-H, *i*-propanol /hexane = 10/90, flow rate 1.0 mL/min, λ = 254 nm); t_r = 6.51 and 7.07 min.

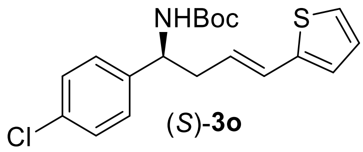

(*S*)-**3o**

***tert*-butyl (*S,E*)-(1-(4-chlorophenyl)-4-(thiophen-2-yl)but-3-en-1-yl)carbamate (Figure 5)** Following **General Reaction Procedure A:** Yield (79%); white solid, m.p. 136-138 °C; $[\alpha]^{30}_{D}$ = -18.3 (*c* 0.58, CH_2Cl_2); ^{1}H NMR (400 MHz, $CDCl_3$) δ 7.31 (d, *J* = 8.5 Hz, 2H), 7.21 (d, *J* = 8.4 Hz, 2H), 7.11 (d, *J* = 5.1 Hz, 1H), 6.94 (dd, *J* = 5.1, 3.5 Hz, 1H), 6.88 (d, *J* = 3.3 Hz, 1H), 6.56 (d, *J* = 15.7 Hz, 1H), 5.96 – 5.76 (m, 1H), 4.91 (s, 1H), 4.77 (s, 1H), 2.59 (s, 2H), 1.40 (s, 9H). ^{13}C NMR (101 MHz, $CDCl_3$) δ 155.0, 142.0, 140.8, 132.8, 128.7, 127.5, 127.3, 126.6, 125.2, 124.6, 123.9, 79.8, 53.7, 40.1, 28.3. HRMS (ESI+) Calcd. For $C_{19}H_{22}ClNO_2SNa$ ($[M+Na]^+$): 386.0952, found: 386.0955. The product was analyzed by HPLC to determine the enantiomeric excess: 96% ee (Chiralpak OD-H, *i*-propanol /hexane = 10/90, flow rate 1.0 mL/min, λ = 254 nm); t_r = 7.54 and 8.85 min.

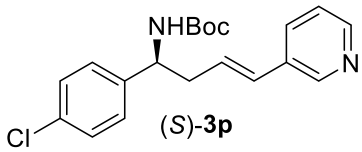

(*S*)-**3p**

***tert*-butyl (*S,E*)-(1-(4-chlorophenyl)-4-(pyridin-3-yl)but-3-en-1-yl)carbamate (Figure 5)** Following **General Reaction Procedure A:** Yield (64%); white solid, m.p. 115-117 °C; $[\alpha]^{30}_{D}$ = -22.2 (*c* 0.36, CH_2Cl_2); ^{1}H NMR (400 MHz, $CDCl_3$) δ 8.48 (d, *J* = 26.0 Hz, 2H), 7.61 (d, *J* = 8.0 Hz, 1H), 7.32 (d, *J* = 8.4 Hz, 2H), 7.25 – 7.22 (m, 3H), 6.43 (d, *J* = 16.0 Hz, 1H), 6.16 – 6.08 (m, 1H), 4.98 – 4.96 (m, 1H), 4.81 (brs, 1H), 2.67 (m, 2H), 1.39 (s, 9H). ^{13}C NMR (101 MHz, $CDCl_3$) δ 155.0, 148.5, 148.1, 140.5, 133.0, 132.6, 129.8, 128.8, 127.7, 127.6, 123.4, 79.8, 53.7, 40.4, 28.3. HRMS (ESI+) Calcd. For $C_{20}H_{23}ClN_2O_2Na$ ($[M+Na]^+$): 381.1340, found: 381.1345. The product was analyzed by HPLC to determine the enantiomeric excess: 90% ee (Chiralpak OD-H, *i*-propanol /hexane = 10/90, flow rate 1.0 mL/min, λ = 254 nm); t_r = 11.97 and 17.69 min.

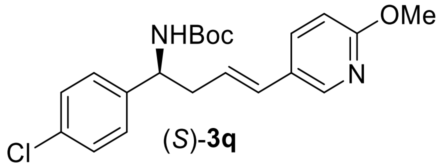

(*S*)-**3q**

***tert*-butyl (*S,E*)-(1-(4-chlorophenyl)-4-(6-methoxypyridin-3-yl)but-3-en-1-yl)carbamate (Figure 5)** Following **General Reaction Procedure A:** Yield (86%); white solid, m.p. 120-122 °C; $[\alpha]^{30}_{D}$ = -21.1 (*c* 0.90, CH_2Cl_2); ^{1}H NMR (400 MHz, $CDCl_3$) δ 8.02 (d, *J* = 2.4 Hz, 1H), 7.56 (dd, *J* = 8.8, 2.4 Hz, 1H), 7.31 (d, *J* = 8.4 Hz, 2H), 7.22 (d, *J* = 8.4 Hz, 2H), 6.68 (d, *J* = 8.8 Hz, 1H), 6.37 (d, *J* = 16.0 Hz, 1H), 6.02 – 5.85 (m, 1H), 4.99 – 6.97 (m, 1H), 4.78 (brs, 1H), 3.92 (s, 3H), 2.62 (m, 2H), 1.39 (s, 9H). ^{13}C NMR (101 MHz, $CDCl_3$) δ 163.4, 155.1, 145.1, 140.8, 135.3, 132.8, 129.4, 128.6, 127.5, 126.1, 124.3, 110.8, 79.7, 53.8, 53.4, 40.3, 28.2. HRMS (ESI+) Calcd. For $C_{21}H_{25}ClN_2O_2Na$ ([M+Na]$^+$): 411.1446, found: 411.1449. The product was analyzed by HPLC to determine the enantiomeric excess: 96% ee (Chiralpak OD-H, *i*-propanol /hexane = 10/90, flow rate 1.0 mL/min, λ = 254 nm); t_r = 7.28 and 8.66 min.

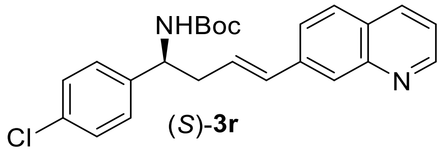

(*S*)-**3r**

***tert*-butyl (*S,E*)-(1-(4-chlorophenyl)-4-(quinolin-7-yl)but-3-en-1-yl)carbamate (Figure 5)** Following **General Reaction Procedure A:** Yield (65%); white solid, m.p. 170-172 °C; $[\alpha]^{30}_{D}$ = -10.0 (*c* 0.90, CH_2Cl_2); ^{1}H NMR (400 MHz, Chloroform-*d*) δ 8.85 (s, 1H), 8.10 (d, *J* = 8.4 Hz, 1H), 8.02 (d, *J* = 8.8 Hz, 1H), 7.74 (d, *J* = 9.2 Hz, 1H), 7.62 (s, 1H), 7.43 – 7.36 (m, 2H), 7.34 (d, *J* = 8.4 Hz, 2H), 7.30 – 7.22 (m, 2H), 6.62 (d, *J* = 16.0 Hz, 1H), 6.24 – 6.17 (m, 1H), 4.96 – 4.84 (m, 2H), 2.74 – 2.68 (m, 2H), 1.39 (s, 9H). ^{13}C NMR (101 MHz, $CDCl_3$) δ 155.1, 150.1, 147.9, 140.7, 135.9, 135.1, 132.9, 132.7, 129.6, 128.7, 128.4, 127.6, 127.1, 126.7, 125.3, 121.4, 79.8, 53.8, 40.4, 28.3. HRMS (ESI+) Calcd. For $C_{24}H_{25}ClN_2O_2Na$ ([M+Na]$^+$): 431.1497, found: 431.1498. The product was analyzed by HPLC to determine the enantiomeric excess: 90% ee (Chiralpak OD-H, *i*-propanol /hexane = 10/90, flow rate 1.0 mL/min, λ = 254 nm); t_r = 17.67 and 21.72 min.

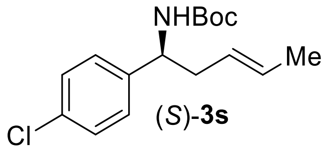

(*S*)-**3s**

***tert*-butyl (*S,E*)-(1-(4-chlorophenyl)pent-3-en-1-yl)carbamate (Figure 5)** Following **General Reaction Procedure A:** Yield (75%); white solid, m.p. 82-84 °C; $[\alpha]^{30}_{D}$ = -4.9 (*c* 0.42, CH_2Cl_2); ^{1}H NMR (400 MHz, $CDCl_3$) δ 7.30 – 7.26 (m, 2H), 7.19 – 7.17 (m, 2H), 5.56 – 5.49 (m, 1H), 5.29 – 5.23 (m, 1H), 4.86 (m, 1H), 4.63 (brs, 1H), 2.39 (m, 2H), 1.64 (d, *J* = 6.4 Hz, 3H), 1.41 (s, 9H). ^{13}C NMR (101 MHz, $CDCl_3$) δ 155.1, 141.3, 132.5, 129.3, 128.5, 127.5, 125.8, 79.6, 53.7, 40.0, 28.3, 18.0. HRMS (ESI+) Calcd. For $C_{16}H_{22}ClNO_2Na$ ($[M+Na]^+$): 318.1231, found: 318.1224. The product was analyzed by HPLC to determine the enantiomeric excess: 91% ee (Chiralpak OD-H, *i*-propanol /hexane = 10/90, flow rate 1.0 mL/min, λ = 254 nm); t_r = 4.97 and 5.42 min.

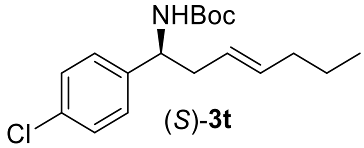

(*S*)-**3t**

***tert*-butyl (*S,E*)-(1-(4-chlorophenyl)hept-3-en-1-yl)carbamate (Figure 5)** Following **General Reaction Procedure A:** Yield (51%); white solid, m.p. 101-103 °C; $[\alpha]^{30}_{D}$ = -4.2 (*c* 0.12, CH_2Cl_2); ^{1}H NMR (400 MHz, $CDCl_3$) δ 7.29 (d, *J* = 8.4 Hz, 2H), 7.18 (d, *J* = 8.4 Hz, 2H), 5.49 (dt, *J* = 13.8, 6.8 Hz, 1H), 5.31 – 5.15 (m, 1H), 4.86 (m, 1H), 4.63 (brs, 1H), 2.40 – 2.38 (m, 2H), 2.03 – 1.89 (m, 2H), 1.48 – 1.32 (m, 11H), 0.86 (t, *J* = 7.2 Hz, 3H). ^{13}C NMR (101 MHz, $CDCl_3$) δ 155.1, 138.0, 134.8, 132.6, 128.5, 127.6, 124.8, 79.6, 53.8, 40.0, 34.6, 28.3, 22.4, 13.6. HRMS (ESI+) Calcd. For $C_{18}H_{26}ClNO_2Na$ ($[M+Na]^+$): 346.1544, found: 346.1549. The product was analyzed by HPLC to determine the enantiomeric excess: 94% ee (Chiralpak OD-H, *i*-propanol /hexane = 10/90, flow rate 1.0 mL/min, λ = 254 nm); t_r = 4.97 and 5.42 min.

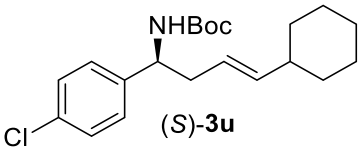

(*S*)-**3u**

***tert*-butyl (*S,E*)-(1-(4-chlorophenyl)-4-cyclohexylbut-3-en-1-yl)carbamate (Figure 5)** Following **General Reaction Procedure A:** Yield (67%); white solid, m.p. 119-121 °C; $[\alpha]^{30}_{D}$ = -8.1 (*c* 0.42, CH_2Cl_2); ^1H NMR (400 MHz, $CDCl_3$) δ 7.32 – 7.25 (m, 2H), 7.17 (d, *J* = 8.4 Hz, 2H), 5.44 (dd, *J* = 15.2, 6.8 Hz, 1H), 5.29 – 5.09 (m, 1H), 4.85 (m, 1H), 4.62 (brs, 1H), 2.38 – 2.36 (m, 2H), 1.92 – 1.88 (m, 1H), 1.72 – 1.63 (m, 4H), 1.41 (s, 9H), 1.25 – 1.21 (m, 4H), 1.05 – 0.99 m, 2H). ^{13}C NMR (101 MHz, $CDCl_3$) δ 155.1, 141.4, 141.0, 132.5, 128.5, 127.6, 121.0, 79.6, 53.8, 40.6, 40.0, 32.9, 28.3, 26.1, 25.9. HRMS (ESI+) Calcd. For $C_{21}H_{30}ClNO_2Na$ ($[M+Na]^+$): 386.1857, found: 386.1549. The product was analyzed by HPLC to determine the enantiomeric excess: 98% ee (Chiralpak OD-H, *i*-propanol /hexane = 3/97, flow rate 1.0 mL/min, λ = 254 nm); t_r = 5.04 and 5.75 min.

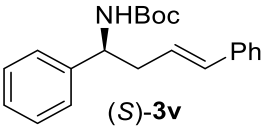

(*S*)-**3v**

***tert*-butyl (*S,E*)-(1,4-diphenylbut-3-en-1-yl)carbamate (Figure 6)** Following **General Reaction Procedure A:** Yield (82%); white solid, m.p. 100-102 °C; $[\alpha]^{30}_{D}$ = -17.7 (*c* 0.43, CH_2Cl_2); ^1H NMR (400 MHz, $CDCl_3$) δ 7.37 – 7.26 (m, 9H), 7.23 – 7.18 mz, 1H), 6.45 (d, *J* = 16.0 Hz, 1H), 6.06 (dt, *J* = 16.0, 7.2 Hz, 1H), 4.93 – 4.83 (m, 2H), 2.68 (m, 2H), 1.40 (s, 9H). ^{13}C NMR (101 MHz, $CDCl_3$) δ 155.2, 142.2, 137.1, 133.1, 128.5, 128.5, 127.3, 127.2, 126.2, 126.1, 125.5, 79.5, 54.3, 40.5, 28.3. HRMS (ESI+) Calcd. For $C_{21}H_{25}NO_2Na$ ($[M+Na]^+$): 346.1778, found: 346.1778. The product was analyzed by HPLC to determine the enantiomeric excess: 97% ee (Chiralpak AD-H, *i*-propanol /hexane = 10/90, flow rate 1.0 mL/min, λ = 254 nm); t_r = 7.97 and 9.33 min.

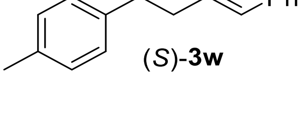

(*S*)-**3w**

***tert*-butyl (*S,E*)-(4-phenyl-1-(*p*-tolyl)but-3-en-1-yl)carbamate (Figure 6)** Following **General Reaction Procedure B:** Yield (79%); white solid, m.p. 96-98 °C; $[\alpha]^{30}_{D}$ = -24.4 (*c* 0.72, CH_2Cl_2); ^1H NMR (400 MHz, $CDCl_3$) δ 7.32 – 7.25 (m, 4H), 7.22 – 7.12 (m, 5H), 6.45 (d, *J* = 16.0 Hz, 1H), 6.10 – 5.98 (m, 1H), 4.90 (m, 1H), 4.79 (brs, 1H), 2.66 (m, 2H), 2.33 (s, 3H), 1.39 (s, 9H). ^{13}C NMR (101 MHz, $CDCl_3$) δ 155.1, 139.2, 137.2, 136.7, 132.9, 129.2, 128.4, 127.2, 126.10, 126.07, 125.6, 79.4, 54.0, 40.5, 28.3, 21.0. HRMS (ESI+) Calcd. For $C_{22}H_{27}NO_2Na$ ($[M+Na]^+$): 360.1934, found: 360.1934. The product was analyzed by HPLC to determine the enantiomeric excess: 97% ee (Chiralpak AD-H, *i*-propanol /hexane = 10/90, flow rate 1.0 mL/min, λ = 254 nm); t_r = 4.18 and 8.49 min.

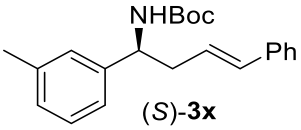

(*S*)-**3x**

***tert*-butyl (*S,E*)-(4-phenyl-1-(*m*-tolyl)but-3-en-1-yl)carbamate (Figure 6)** Following **General Reaction Procedure B:** Yield (81%); white solid, m.p. 86-88 °C; $[\alpha]^{30}_{D}$ = -18.7 (*c* 0.31, CH_2Cl_2); ^1H NMR (400 MHz, $CDCl_3$) δ 7.32 – 7.26 (m, 4H), 7.24 – 7.18 (m, 2H), 7.12 – 7.05 (m, 3H), 6.45 (d, *J* = 16.0 Hz, 1H), 6.16 – 5.99 (m, 1H), 4.92 (m, 1H), 4.79 (brs, 1H), 2.66 (m, 2H), 2.35 (s, 3H), 1.40 (s, 9H). ^{13}C NMR (101 MHz, $CDCl_3$) δ 155.4, 155.2, 142.2, 138.1, 137.2, 133.0, 128.4, 127.9, 127.2, 127.0, 126.1, 125.6, 123.1, 79.44, 54.28, 40.53, 28.26, 21.47. HRMS (ESI+) Calcd. For $C_{22}H_{27}NO_2Na$ ($[M+Na]^+$): 360.1934, found: 360.1939. The product was analyzed by HPLC to determine the enantiomeric excess: 98% ee (Chiralpak AD-H, *i*-propanol /hexane = 10/90, flow rate 1.0 mL/min, λ = 254 nm); t_r = 6.51 and 7.57 min.

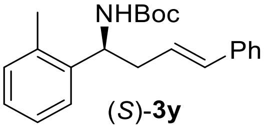

(*S*)-**3y**

***tert*-butyl (*S,E*)-(4-phenyl-1-(*o*-tolyl)but-3-en-1-yl)carbamate (Figure 6)** Following **General Reaction Procedure B:** Yield (71%); white solid, m.p. 81-83 °C; $[\alpha]^{30}_{D}$ = -

32.6 (c 0.74, CH_2Cl_2); ^{1}H NMR (400 MHz, $CDCl_3$) δ 7.33 – 7.25 (m, 4H), 7.24 – 7.18 (m, 3H), 7.16 – 7.12 (m, 2H), 6.46 (d, J = 16.0 Hz, 1H), 6.12 – 6.01 (m, 1H), 5.04 (brs, 1H), 4.93 – 4.91 (m, 1H), 2.63 (m, 2H), 2.40 (s, 3H), 1.39 (s, 9H). ^{13}C NMR (101 MHz, $CDCl_3$) δ 155.1, 140.3, 137.2, 135.3, 132.9, 130.7, 128.5, 127.2, 127.0, 126.1, 126.1, 125.6, 124.8, 79.4, 50.5, 39.5, 28.3, 19.2. HRMS (ESI+) Calcd. For $C_{22}H_{27}NO_2Na$ ($[M+Na]^+$): 360.1934, found: 360.1938. The product was analyzed by HPLC to determine the enantiomeric excess: 95% ee (Chiralpak AD-H, *i*-propanol /hexane = 10/90, flow rate 1.0 mL/min, λ = 254 nm); t_r = 7.09 and 9.71 min.

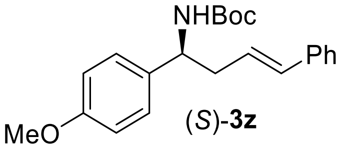

(*S*)-**3z**

***tert*-butyl (*S,E*)-(1-(4-methoxyphenyl)-4-phenylbut-3-en-1-yl)carbamate (Figure 6)**

Following **General Reaction Procedure B:** Yield (78%); white solid, m.p. 118-120 °C; $[\alpha]^{30}_D$ = -30.8 (c 0.43, CH_2Cl_2); ^{1}H NMR (400 MHz, $CDCl_3$) δ 7.31 – 7.26 (m, 4H), 7.24 – 7.18 (m, 3H), 6.87 (d, J = 8.87 Hz, 2H), 6.44 (d, J = 16.0 Hz, 1H), 6.10 – 5.97 (m, 1H), 4.88 – 4.76 (m, 2H), 3.80 (s, 3H), 2.66 (m, 2H), 1.39 (s, 9H). ^{13}C NMR (101 MHz, $CDCl_3$) δ 158.6, 155.1, 137.2, 134.3, 133.0, 128.5, 127.3, 127.2, 126.1, 125.7, 113.9, 79.4, 55.2, 53.8, 40.4, 28.3. HRMS (ESI+) Calcd. For $C_{22}H_{27}NO_3Na$ ($[M+Na]^+$): 376.1883, found: 376.1884. The product was analyzed by HPLC to determine the enantiomeric excess: 97% ee Chiralpak AD-H, *i*-propanol /hexane = 10/90, flow rate 1.0 mL/min, λ = 254 nm); t_r = 9.53 and 11.56 min.

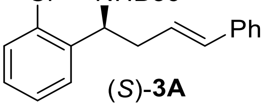

(*S*)-**3A**

***tert*-butyl (*S,E*)-(1-(2-chlorophenyl)-4-phenylbut-3-en-1-yl)carbamate (Figure 6)**

Following **General Reaction Procedure A:** Yield (73%); white solid, m.p. 100-102 °C; $[\alpha]^{30}_D$ = -15.0 (c 0.27, CH_2Cl_2); ^{1}H NMR (400 MHz, Chloroform-*d*) δ 7.40 – 7.30 (m, 6H), 7.24 – 7.17 (m, 3H), 6.47 (d, J = 16.0 Hz, 1H), 6.10 – 6.01 (m, 1H), 5.22 – 5.10

(m, 2H), 2.72 – 2.64 (m, 2H), 1.40 (s, 9H). ^{13}C NMR (101 MHz, $CDCl_3$) δ 154.9, 139.7, 137.0, 133.4, 132.5, 130.0, 128.5, 128.3, 127.4, 127.1, 126.9, 126.2, 125.1, 79.7, 51.8, 38.6, 28.3. HRMS (ESI+) Calcd. For $C_{21}H_{24}ClNO_2Na$ ($[M+Na]^+$): 380.1388, found: 380.1393. The product was analyzed by HPLC to determine the enantiomeric excess: 95% ee (Chiralpak OJ-H, *i*-propanol /hexane = 10/90, flow rate 1.0 mL/min, λ = 254 nm); t_r = 6.61 and 13.64 min..

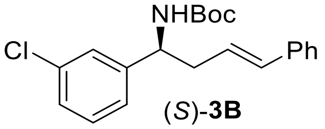

(*S*)-**3B**

***tert*-butyl (*S,E*)-(1-(3-chlorophenyl)-4-phenylbut-3-en-1-yl)carbamate (Figure 6)**

Following **General Reaction Procedure A:** Yield (83%); white solid, m.p. 99-101 °C; $[\alpha]^{30}_D$ = -18.2 (*c* 0.85, CH_2Cl_2); ^{1}H NMR (400 MHz, $CDCl_3$) δ 7.34 – 7.28 (m, 5H), 7.26 – 7.19 (m, 3H), 7.17 (d, *J* = 7.2 Hz, 1H), 6.46 (d, *J* = 16.0 Hz, 1H), 6.06 – 5.94 (m, 1H), 4.95 (m, 1H), 4.80 (brs, 1H), 2.65 – 2.62 (m, 2H), 1.40 (s, 9H). ^{13}C NMR (101 MHz, $CDCl_3$) δ 155.1, 144.6, 136.9, 134.4, 133.5, 129.8, 128.5, 127.4, 127.3, 126.3, 126.2, 124.8, 124.4, 79.8, 53.8, 40.3, 28.3. HRMS (ESI+) Calcd. For $C_{21}H_{24}ClNO_2Na$ ($[M+Na]^+$): 380.1388, found: 380.1389. The product was analyzed by HPLC to determine the enantiomeric excess: 96% ee (Chiralpak OJ-H, *i*-propanol /hexane = 10/90, flow rate 1.0 mL/min, λ = 254 nm); t_r = 7.84 and 9.94 min.

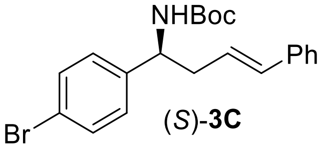

(*S*)-**3C**

***tert*-butyl (*S,E*)-(1-(4-bromophenyl)-4-phenylbut-3-en-1-yl)carbamate (Figure 6)**

Following **General Reaction Procedure A:** Yield (88%); white solid, m.p. 110-112 °C; $[\alpha]^{30}_D$ = -18.9 (*c* 0.71, CH_2Cl_2); ^{1}H NMR (400 MHz, $CDCl_3$) δ 7.46 (d, *J* = 8.4 Hz, 2H), 7.30 – 7.29 (m 4H), 7.25 – 7.20 (m, 1H), 7.17 (d, *J* = 8.4 Hz, 2H), 6.45 (d, *J* = 16.0 Hz, 1H), 6.09 – 5.95 (m, 1H), 4.92 (m, 1H), 4.77 (brs, 1H), 2.63 (m, 2H), 1.39 (s, 9H). ^{13}C NMR (101 MHz, $CDCl_3$) δ 155.1, 141.5, 136.9, 133.5, 131.6, 128.6, 127.9, 127.5, 126.2,

124.8, 120.9, 79.8, 53.8, 40.3, 28.3. HRMS (ESI+) Calcd. For $C_{21}H_{24}BrNO_2Na$ ([M+Na]$^+$): 424.0883, found: 424.0883. The product was analyzed by HPLC to determine the enantiomeric excess: 95% ee (Chiralpak OD-H, *i*-propanol /hexane = 10/90, flow rate 1.0 mL/min, λ = 254 nm); t_r = 7.51 and 8.25 min.

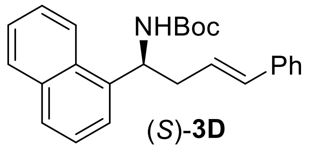

(*S*)-**3D**

***tert*-butyl (*S,E*)-(1-(naphthalen-1-yl)-4-phenylbut-3-en-1-yl)carbamate (Figure 6)**

Following **General Reaction Procedure A:** Yield (88%); white solid, m.p. 104-106 °C; $[\alpha]^{30}_D$ = -49.0 (*c* 0.52, CH_2Cl_2); ^{1}H NMR (400 MHz, Chloroform-*d*) δ 8.16 (d, *J* = 8.4 Hz, 1H), 7.88 (d, *J* = 8.4 Hz, 1H), 7.82 – 7.75 (m, 1H), 7.61 – 7.42 (m, 4H), 7.35 – 7.23 (m, 4H), 7.22 – 7.18 (m, 1H), 6.51 (d, *J* = 16.0 Hz, 1H), 6.15 – 6.08 (m, 1H), 5.73 – 5.63 (m, 1H), 5.08 – 4.99 (m, 1H), 2.96 – 2.70 (m, 2H), 1.41 (s, 9H). ^{13}C NMR (101 MHz, $CDCl_3$) δ 155.1, 137.7, 137.1, 134.0, 133.0, 130.8, 128.9, 128.5, 128.0, 127.3, 126.3, 126.1, 125.7, 125.7, 125.2, 123.1, 122.6, 79.6, 50.1, 39.4, 28.3. HRMS (ESI+) Calcd. For $C_{25}H_{27}NO_2Na$ ([M+Na]$^+$): 396.1934, found: 396.1938. The product was analyzed by HPLC to determine the enantiomeric excess: 95% ee (Chiralpak AD-H, *i*-propanol /hexane = 10/90, flow rate 1.0 mL/min, λ = 254 nm); t_r = 8.51 and 12.48 min.

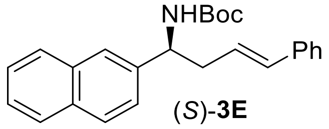

(*S*)-**3E**

***tert*-butyl (*S,E*)-(1-(naphthalen-2-yl)-4-phenylbut-3-en-1-yl)carbamate (Figure 6)**

Following **General Reaction Procedure A:** Yield (89%); white solid, m.p. 120-122 °C; $[\alpha]^{30}_D$ = -38.1 (*c* 0.57, CH_2Cl_2); ^{1}H NMR (400 MHz, $CDCl_3$) δ 7.89 – 7.78 (m, 3H), 7.73 (s, 1H), 7.52 – 7.39 (m, 3H), 7.32 – 7.24 (m, 4H), 7.22 – 7.19 (m, 1H), 6.48 (d, *J* = 16.0 Hz, 1H), 6.11 – 6.03 (m, 1H), 5.04 – 5.00 (m, 2H), 2.76 (m, 2H), 1.41 (s, 9H). ^{13}C NMR (101 MHz, $CDCl_3$) δ 155.2, 139.7, 137.0, 133.3, 133.2, 132.7, 128.5, 128.4, 127.9, 127.6, 127.3, 126.1, 126.1, 125.7, 125.4, 124.8, 124.5, 79.6, 54.4, 40.4, 28.3.

HRMS (ESI+) Calcd. For $C_{25}H_{27}NO_2Na$ ($[M+Na]^+$): 396.1934, found: 396.1929. The product was analyzed by HPLC to determine the enantiomeric excess: 96% ee (Chiralpak AD-H, *i*-propanol /hexane = 10/90, flow rate 1.0 mL/min, λ = 254 nm); t_r = 9.47 and 10.13 min.

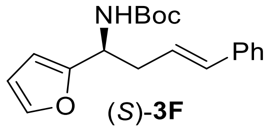

(*S*)-**3F**

***tert*-butyl (*S*,*E*)-(1-(furan-2-yl)-4-phenylbut-3-en-1-yl)carbamate (Figure 6)** Following **General Reaction Procedure B:** Yield (63%); white solid, m.p. 87-89 °C; $[\alpha]^{30}_D$ = -40.4 (*c* 0.67, CH_2Cl_2); ^{1}H NMR (400 MHz, $CDCl_3$) δ 7.36 – 7.18 (m, 6H), 6.45 (d, *J* = 16.0 Hz, 1H), 6.31 – 6.29 (m, 1H), 6.18 (d, *J* = 3.2 Hz, 1H), 6.12 – 6.04 (m, 1H), 5.02 – 4.93 (dm, 2H), 2.74 – 2.70 (m, 2H), 1.42 (s, 9H). ^{13}C NMR (101 MHz, $CDCl_3$) δ 155.1, 154.3, 141.7, 137.1, 133.1, 128.4, 127.2, 126.1, 125.1, 110.1, 105.9, 79.7, 48.4, 37.8, 28.2. HRMS (ESI+) Calcd. For $C_{19}H_{23}NO_3Na$ ($[M+Na]^+$): 336.1570, found: 336.1575. The product was analyzed by HPLC to determine the enantiomeric excess: 95% ee (Chiralpak OD-H, *i*-propanol /hexane = 10/90, flow rate 1.0 mL/min, λ = 254 nm); t_r = 5.18 and 5.81 min.

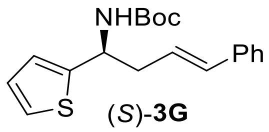

(*S*)-**3G**

***tert*-butyl (*S*,*E*)-(4-phenyl-1-(thiophen-2-yl)but-3-en-1-yl)carbamate (Figure 6)** Following **General Reaction Procedure B:** Yield (76%); white solid, m.p. 90-92 °C; $[\alpha]^{30}_D$ = -28.3 (*c* 0.29, CH_2Cl_2); ^{1}H NMR (400 MHz, Chloroform-*d*) δ 7.36 – 7.28 (m, 4H), 7.23 – 7.19 (m, 2H), 6.98 – 6.95 (m, 2H), 6.49 (d, *J* = 16.0 Hz, 1H), 6.19 – 6.10 (m, 1H), 5.12 (m, 1H), 4.88 (brs, 1H), 2.80 – 2.75 (m, 2H), 1.42 (s, 9H). ^{13}C NMR (101 MHz, $CDCl_3$) δ 155.0, 146.2, 137.1, 133.5, 128.5, 127.4, 126.8, 126.2, 125.0, 124.1, 124.0, 79.8, 50.2, 40.6, 28.3. HRMS (ESI+) Calcd. For $C_{19}H_{23}NO_2SNa$ ($[M+Na]^+$): 352.1342, found: 352.1344. The product was analyzed by HPLC to determine the

enantiomeric excess: 95% ee (Chiralpak AD-H, *i*-propanol /hexane = 10/90, flow rate 1.0 mL/min, λ = 254 nm); t_r = 8.55 and 10.31 min.

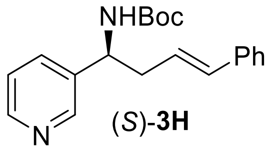

(*S*)-**3H**

***tert*-butyl (*S*,*E*)-(4-phenyl-1-(pyridin-3-yl)but-3-en-1-yl)carbamate (Figure 6)** Following **General Reaction Procedure A:** Yield (74%); white solid, m.p. 117-119 °C; $[\alpha]^{30}_{D}$ = -19.2 (*c* 0.91, CH_2Cl_2); ^1H NMR (400 MHz, $CDCl_3$) δ 8.67 – 8.47 (m, 2H), 7.62 – 7.59 (m, 1H), 7.33 – 7.22 (m, 6H), 6.46 (d, *J* = 16.0 Hz, 1H), 6.09 – 5.99 (m, 1H), 5.12 – 5.09 (m, 1H), 4.86 (brs, 1H), 2.68 (m, 2H), 1.39 (s, 9H). ^{13}C NMR (101 MHz, $CDCl_3$) δ 155.02, 148.56, 148.06, 137.77, 136.70, 133.83, 128.51, 127.51, 126.16, 126.12, 124.34, 123.34, 79.88, 53.39, 40.06, 28.22. HRMS (ESI+) Calcd. For $C_{20}H_{25}N_2O_2$ ($[M+Na]^+$): 325.1911, found: 325.1910. The product was analyzed by HPLC to determine the enantiomeric excess: 97% ee (Chiralpak OJ-H, *i*-propanol /hexane = 10/90, flow rate 1.0 mL/min, λ = 254 nm); t_r = 9.88 and 11.98 min.

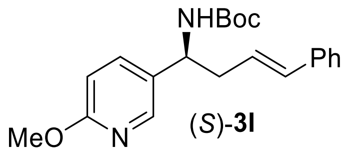

(*S*)-**3I**

***tert*-butyl (*S*,*E*)-(1-(6-methoxypyridin-3-yl)-4-phenylbut-3-en-1-yl)carbamate (Figure 6)** Following **General Reaction Procedure A:** Yield (76%); white solid, m.p. 120-122 °C; $[\alpha]^{30}_{D}$ = -22.9 (*c* 0.35, CH_2Cl_2); ^1H NMR (400 MHz, Chloroform-*d*) δ 8.11 (s, 1H), 7.51 (dd, *J* = 8.8, 2.4 Hz, 1H), 7.31 – 7.29 (m, 4H), 7.24 – 7.19 (m, 1H), 6.73 (d, *J* = 8.8 Hz, 1H), 6.46 (d, *J* = 16.0 Hz, 1H), 6.09 – 6.01 (m, 1H), 4.88 - 4.78 (m, 2H), 3.93 (s, 3H), 2.68 – 2.64 (m, 2H), 1.39 (s, 9H). ^{13}C NMR (101 MHz, $CDCl_3$) δ 163.4, 155.0, 144.8, 137.0, 136.9, 133.6, 130.5, 128.5, 127.4, 126.1, 124.8, 110.7, 53.5, 51.8, 40.1, 28.3. HRMS (ESI+) Calcd. For $C_{21}H_{26}N_2O_3Na$ ($[M+Na]^+$): 377.1836, found: 377.1840. The product was analyzed by HPLC to determine the enantiomeric excess: 95% ee (Chiralpak IA-H, *i*-propanol /hexane = 20/80, flow rate 1.0 mL/min, λ = 254

nm); t_r = 7.82 and 18.67 min.

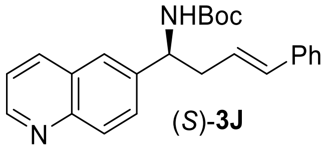
(*S*)-**3J**

***tert*-butyl (*S*,*E*)-(4-phenyl-1-(quinolin-6-yl)but-3-en-1-yl)carbamate (Figure 6)** Following **General Reaction Procedure A:** Yield (88%); white solid, m.p. 138-140 °C; $[\alpha]^{30}_D$ = -39.4 (*c* 0.82, CH_2Cl_2); ^{1}H NMR (400 MHz, $CDCl_3$) δ 8.90 – 8.88 (m, 1H), 8.13 – 8.09 (m, 2H), 7.75 – 7.64 (m, 2H), 7.40 – 7.36 (m, 1H), 7.30 – 7.24 (m, 4H), 7.23 – 7.20 (m, 1H), 6.48 (d, *J* = 16.0 Hz, 1H), 6.10 – 5.98 (m, 1H), 5.19 (m, 1H), 5.02 (brs, 1H), 2.76 (m, 2H), 1.41 (s, 9H). ^{13}C NMR (101 MHz, $CDCl_3$) δ 155.2, 150.2, 147.6, 140.6, 136.9, 136.0, 133.5, 129.8, 128.5, 128.1, 128.0, 127.4, 126.1, 124.9, 124.7, 121.3, 79.8, 54.2, 40.3, 28.3. HRMS (ESI+) Calcd. For $C_{24}H_{26}N_2O_2Na$ ([M+Na]$^+$): 397.1886, found: 397.1895. The product was analyzed by HPLC to determine the enantiomeric excess: 97% ee (Chiralpak OD-H, *i*-propanol /hexane = 25/75, flow rate 1.0 mL/min, λ = 254 nm); t_r = 7.38 and 9.62 min.

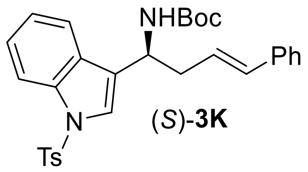
(*S*)-**3K**

***tert*-butyl (*S*,*E*)-(4-phenyl-1-(1-tosyl-1H-indol-3-yl)but-3-en-1-yl)carbamate (Figure 6)** Following **General Reaction Procedure B:** Yield (72%); white solid, m.p. 132-134 °C; $[\alpha]^{30}_D$ = -26.5 (*c* 0.54, CH_2Cl_2); ^{1}H NMR (400 MHz, $CDCl_3$) δ 7.97 (d, *J* = 8.2 Hz, 1H), 7.66 (d, *J* = 8.2 Hz, 2H), 7.59 (d, *J* = 7.6 Hz, 1H), 7.47 (s, 1H), 7.32 – 7.22 (m, 7H), 7.03 (d, *J* = 7.6 Hz, 2H), 6.49 (d, *J* = 16.0 Hz, 1H), 6.13 – 6.05 (m, 1H), 5.12 (m, 1H), 4.80 (brs, 1H), 2.84 (m, 2H), 2.28 (s, 3H), 1.42 (s, 9H). ^{13}C NMR (101 MHz, $CDCl_3$) δ 155.1, 144.9, 137.1, 135.4, 134.9, 133.2, 129.8, 129.3, 128.5, 127.4, 126.7, 126.2, 125.4, 125.0, 123.5, 123.3, 123.2, 120.0, 113.4, 79.8, 46.8, 38.2, 29.7, 21.5. HRMS (ESI+) Calcd. For $C_{30}H_{32}N_2O_4SNa$ ([M+Na]$^+$): 539.1975, found: 539.1977. The product was analyzed by HPLC to determine the enantiomeric excess:

95% ee (Chiralpak OD-H, *i*-propanol /hexane = 25/75, flow rate 1.0 mL/min, λ = 254 nm); t_r = 6.89 and 8.13 min.

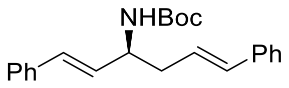

(S)-**3L**

***tert*-butyl ((*S*,1*E*,5*E*)-1,6-diphenylhexa-1,5-dien-3-yl)carbamate (Figure 6)** Following **General Reaction Procedure A:** Yield (88%); white solid, m.p. 102-104 °C; $[\alpha]^{30}_D$ = 1.7 (c 0.24, CH_2Cl_2); ^{1}H NMR (400 MHz, $CDCl_3$) δ 7.40 – 7.27 (m, 8H), 7.25 – 7.19 (m, 2H), 6.56 – 6.46 (m, 2H), 6.23 – 6.15 (m, 2H), 4.66 (brs, 1H), 4.48 (brs, 1H), 2.56 – 2.53 (m, 2H), 1.44 (s, 9H). ^{13}C NMR (101 MHz, $CDCl_3$) δ 155.2, 137.1, 136.7, 133.2, 130.1, 129.8, 128.51, 128.5, 127.5, 127.3, 126.4, 126.1, 125.3, 79.5, 51.9, 39.1, 28.4. HRMS (ESI+) Calcd. For $C_{23}H_{25}NO_2Na$ ($[M+Na]^+$): 372.1934, found: 372.1936. The product was analyzed by HPLC to determine the enantiomeric excess: 95% ee (Chiralpak OD-H, *i*-propanol /hexane = 10/90, flow rate 1.0 mL/min, λ = 254 nm); t_r = 10.21 and 15.55 min.

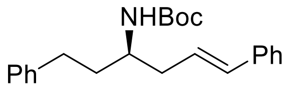

(R)-**3M**

***tert*-butyl (*R*,*E*)-(1,6-diphenylhex-5-en-3-yl)carbamate (Figure 6)** Following **General Reaction Procedure A:** Yield (72%); white solid, m.p. 82-84 °C; $[\alpha]^{30}_D$ = -17.0 (c 0.63, CH_2Cl_2); ^{1}H NMR (400 MHz, $CDCl_3$) δ 7.34 – 7.26 (m, 6H), 7.20 – 7.17 (m, 4H), 6.41 (d, J = 16.0 Hz, 1H), 6.24 – 6.12 (m, 1H), 4.46 – 4.43 (m, 1H), 3.80 (m, 1H), 2.79 – 2.61 (m, 2H), 2.51 – 2.29 (m, 2H), 1.94 – 1.77 (m, 1H), 1.73 – 1.67 (m, 1H), 1.43 (s, 9H). ^{13}C NMR (101 MHz, $CDCl_3$) δ 155.5, 141.8, 137.2, 132.8, 128.4, 128.4, 128.4, 128.3, 127.1, 126.2, 126.0, 125.8, 79.1, 50.2, 45.2, 40.0, 36.7, 28.3. HRMS (ESI+) Calcd. For $C_{23}H_{29}NO_2Na$ ($[M+Na]^+$): 374.2091, found: 374.2091. The product was analyzed by HPLC to determine the enantiomeric excess: 98% ee (Chiralpak OD-H, *i*-propanol /hexane = 10/90, flow rate 1.0 mL/min, λ = 254 nm); t_r = 7.27 and 8.25 min.

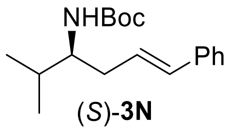

(*S*)-**3N**

***tert*-butyl (*S*,*E*)-(2-methyl-6-phenylhex-5-en-3-yl)carbamate (Figure 6)** Following **General Reaction Procedure A:** Yield (78%); white solid, m.p. 88-90 °C; $[\alpha]^{30}_{D}$ = 5.2 (*c* 0.60, CH_2Cl_2); ^{1}H NMR (400 MHz, Chloroform-*d*) δ 7.37 – 7.32 (m, 2H), 7.31 – 7.27 (m, 1H), 7.23 – 7.17 (m, 1H), 6.42 (d, *J* = 16.0 Hz, 1H), 6.18 – 6.13 (m, 1H), 4.40 – 4.37 (m, 1H), 3.61 – 3.58 (m, 1H), 2.42 – 2.28 (m, 2H), 1.82 – 1.73 (m, 1H), 1.40 (s, 9H), 0.96 (d, *J* = 6.8 Hz, 3H), 0.92 (d, *J* = 6.8 Hz, 3H). ^{13}C NMR (101 MHz, $CDCl_3$) δ 155.8, 137.4, 132.2, 128.4, 127.0, 126.8, 126.0, 79.0, 55.3, 36.2, 31.4, 28.4, 19.4, 17.8. HRMS (ESI+) Calcd. For $C_{18}H_{27}NO_2Na$ ($[M+Na]^+$): 312.1934, found: 312.1939. The product was analyzed by HPLC to determine the enantiomeric excess: 97% ee (Chiralpak OD-H, *i*-propanol /hexane = 10/90, flow rate 1.0 mL/min, λ = 254 nm); t_r = 3.94 and 4.37 min.

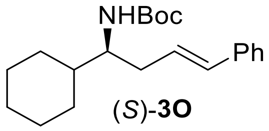

(*S*)-**3O**

***tert*-butyl (*S*,*E*)-(1-cyclohexyl-4-phenylbut-3-en-1-yl)carbamate (Figure 6)** Following **General Reaction Procedure A:** Yield (72%); white solid, m.p. 110-112 °C; $[\alpha]^{30}_{D}$ = 15.4 (*c* 0.74, CH_2Cl_2); ^{1}H NMR (400 MHz, $CDCl_3$) δ 7.37 – 7.17 (m, 5H), 6.41 (d, *J* = 16.0 Hz, 1H), 6.17 (dt, *J* = 16.0, 7.2 Hz, 1H), 4.31 – 4.38 (m, 1H), 3.58 (brs, 1H), 2.43 – 2.40 (m, 1H), 2.32 – 2.24 (m, 1H), 1.78 – 1.65 (m, 5H), 1.39 (s, 9H), 1.29 – 0.94 (m, 6H). ^{13}C NMR (101 MHz, $CDCl_3$) δ 155.8, 146.7, 137.5, 132.2, 128.4, 127.0, 126.0, 78.9, 54.8, 41.4, 36.0, 29.8, 27.4, 26.4, 26.2. HRMS (ESI+) Calcd. For $C_{21}H_{31}NO_2Na$ ($[M+Na]^+$): 352.2247, found: 352.2250. The product was analyzed by HPLC to determine the enantiomeric excess: 97% ee (Chiralpak OD-H, *i*-propanol /hexane = 10/90, flow rate 1.0 mL/min, λ = 254 nm); t_r = 4.11 and 4.87 min.

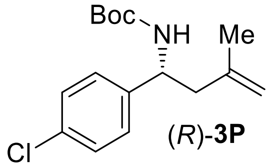
(*R*)-**3P**

***tert*-butyl (*R*)- (1-(4-chlorophenyl)-3-methylbut-3-en-1-yl)carbamate (Figure 7)** Following **General Reaction Procedure E:** Yield (88%); white solid, m.p. 82-84 °C; $[\alpha]^{30}_{D}$ = 11.4 (*c* 0.78, CH_2Cl_2); ^{1}H NMR (400 MHz, $CDCl_3$) δ 7.29 (d, *J* = 8.4 Hz, 2H), 7.21 (d, *J* = 8.4 Hz, 2H), 4.83-4.73 (m, 4H), 2.38-2.34 (m, 2H), 1.72 (s, 3H), 1.39 (s, 9H). ^{13}C NMR (101 MHz, $CDCl_3$) δ 155.2, 141.7, 141.4, 132.6, 128.6, 127.4, 114.1, 79.6, 52.2, 45.7, 28.3, 21.9. HRMS (ESI+) Calcd. For $C_{16}H_{22}ClNO_2Na$ ($[M+Na]^+$): 318.1231, found: 318.1228. The product was analyzed by HPLC to determine the enantiomeric excess: 93% ee (Chiralpak AS-H, *i*-propanol /hexane = 3/97, flow rate 1.0 mL/min, λ = 230 nm); t_r = 5.40 and 6.64 min.

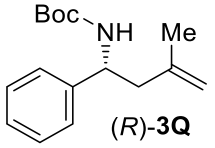
(*R*)-**3Q**

***tert*-butyl (*R*)-(3-methyl-1-(*p*-tolyl)but-3-en-1-yl)carbamate (Figure 7)** Following **General Reaction Procedure E:** Yield (91%); white solid, m.p. 78-80 °C; $[\alpha]^{30}_{D}$ = 10.1 (*c* 0.80, CH_2Cl_2), for deprotected product: $[\alpha]^{30}_{D}$ = 15.2 (*c* 0.68, $CHCl_3$); ^{1}H NMR (400 MHz, $CDCl_3$) δ 7.39 – 7.18 (m, 5H), 4.82-4.73 (m, 4H), 2.42-2.39 (m, 2H), 1.73 (s, 3H), 1.40 (s, 9H). ^{13}C NMR (101 MHz, $CDCl_3$) δ 155.2, 143.0, 141.9, 128.4, 127.0, 126.0, 113.8, 79.4, 52.8, 45.8, 28.3, 21.9. HRMS (ESI+) Calcd. For $C_{16}H_{23}NO_2Na$ ($[M+Na]^+$): 284.1629, found: 284.1621. The product was analyzed by HPLC to determine the enantiomeric excess: 95% ee (Chiralpak AS-H, *i*-propanol /hexane = 3/97, flow rate 1.0 mL/min, λ = 230 nm); t_r = 5.05 and 5.83 min.

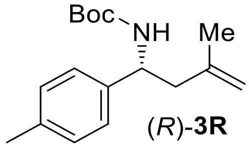
(*R*)-**3R**

***tert*-butyl (*R*)-(3-methyl-1-phenylbut-3-en-1-yl)carbamate (Figure 7)** Following

General Reaction Procedure E: Yield (84%); white solid, m.p. 77-79 °C; $[\alpha]^{30}_D$ = 12.2 (*c* 0.48, CH_2Cl_2); ^{1}H NMR (400 MHz, $CDCl_3$) δ 7.17 (d, *J* = 8.0 Hz, 2H), 7.12 (d, *J* = 8.0 Hz, 2H), 4.81-4.72 (d, *J* = 32.5 Hz, 4H), 2.41 – 2.37 (m, 2H), 2.32 (s, 3H), 1.72 (s, 3H), 1.39 (s, 9H). ^{13}C NMR (101 MHz, $CDCl_3$) δ 155.2, 142.0, 140.1, 136.6, 129.1, 126.0, 113.6, 79.4, 52.4, 45.8, 28.3, 22.0, 21.0. HRMS (ESI+) Calcd. For $C_{17}H_{28}NO_2Na$ ($[M+Na]^+$): 298.1777, found: 298.1778. The product was analyzed by HPLC to determine the enantiomeric excess: 91% ee (Chiralpak AS-H, *i*-propanol /hexane = 3/97, flow rate 1.0 mL/min, λ = 230 nm); t_r = 5.08 and 6.43 min.

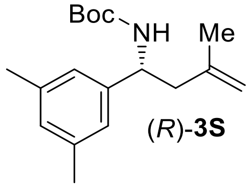

(*R*)-**3S**

***tert*-butyl (*R*)-(1-(3,5-dimethylphenyl)-3-methylbut-3-en-1-yl)carbamate (Figure 7)** Following **General Reaction Procedure E:** Yield (74%); white solid, m.p. 81-83 °C; $[\alpha]^{30}_D$ = 12.8 (*c* 0.65, CH_2Cl_2); ^{1}H NMR (400 MHz, $CDCl_3$) δ 6.88 (s, 3H), 4.82-4.74 (m, 4H), 2.45 – 2.35 (m, 2H), 2.30 (s, 6H), 1.74 (s, 3H), 1.40 (s, 9H). ^{13}C NMR (101 MHz, $CDCl_3$) δ 155.3, 142.1, 137.9, 128.7, 123.8, 113.5, 79.3, 52.7, 46.0, 28.3, 21.9, 21.3. HRMS (ESI+) Calcd. For $C_{18}H_{27}NO_2Na$ ($[M+Na]^+$): 312.1954, found: 312.1946. The product was analyzed by HPLC to determine the enantiomeric excess: 83% ee (Chiralpak AS-H, *i*-propanol /hexane = 3/97, flow rate 1.0 mL/min, λ = 230 nm); t_r = 3.90 and 4.87 min.

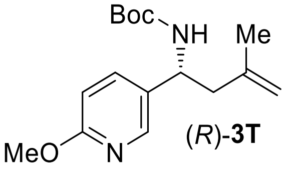

(*R*)-**3T**

***tert*-butyl (*R*)-(1-(6-methoxypyridin-3-yl)-3-methylbut-3-en-1-yl)carbamate (Figure 7)** Following **General Reaction Procedure E:** Yield (92%); white solid, m.p. 90-92 °C; $[\alpha]^{30}_D$ = 15.1 (*c* 0.66, CH_2Cl_2); ^{1}H NMR (400 MHz, $CDCl_3$) δ 8.09 (s, 1H), 7.50 (dd, *J* = 8.4, 2.4 Hz, 1H), 6.71 (d, *J* = 8.4 Hz, 1H), 4.72 – 4.69 (m, 4H), 3.92 (s,

3H), 2.41 (d, J = 7.2 Hz, 2H), 1.73 (s, 3H), 1.39 (s, 9H). ^{13}C NMR (101 MHz, $CDCl_3$) δ 163.4, 155.1, 144.8, 141.3, 136.7, 131.1, 114.2, 110.7, 79.6, 53.4, 50.2, 45.3, 28.3, 21.9. HRMS (ESI+) Calcd. For $C_{16}H_{25}NO_2$ ([M+H]$^+$): 293.1861, found: 293.1860. The product was analyzed by HPLC to determine the enantiomeric excess: 91% ee (Chiralpak AS-H, *i*-propanol /hexane = 3/97, flow rate 1.0 mL/min, λ = 230 nm); t_r = 7.14 and 7.99 min.

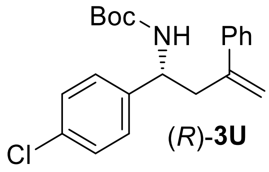

(*R*)-**3U**

***tert*-butyl (*R*)-(1-(4-chlorophenyl)-3-phenylbut-3-en-1-yl)carbamate (Figure 7)** Following **General Reaction Procedure E:** Yield (85%); white solid, m.p. 94-96 °C; $[\alpha]^{30}_D$ = 9.8 (c 0.34, CH_2Cl_2); ^{1}H NMR (400 MHz, $CDCl_3$) δ 7.37 – 7.23 (m, 7H), 7.12 (d, J = 8.4 Hz, 2H), 5.28 (s, 1H), 5.00 (s, 1H), 4.84-4.82 (m, 1H), 4.63 (brs, 1H), 2.88 (d, J = 6.0 Hz, 2H), 1.37 (s, 9H). ^{13}C NMR (101 MHz, $CDCl_3$) δ 155.0, 144.5, 141.3, 140.1, 132.7, 128.5, 128.5, 127.8, 127.6, 126.2, 115.9, 79.6, 53.0, 43.2, 28.2. HRMS (ESI+) Calcd. For $C_{21}H_{24}ClNO_2Na$ ([M+Na]$^+$): 380.1388, found: 380.1389. The product was analyzed by HPLC to determine the enantiomeric excess: 84% ee (Chiralpak AS-H, *i*-propanol /hexane = 3/97, flow rate 1.0 mL/min, λ = 230 nm); t_r = 8.96 and 11.14 min.

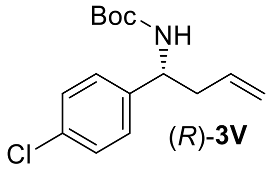

(*R*)-**3V**

***tert*-butyl (*R*)-(1-(4-chlorophenyl)but-3-en-1-yl)carbamate (Figure 7)** Following **General Reaction Procedure E:** Yield (42%); white solid, m.p. 94-96 °C; $[\alpha]^{30}_D$ = 9.8 (c 0.34, CH_2Cl_2); ^{1}H NMR (400 MHz, $CDCl_3$) δ 7.29 (d, J = 8.4 Hz, 2H), 7.20 (d, J = 8.4 Hz, 2H), 5.70 – 5.59 (m, 1H), 5.13 – 5.08 (m, 2H), 4.87 (m, 1H), 4.70 (brs, 1H), 2.47 (m, 2H), 1.41 (s, 9H). ^{13}C NMR (101 MHz, $CDCl_3$) δ 155.1, 141.0, 133.5, 132.7,

128.6, 127.5, 118.6, 79.7, 53.4, 41.1, 28.3. HRMS (ESI+) Calcd. For $C_{15}H_{20}ClNO_2Na$ ($[M+Na]^+$): 304.1081, found: 304.1075. The product was analyzed by HPLC to determine the enantiomeric excess: 80% ee (Chiralpak AD-H, *i*-propanol /hexane = 3/97, flow rate 1.0 mL/min, λ = 230 nm); t_r = 7.64 and 8.81 min.

Synthesis of (*S,E*)-3a and (*S,Z*)-3s using the set of mismatched catalyst combination [Cu(I)/(*R,R$_p$*)-L1 + Ir(I)/(*S,S,S*)-L2]

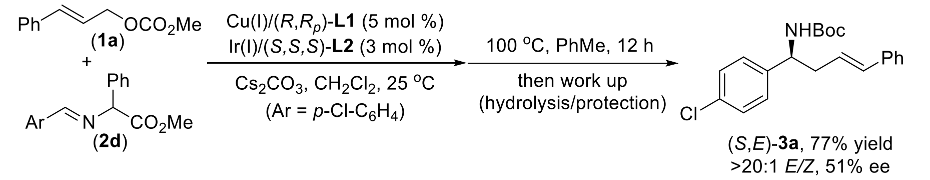

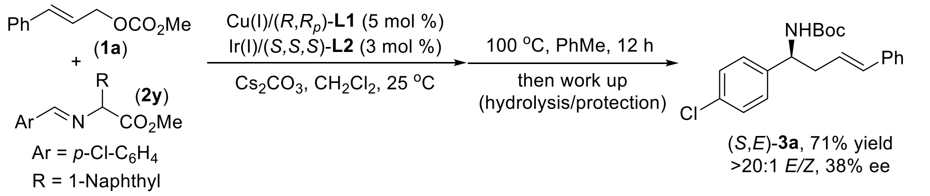

The reaction was conducted following **general reaction procedure C** except with the set of mismatched catalyst combination [Cu(I)/(*R,R$_p$*)-**L1** + Ir(I)/(*S,S,S*)-**L2**]. Using mismatched catalyst combination, compound **3a** was still isolated with exclusively *E*-selectivity. However, the enantioselectivity dropped dramatically in comparison with the result using matched catalyst combination. We proposed the generated allylation intermediate would undergo the 2-aza-Cope rearrangement via the transition states in the fourth quadrant, while the bulkier phenyl group being placed at the axial position would increase the activation barrier of the corresponding transition state and thus reduce the efficacy of chirality transfer.

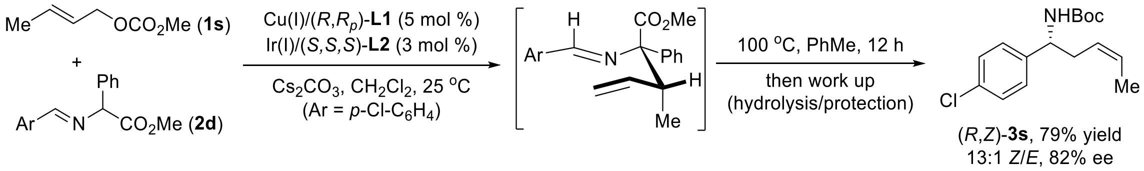

The reaction was conducted following **general reaction procedure C** except with the set of mismatched catalyst combination [Cu(I)/(*R,R$_p$*)-**L1** + Ir(I)/(*S,S,S*)-**L2**]. The

(*Z*)-geometry **3s** was isolated as major product, and the *Z/E* ratio was 13:1 according to the crude ^{1}HNMR. We proposed a possible transition state to rationalize the stereoselectivity. However, this interesting phenomenon for the synthesis of (*Z*)-geometry of homoallylic amines with high *Z/E* ratio are not suitable for allylic carbonates other than crotyl carbonate (when *n*-propyl, cyclohexyl and 2-phenylethyl substituted allylic carbonates was conducted, a mixture of *Z/E* products with 1.5:1 to 3:1 *Z/E* ratio were obtained).

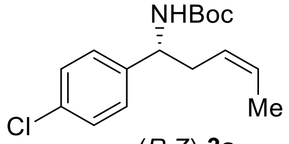

(*R,Z*)-**3s**

***tert*-butyl *(R,Z)*-(1-(4-chlorophenyl)pent-3-en-1-yl)carbamate:** Yield (79%); white solid, m.p. 103-105 °C; $[\alpha]^{30}_{D}$ = 15.6 (*c* 0.34, CH_2Cl_2); ^{1}H NMR (400 MHz, $CDCl_3$) δ 7.29 (d, *J* = 8.4 Hz, 2H), 7.20 (d, *J* = 8.4 Hz, 2H), 5.62 – 5.54 (m, 1H), 5.30 – 5.23 (m, 1H), 4.86 (m, 1H), 4.67 (brs, 1H), 2.48 – 2.47 (m, 2H), 1.57 – 1.55 (m, 3H), 1.41 (s, 9H). ^{13}C NMR (101 MHz, $CDCl_3$) δ 155.1, 141.2, 132.7, 128.5, 127.6, 125.8, 124.9, 79.6, 53.9, 34.0, 28.3, 12.9. HRMS (ESI+) Calcd. For $C_{16}H_{22}ClNO_2Na$ ($[M+Na]^+$): 318.1231, found: 318.1227. The product was analyzed by HPLC to determine the enantiomeric excess: 82% ee (Chiralpak AS-H, *i*-propanol /hexane = 3/97, flow rate 1.0 mL/min, λ = 230 nm); t_r = 5.59 and 6.90 min.

Synthesis of (*S*)-5 and 6

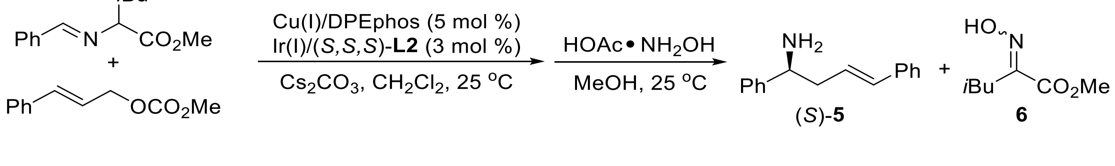

A flame dried Schlenk tube I was cooled to rt and filled with N_2. To this flask were added $[Ir(COD)Cl]_2$ (0.15 mmol, 1.5 mol %), phosphoramidite ligand (*S,S,S*)-**L2** (0.3 mmol, 3 mol %), degassed THF (10 mL) and degassed *n*-propylamine (10 mL). The reaction mixture was heated at 50 °C for 30 min and then the volatile solvents were

removed under vacuum to give a pale yellow solid. Meanwhile, in a separated Schlenk tube II, DPEPhos (0.55 mmol, 5.5 mol %) and $Cu(MeCN)_4BF_4$ (0.5 mmol, 5 mol %) were dissolved in 5 mL of DCM, and stirred at room temperature for about 0.5 h. The Cu/DPEphos complex solution was then transferred to the Schlenk tube I containing iridium complex via syringe. Allylic carbonates (10 mmol), aldimine esters (15 mmol), Cs_2CO_3 (20 mmol), DCM (5 mL) were then added. The reaction was stirred at room temperature for 18 h, a solution of $HONH_2$•AcOH in methanol [1M, 20 mL, prepared from $HONH_2$•HCl, NaOH (solid, 1 equiv), and AcOH (1 equiv) in methanol] was added to the reaction mixture. After being stirred at the same temperature for 3 h, the organic solvent was removed by rotary evaporation and the residue was purified by column chromatography to give the product (*S*)-**5a** (1.96 g, 88%, Rf = 0.2, petroleum/ethyl acetate = 2/1) and **6** (1.43 g, 90%, Rf = 0.6, petroleum/ethyl acetate = 4/1).

Synthesis of 7

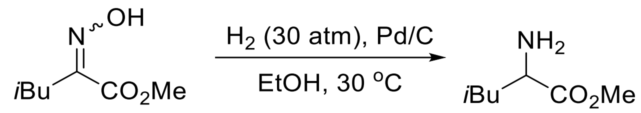

A suspension of Pd/C (0.15 g) and **6** (1.43 g) in 10 mL EtOH and H_2 was stirred at 30 °C under 30 atm hydrogen atmosphere. After being stirred for 6 h, the mix was filtered through a pad of Celite to remove the Pd/C powder. The organic solvent was then removed by rotary evaporation to give the desired product DL-methyl leucine **7** in >98% purity (1.43 g, 99%).

Synthesis of (2*R*,3*S*,5*S*)-12

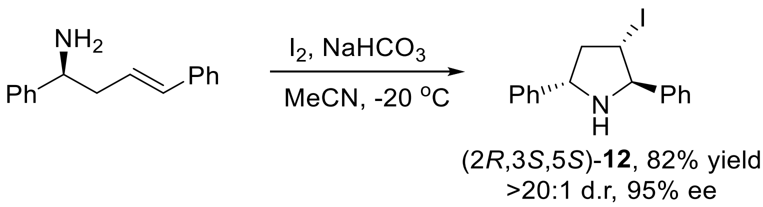

I_2 (1.0 mmol) was added to a MeCN (3 mL) solution of (*S*)-**5** (0.5 mmol) and $NaHCO_3$ (1 mmol) at -20 °C. The mixture was stirred for 12 h before quenched by

addition of CH_2Cl_2 (5 mL) and saturated $Na_2S_2O_3$. The mixture was separated, and the aqueous layer was extracted with additional portions of CH_2Cl_2. The combined organic layers were combined, concentrated, and purified using flash chromatography (hexanes/EtOAc = 20:1) to give the desired product as a beige solid (143 mg, 82%).

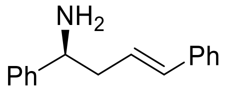

(*S*)-**5**

***(S,E)*-1,4-diphenylbut-3-en-1-amine:** Yield (88%); white solid, m.p. 94-96 °C; $[\alpha]^{30}_{D}$ = -25.2 (*c* 0.98, CH_2Cl_2); ^{1}H NMR (400 MHz, $CDCl_3$) δ 7.39 – 7.15 (m, 10H), 6.48 (d, *J* = 16.0 Hz, 1H), 6.24 – 6.08 (m, 1H), 4.07 (dd, *J* = 8.0, 5.2 Hz, 1H), 2.61 – 2.44 (m, 2H), 1.65 (brs, 2H). ^{13}C NMR (101 MHz, $CDCl_3$) δ 145.7, 137.3, 132.8, 128.5, 128.4, 127.2, 127.0, 126.9, 126.3, 126.0, 55.7, 43.4. HRMS (ESI+) Calcd. For $C_{16}H_{17}NNa$ ($[M+Na]^+$): 246.1253, found: 246.1254. The product was analyzed by HPLC to determine the enantiomeric excess: 95% ee (Chiralpak OD-H, *i*-propanol /hexane = 40/60, flow rate 1.0 mL/min, λ = 254 nm); t_r = 7.05 and 9.39 min.

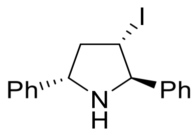

(2*R*,3*S*,5*S*)-**12**

(2*R*,3*S*,5*S*)-3-iodo-2,5-diphenylpyrrolidine: Yield (82%); colorless oil; $[\alpha]^{30}_{D}$ = -65.2 (*c* 0.48, CH_2Cl_2); ^{1}H NMR (400 MHz, $CDCl_3$) δ 7.54 – 7.42 (m, 2H), 7.42 – 7.21 (m, 8H), 4.59 – 4.55 (mz, 2H), 4.14 – 4.07 (m, 1H), 3.04 – 2.98 (m, 1H), 2.38 – 2.30 (m, 1H), 2.08 (brs, 1H). ^{13}C NMR (101 MHz, $CDCl_3$) δ 142.9, 140.4, 132.7, 128.8, 128.6, 128.2, 127.5, 126.8, 72.8, 61.1, 48.4, 28.3. HRMS (ESI+) Calcd. For $C_{16}H_{16}INNa$ ($[M+Na]^+$): 372.0220, found: 372.0227. The product was analyzed by HPLC to determine the enantiomeric excess: 95% ee (Chiralpak IA-H, *i*-propanol /hexane = 3/97, flow rate 1.0 mL/min, λ = 254 nm); t_r = 12.14 and 17.17 min.

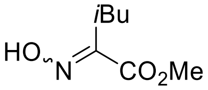

7

methyl 2-(hydroxyimino)-4-methylpentanoate: Yield (90%); light yellow solid, m.p. 98-100 °C; ^{1}H NMR (400 MHz, $CDCl_3$) δ 3.85 (s, 3H), 2.54 (d, *J* = 7.2 Hz, 2H), 2.11 – 2.00 (m, 1H), 0.94 (d, *J* = 6.8 Hz, 6H). ^{13}C NMR (101 MHz, $CDCl_3$) δ 164.3, 152.5, 52.6, 33.2, 26.5, 22.6. HRMS (ESI+) Calcd. For $C_7H_{13}INO_3Na$ ($[M+Na]^+$): 182.0788, found: 182.0795.

Synthesis of (*S*)-10

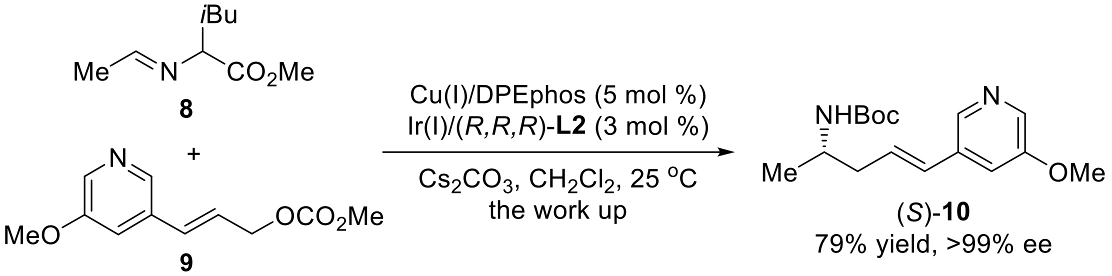

A flame dried Schlenk tube I was cooled to rt and filled with N_2. To this flask were added $[Ir(COD)Cl]_2$ (0.03 mmol, 1.5 mol %), phosphoramidite ligand (*R,R,R*)-**L2** (0.06 mmol, 3 mol %), degassed THF (3 mL) and degassed *n*-propylamine (3 mL). The reaction mixture was heated at 50 °C for 30 min and then the volatile solvents were removed under vacuum to give a pale yellow solid. Meanwhile, in a separated Schlenk tube II, DPEPhos (0.11 mmol, 5.5 mol %) and $Cu(MeCN)_4BF_4$ (0.1 mmol, 5 mol %) were dissolved in 3 mL of DCM, and stirred at room temperature for about 0.5 h. The Cu/DPEphos complex solution was then transferred to the Schlenk tube I containing iridium complex via syringe. Allylic carbonates **9** (2.0 mmol), aldimine esters **8** (3.0 mmol), Cs_2CO_3 (4.0 mmol), DCM (3 mL) were then added. The reaction was stirred at room temperature for 18 h and 2 N HCl (8 mL) was added to the mixture. After stirring at rt for 0.5 h, 2 N NaOH (15 mL) and Boc_2O (880 mg, 4 mmol) was then added and stirring at rt for 3 h. The layers were separated, and the aqueous layer was extracted with DCM (20 mL x 2). The combined organic components were washed with saturated brine (20 mL), dried over anhydrous Na_2SO_4, filtration and evaporated in vacuum. The

crude product was purified by column chromatography to give the desired product (*S*)-**10** (0.46 g, 79%).

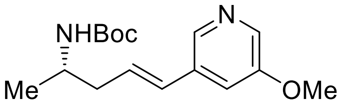

(*S*)-**10**

***tert*-butyl *(S,E)*-(5-(5-methoxypyridin-3-yl)pent-4-en-2-yl)carbamate:** Yield (79%); colorless oil; $[\alpha]^{30}_{D}$ = 5.2 (*c* 0.60, CH_2Cl_2); ^{1}H NMR (400 MHz, $CDCl_3$) δ 8.16 (s, 2H), 7.17 (s, 1H), 6.40 (d, *J* = 16.0 Hz, 1H), 6.30 – 6.21 (m, 1H), 4.47 (brs, 1H), 3.87 – 3.78 (m, 4H), 2.40 – 2.36 (m, 2H), 1.42 (s, 9H), 1.18 (d, *J* = 6.7 Hz, 3H). ^{13}C NMR (101 MHz, $CDCl_3$) δ 155.6, 155.2, 140.6, 136.2, 133.5, 129.2, 128.8, 116.6, 79.2, 55.5, 46.1, 40.8, 28.3, 20.7. HRMS (ESI+) Calcd. For $C_{16}H_{24}N_2O_3Na$ ($[M+Na]^+$): 315.1679, found: 315.1675. The product was analyzed by HPLC to determine the enantiomeric excess: >99% ee (Chiralpak OD-H, *i*-propanol /hexane = 25/75, flow rate 1.0 mL/min, λ = 254 nm); t_r = 4.31 and 4.71 min.

Synthesis of (*S*)-13 and (*S*)-14 catalyzed by (2*R*,3*S*,5*S*)-12

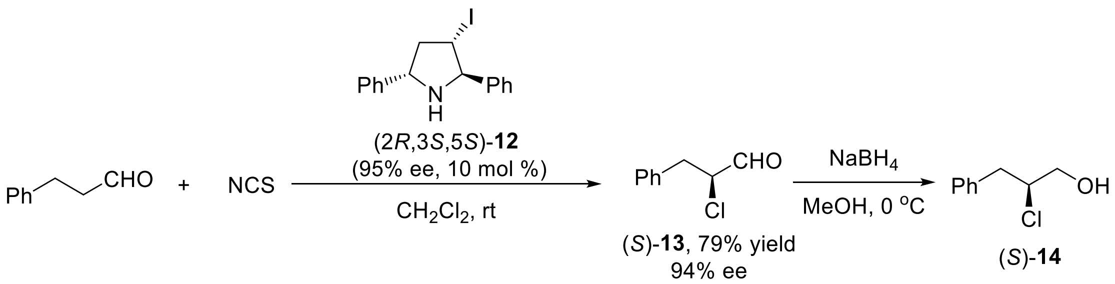

The catalyst (2*R*,3*S*,5*S*)-**12** (0.05mmol, 10 mol%, 95% ee) was added to a stirred ice-cooled (0 °C) solution of the aldehyde (0.5 mmol) in CH_2Cl_2 (1.0 mL) followed by the addition of NCS (87 mg, 0.65 mmol, 1.3 eq.). The reaction mixture was stirred until the aldehyde was completely consumed as determined by 1H NMR spectroscopy of the reaction mixture. Pentane was added to the reaction mixture and the precipitated NCS and succinimide were filtered off. After removal of the solvents, the crude product was dissolved in MeOH (1 mL), $NaBH_4$ (50 mg) was added and the mixture was stirred at 0 °C for 1 h to reduce the α-chlorinated aldehyde to the corresponding alcohol.

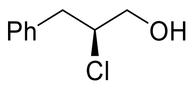

(*S*)-**14**

(*S*)-2-chloro-3-phenylpropan-1-ol: Yield (79%); colorless oil; ^{1}H NMR (400 MHz, $CDCl_3$) δ 7.33 – 7.22 (m, 5H), 4.26 – 4.20 (m, 1H), 3.85 – 3.79 (m, 1H), 3.72 – 3.65 (m, 1H), 3.14 (dd, *J* = 14.0, 7.2 Hz, 1H), 3.05 (dd, *J* = 14.0, 7.2 Hz, 1H), 2.08 – 2.04 (m, 1H). ^{13}C NMR (101 MHz, $CDCl_3$) δ 137.0, 129.3, 128.6, 127.0, 65.8, 64.9, 40.6. HRMS (ESI+) Calcd. For $C_9H_{11}ClONa$ ($[M+Na]^+$): 193.0391, found: 193.0394. The product was analyzed by HPLC to determine the enantiomeric excess: 94% ee (Chiralpak OD-H, *i*-propanol /hexane = 3/97, flow rate 1.0 mL/min, λ = 210 nm); t_r = 11.1 and 16.0 min.

Mechanistic Study

General procedure and characterization data of for rearranged imine:

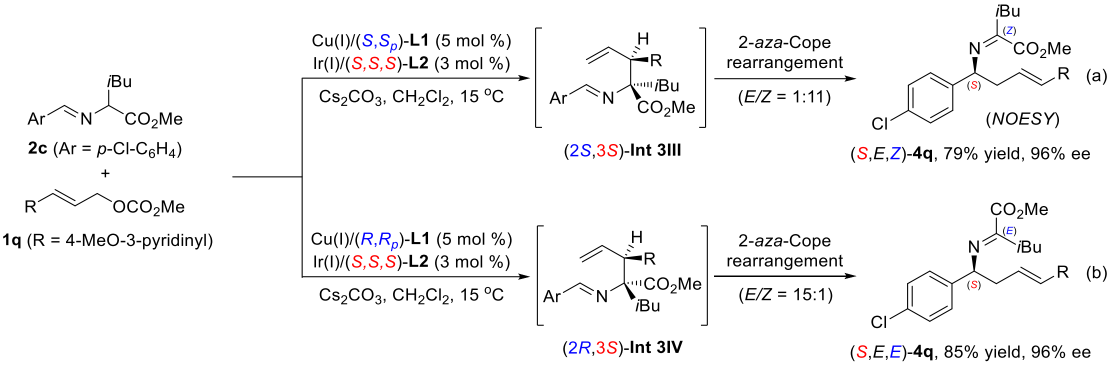

A flame dried Schlenk tube was cooled to rt and filled with N_2. To this flask were added $[Ir(COD)Cl]_2$ (0.003 mmol, 1.5 mol %), phosphoramidite ligand (*S,S,S*)-**L2** (0.006 mmol, 3 mol %), degassed THF (0.5 mL) and degassed *n*-propylamine (0.5 mL). The reaction mixture was heated at 50 °C for 30 min and then the volatile solvents were removed under vacuum to give a pale yellow solid. Meanwhile, in a separated Schlenk tube, **L1** [(*S,S$_p$*) or (*R,R$_p$*), 0.011 mmol, 5.5 mol %] and $Cu(MeCN)_4BF_4$ (0.01 mmol, 5 mol %) were dissolved in 0.5 mL of DCM, and stirred at room temperature for about 0.5 h. The Cu/**L1** complex solution was then transferred to the Schlenk tube containing iridium complex via syringe. Allylic carbonate **1q** (0.20 mmol), leucine derived aldimine ester **2c** (0.30 mmol), Cs_2CO_3 (0.30 mmol) and DCM (0.5 mL) were then added and the reaction was cooled to 15 °C. The cascade reaction was finished smoothly in 12 h (monitored by ^{1}H NMR spectroscopy). The organic solvent was removed by rotary evaporation and the residue was purified by column chromatography to give the product (petroleum/ethyl acetate/Et_3N = 100/50/5).

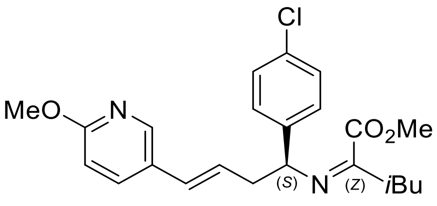

(*S,E,Z*)-**4q**

methyl (*Z*)-2-(((*S*,*E*)-1-(4-chlorophenyl)-4-(6-methoxypyridin-3-yl)but-3-en-1-yl)imino)-4-methylpentanoate: Yield (79%); colorless oil; ^{1}H NMR (400 MHz, $CDCl_3$) δ 8.02 (d, *J* = 2.4 Hz, 1H), 7.57 (dd, *J* = 8.8, 2.4 Hz, 1H), 7.30 – 7.26 (m, 4H), 6.68 (d, *J* = 8.8 Hz, 1H), 6.29 (d, *J* = 6.0 Hz, 1H), 5.95 – 5.87 (m, 1H), 4.49 (dd, *J* = 7.6, 6.0 Hz, 1H), 3.92 (s, 3H), 3.76 (s, 3H), 2.72 – 2.65 (m, 2H), 2.37 (dd, *J* = 7.2, 1.2 Hz, 2H), 2.00 – 1.93 (mz, 1H), 0.91 (dd, *J* = 6.5, 5.2 Hz, 6H). ^{13}C NMR (101 MHz, $CDCl_3$) δ 165.5, 163.3, 161.8, 144.9, 141.3, 135.4, 132.8, 128.5, 128.3, 126.6, 125.8, 110.8, 67.4, 53.4, 51.7, 46.1, 42.1, 25.6, 22.32, 22.27. HRMS (ESI+) Calcd. For $C_{23}H_{28}N_2O_3$ ([M+H]$^+$): 415.1783, found: 415.1788. The enantiomeric excess was determined after work up following general workup procedure.

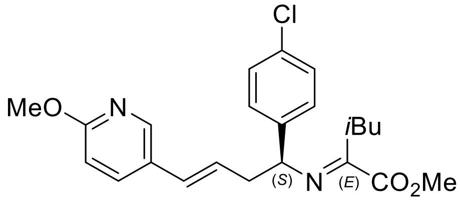

(*S*,*E*,*E*)-**4q**

methyl (*E*)-2-(((*S*,*E*)-1-(4-chlorophenyl)-4-(6-methoxypyridin-3-yl)but-3-en-1-yl)imino)-4-methylpentanoate: Yield (85%); colorless oil; ^{1}H NMR (400 MHz, CD_2Cl_2) δ 7.91 (d, *J* = 2.4 Hz, 1H), 7.46 (dd, *J* = 8.8, 2.4 Hz, 1H), 7.28 – 7.21 (m, 4H), 6.57 (d, *J* = 8.8 Hz, 1H), 6.22 (d, *J* = 16.0 Hz, 1H), 5.87 – 5.79 (m, 1H), 4.65 (t, *J* = 6.8 Hz, 1H), 3.79 (s, 3H), 3.71 (s, 3H), 2.67 – 2.56 (m, 2H), 2.42 – 2.31 (m, 2H), 1.79 – 1.73 (m, 1H), 0.80 (d, *J* = 6.8 Hz, 3H), 0.69 (d, *J* = 6.8 Hz, 3H). ^{13}C NMR (101 MHz, CD_2Cl_2) δ 166.3, 163.8, 163.3, 145.4, 141.8, 135.6, 133.1, 129.3, 129.0, 129.0, 126.8, 125.6, 111.0, 65.4, 53.6, 52.8, 42.80, 37.6, 27.4, 22.9, 22.6. HRMS (ESI+) Calcd. For $C_{23}H_{28}N_2O_3$ ([M+H]$^+$): 415.1783, found: 415.1790. The enantiomeric excess was determined after work up following general workup procedure.

Investigation of the acceleration effect of copper and iridium catalysts on 2-aza-

Cope rearrangement:

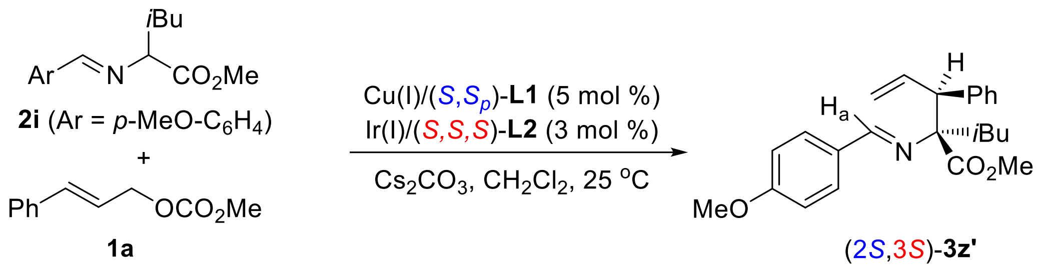

The reaction was performed following **general reaction procedure A** without further work up. (2*S*,3*S*)-**3z'** was isolated as single isomer, which was diluted in CD_2Cl_2, and taken a ^{1}H NMR spectrum using CH_2Br_2 as internal standard.

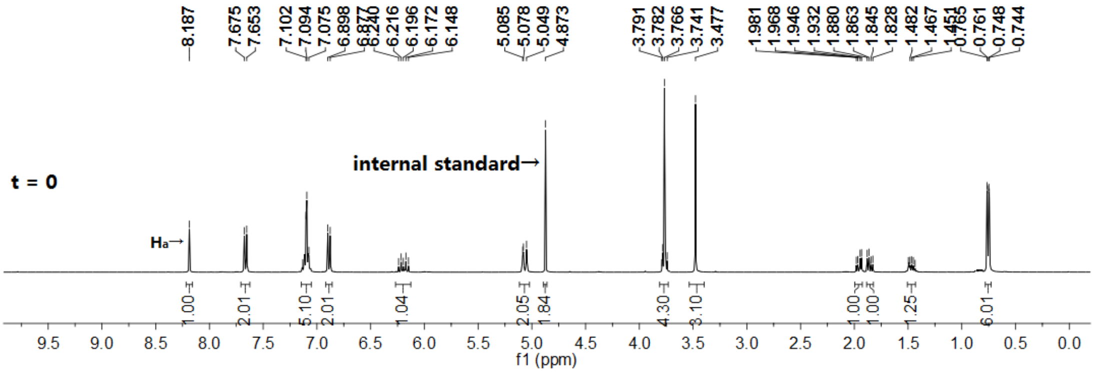

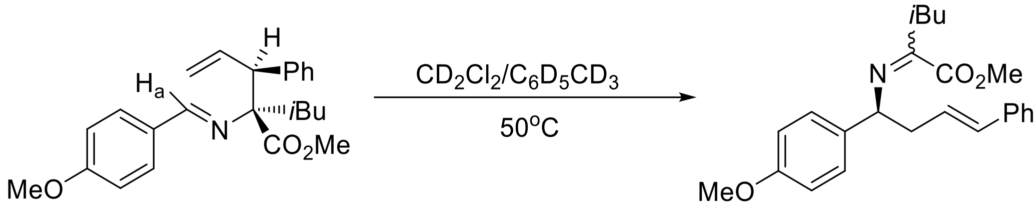

(2*S*,3*S*)-**3z'** (0.2 mmol) and CH_2Br_2 (0.2 mmol) were dissolved in a mixed solution of CD_2Cl_2 (250 μL) and *d*8-toluene (250 μL) and transferred to a dry NMR tube. The NMR tube was sealed up, heated to 50 °C for the specified time and submitted to the NMR analysis.

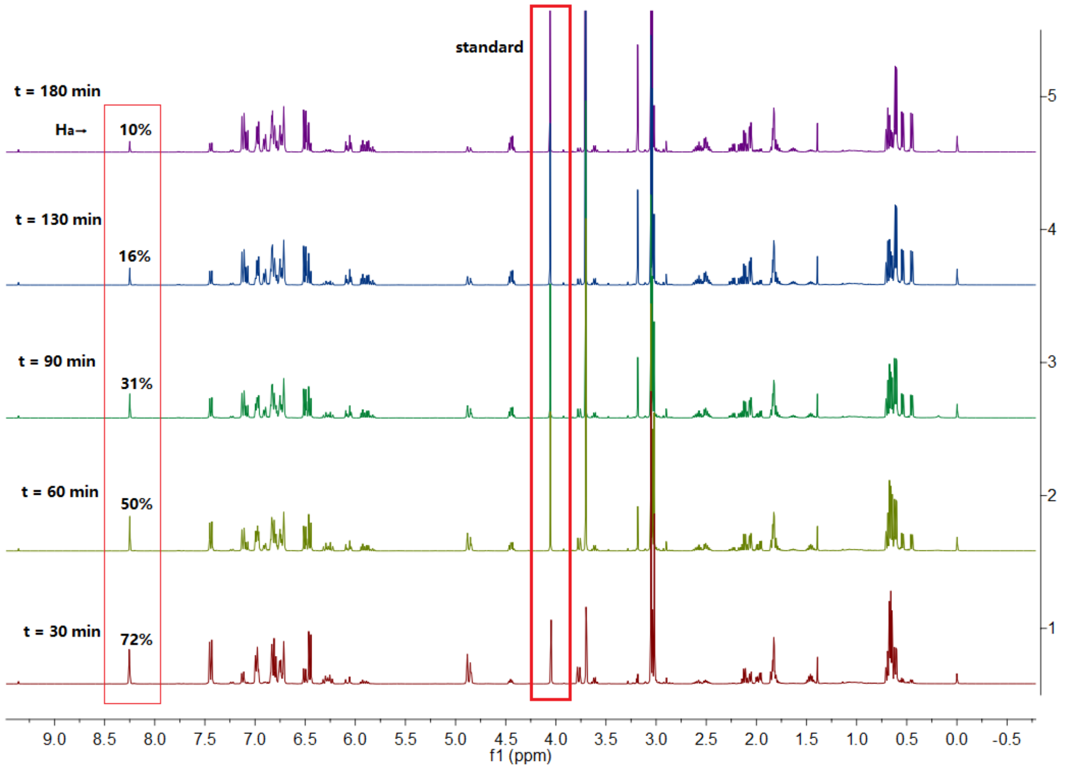

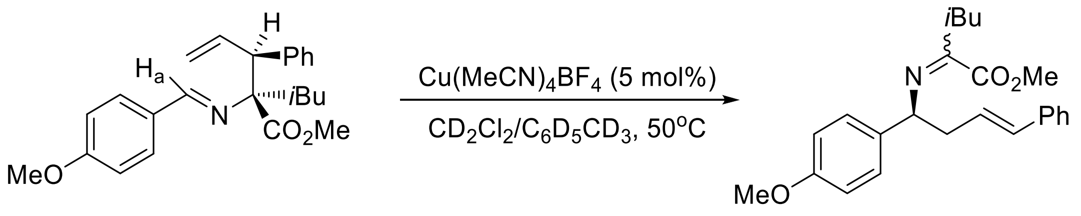

(2*S*,3*S*)-**3z'** (0.2 mmol), CH_2Br_2 (0.2 mmol) and $Cu(MeCN)_4BF_4$ (0.01 mmol) were dissolved in a mixed solution of CD_2Cl_2 (250 μL) and *d*8-toluene (250 μL) and transferred to a dry NMR tube. The NMR tube was sealed up, heated to 50 ºC for the specified time and submitted to the NMR analysis.

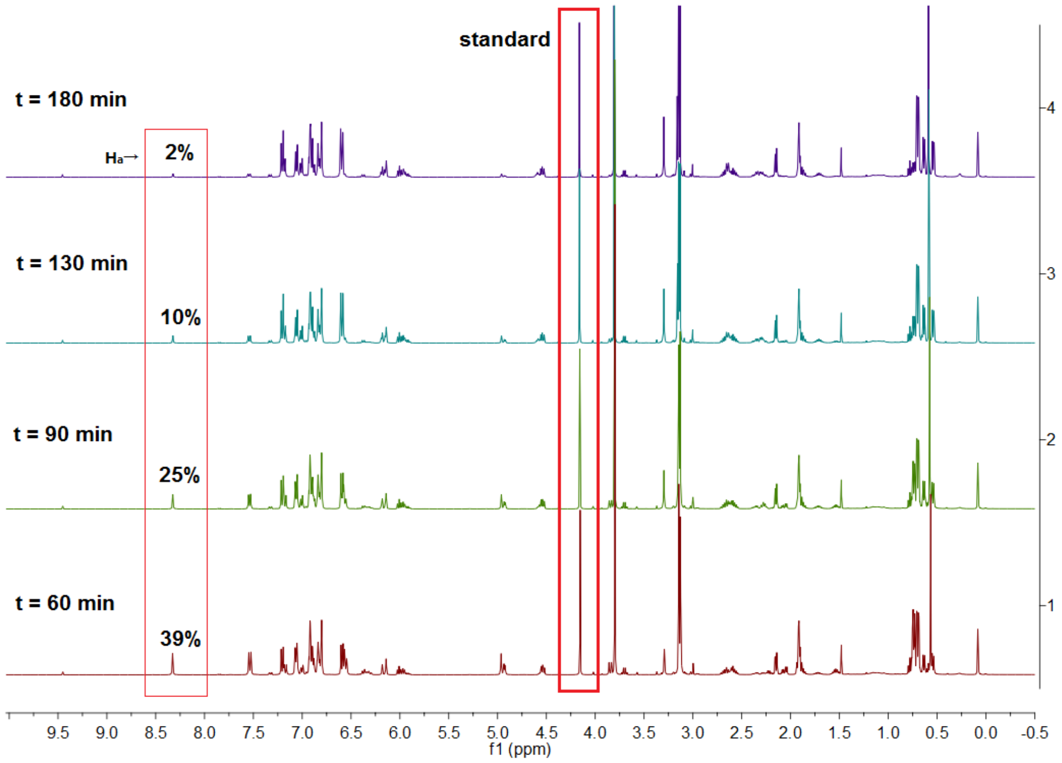

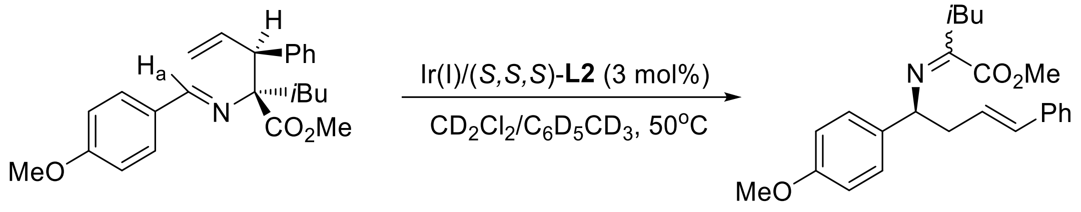

(2*S*,3*S*)-**3z'** (0.2 mmol), CH_2Br_2 (0.2 mmol) and Ir(I)/(*S*,*S*,*S*)-**L2** complex (0.006 mmol) were dissolved in a mixed solution of CD_2Cl_2 (250 µL) and *d*8-toluene (250 µL) and transferred to a dry NMR tube. The NMR tube was sealed up, heated to 50 °C for the specified time and submitted to the NMR analysis.

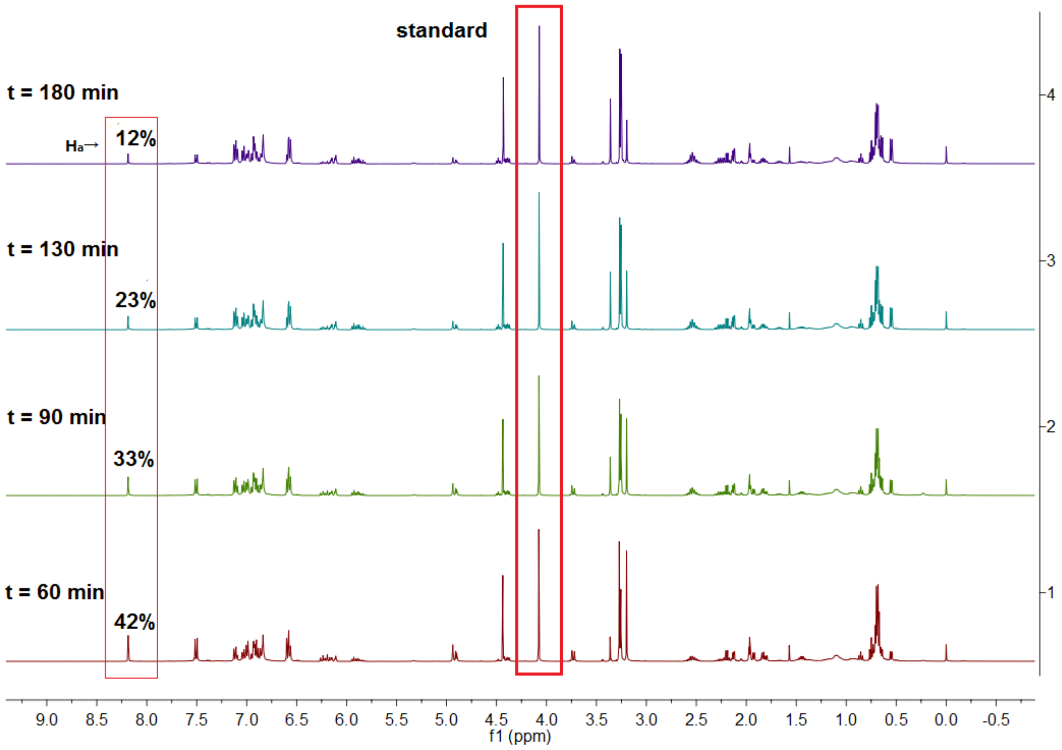

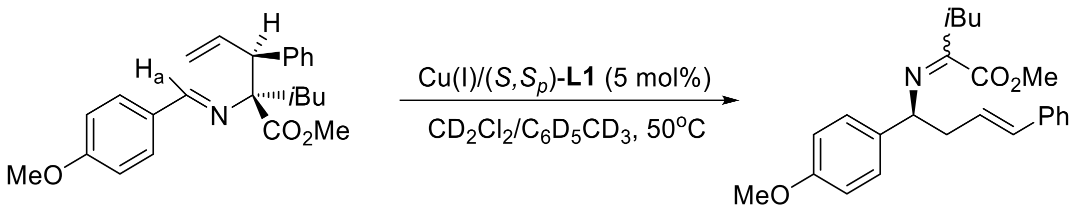

(2*S*,3*S*)-**3z'** (0.2 mmol), CH_2Br_2 (0.2 mmol) and Cu(I)/(*S*,S_p)-**L1** complex (0.01 mmol) were dissolved in a mixed solution of CD_2Cl_2 (250 µL) and *d*8-toluene (250 µL) and transferred to a dry NMR tube. The NMR tube was sealed up, heated to 50 °C for the specified time and submitted to the NMR analysis.

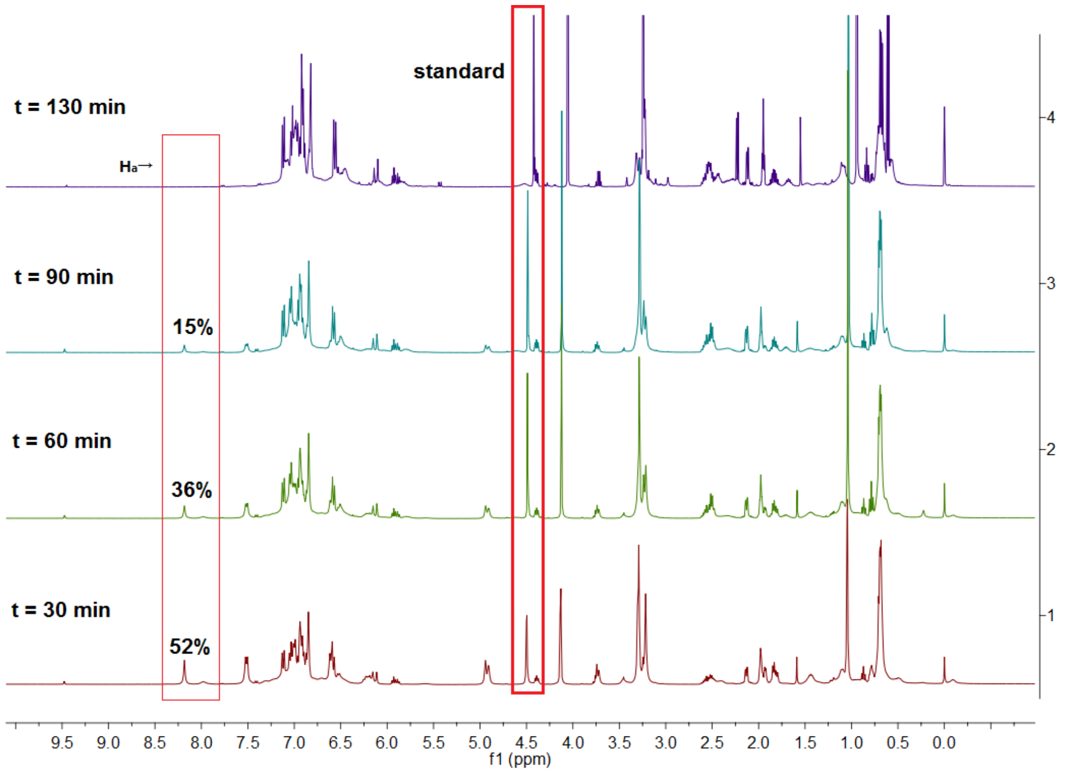

Investigation on steric effect of 2-aza-Cope rearrangement

The screening of aldimine esters with different substituents strongly indicated that the steric effect is the key fact for 2-aza-Cope rearrangement (Table S1). For instance, aldimine esters with less bulky α-substituents such as benzyl and methyl group provide the allylated intermediates without sufficient steric congestion, and therefore subsequent 2-aza-Cope rearrangement proceeded slowly. On the other hand, for glycine derived aldimine ester **2C**, the corresponding allylated intermediate was unstable at high temperature, and most of the intermediate decomposed in toluene at 110 ºC.

Supplementary Table 1. Initial Test to Find an Aldimine Ester Bearing an Appropriate a-Substituent to Facilitate Both Allylation and 2-aza-Cope Rearrangement.

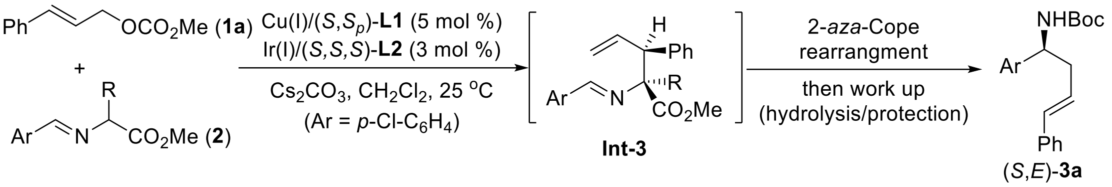

Entry[a]	R	allylation	*T* for 2-*aza*-Cope/°C	2-*aza*-Cope	yield (%)[b]	ee (%)[c]
1	*tert*-Butyl (**2a**)	×	25	-	-	-
2	*iso*-Propyl (**2b**)	×	25	-	-	-
3	*iso*-Butyl (**2c**)	√	25	√	86	96
4	Phenyl (**2d**)	√	25	√	83	95
5	Benzyl (**2A**)	√	110	√	12	-
6	Methyl (**2B**)	√	110-150	×	-	-
7	H (**2C**)	√	110	decomposed	-	-

[a]All reactions were carried out with 0.20 mmol of **1a** and 0.30 mmol of **2** in 1 mL of CH_2Cl_2.
[b]Isolated yield.
[c]Ee was determined by HPLC analysis.

^{31}P NMR analysis of the mixture of Cu/Ir complexes and control experiment

Preparation of Cu(I)-**L3** complex in CD_2Cl_2

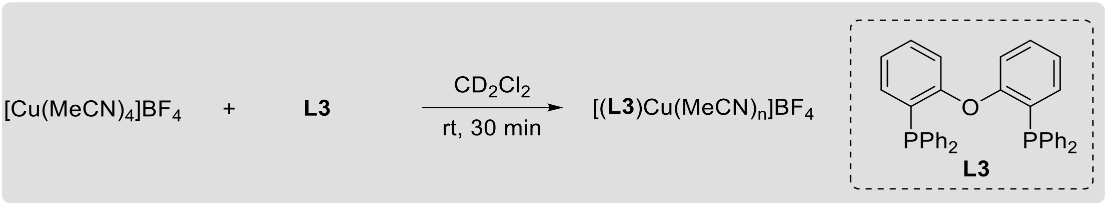

A 5 mL glass vial was charged with $[Cu(MeCN)_4]BF_4$ (12.6 mg, 40 μmol) and **L3** (21.6 mg, 40 μmol) under argon atmosphere. To this mixture was added 500 μL CD_2Cl_2, and the resulting mixture was stirred for 30 min at room temperature to afford clear yellow solution. The solution was transferred to a dried NMR tube, and was submitted to NMR analysis.

Preparation of Ir(III)-**L2** complex

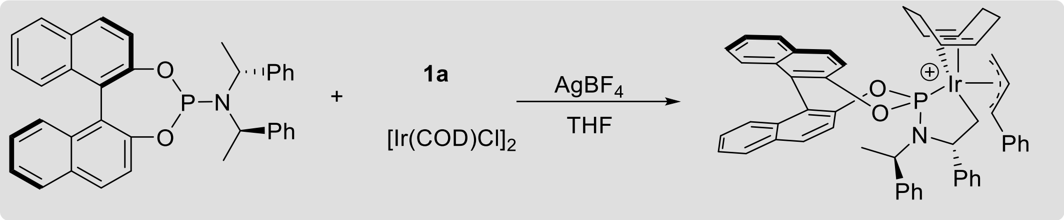

The iridium complex was prepared according to Hartwig's method.[7] Dissolved the complex (10 μmol) with 500 μL CD_2Cl_2 and transferred to a dried NMR tube for NMR analysis.

Preparation of a mixed complex solution

To a dried NMR tube were successively added the solution of iridium complex (10 μmol, prepared as mentioned before) in 300 μL CD_2Cl_2 and the solution of Cu(I)-**L3** complex (40.0 μmol, prepared as mentioned before) in 300 μL CD_2Cl_2 at room temperature under argon atmosphere, and the resulting mixture was shaken up for 30 sec. The ^{31}P NMR spectra were recorded at 25 °C at 3 h after the two solutions were mixed. No new peak was observed according to the ^{31}P NMR spectra, indicated that the ligand scrambling was negligible or absent in this reaction (Figure S1).

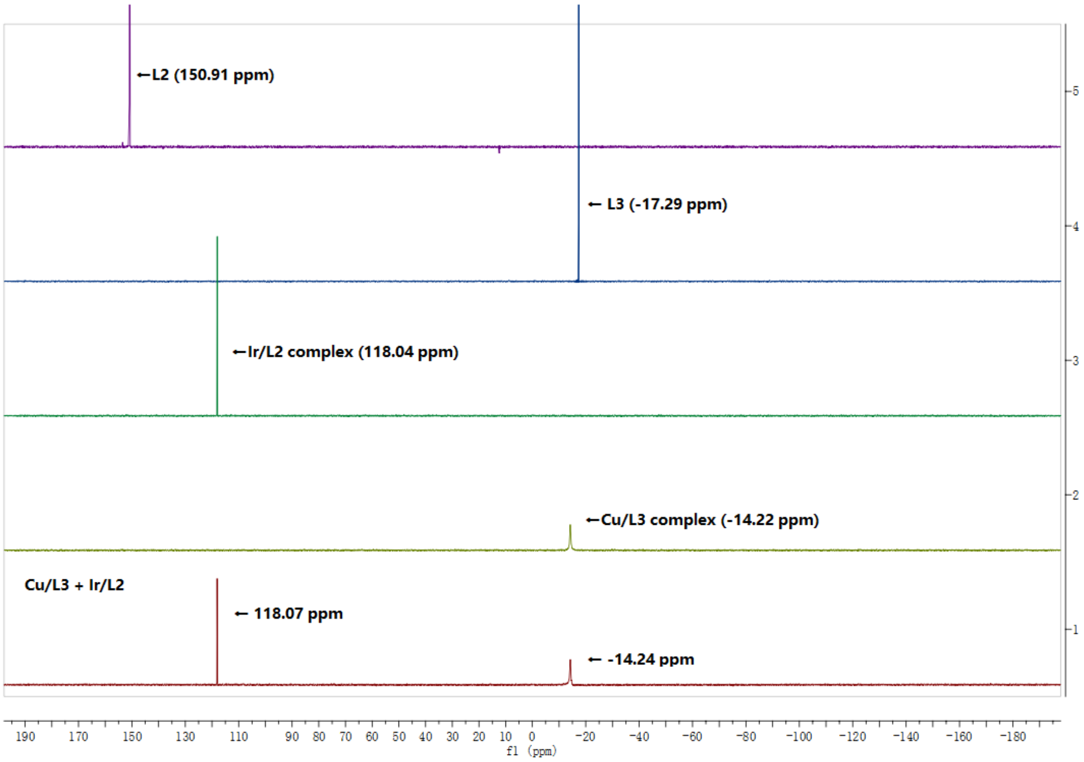

The reaction catalyzed by Cu(I)/**L3**+Ir(I)/**L3**

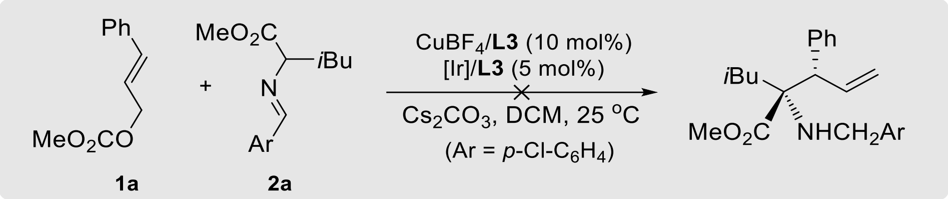

A flame dried Schlenk tube was cooled to rt and filled with N_2. To this flask were added $[Ir(COD)Cl]_2$ (3.4 mg, 5 µmol), (*S,Sp*)-**L3** (16.2 mg, 30 µmol), $Cu(MeCN)_4BF_4$ (6.3 mg, 20 µmol)) and DCM (0.5 mL). The mixture was stirred at room temperature for 30 min. Allylic carbonates (0.2 mmol), aldimine esters (0.3 mmol), Cs_2CO_3 (0.3 mol) and DCM (0.5 mL) were then added. After stirring at room temperature for 12 h, no allylated product was observed by TLC monitoring, both starting materials are remained. The results clearly suggested that the Ir(I)-**L3** complex are incapable of promoting the formation of (π-allyl)-Ir intermediate at the reaction condition.

The reaction catalyzed by Cu(I)/**L2**+Ir(I)/**L2**

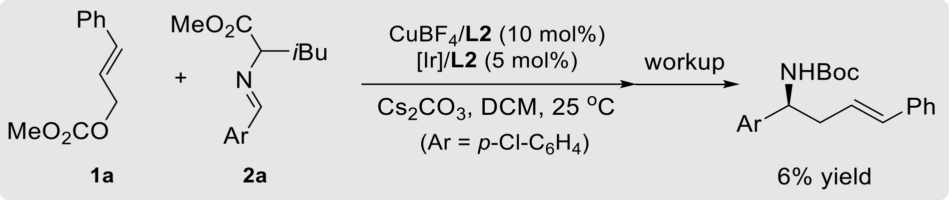

The reaction was performed as the general procedure A described except to use **L2** instead of **L3** as the chiral ligand for copper(I) complex. After reacted at room temperature for 18 h, only trace amount of desired product formed. The result indicated that the active catalyst in our methodology to activated aldimine ester is unlikely to be Cu(I)/**L2** complex.

Supplementary Figures

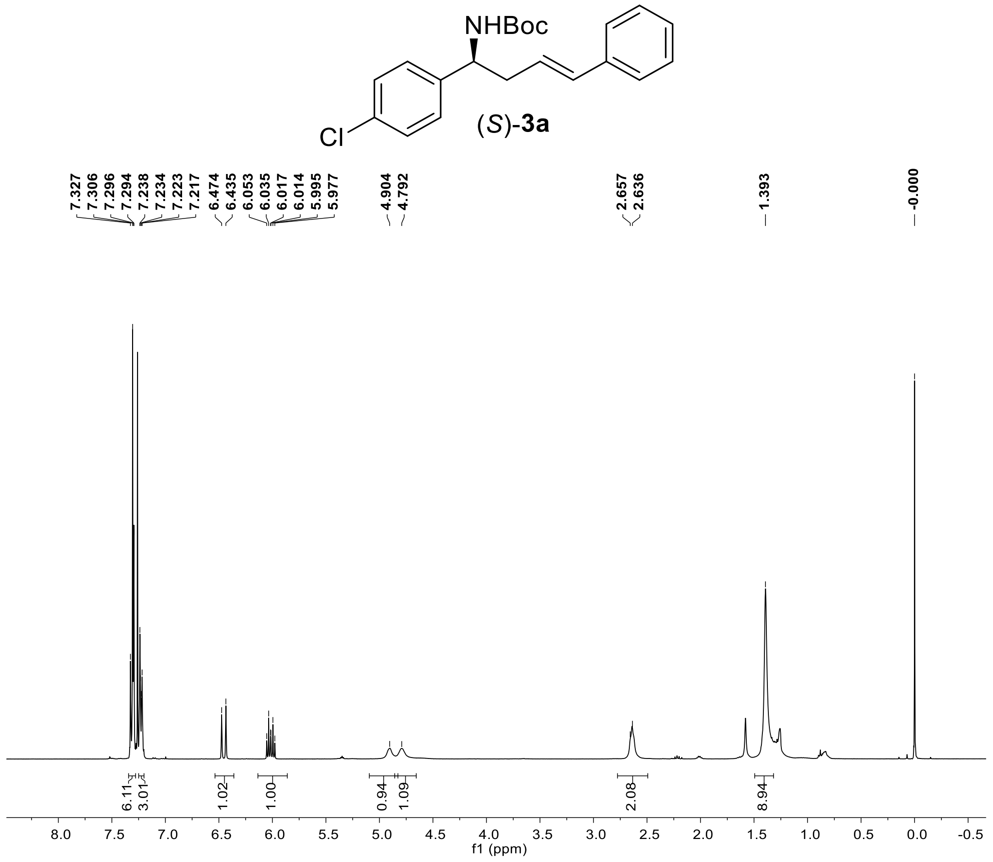

Supplementary Figure 1. ^{1}H NMR spectrum of (*S*)-**3a**

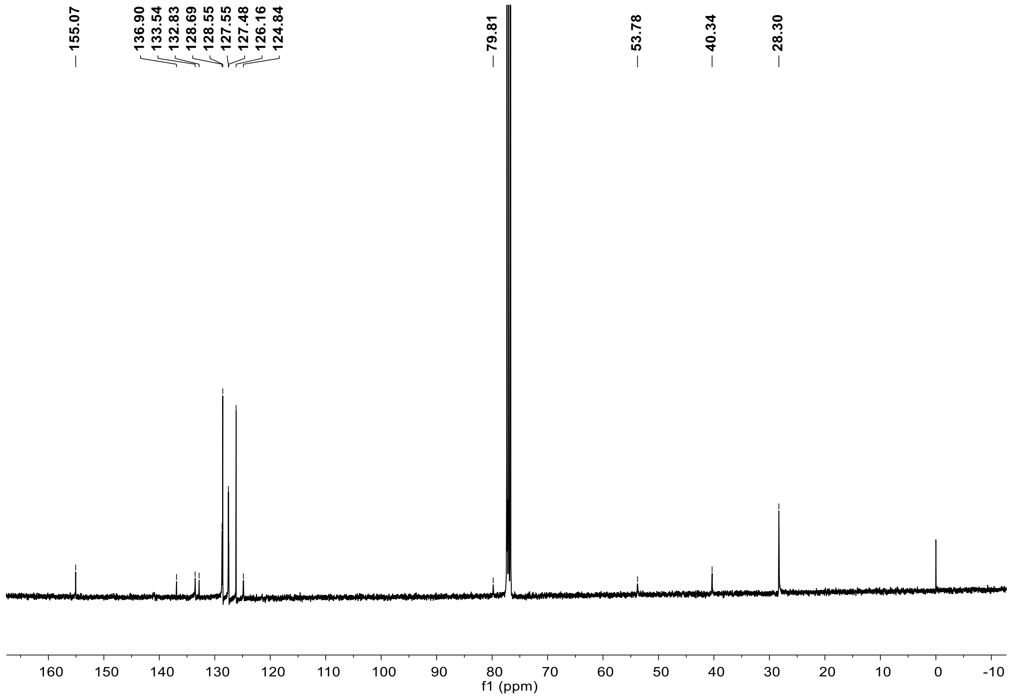

Supplementary Figure 2. ^{13}C NMR spectrum of (*S*)-**3a**

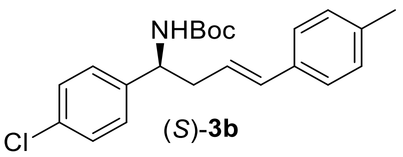

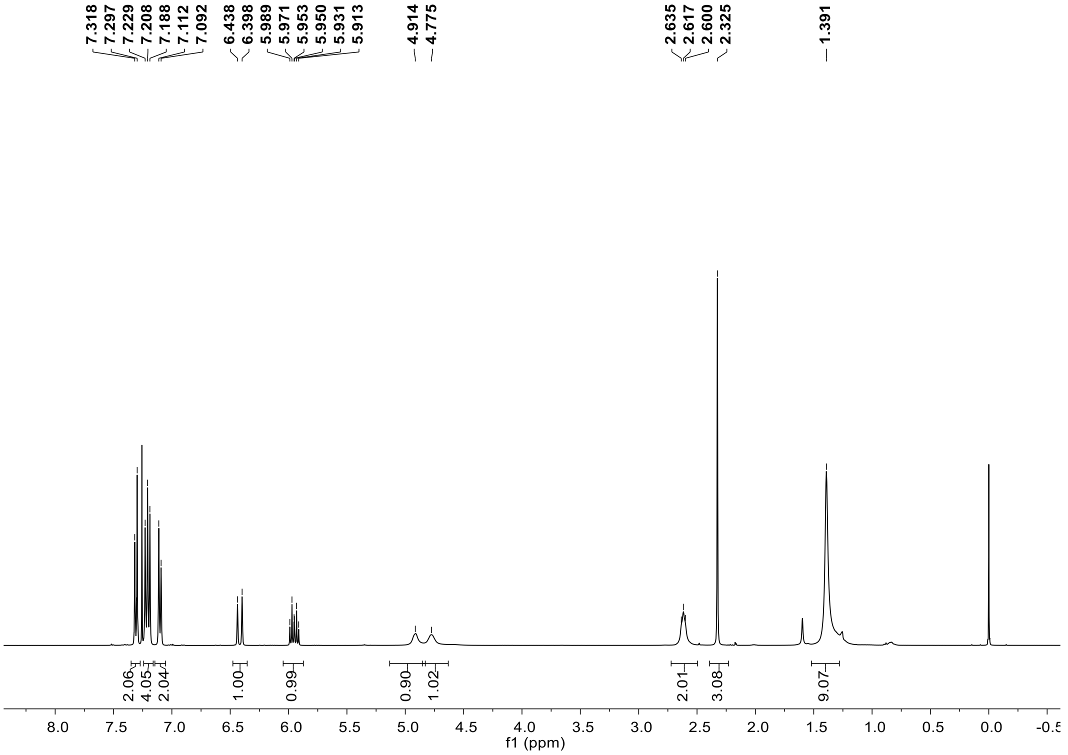

Supplementary Figure 3. ^{1}H NMR spectrum of (*S*)-**3b**

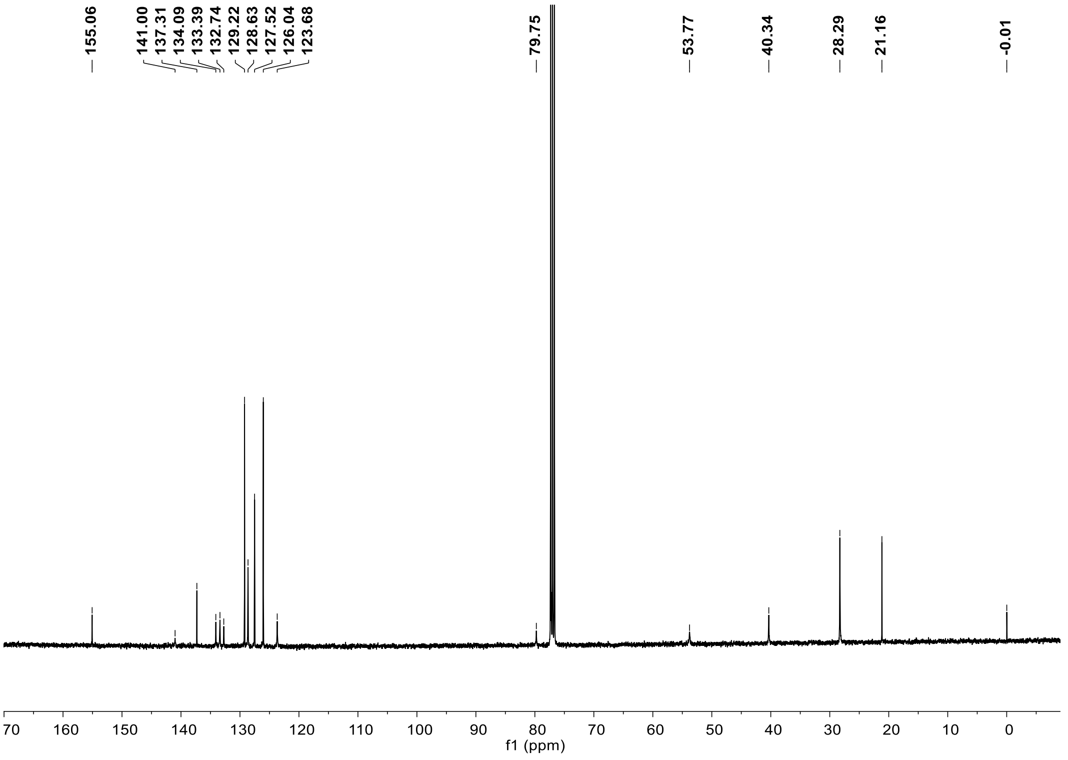

Supplementary Figure 4. ^{13}C NMR spectrum of (*S*)-**3b**

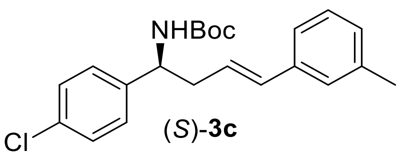

(*S*)-**3c**

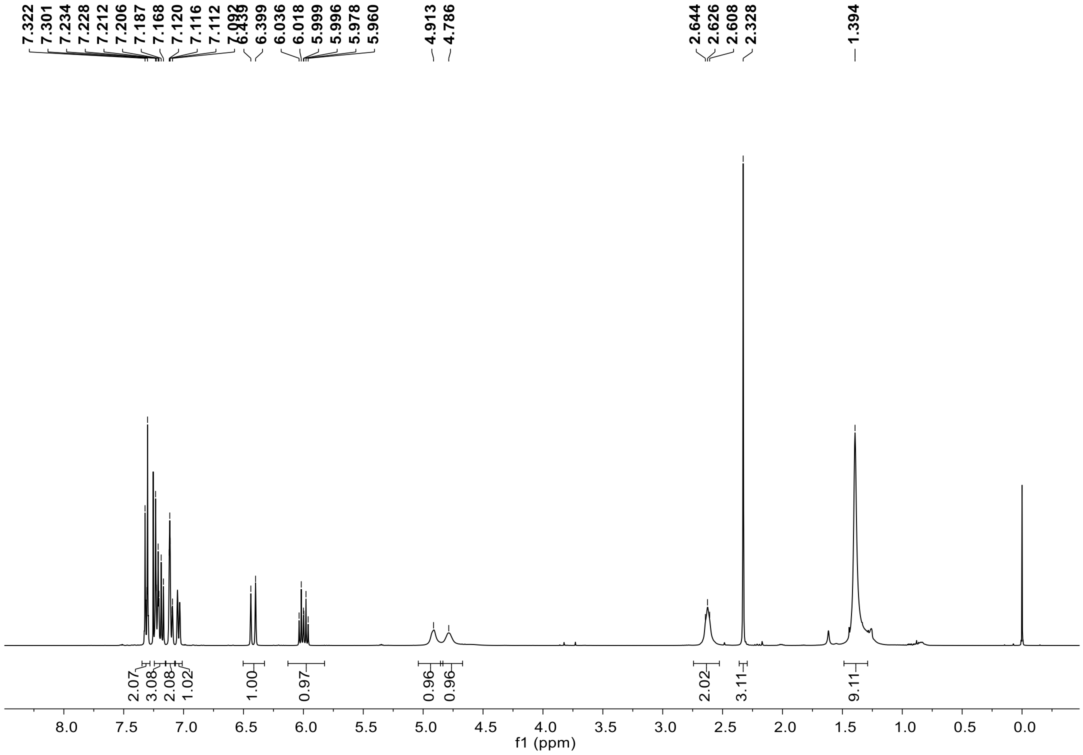

Supplementary Figure 5. ^{1}H NMR spectrum of (*S*)-**3c**

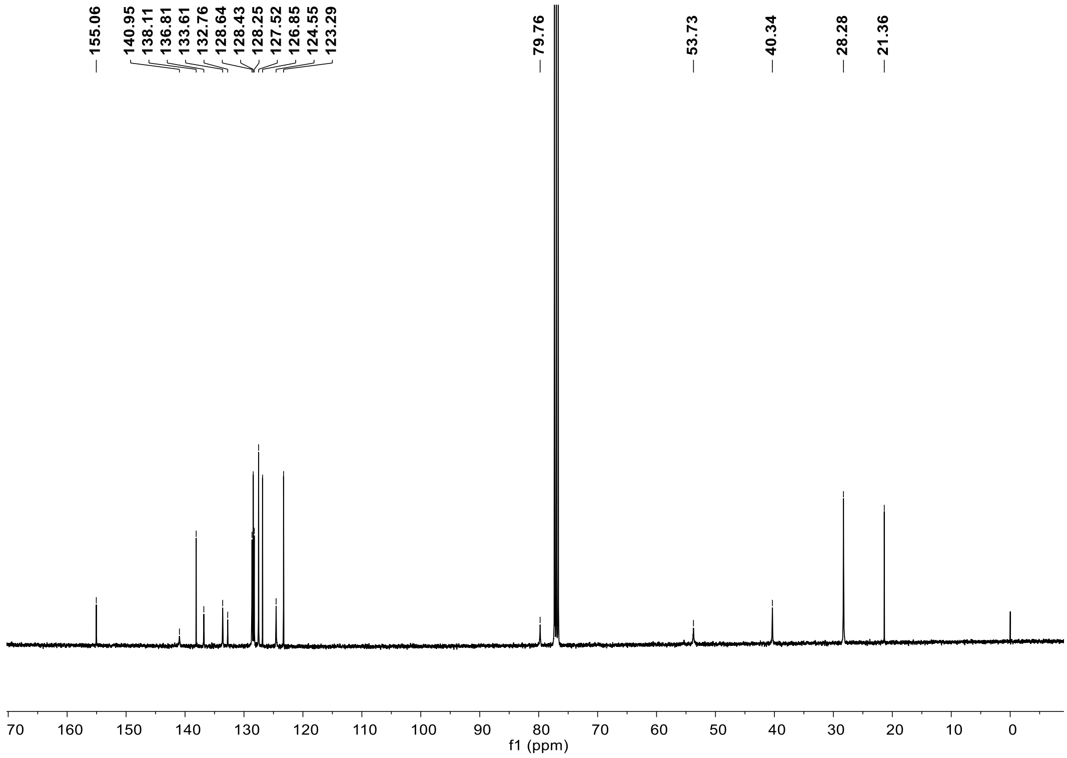

Supplementary Figure 6. ^{13}C NMR spectrum of (*S*)-**3c**

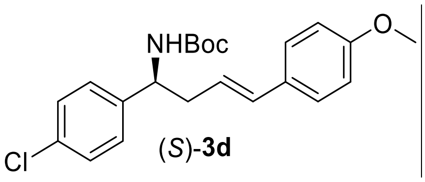

(*S*)-**3d**

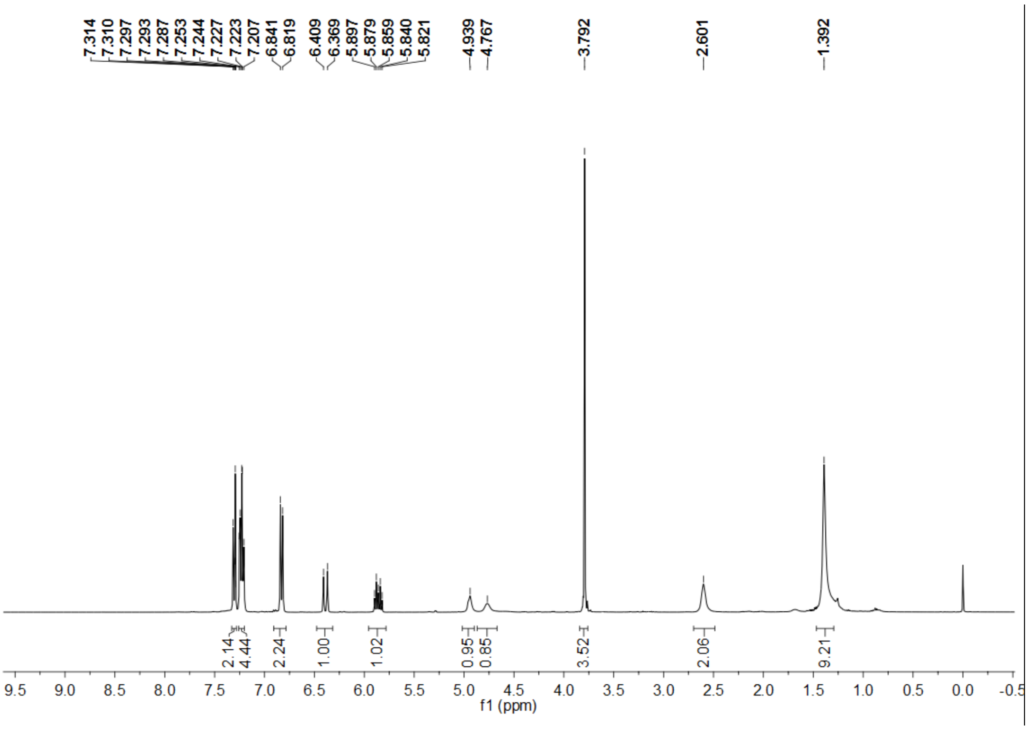

Supplementary Figure 7. ^{1}H NMR spectrum of (*S*)-**3d**

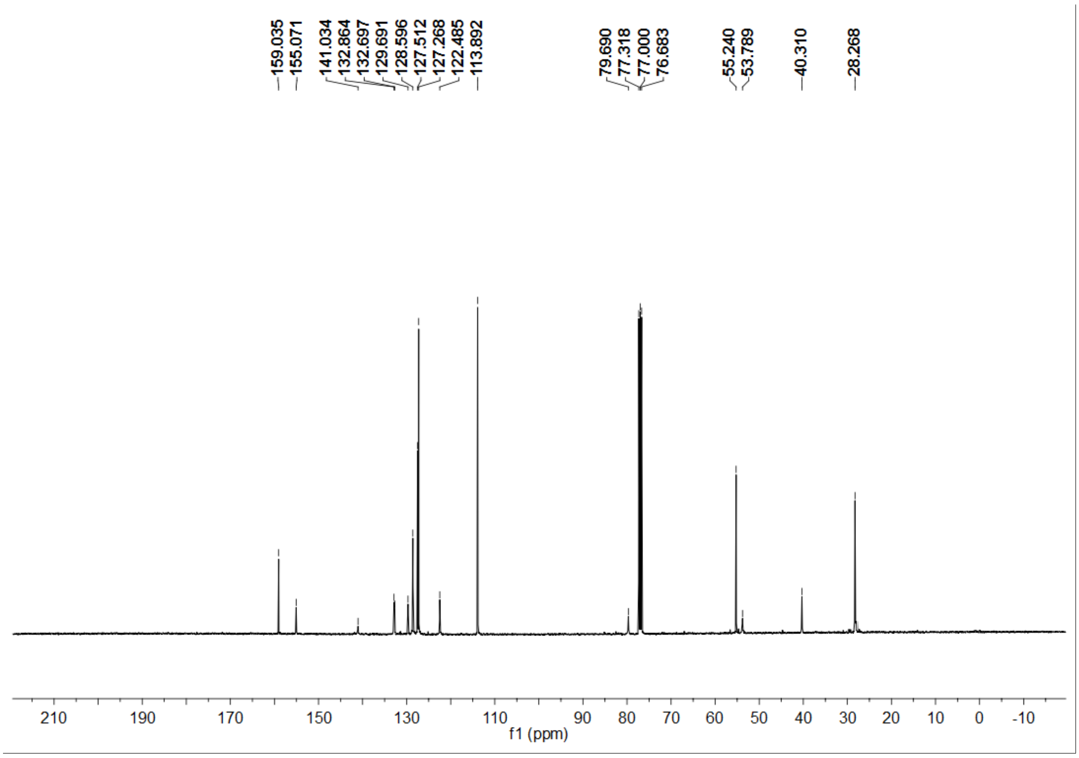

Supplementary Figure 8. ^{13}C NMR spectrum of (*S*)-**3d**

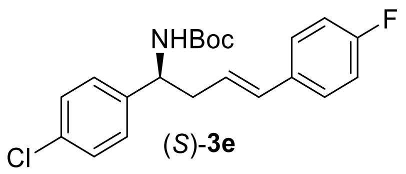

(*S*)-**3e**

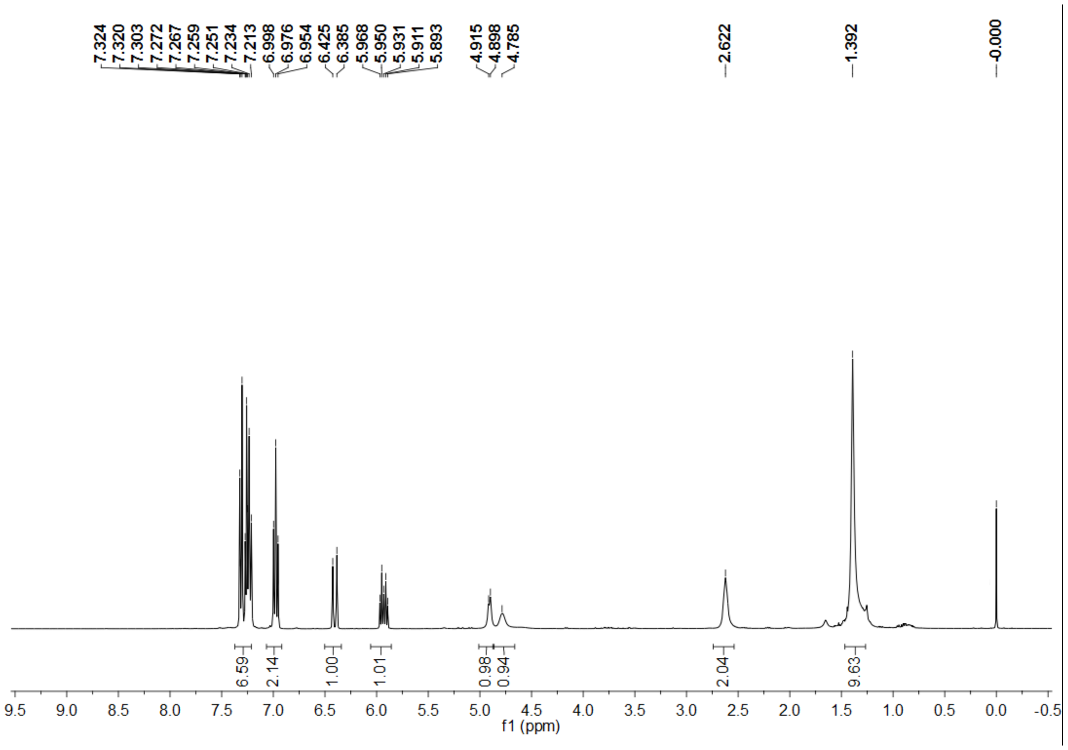

Supplementary Figure 9. ^{1}H NMR spectrum of (*S*)-**3e**

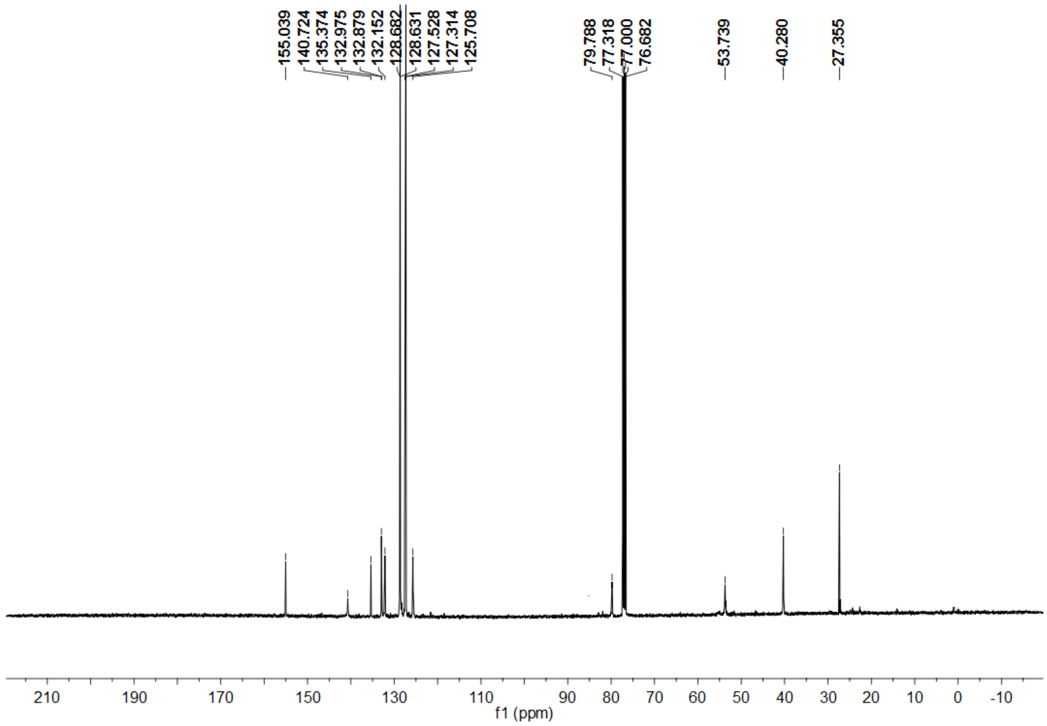

Supplementary Figure 10. ^{1}H NMR spectrum of (*S*)-**3e**

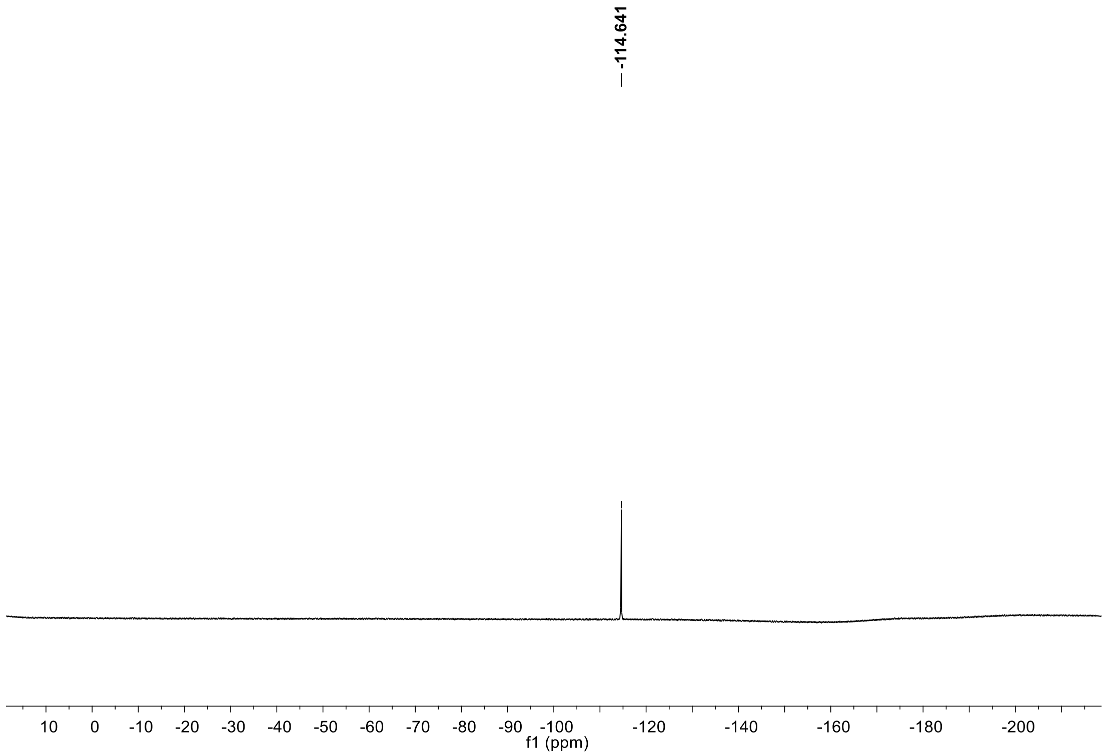

Supplementary Figure 11. ^{19}F NMR spectrum of (*S*)-**3e**

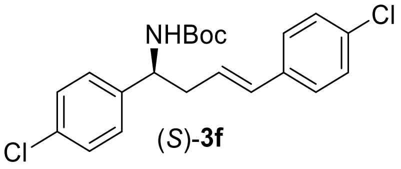

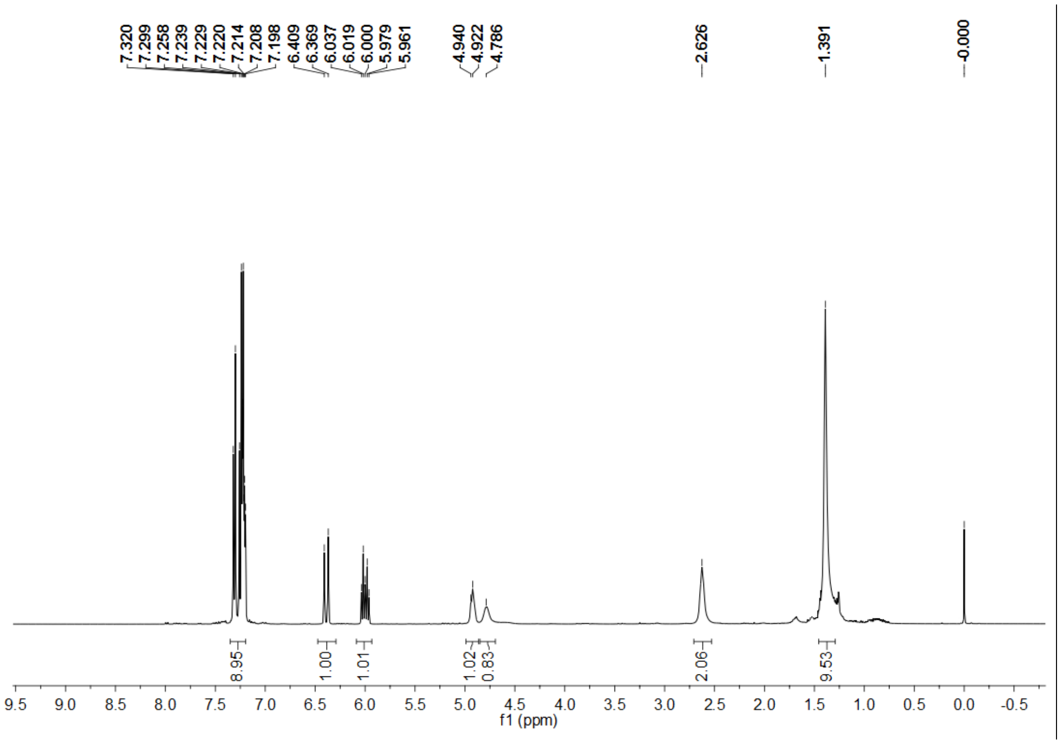

Supplementary Figure 12. ^{1}H NMR spectrum of (*S*)-**3f**

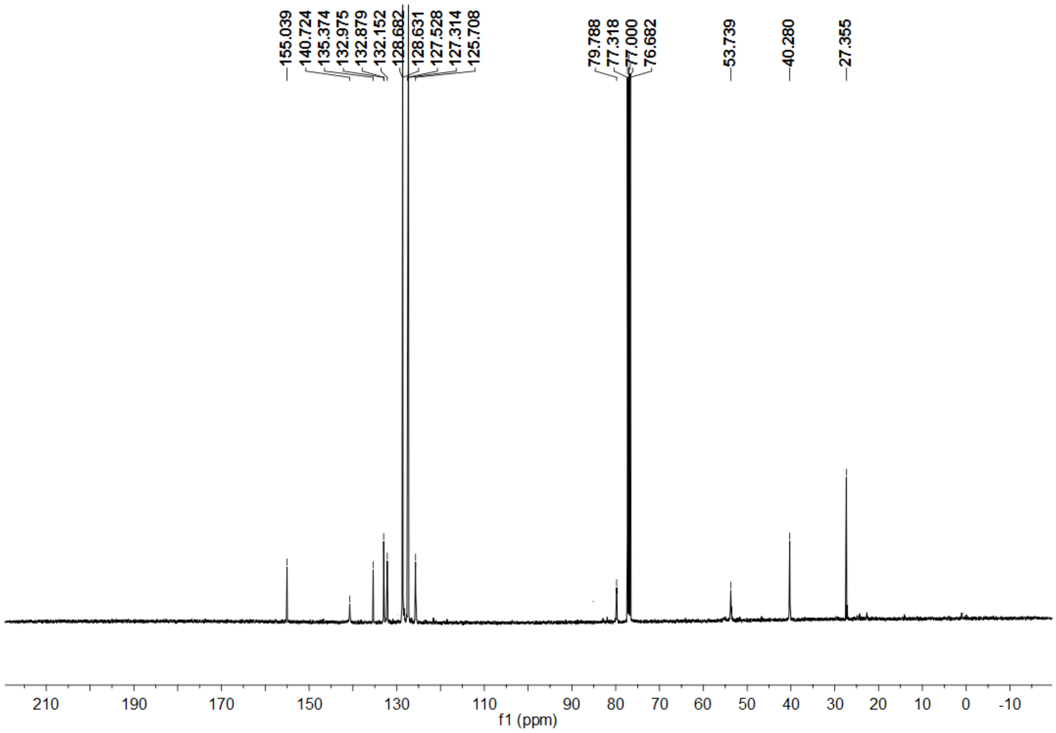

Supplementary Figure 13. ^{13}C NMR spectrum of (*S*)-**3f**

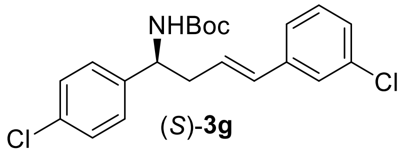

(*S*)-**3g**

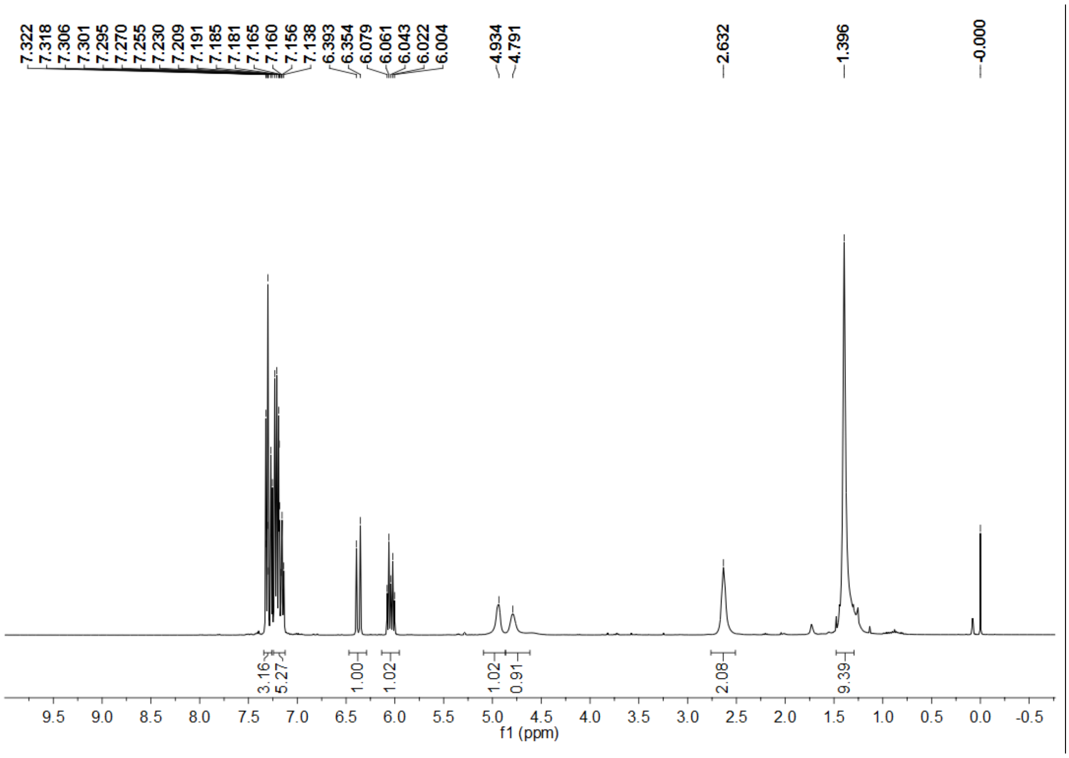

Supplementary Figure 14. ^{1}H NMR spectrum of (*S*)-**3g**

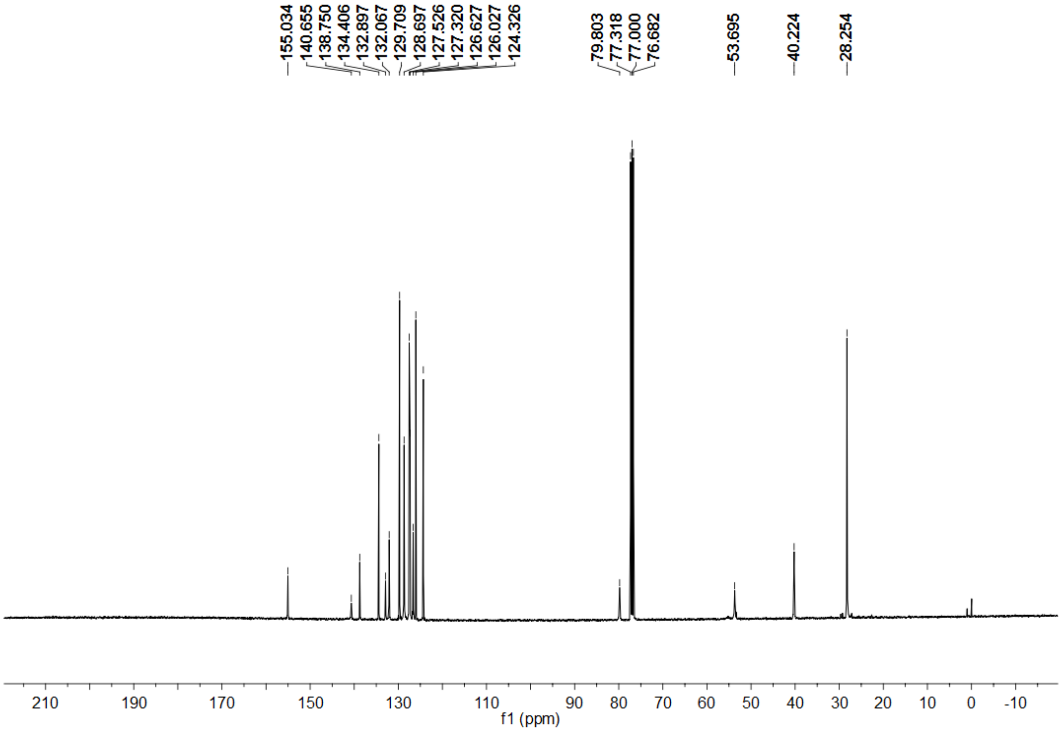

Supplementary Figure 15. ^{1}H NMR spectrum of (*S*)-**3g**

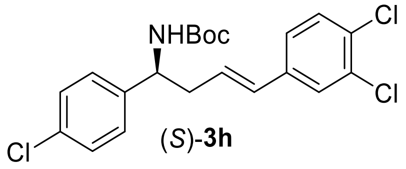

(*S*)-**3h**

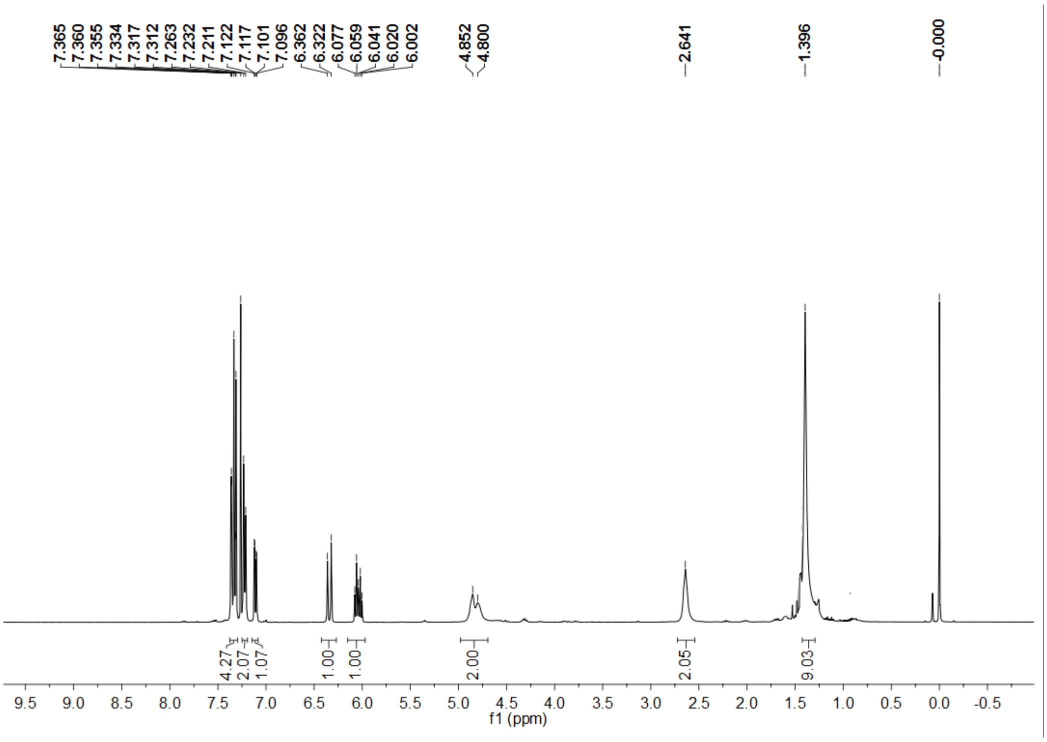

Supplementary Figure 16. ^{1}H NMR spectrum of (*S*)-**3h**

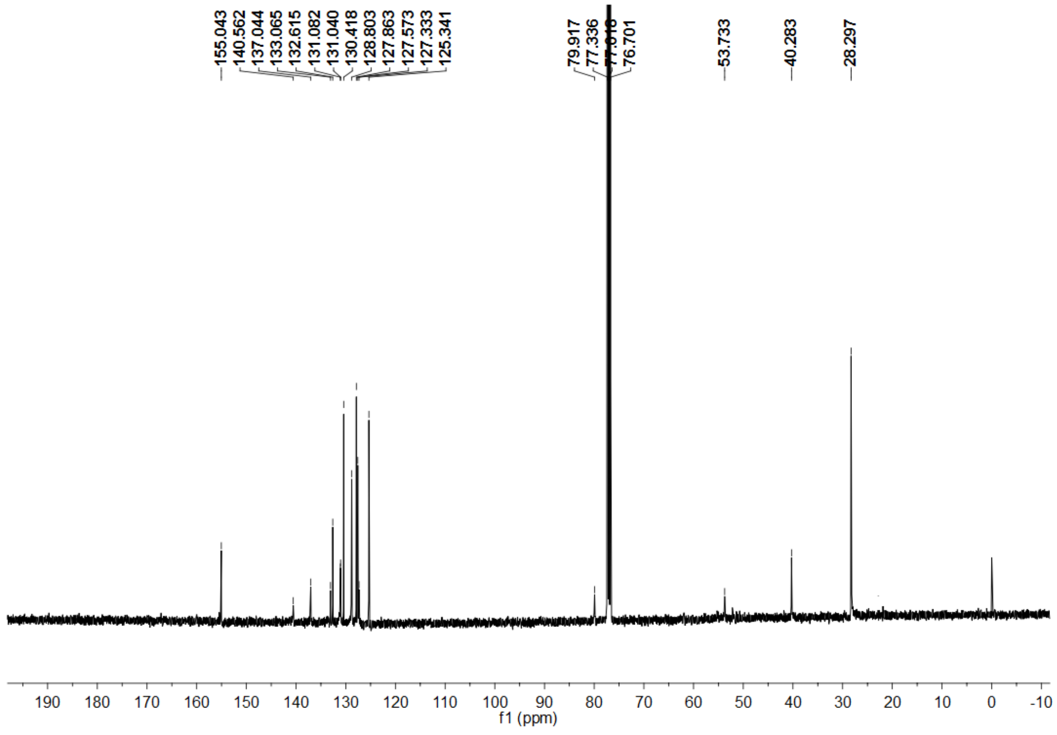

Supplementary Figure 17. ^{13}C NMR spectrum of (*S*)-**3h**

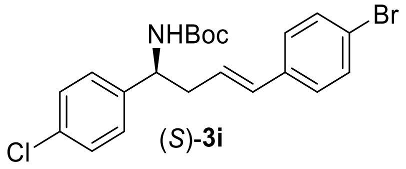

(*S*)-**3i**

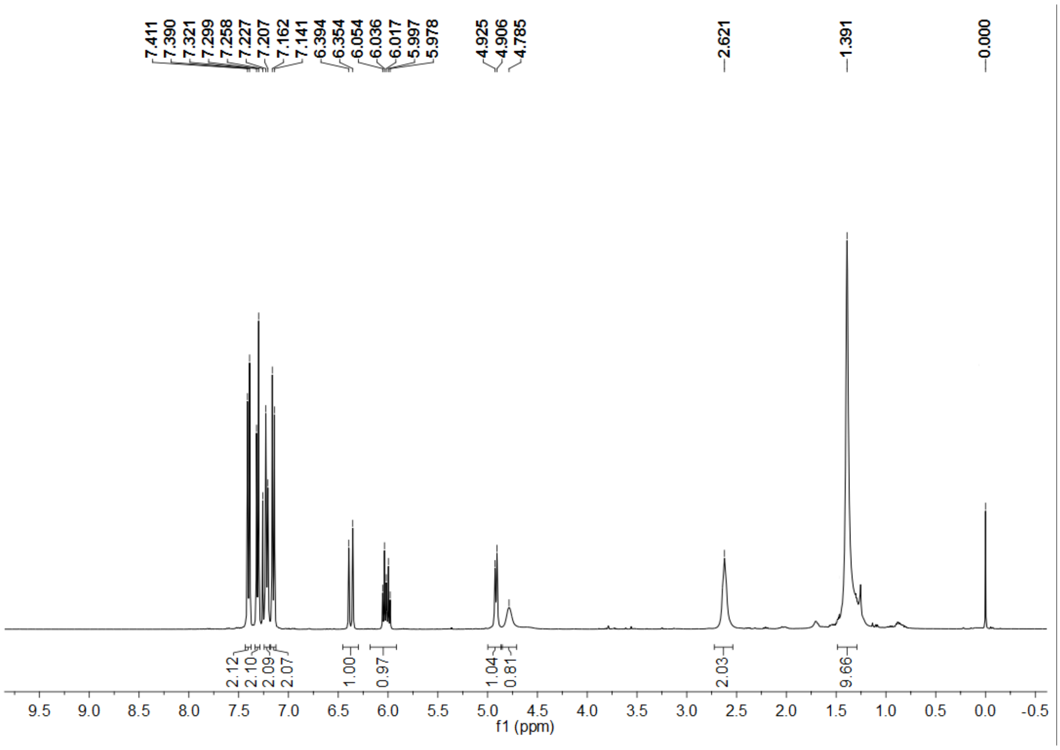

Supplementary Figure 18. ^{1}H NMR spectrum of (*S*)-**3i**

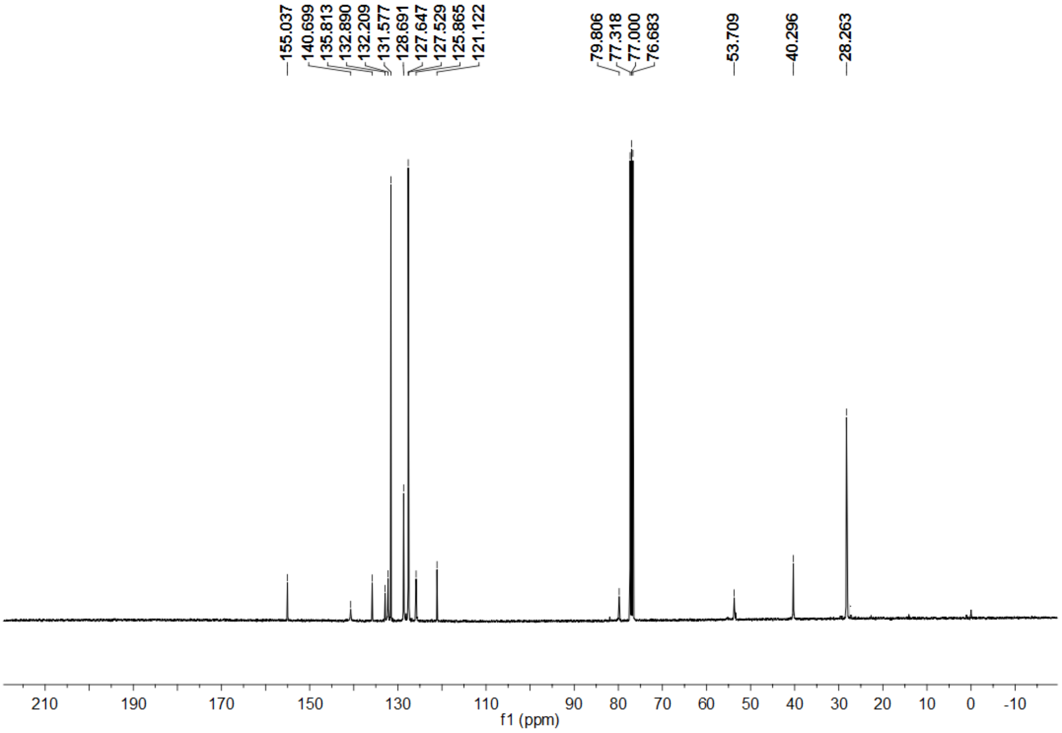

Supplementary Figure 19. ^{13}C NMR spectrum of (*S*)-**3i**

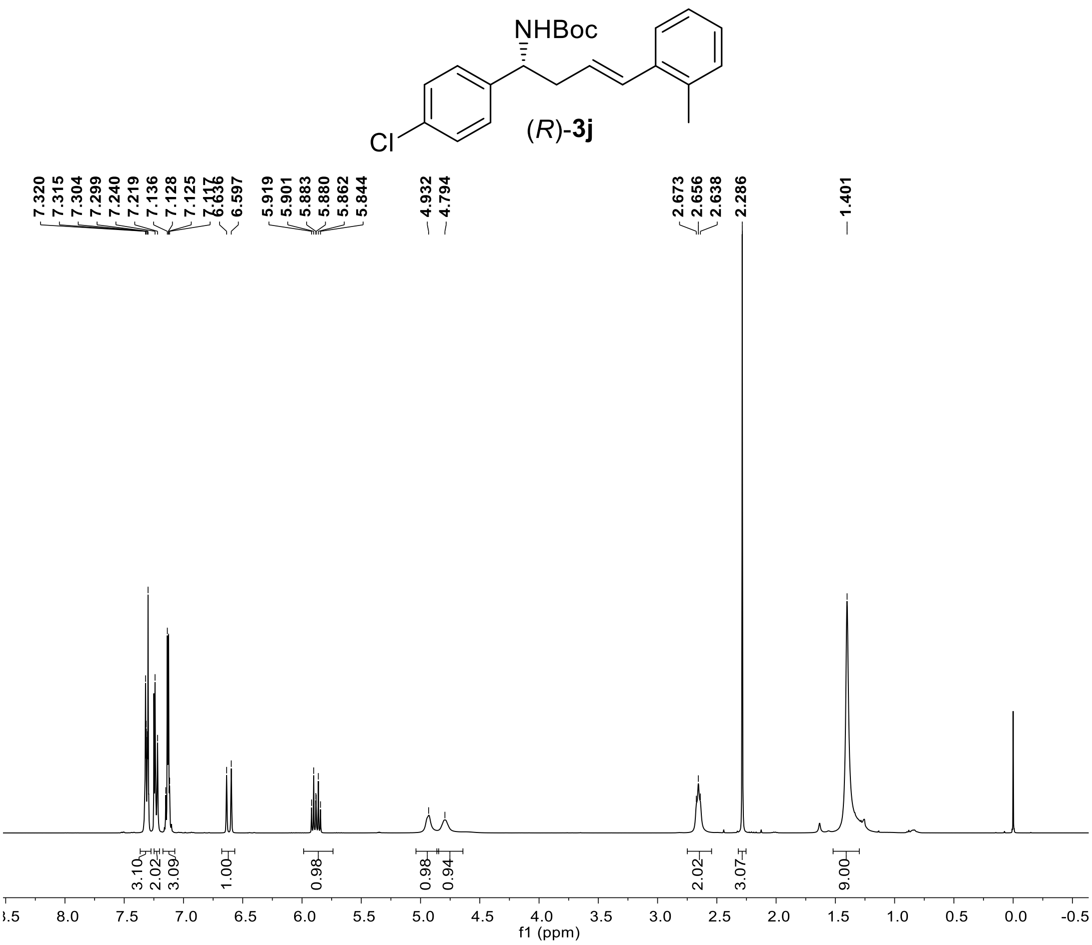

Supplementary Figure 20. ^{1}H NMR spectrum of (*S*)-**3j**

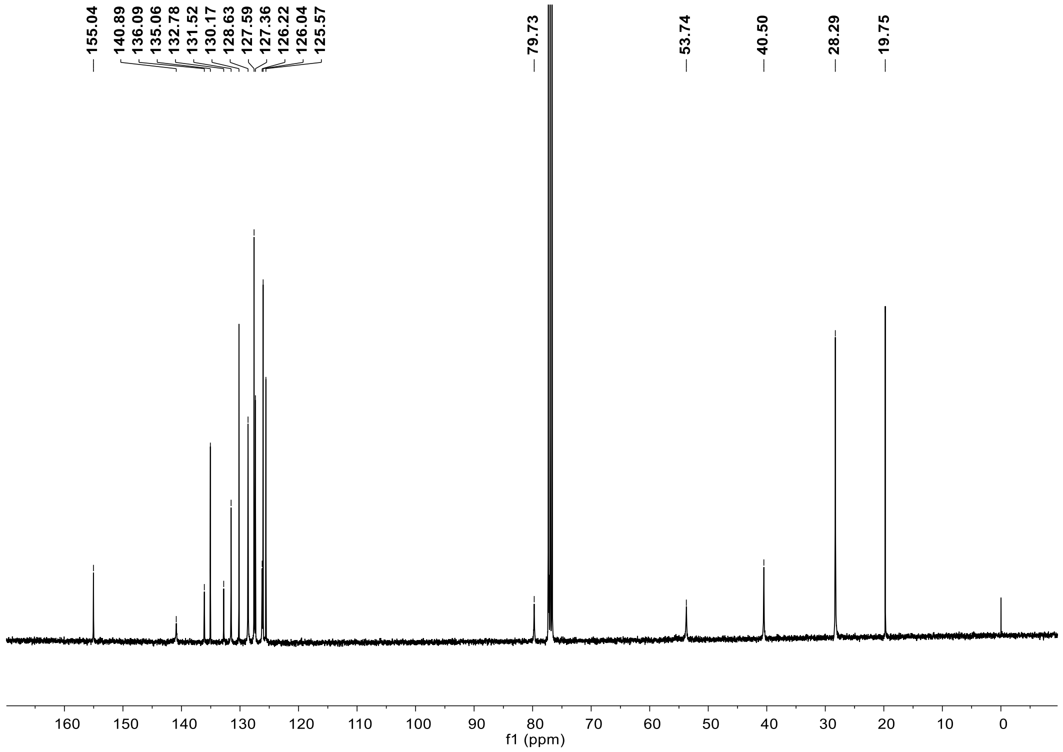

Supplementary Figure 21. ^{1}H NMR spectrum of (*S*)-**3j**

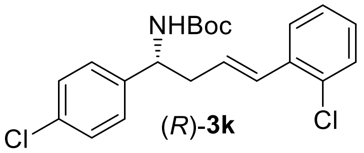

(*R*)-**3k**

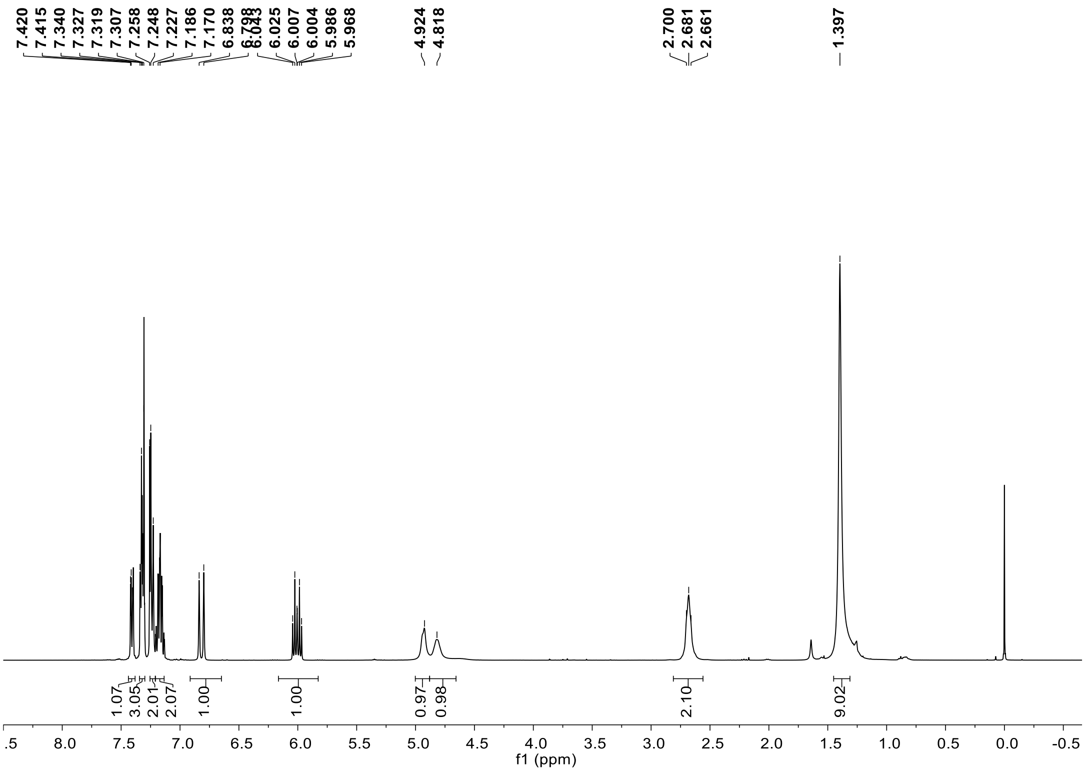

Supplementary Figure 22. ^{1}H NMR spectrum of (*R*)-**3k**

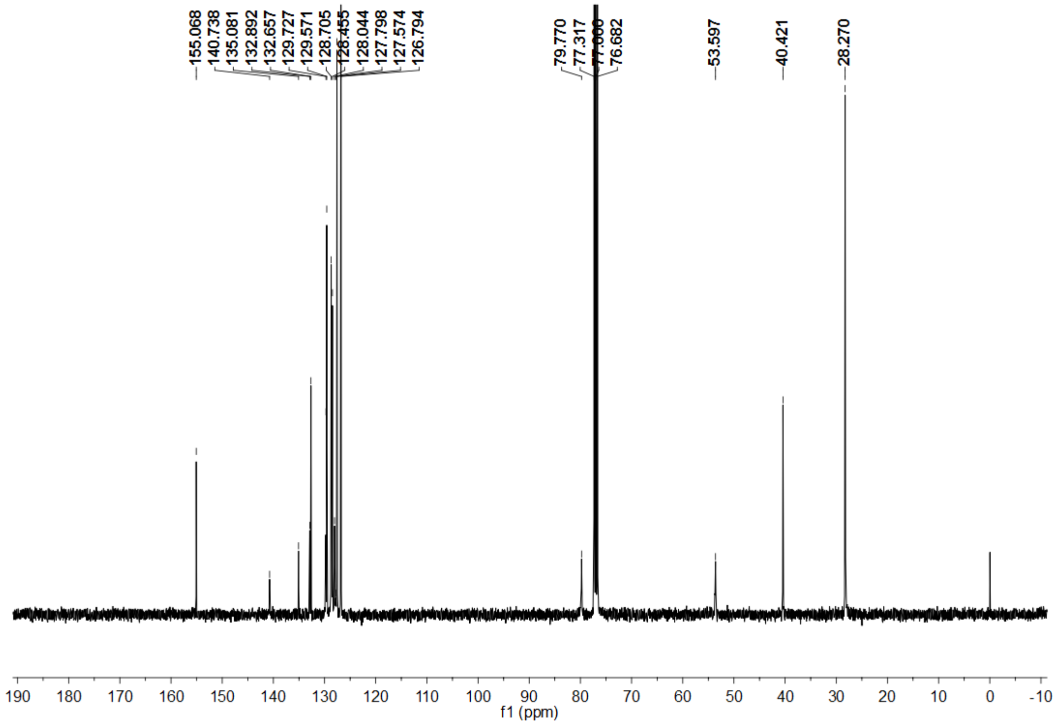

Supplementary Figure 23. ^{13}C NMR spectrum of (*R*)-**3k**

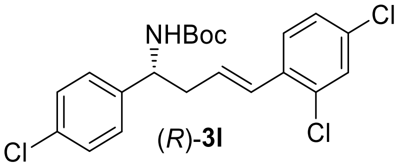

(*R*)-**3l**

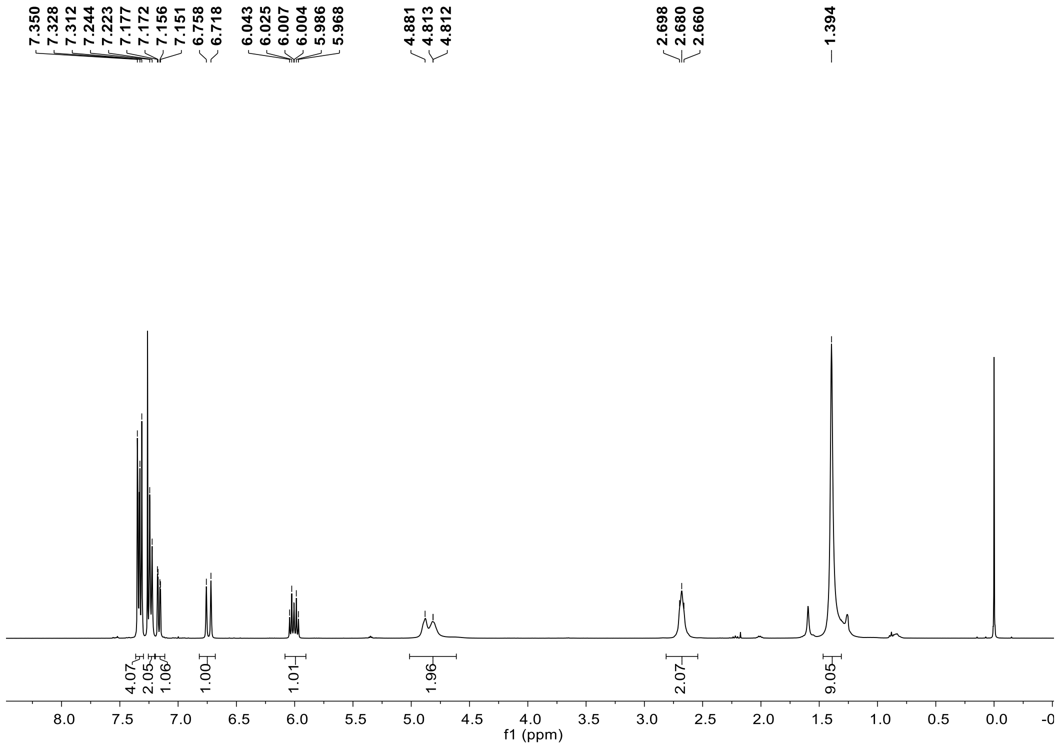

Supplementary Figure 24. ^{1}H NMR spectrum of (*R*)-**3l**

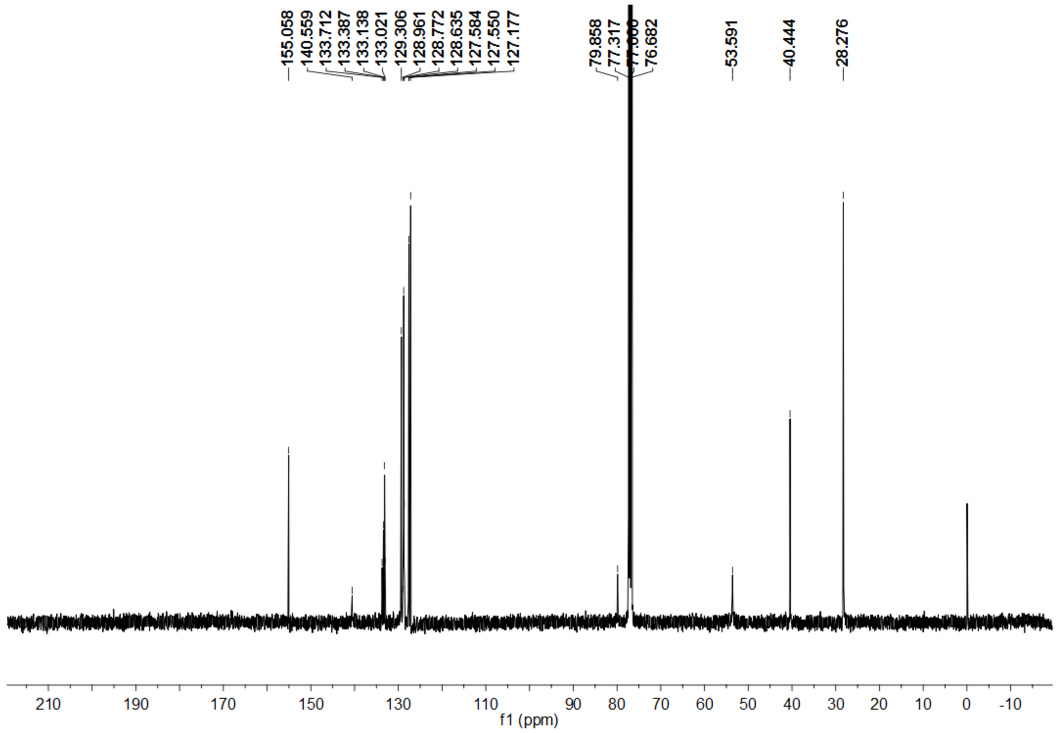

Supplementary Figure 25. ^{1}H NMR spectrum of (*R*)-**3l**

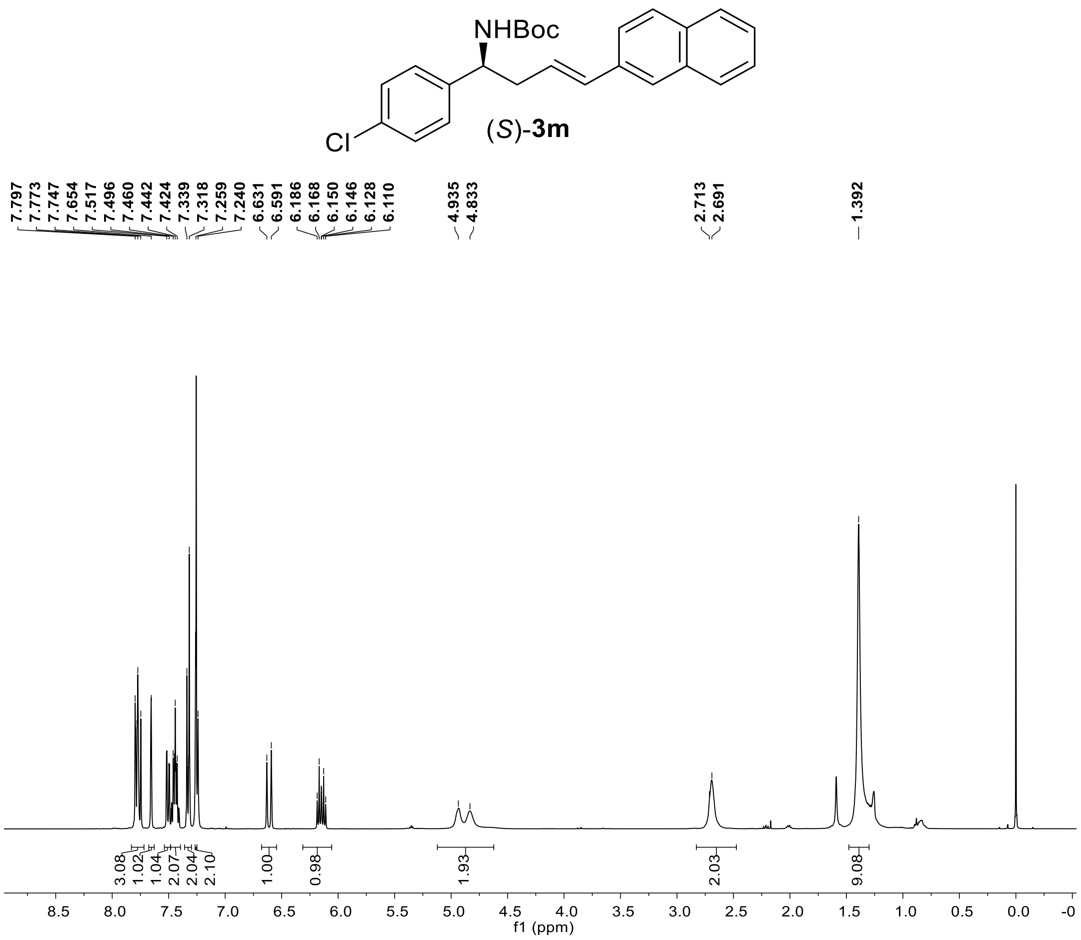

Supplementary Figure 26. ^{1}H NMR spectrum of (*S*)-**3m**

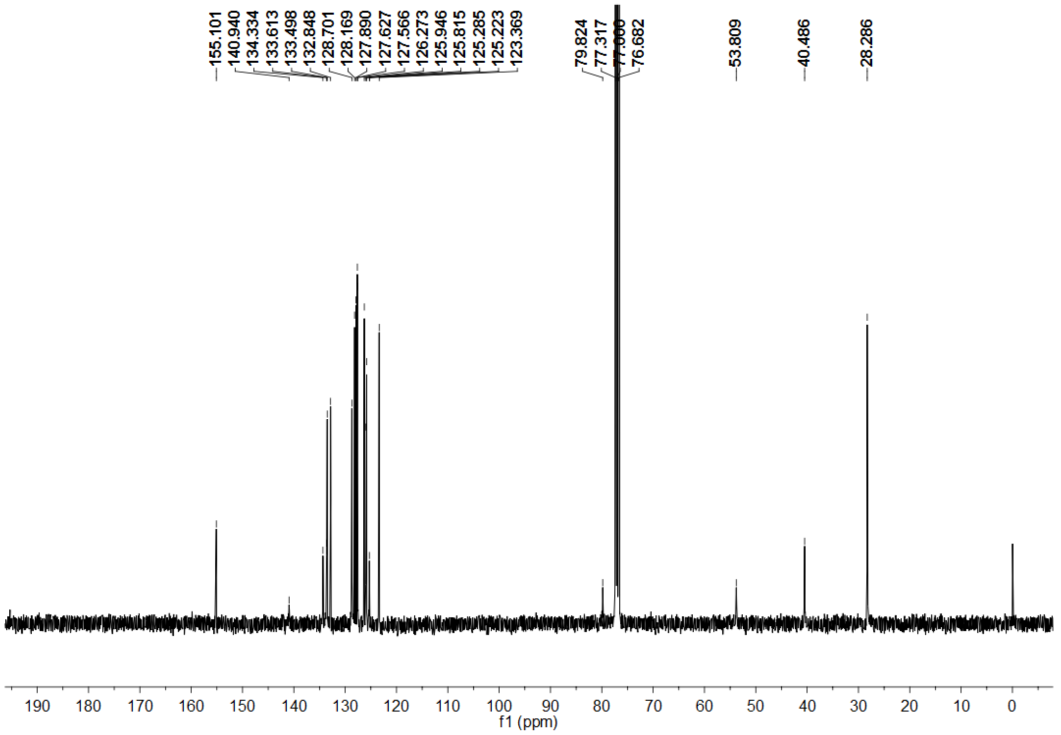

Supplementary Figure 27. ^{13}C NMR spectrum of (*S*)-**3m**

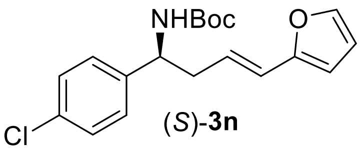

(*S*)-**3n**

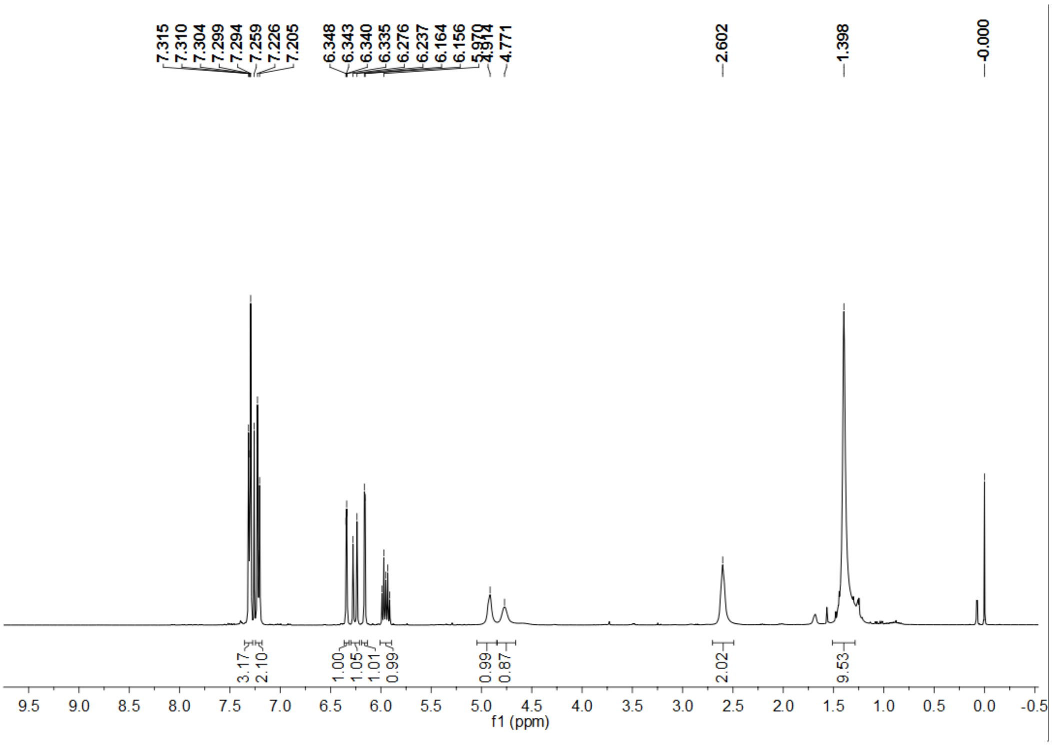

Supplementary Figure 28. ^{1}H NMR spectrum of (*S*)-**3n**

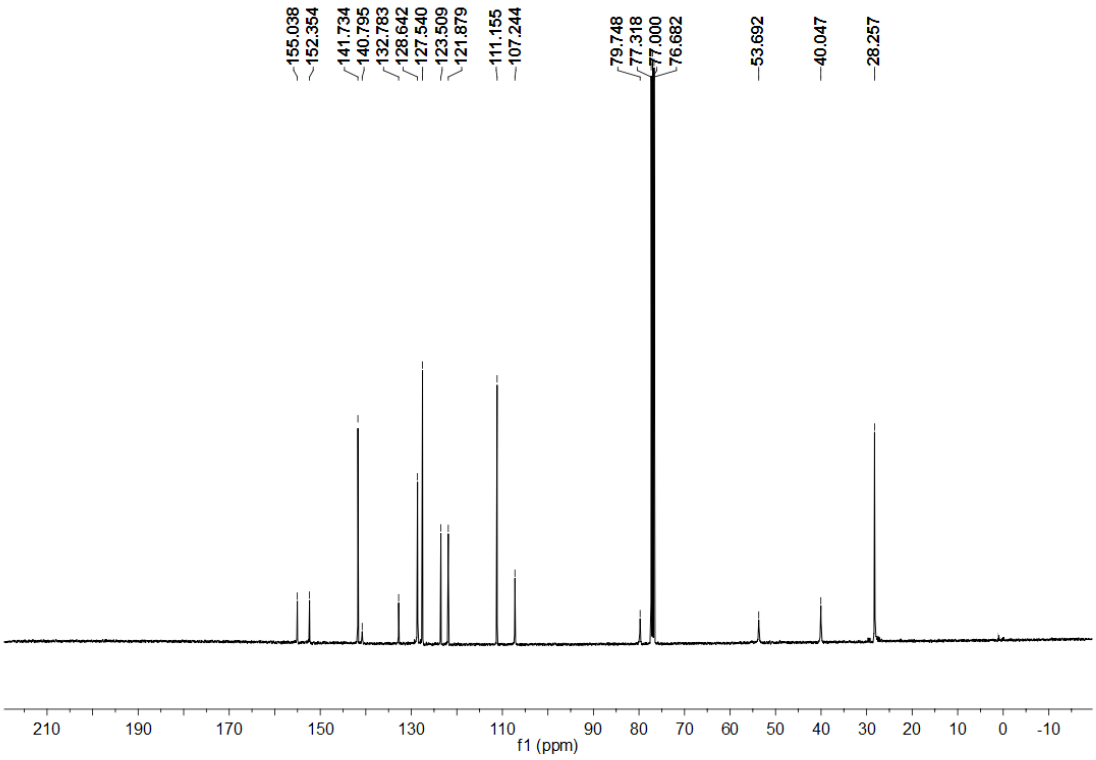

Supplementary Figure 29. ^{13}C NMR spectrum of (*S*)-**3n**

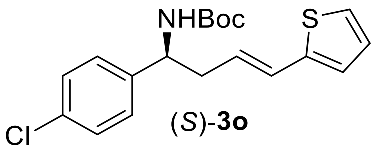

(*S*)-**3o**

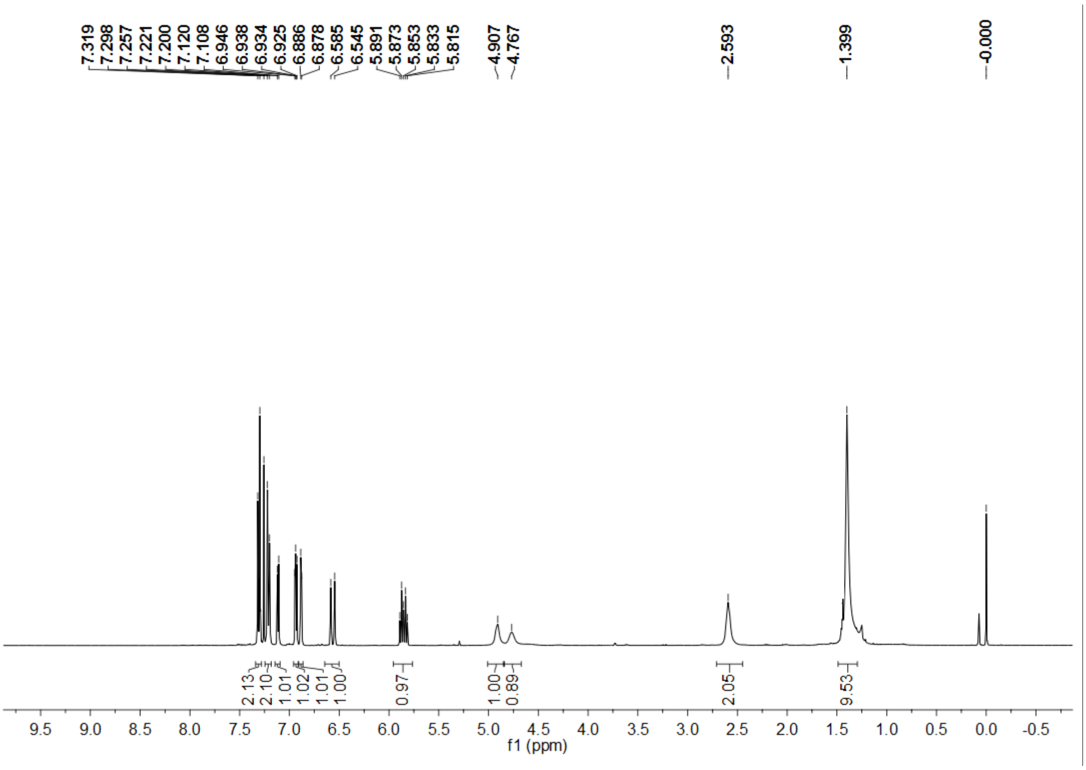

Supplementary Figure 30. ^{1}H NMR spectrum of (*S*)-**3o**

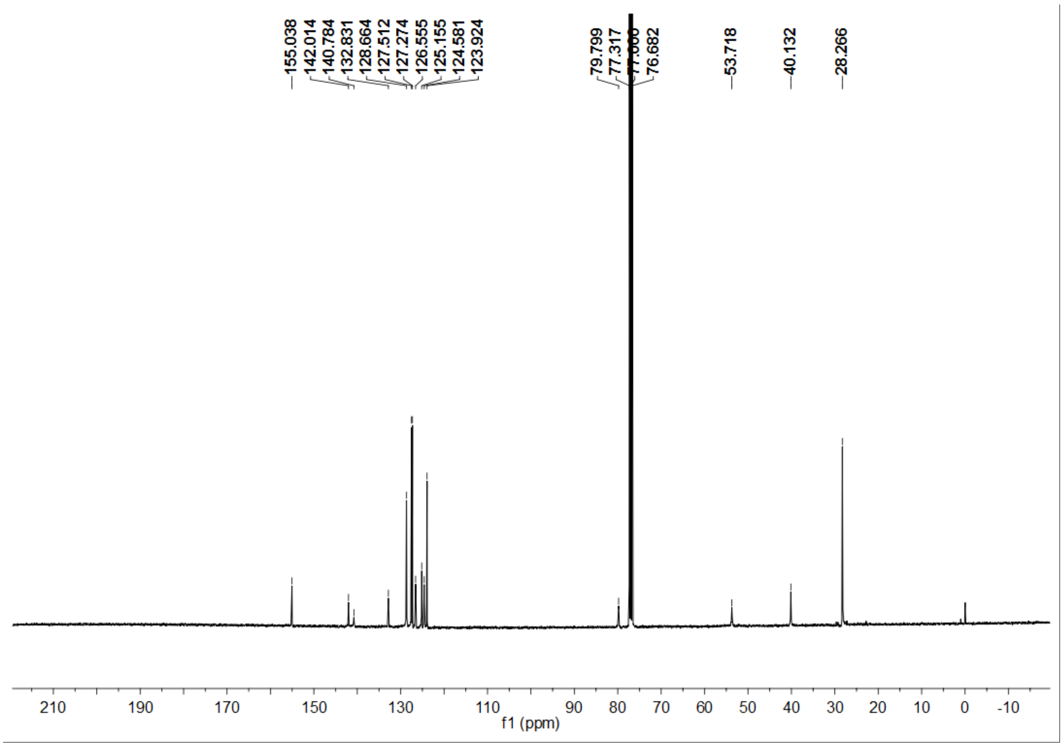

Supplementary Figure 31. ^{13}C NMR spectrum of (*S*)-**3o**

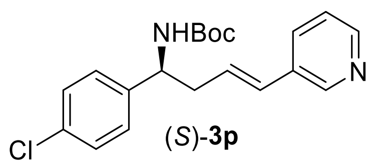

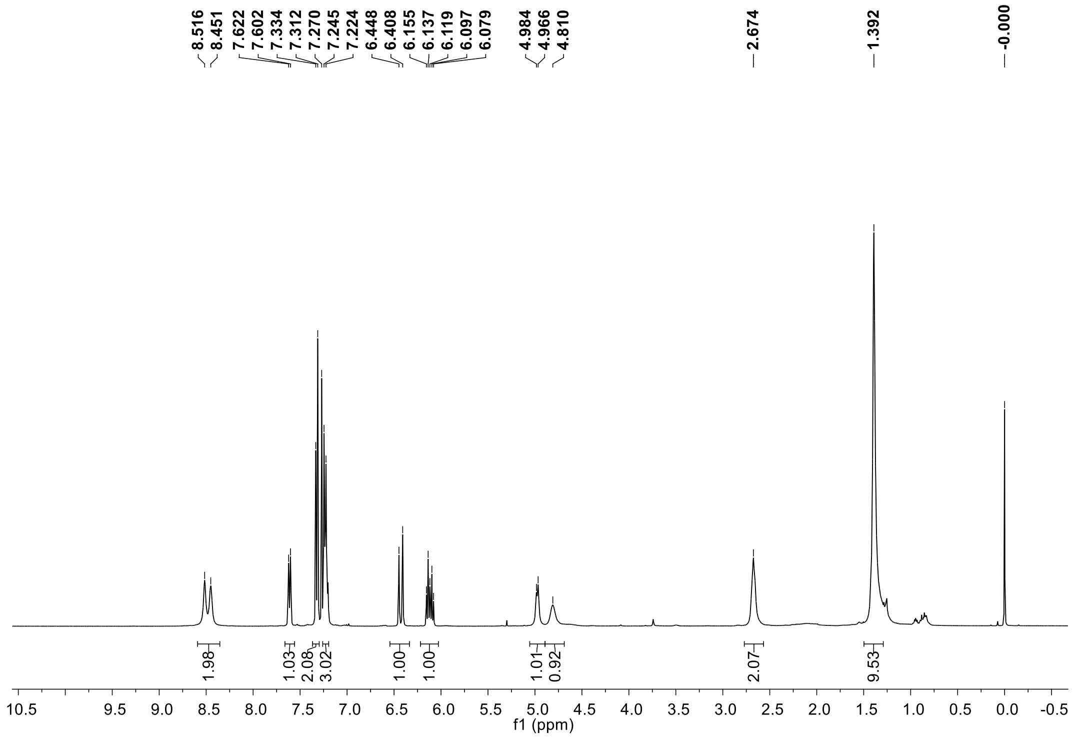

Supplementary Figure 32. ^{1}H NMR spectrum of (*S*)-**3p**

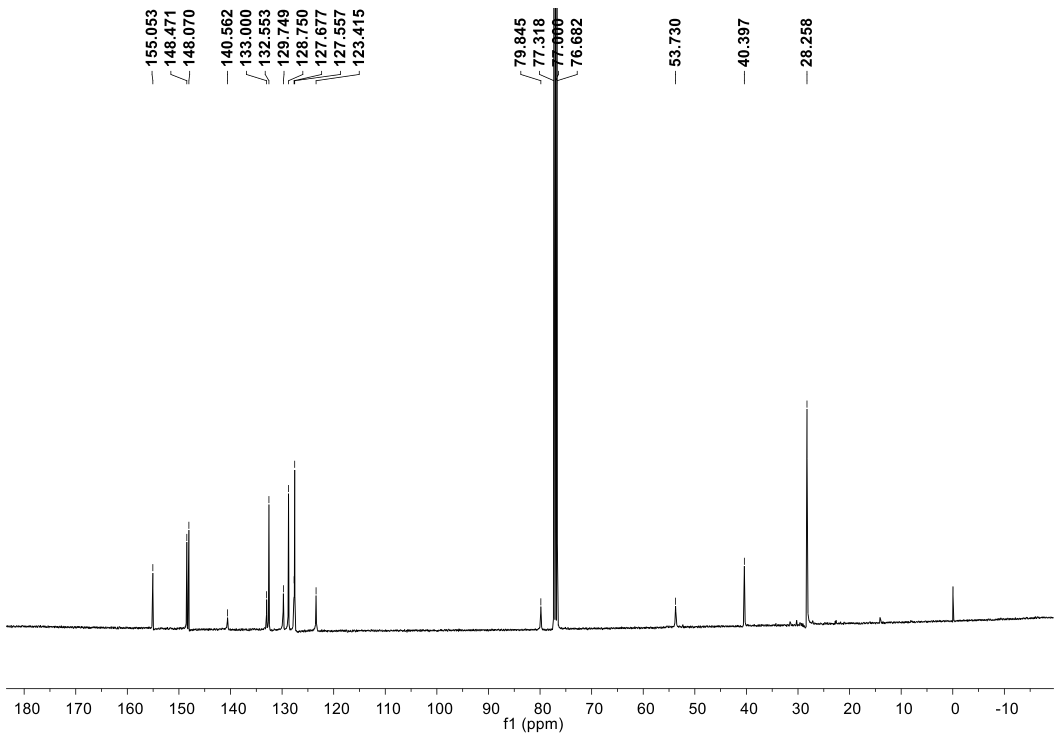

Supplementary Figure 33. ^{13}C NMR spectrum of (*S*)-**3p**

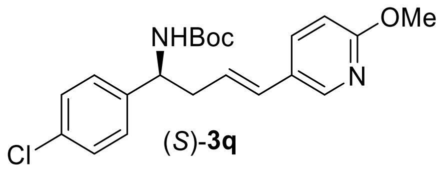

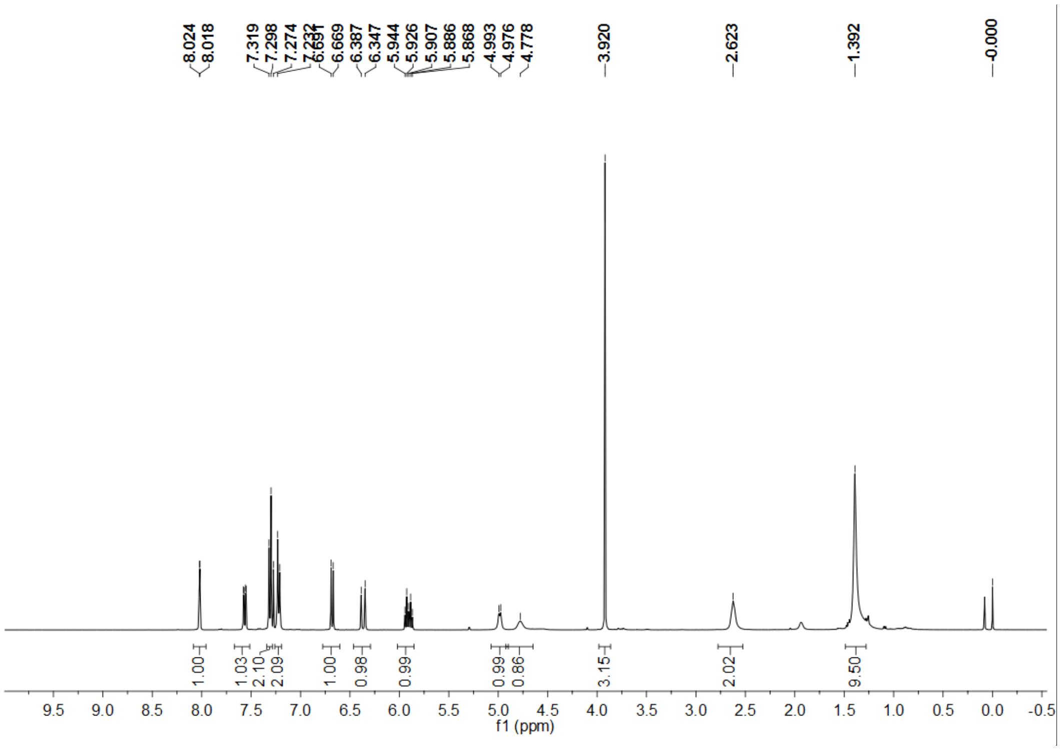

Supplementary Figure 34. ^{1}H NMR spectrum of (*S*)-**3q**

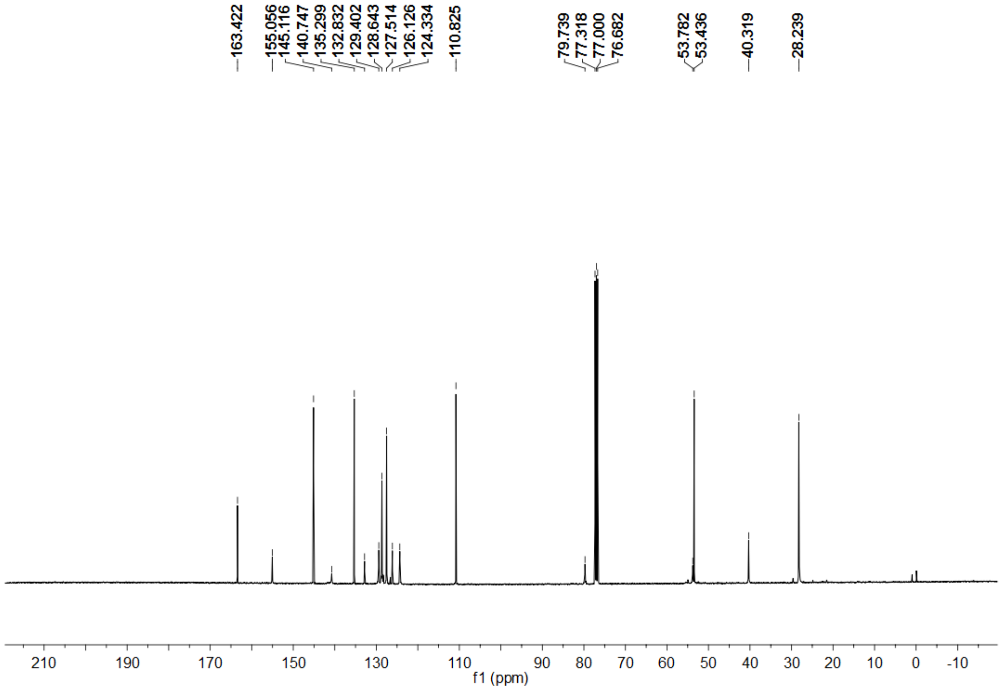

Supplementary Figure 35. ^{13}C NMR spectrum of (*S*)-**3q**

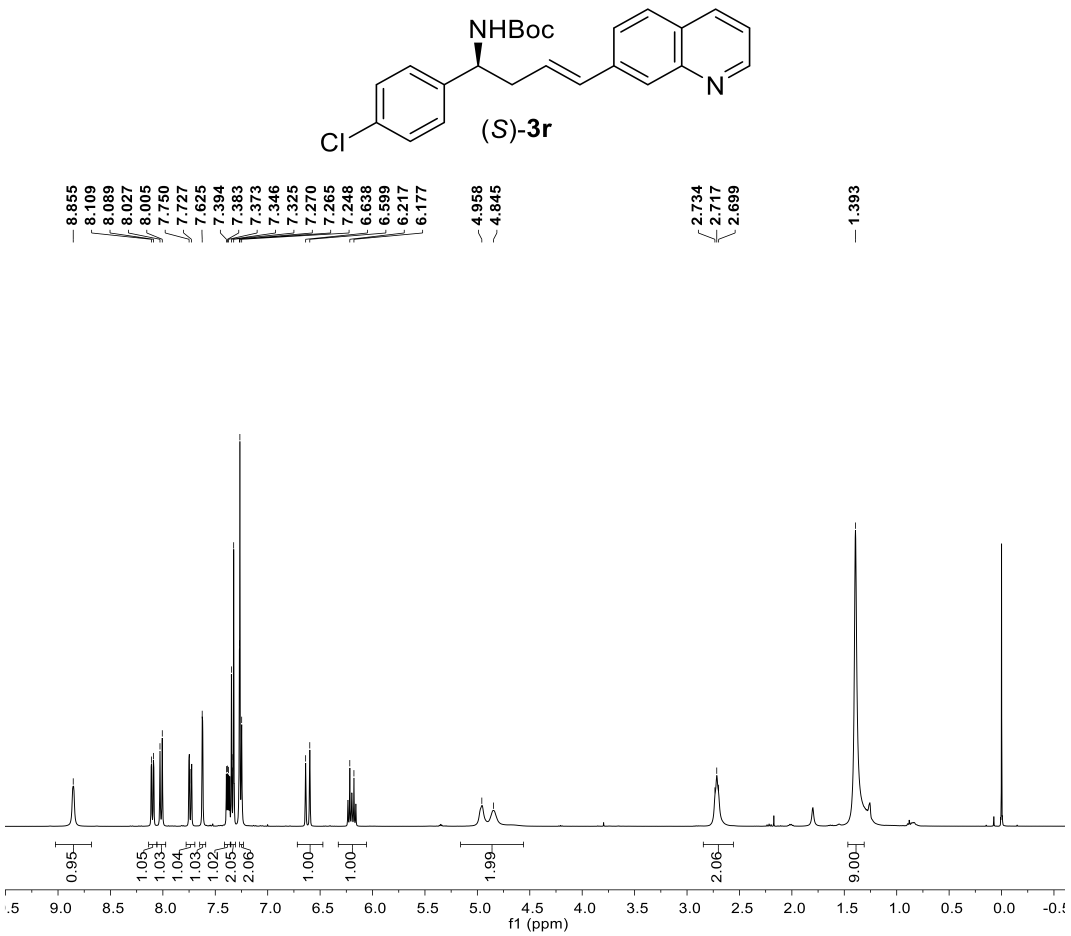

Supplementary Figure 36. ^{1}H NMR spectrum of (*S*)-**3r**

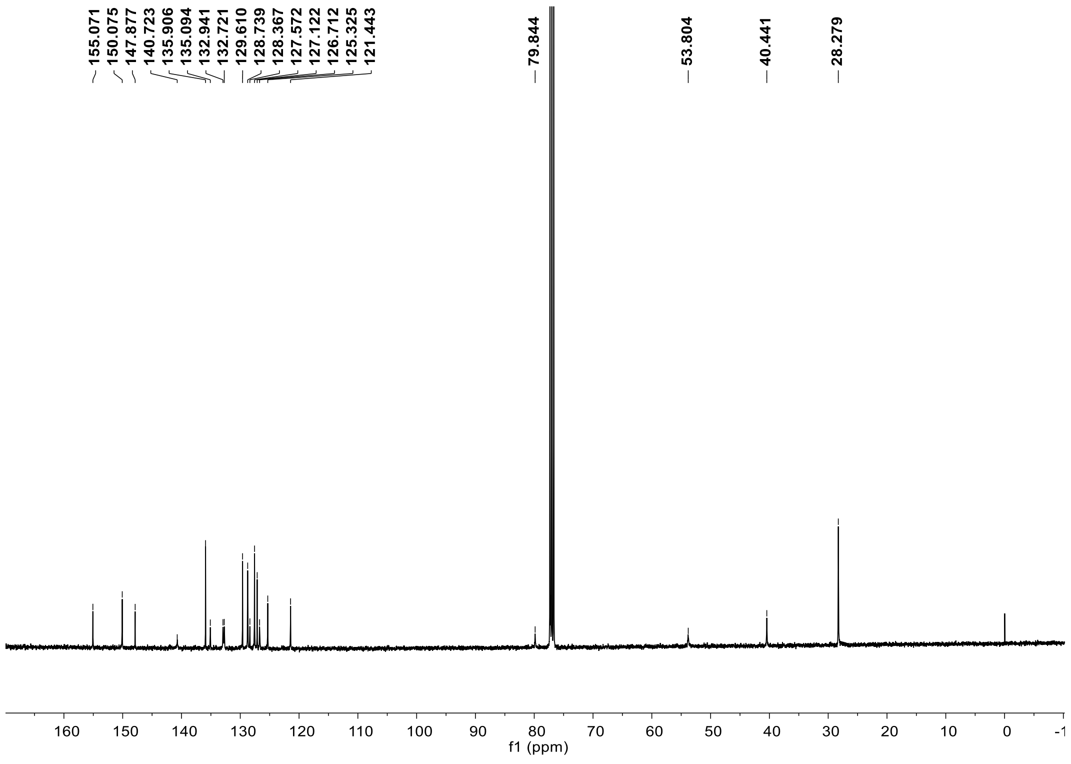

Supplementary Figure 37. ^{13}C NMR spectrum of (*S*)-**3r**

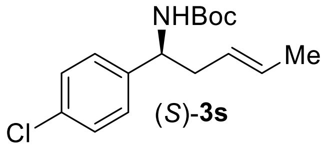

(*S*)-**3s**

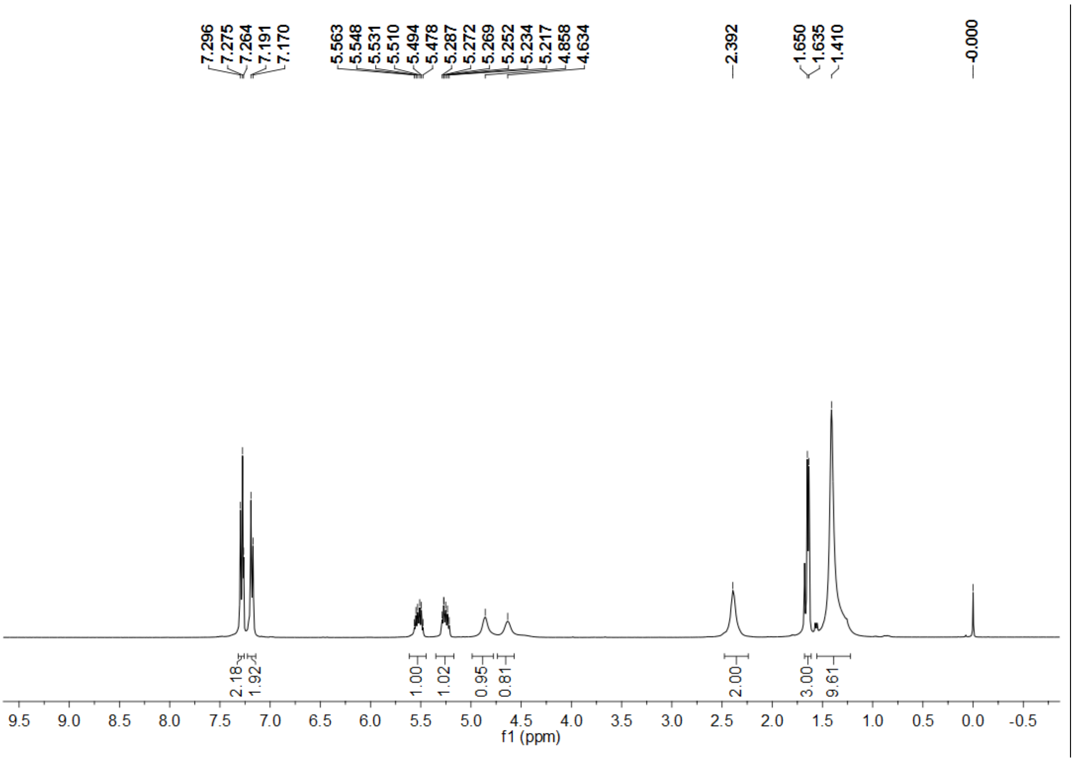

Supplementary Figure 38. ^{1}H NMR spectrum of (*S*)-**3s**

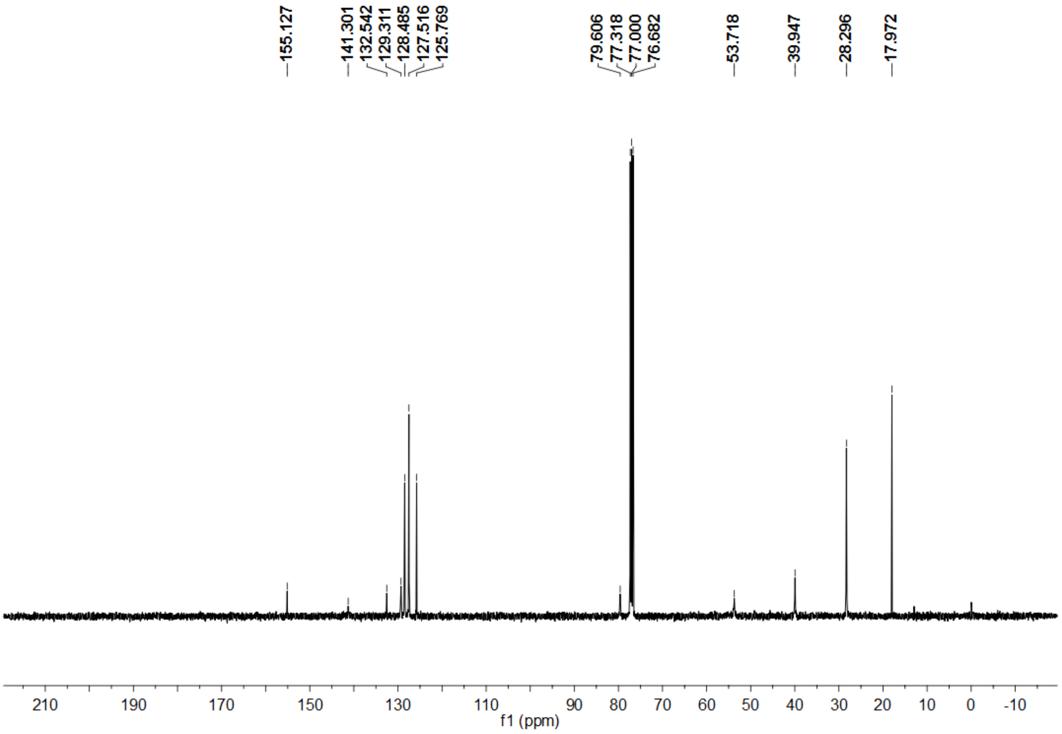

Supplementary Figure 39. ^{13}C NMR spectrum of (*S*)-**3s**

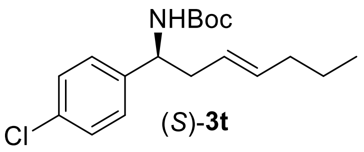

(*S*)-**3t**

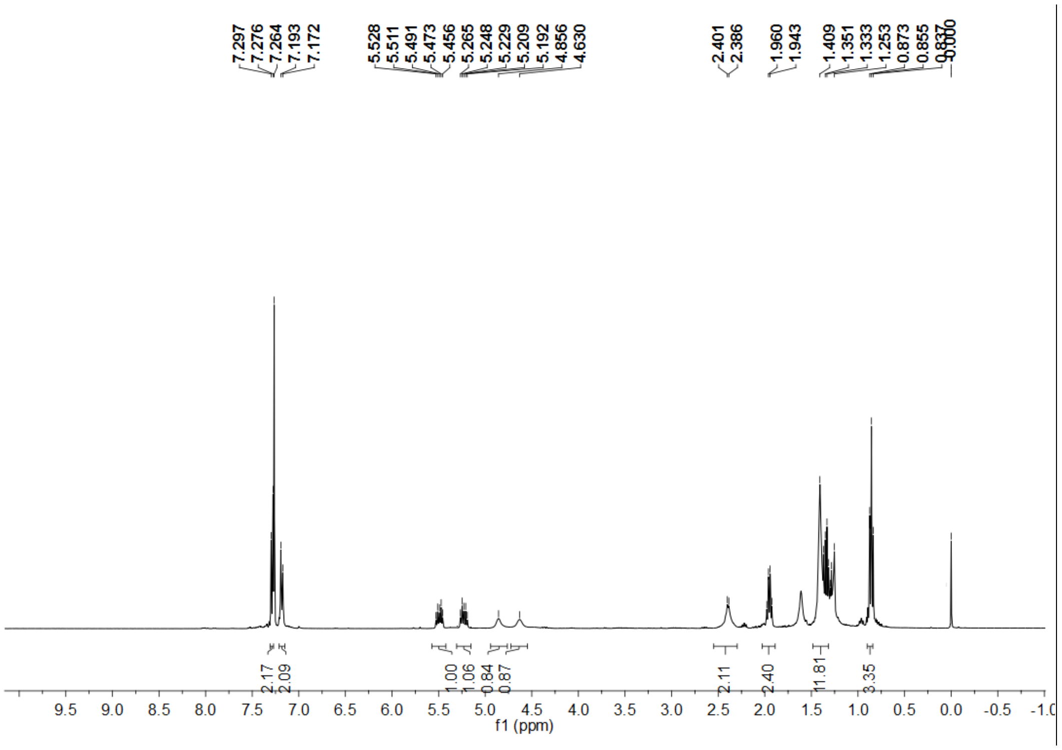

Supplementary Figure 40. ^{1}H NMR spectrum of (*S*)-**3t**

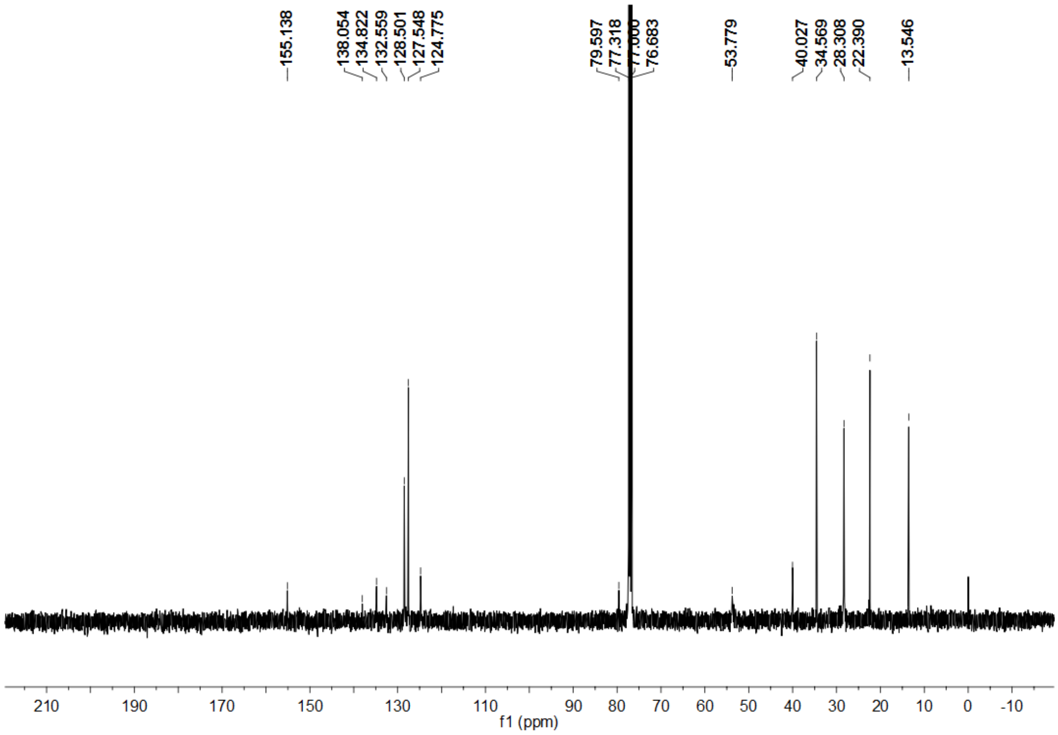

Supplementary Figure 41. ^{13}C NMR spectrum of (*S*)-**3t**

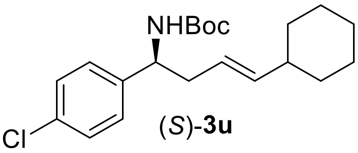

(*S*)-**3u**

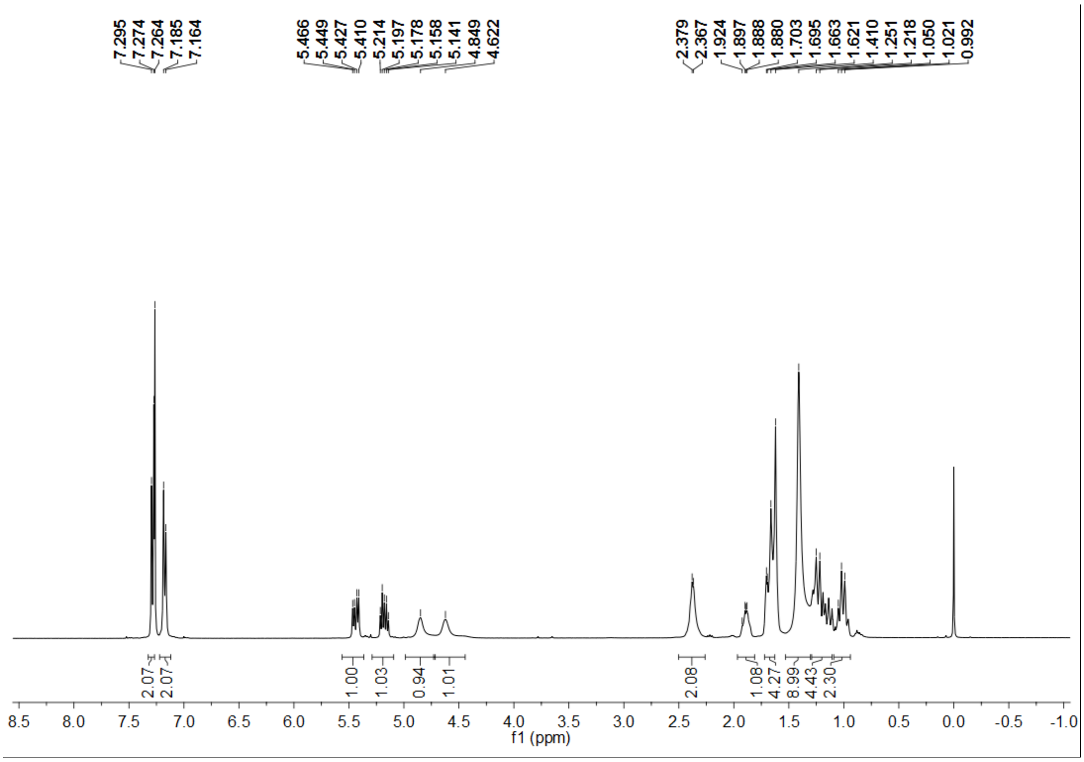

Supplementary Figure 42. ^{1}H NMR spectrum of (*S*)-**3u**

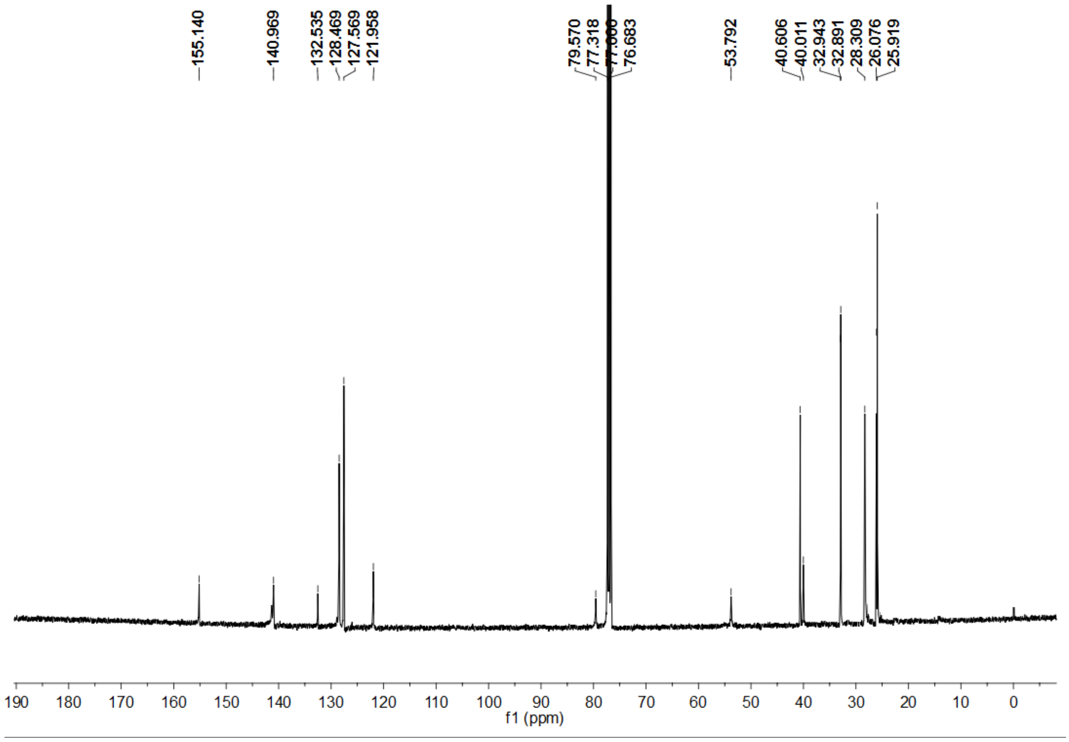

Supplementary Figure 43. ^{13}C NMR spectrum of (*S*)-**3u**

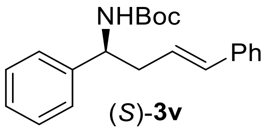

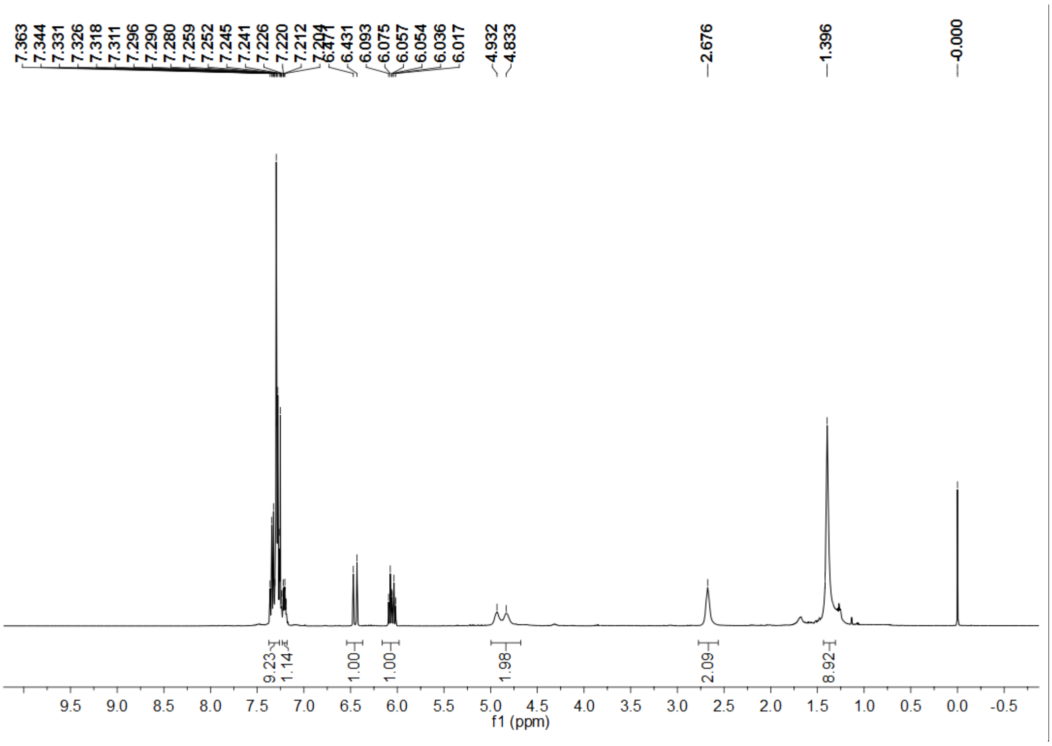

Supplementary Figure 44. ^{1}H NMR spectrum of (*S*)-**3v**

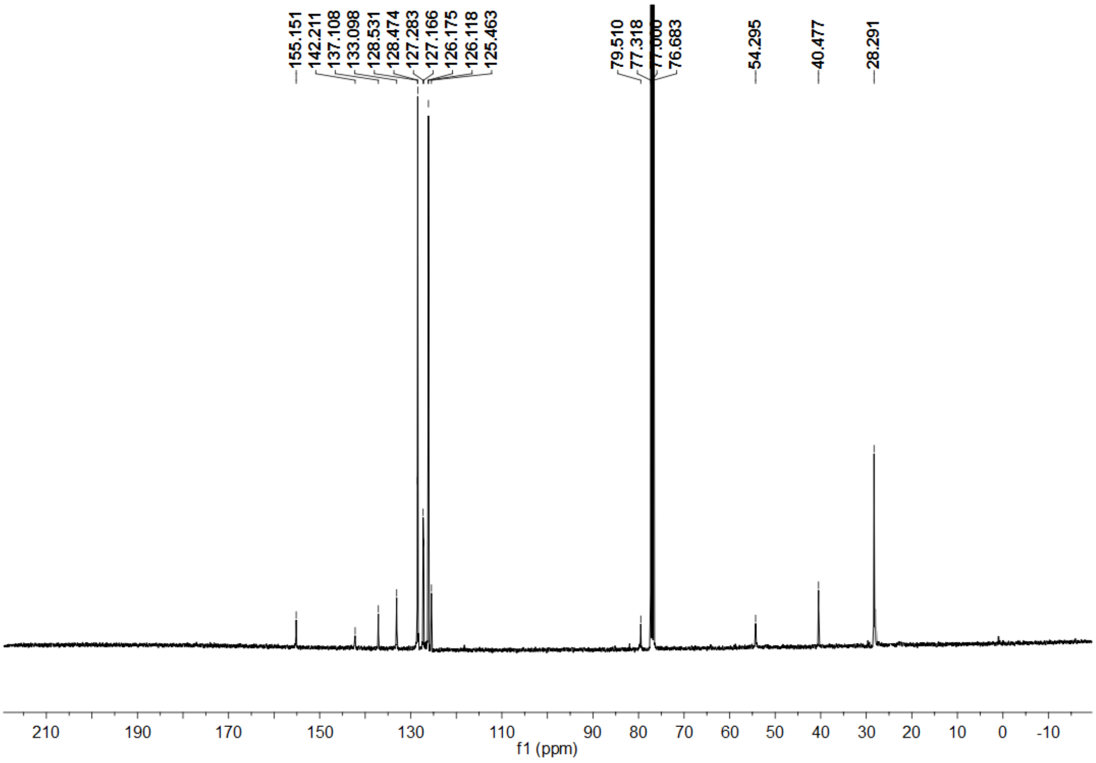

Supplementary Figure 45. ^{13}C NMR spectrum of (*S*)-**3v**

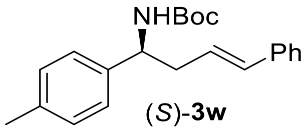

(*S*)-**3w**

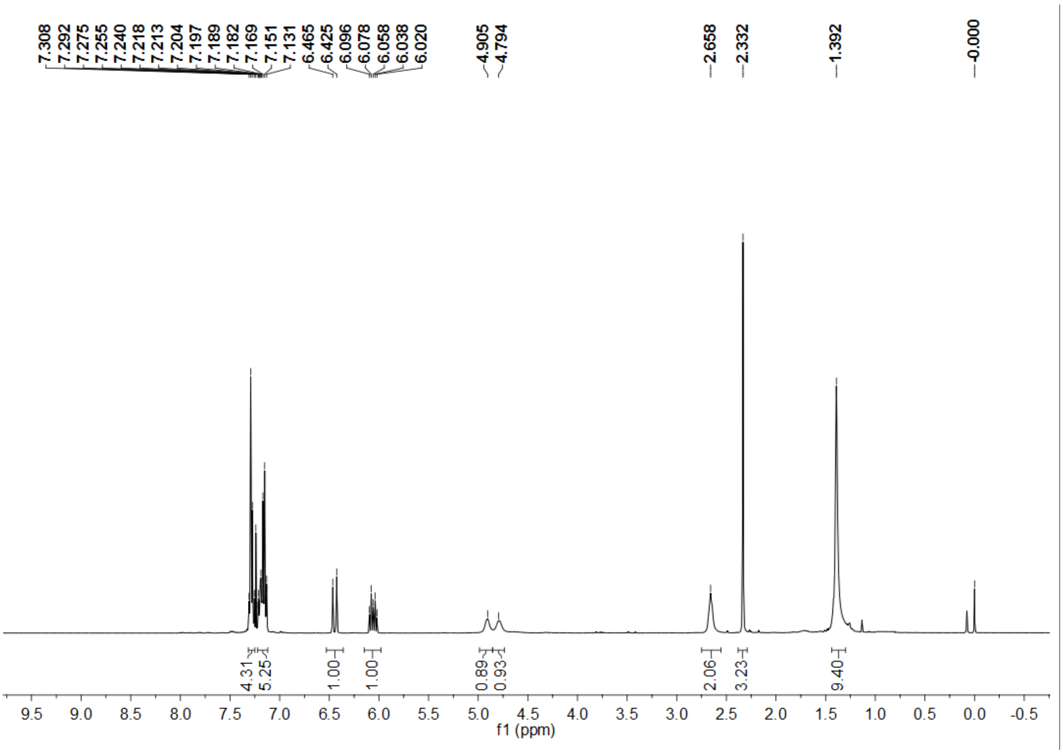

Supplementary Figure 46. ^{1}H NMR spectrum of (*S*)-**3w**

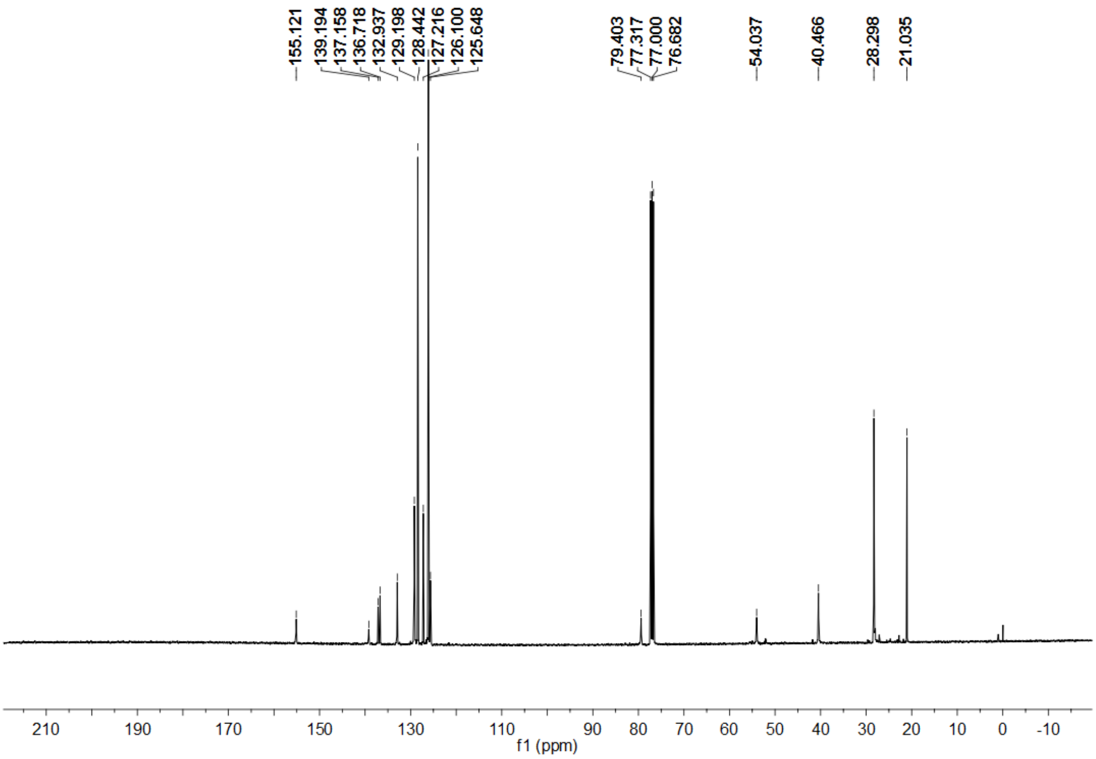

Supplementary Figure 47. ^{13}C NMR spectrum of (*S*)-**3w**

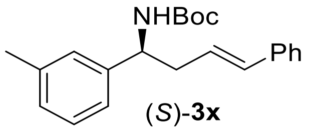

(*S*)-**3x**

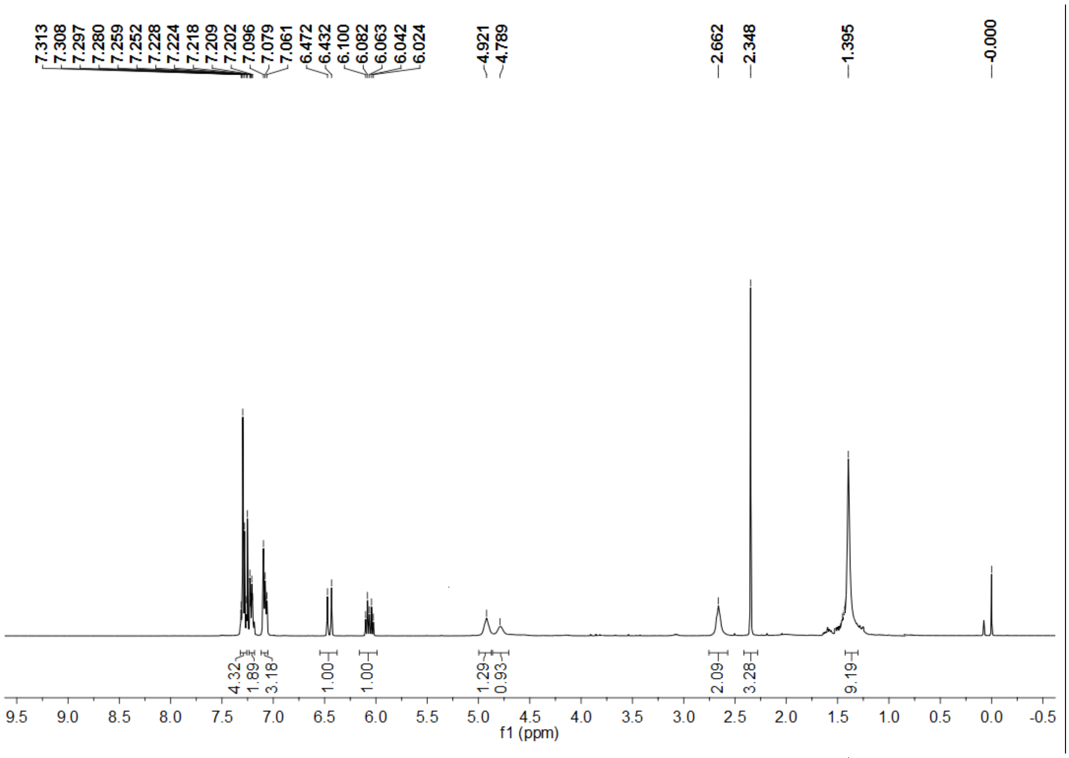

Supplementary Figure 48. ^{1}H NMR spectrum of (*S*)-**3x**

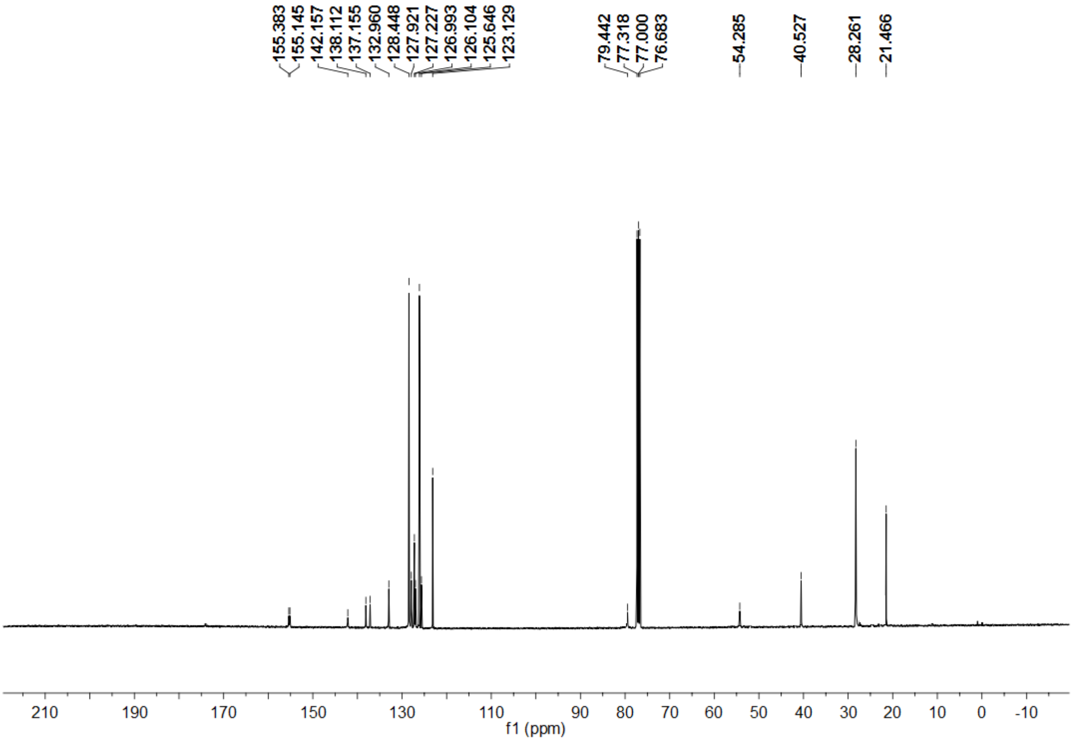

Supplementary Figure 49. ^{13}C NMR spectrum of (*S*)-**3x**

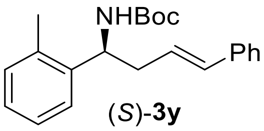

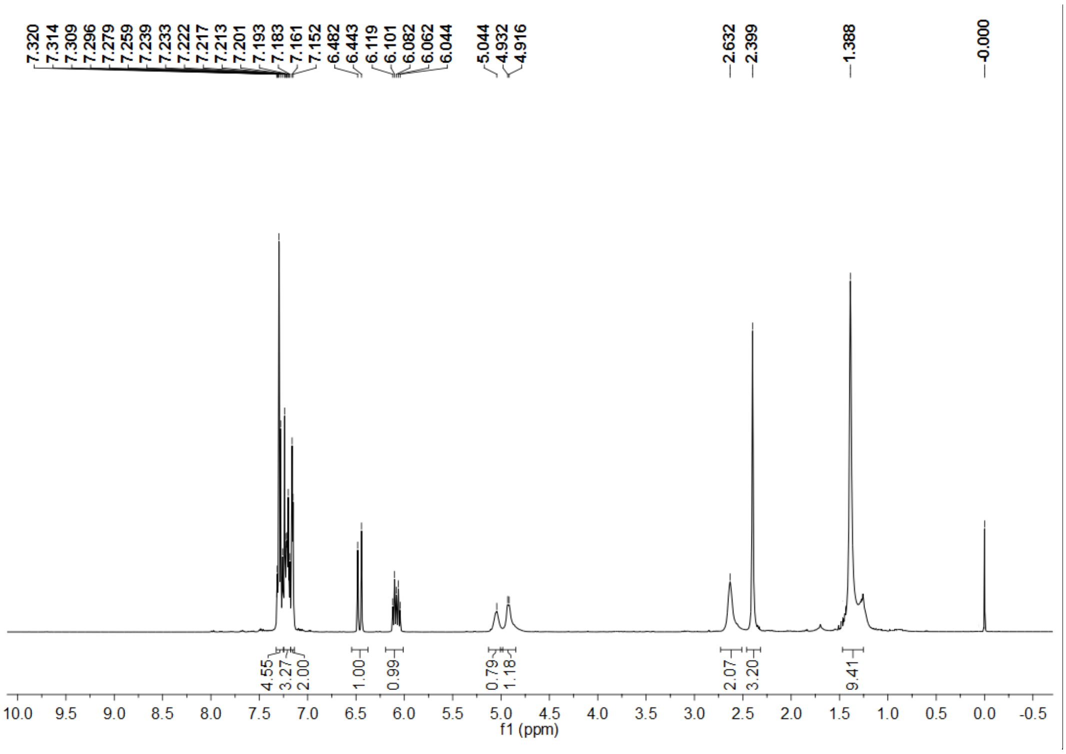

Supplementary Figure 50. ^{1}H NMR spectrum of (*S*)-**3y**

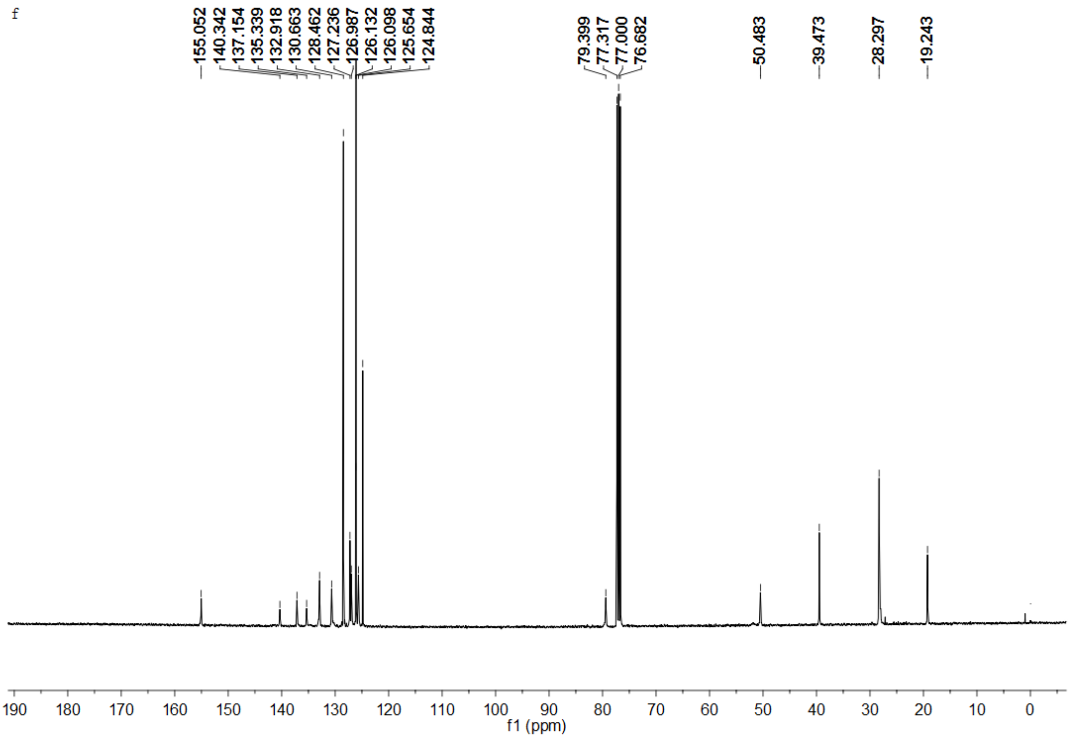

Supplementary Figure 51. ^{13}C NMR spectrum of (*S*)-**3y**

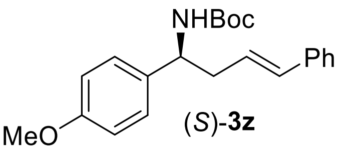

(*S*)-**3z**

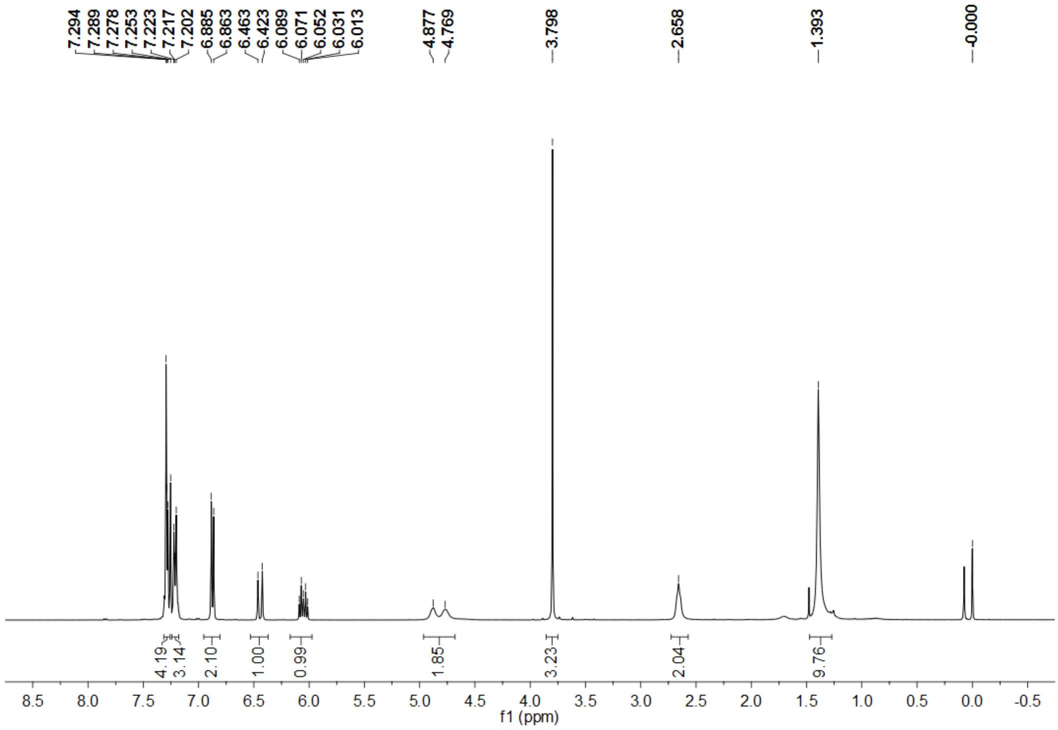

Supplementary Figure 52. ^{1}H NMR spectrum of (*S*)-**3z**

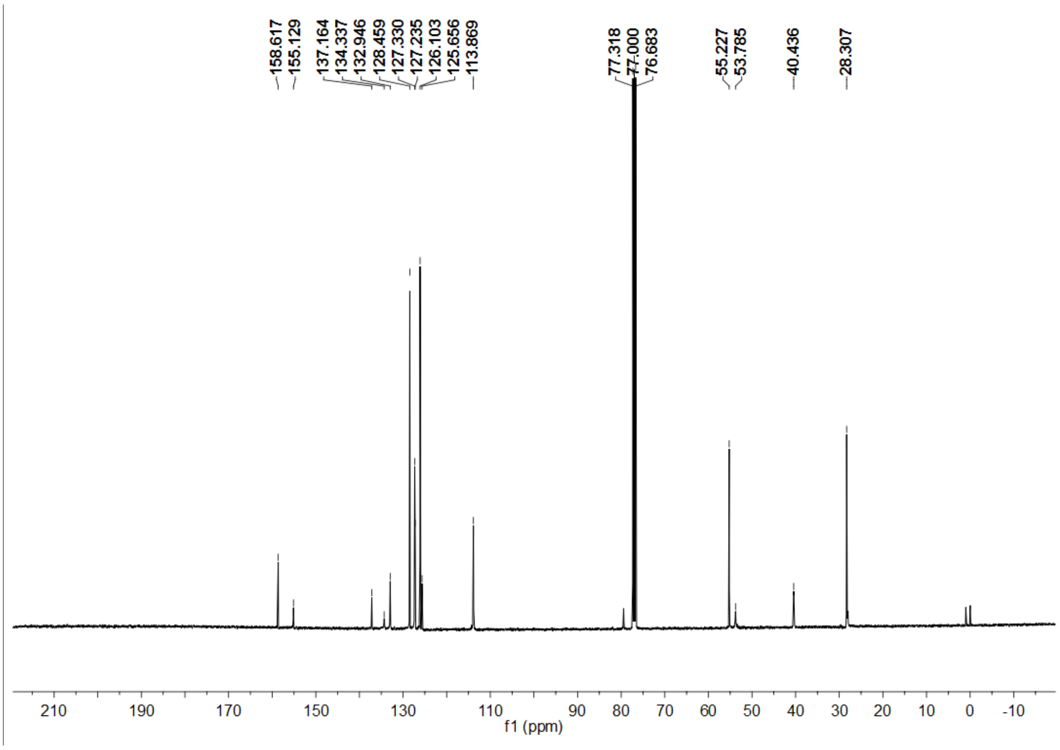

Supplementary Figure 53. ^{13}C NMR spectrum of (*S*)-**3z**

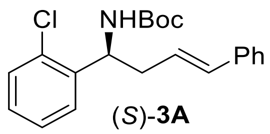

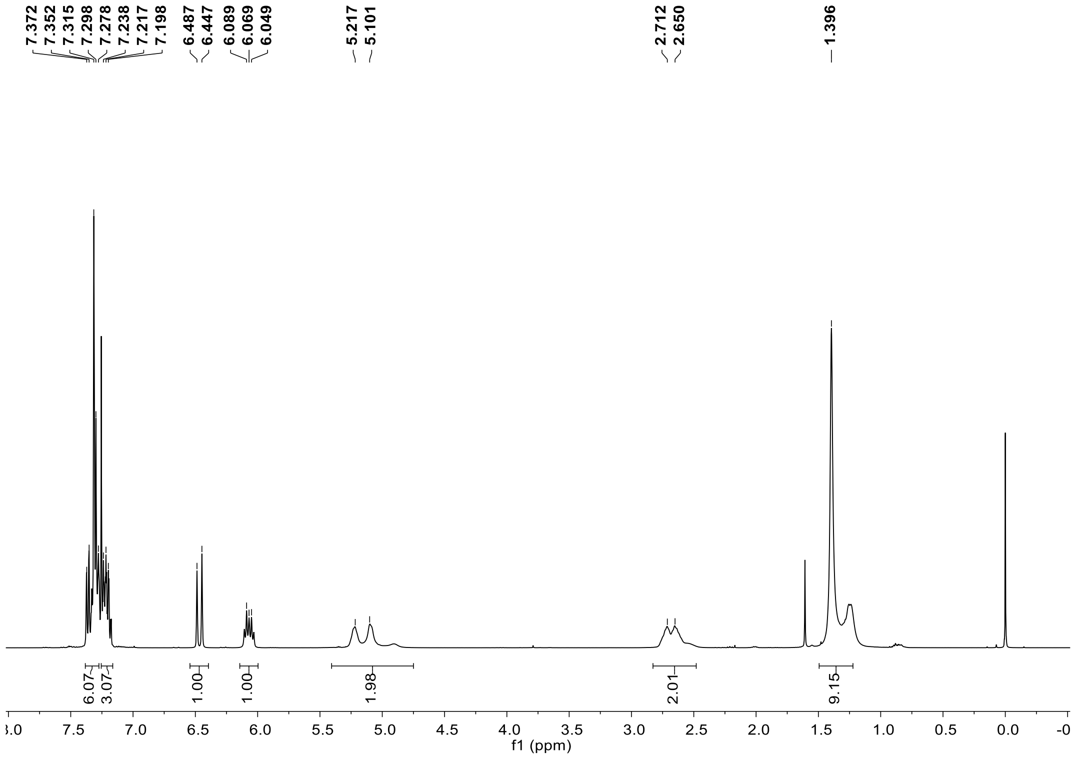

Supplementary Figure 54. ^{1}H NMR spectrum of (*S*)-**3A**

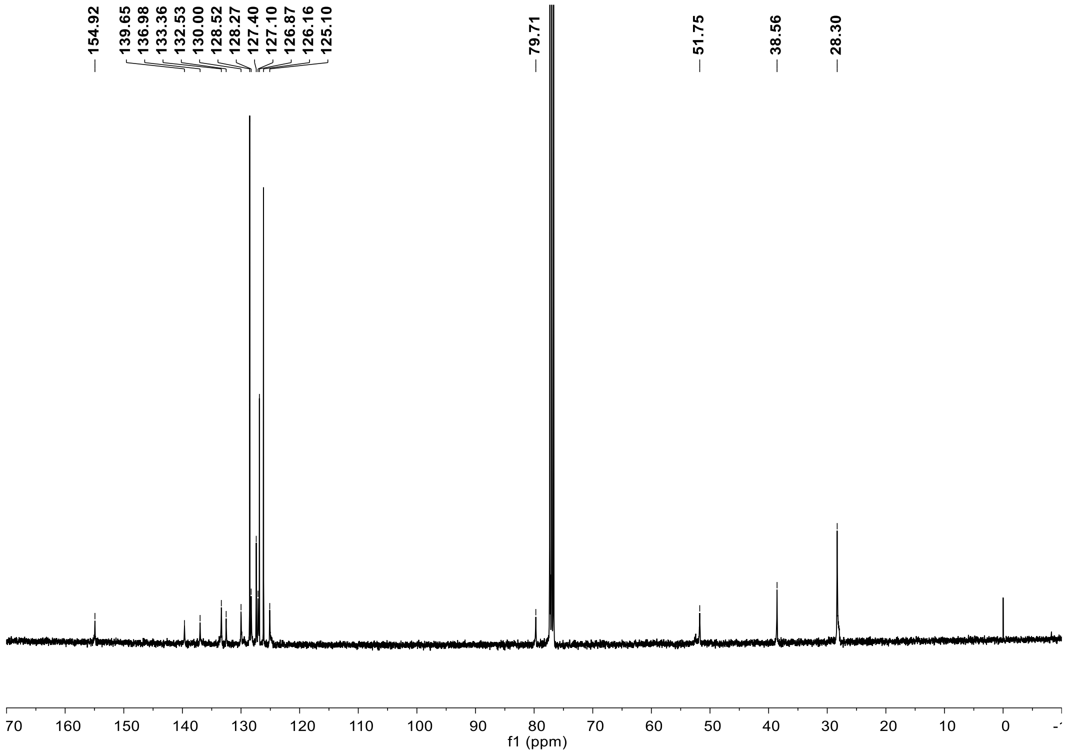

Supplementary Figure 55. ^{13}C NMR spectrum of (*S*)-**3A**

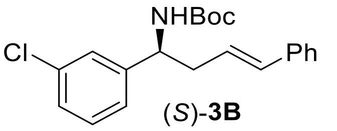

(S)-**3B**

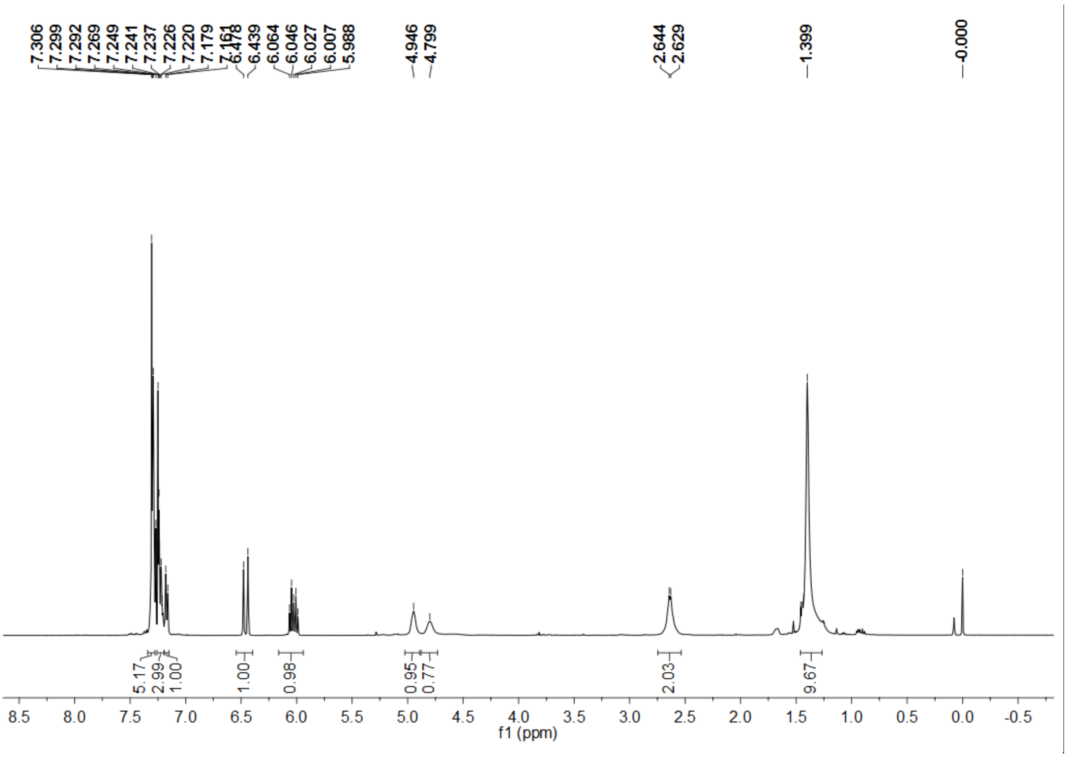

Supplementary Figure 56. ^{1}H NMR spectrum of (S)-**3B**

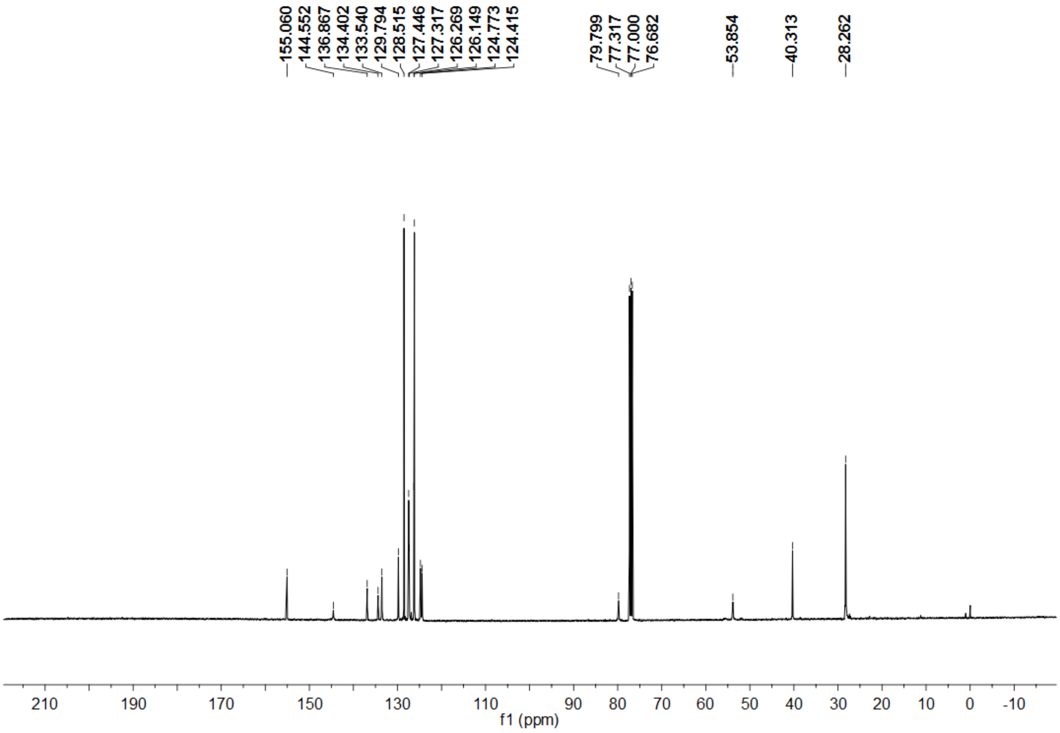

Supplementary Figure 57. ^{13}C NMR spectrum of (S)-**3B**

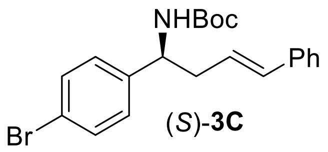

(*S*)-**3C**

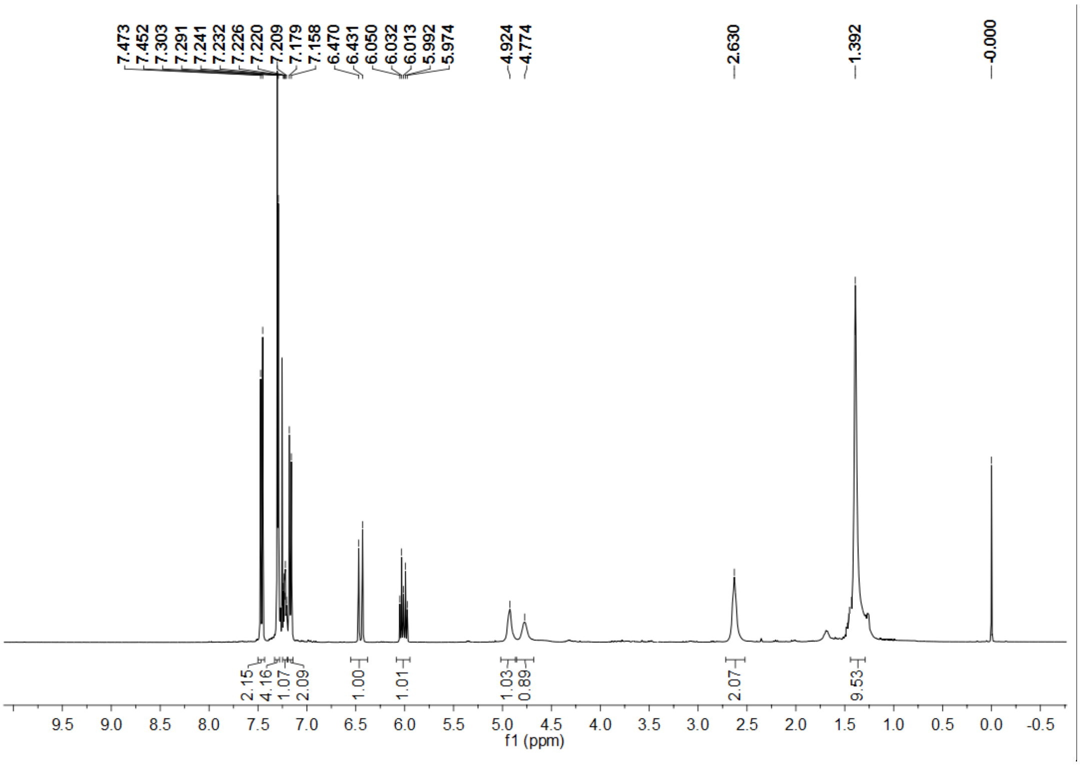

Supplementary Figure 58. ^{1}H NMR spectrum of (*S*)-**3C**

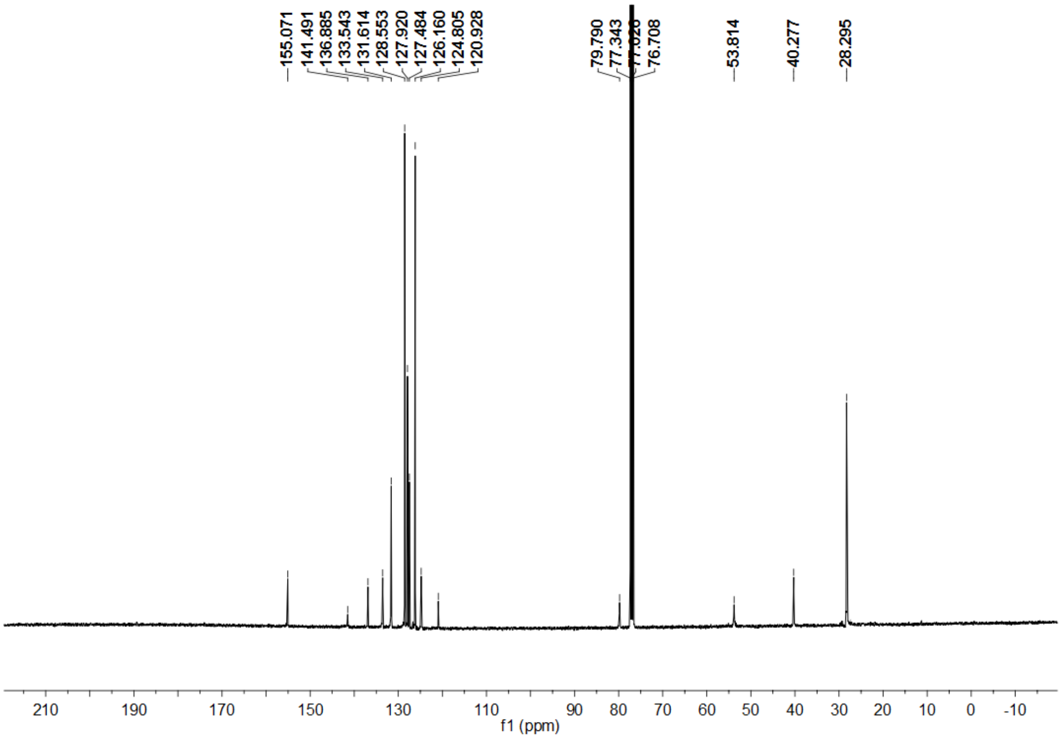

Supplementary Figure 59. ^{13}C NMR spectrum of (*S*)-**3C**

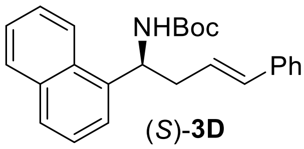

(*S*)-**3D**

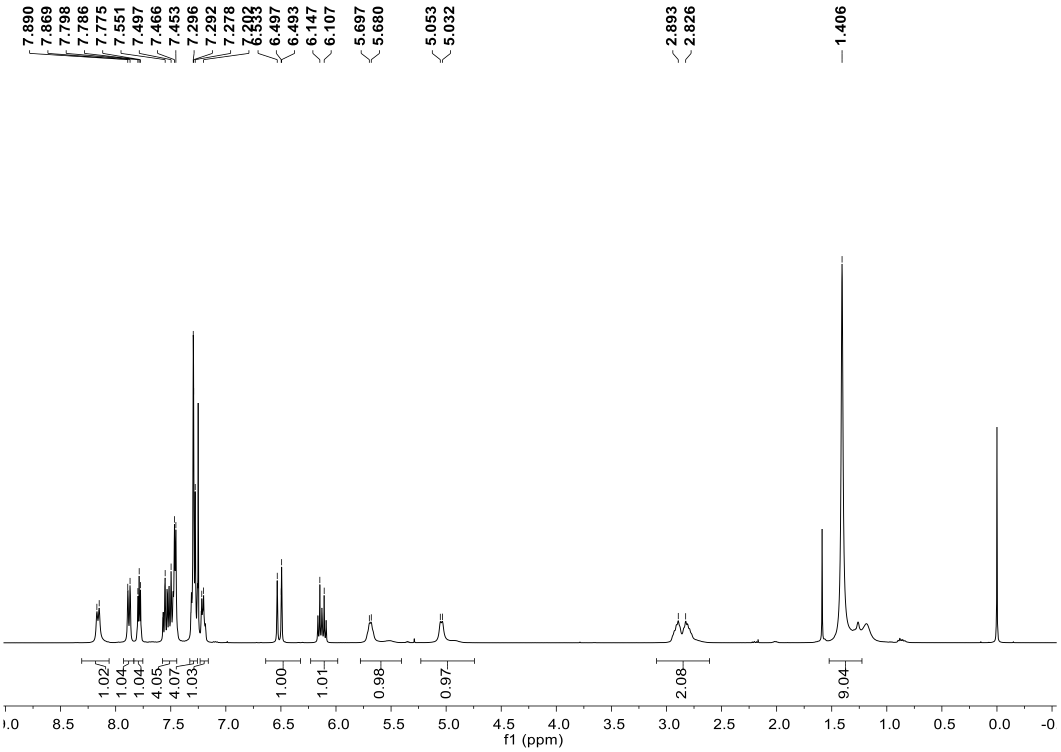

Supplementary Figure 60. ^{1}H NMR spectrum of (*S*)-**3D**

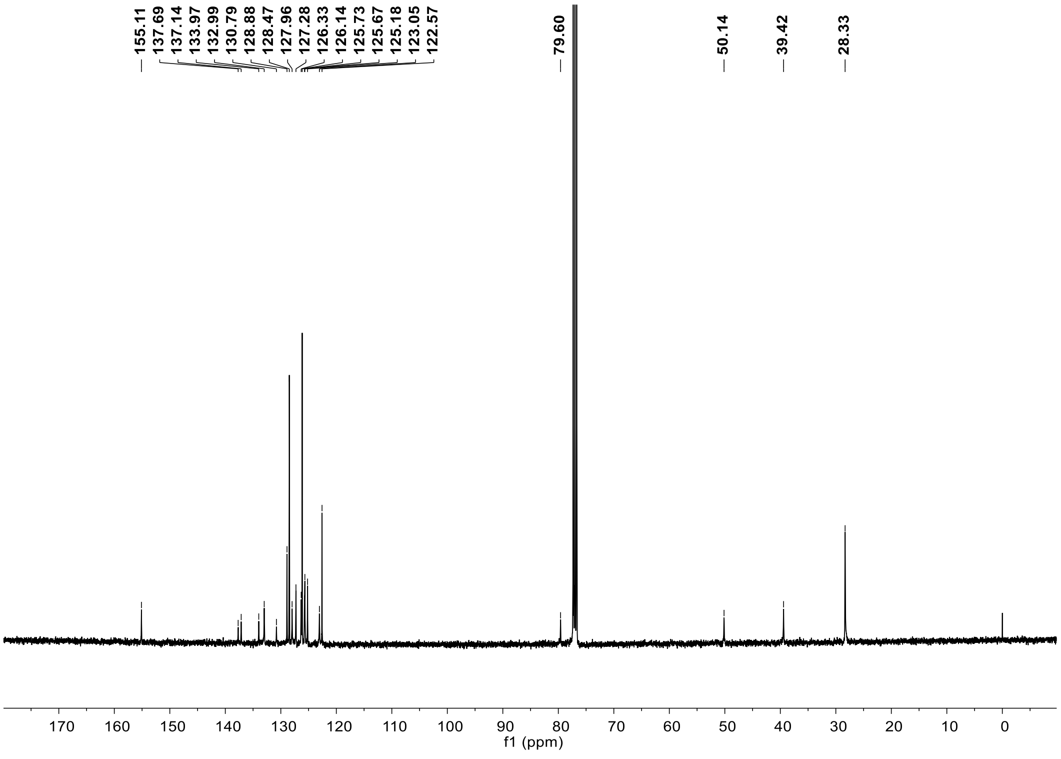

Supplementary Figure 61. ^{13}C NMR spectrum of (*S*)-**3D**

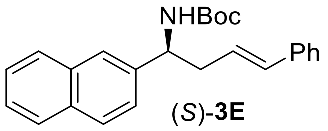

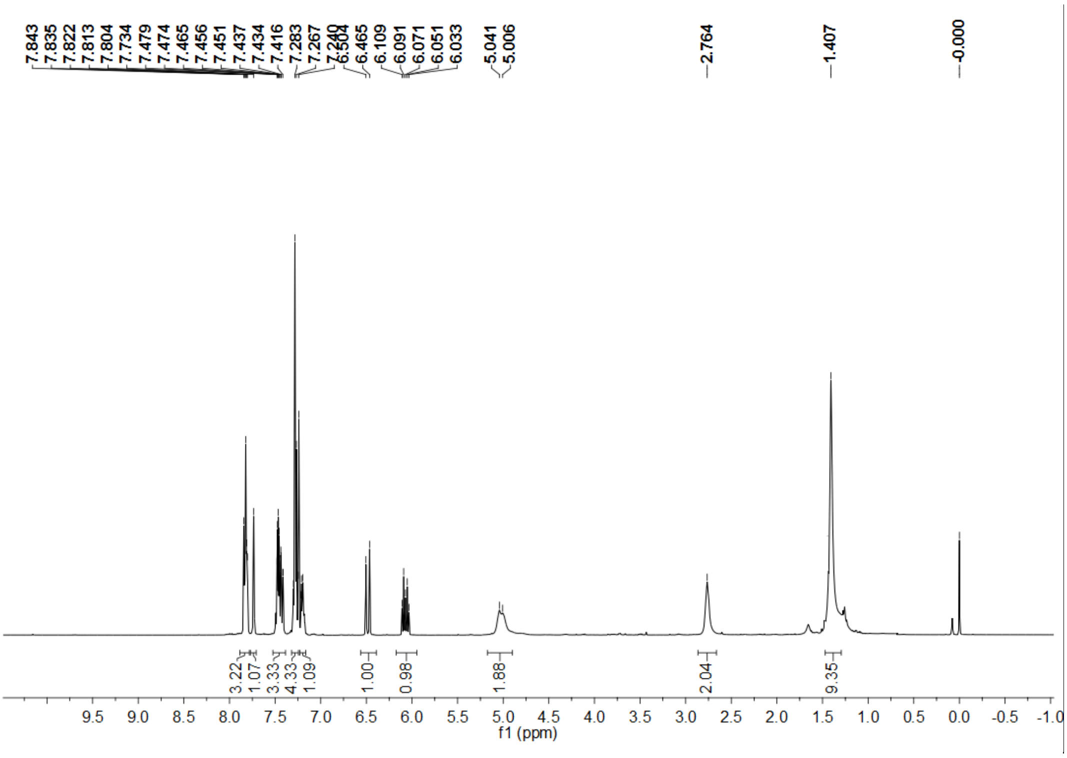

Supplementary Figure 62. ^{1}H NMR spectrum of (*S*)-**3E**

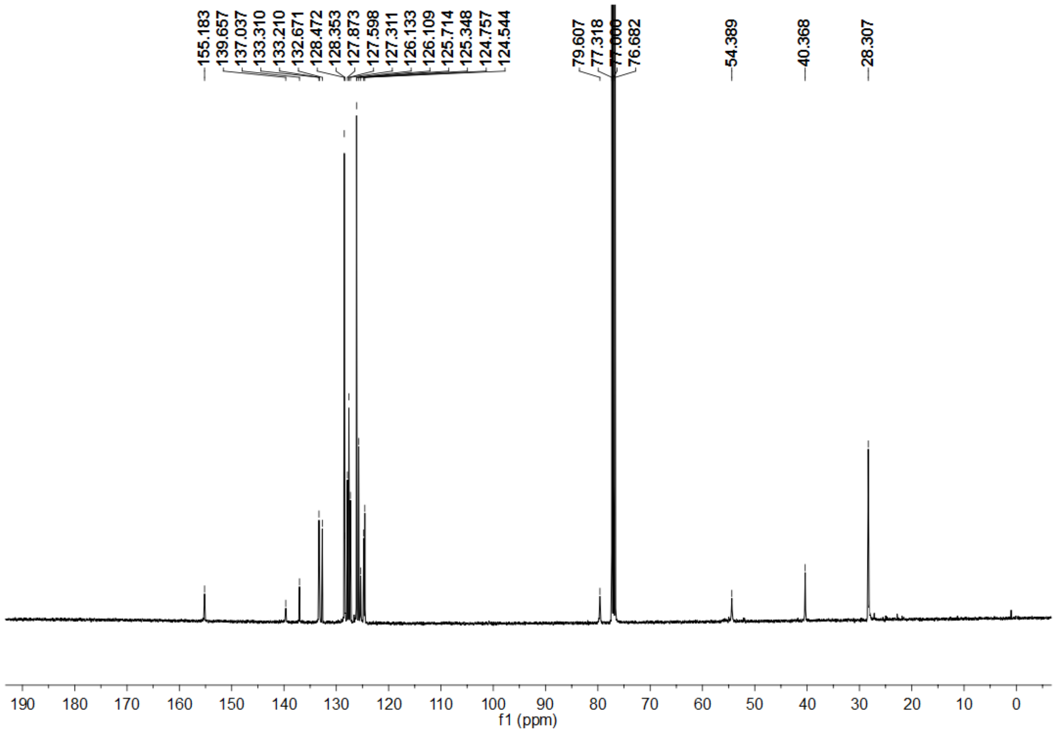

Supplementary Figure 63. ^{13}C NMR spectrum of (*S*)-**3E**

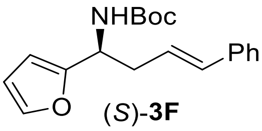

(*S*)-**3F**

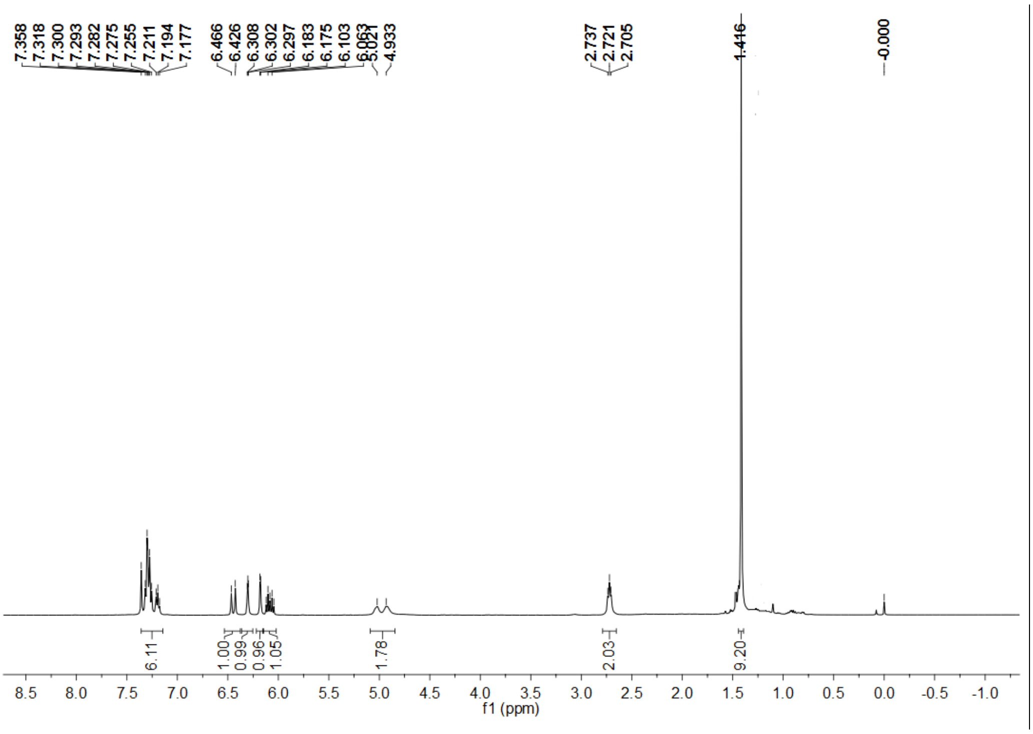

Supplementary Figure 64. ^{1}H NMR spectrum of (*S*)-**3F**

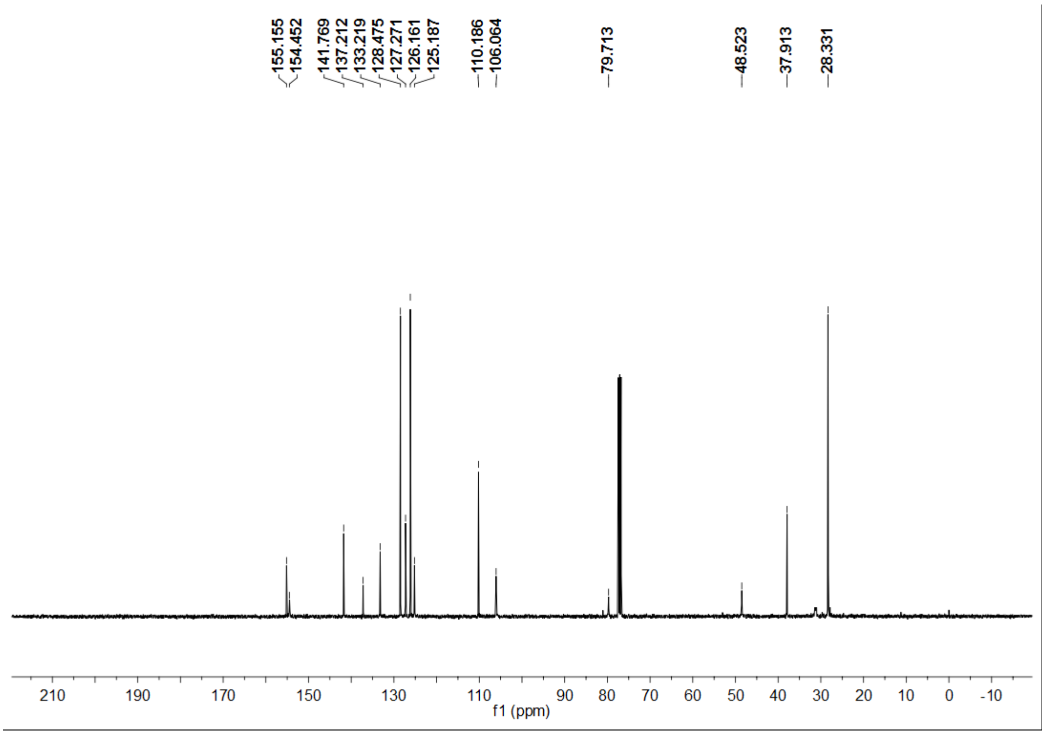

Supplementary Figure 65. ^{13}C NMR spectrum of (*S*)-**3F**

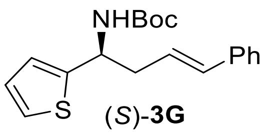

(*S*)-**3G**

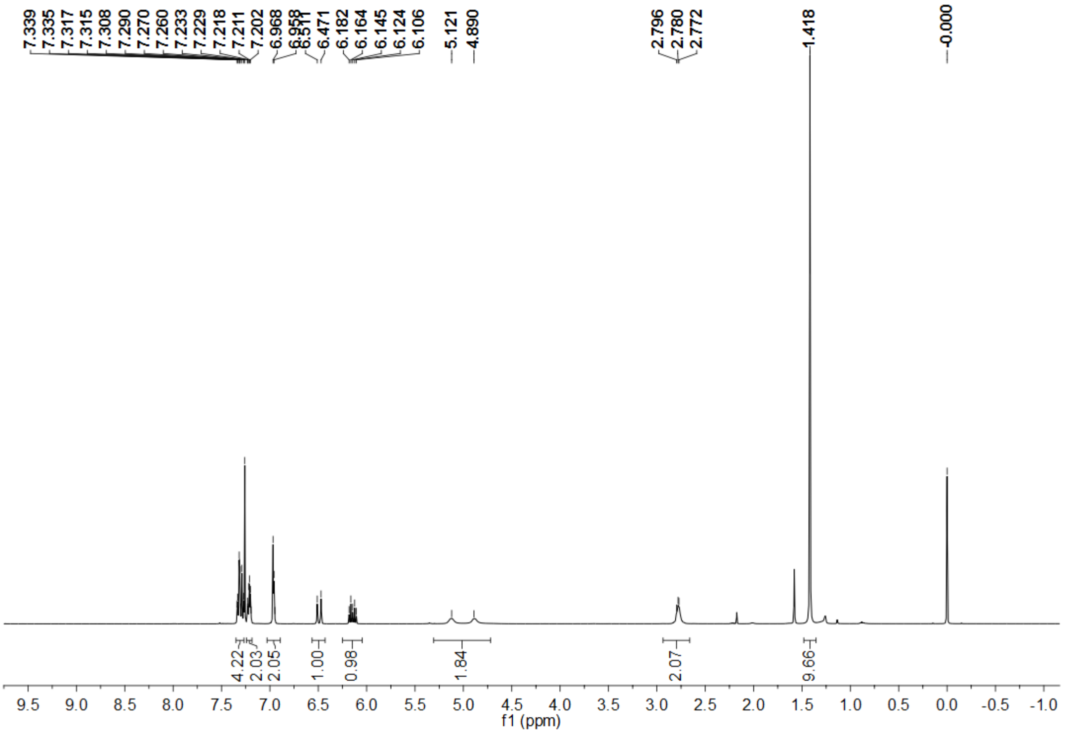

Supplementary Figure 66. ^{1}H NMR spectrum of (*S*)-**3G**

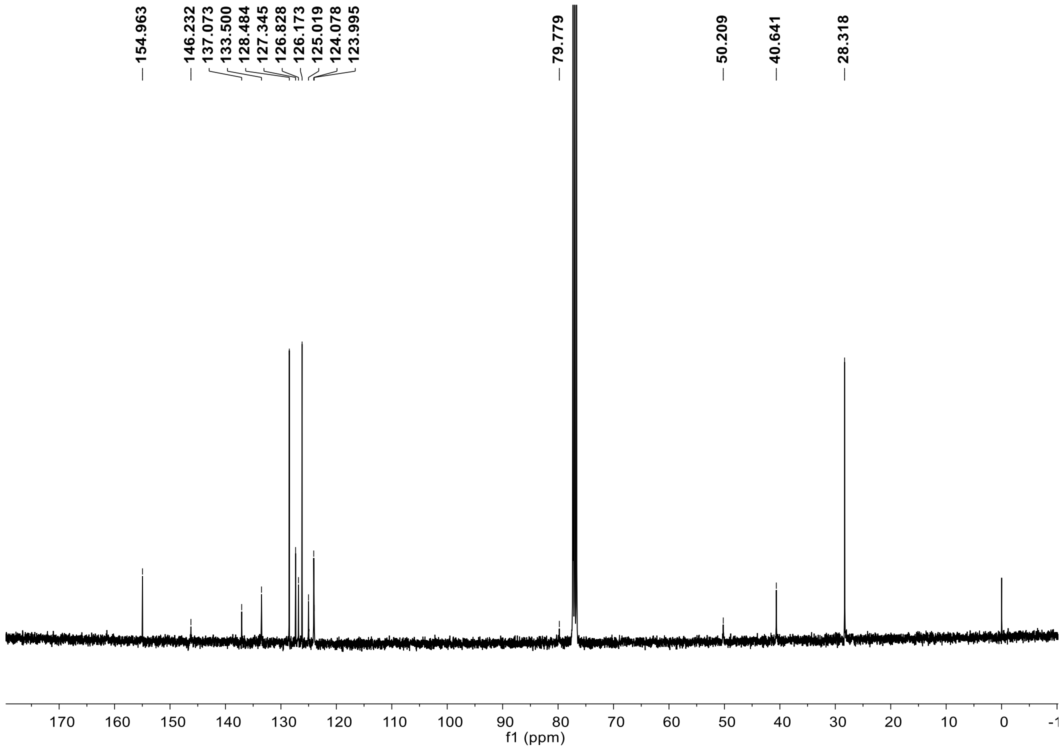

Supplementary Figure 67. ^{13}C NMR spectrum of (*S*)-**3G**

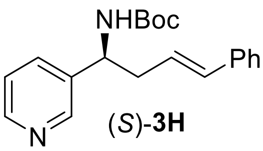

(*S*)-**3H**

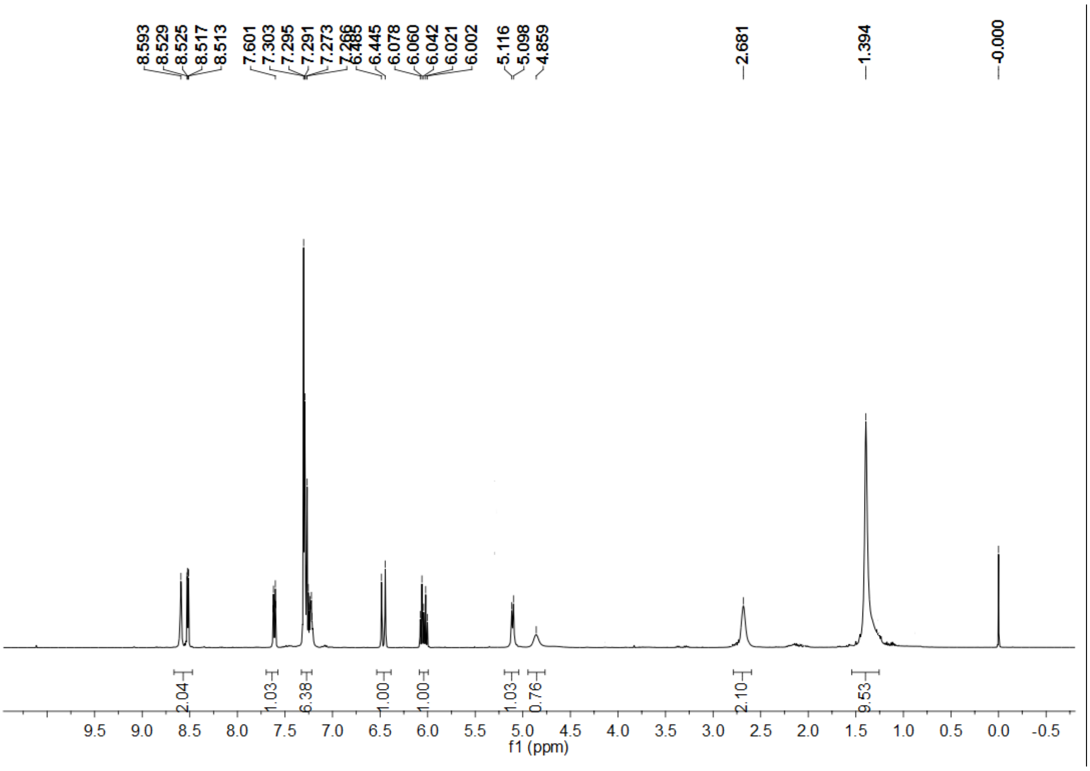

Supplementary Figure 68. ^{1}H NMR spectrum of (*S*)-**3H**

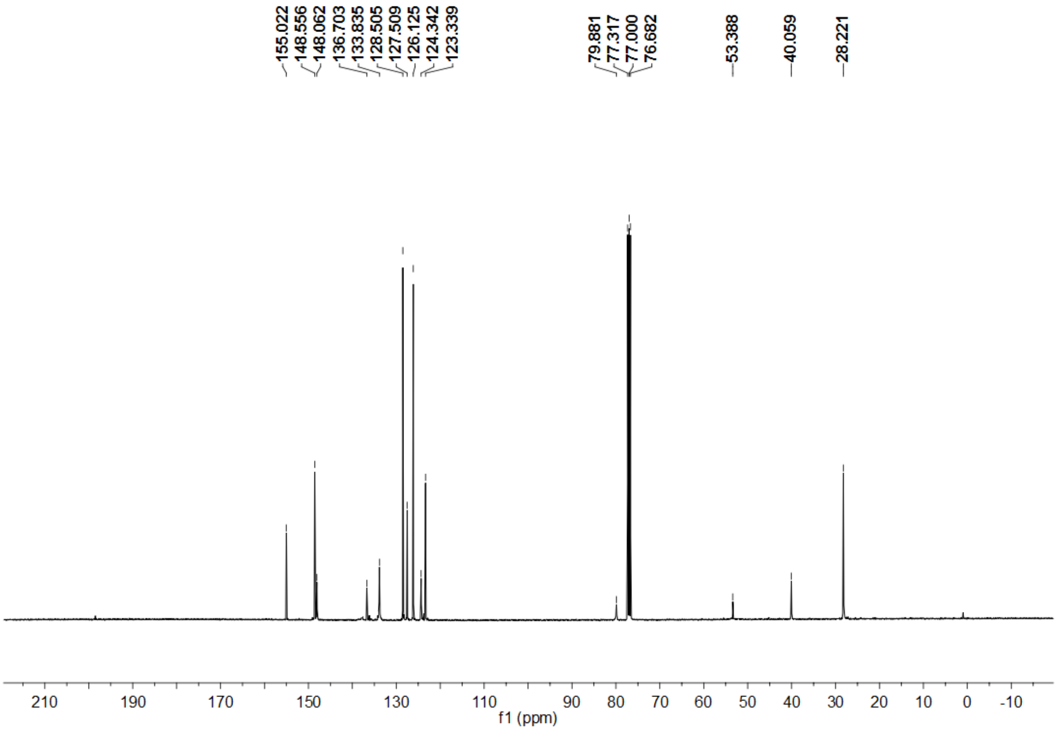

Supplementary Figure 69. ^{13}C NMR spectrum of (*S*)-**3H**

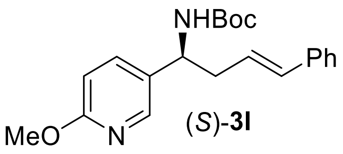

(*S*)-**3l**

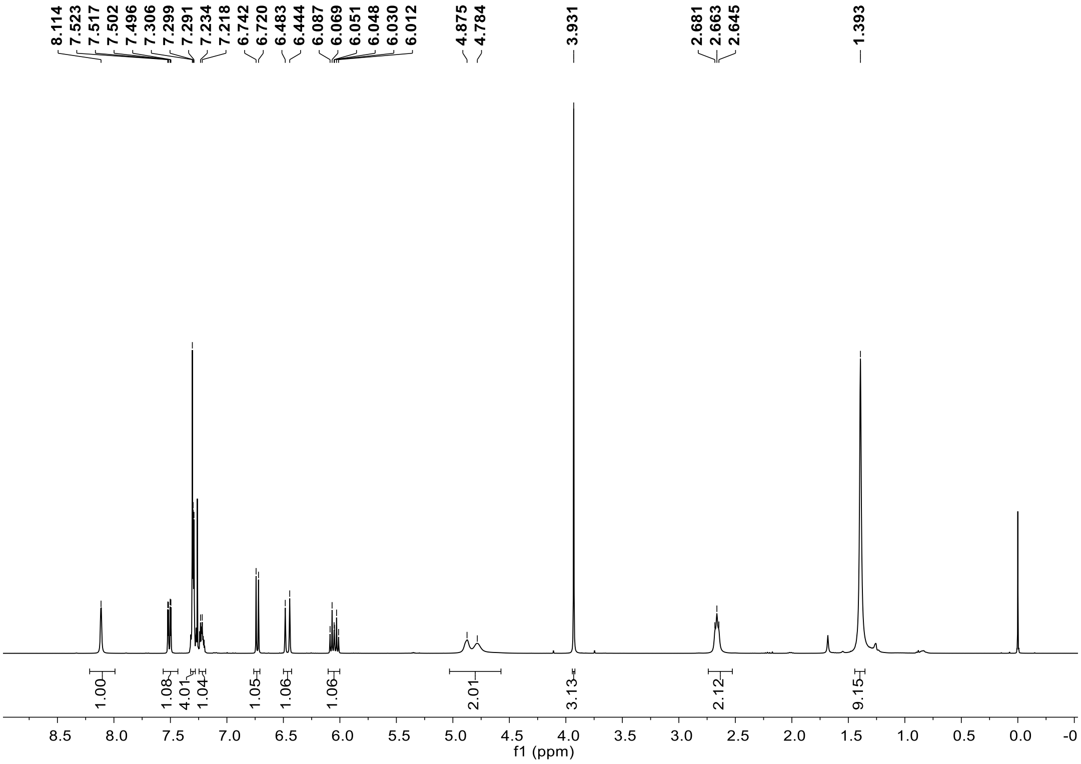

Supplementary Figure 70. ^{1}H NMR spectrum of (*S*)-**3l**

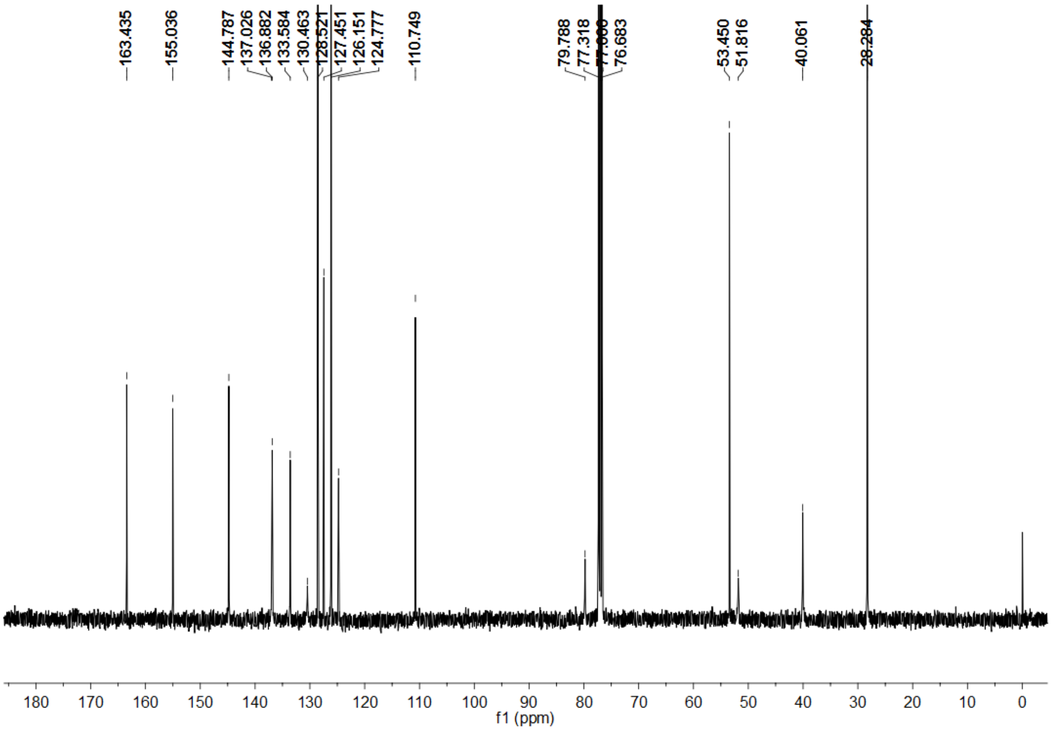

Supplementary Figure 71. ^{13}C NMR spectrum of (*S*)-**3l**

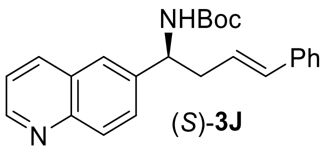

(*S*)-**3J**

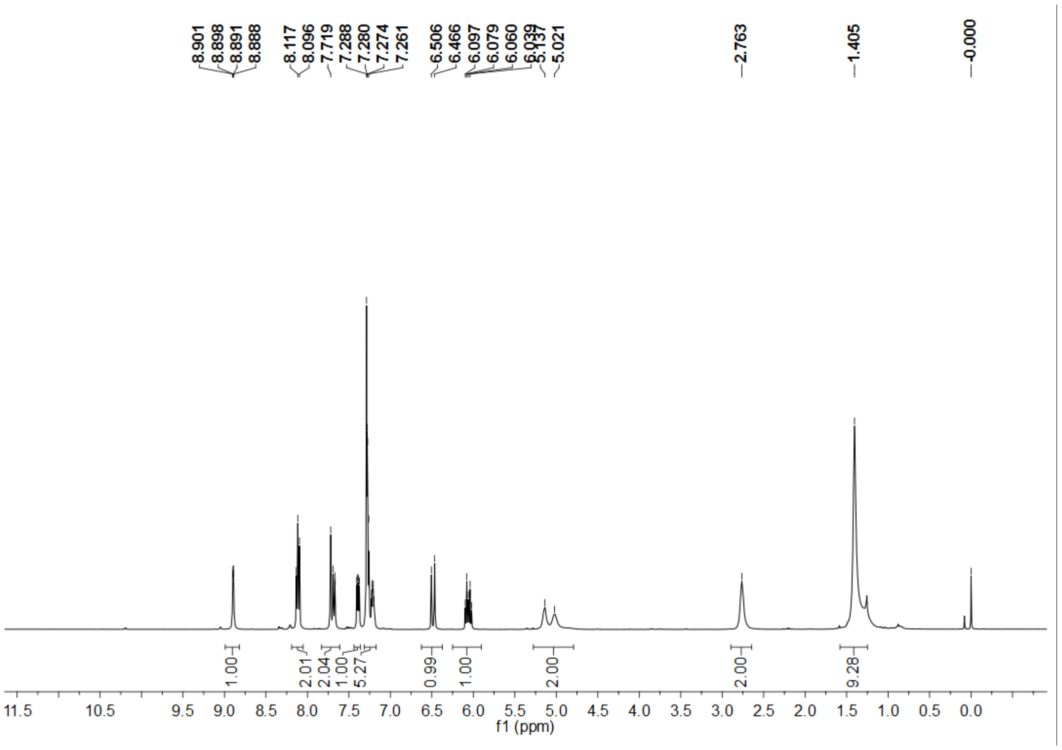

Supplementary Figure 72. ^{1}H NMR spectrum of (*S*)-**3J**

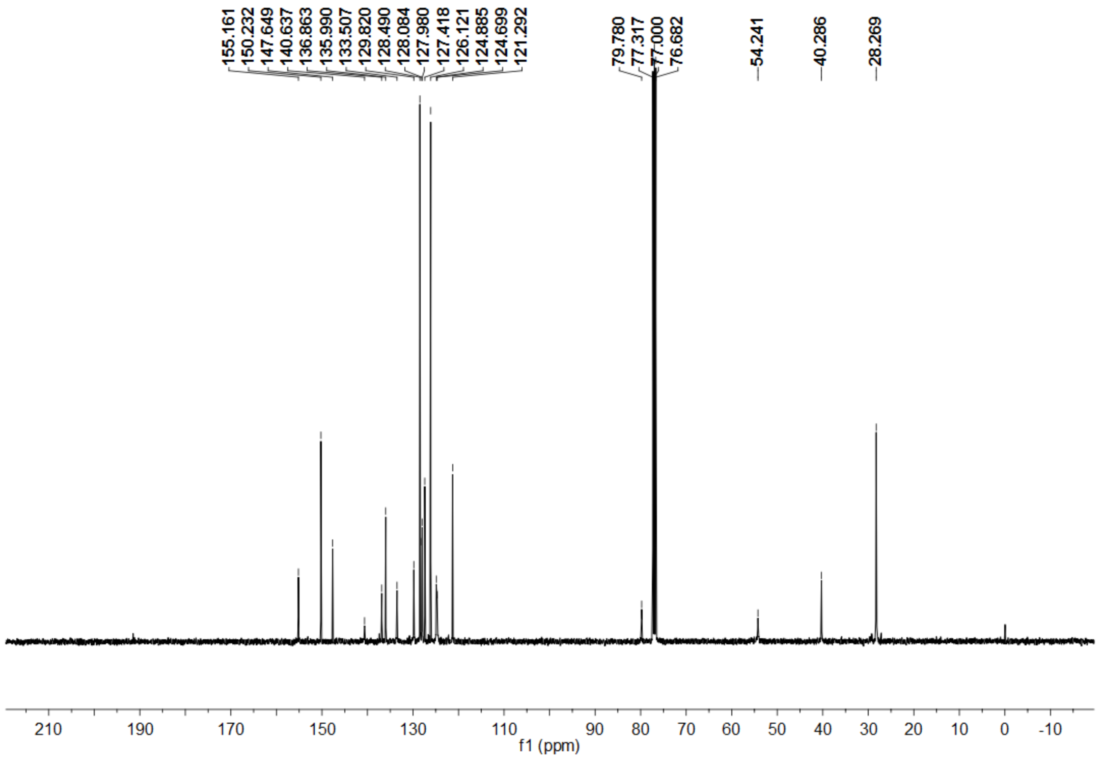

Supplementary Figure 73. ^{13}C NMR spectrum of (*S*)-**3J**

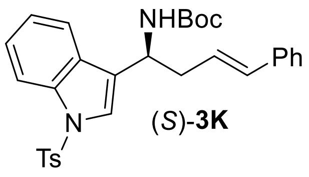

(*S*)-**3K**

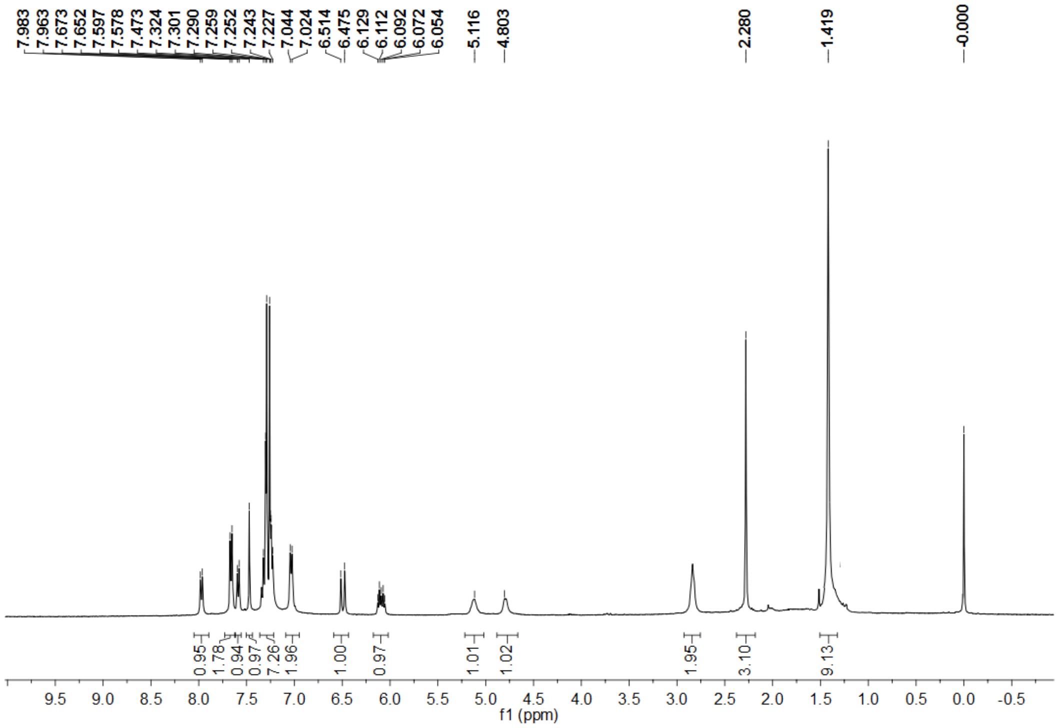

Supplementary Figure 74. ^{1}H NMR spectrum of (*S*)-**3K**

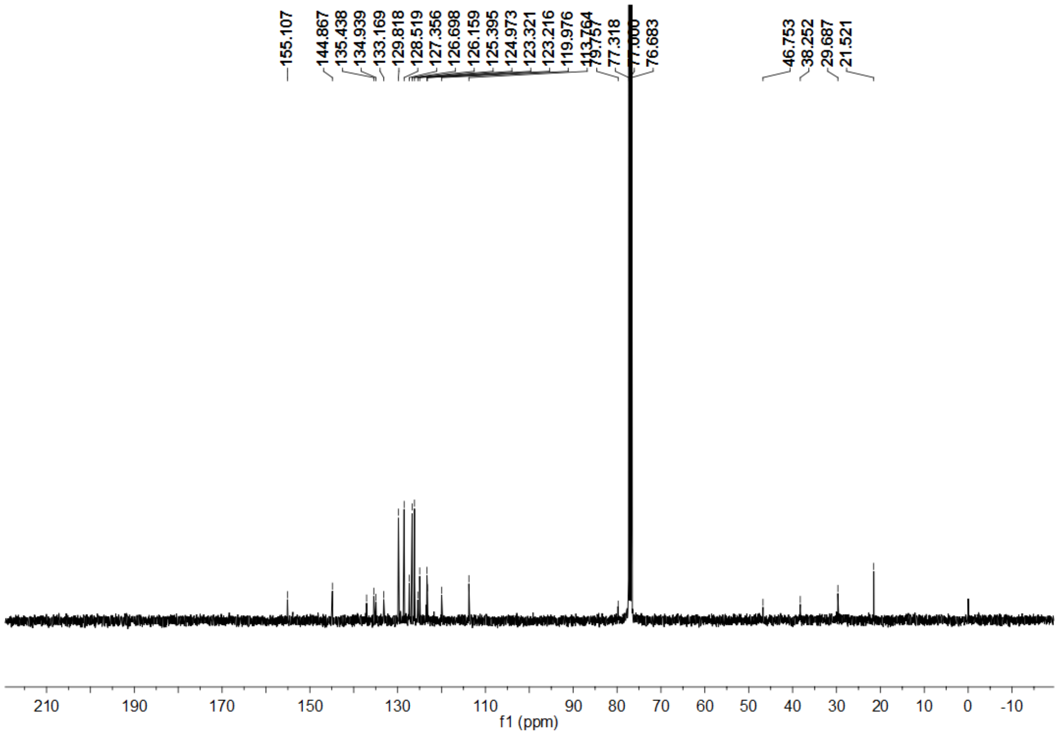

Supplementary Figure 75. ^{13}C NMR spectrum of (*S*)-**3K**

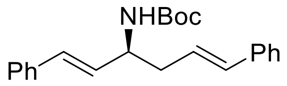

(*S*)-**3L**

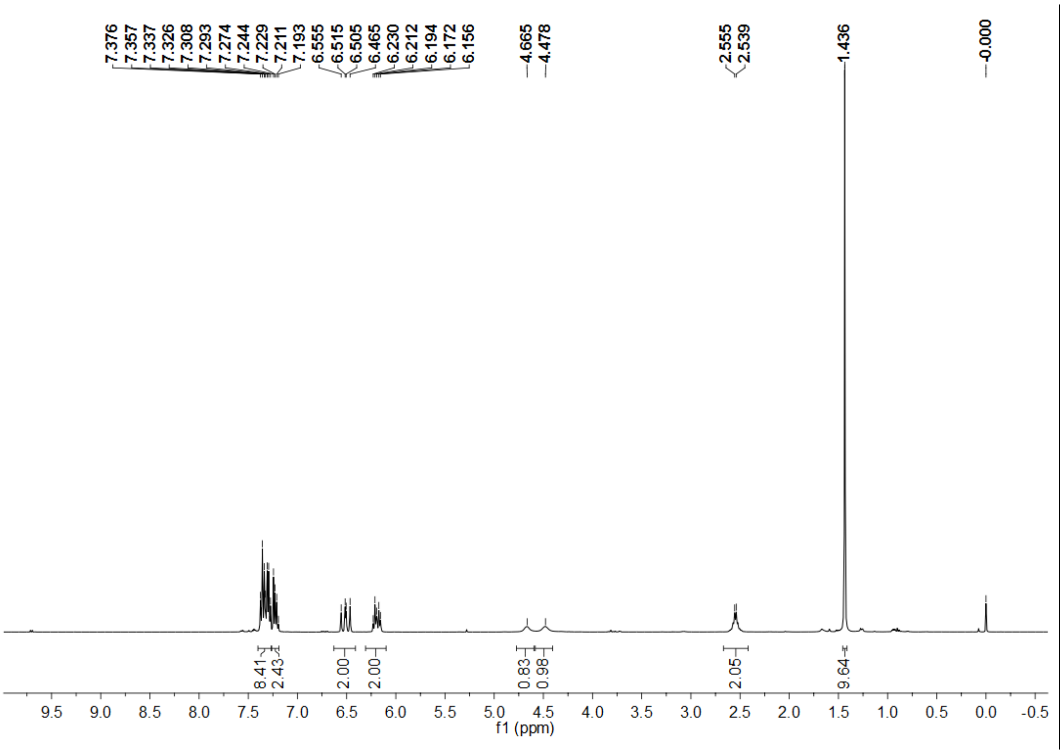

Supplementary Figure 76. ^{1}H NMR spectrum of (*S*)-**3L**

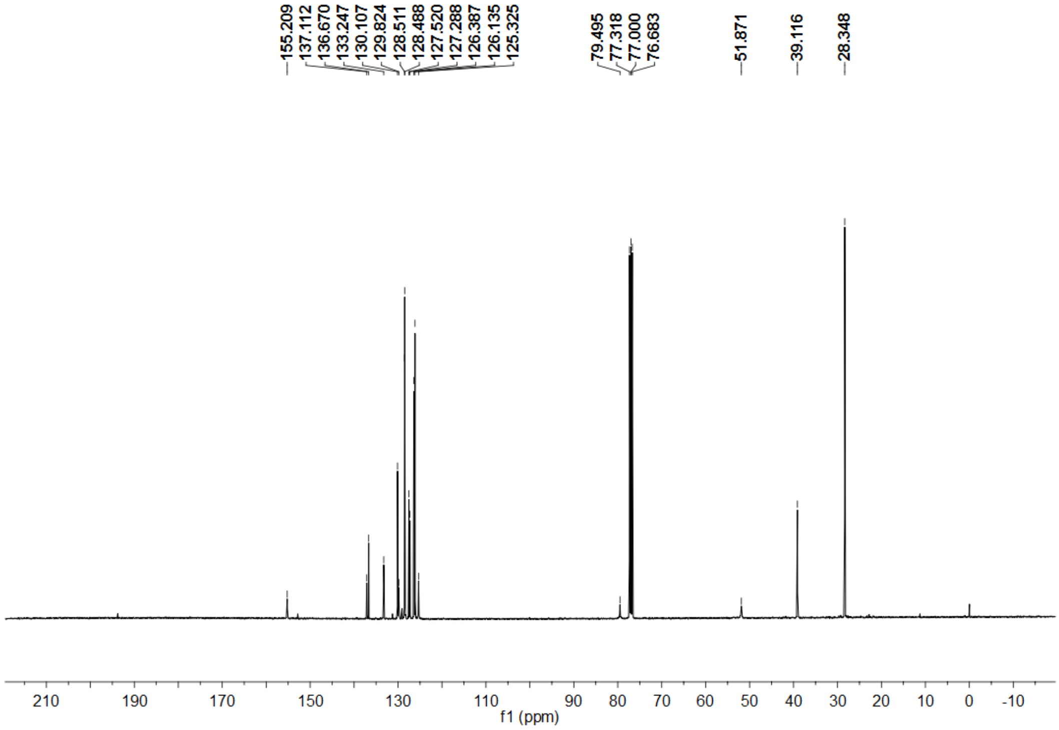

Supplementary Figure 77. ^{13}C NMR spectrum of (*S*)-**3L**

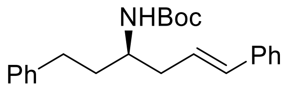

(*R*)-**3M**

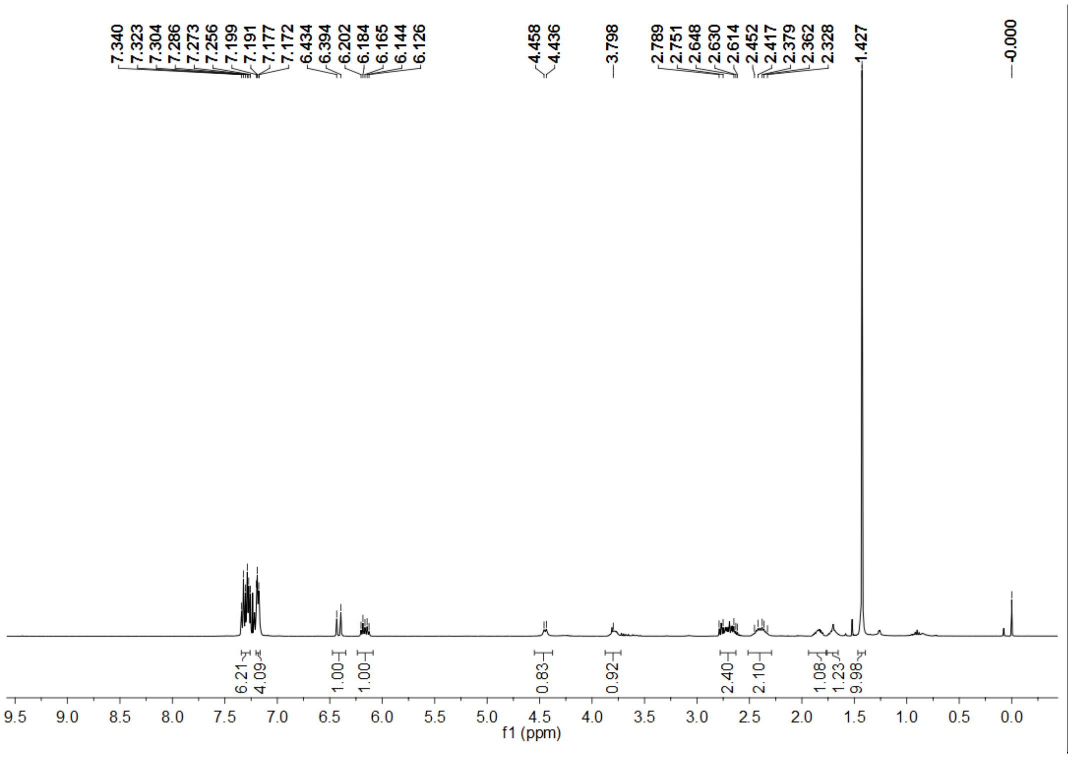

Supplementary Figure 78. ^{1}H NMR spectrum of (*R*)-**3M**

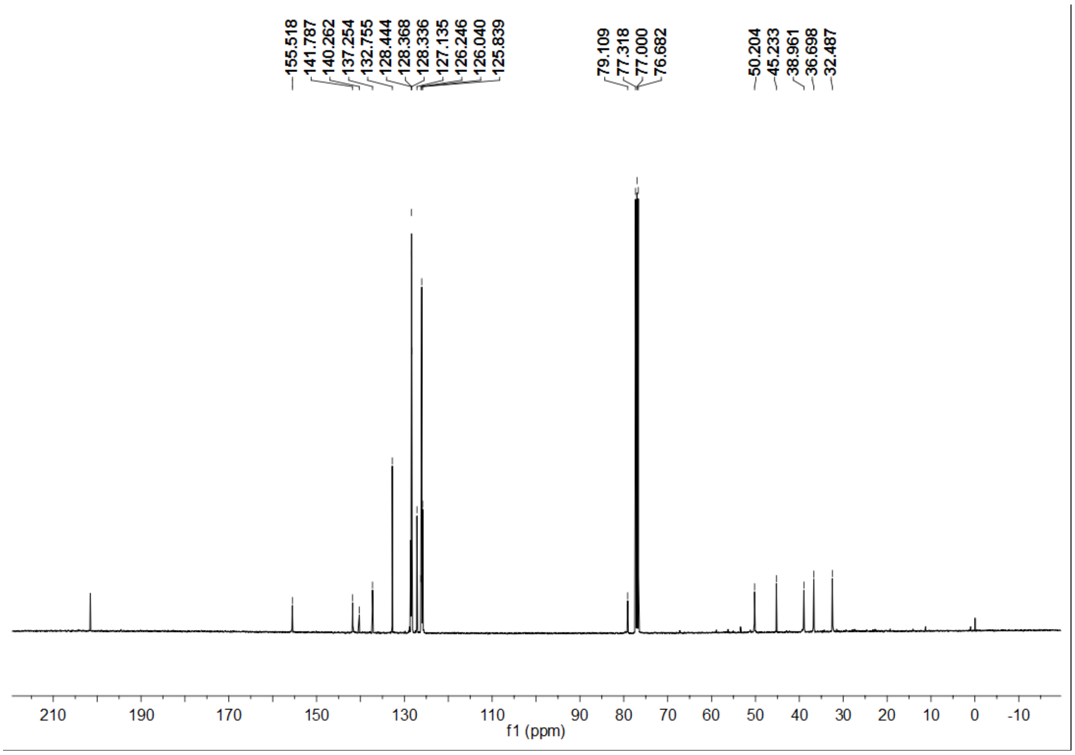

Supplementary Figure 79. ^{13}C NMR spectrum of (*R*)-**3M**

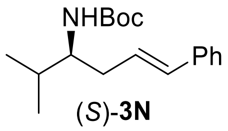

(*S*)-**3N**

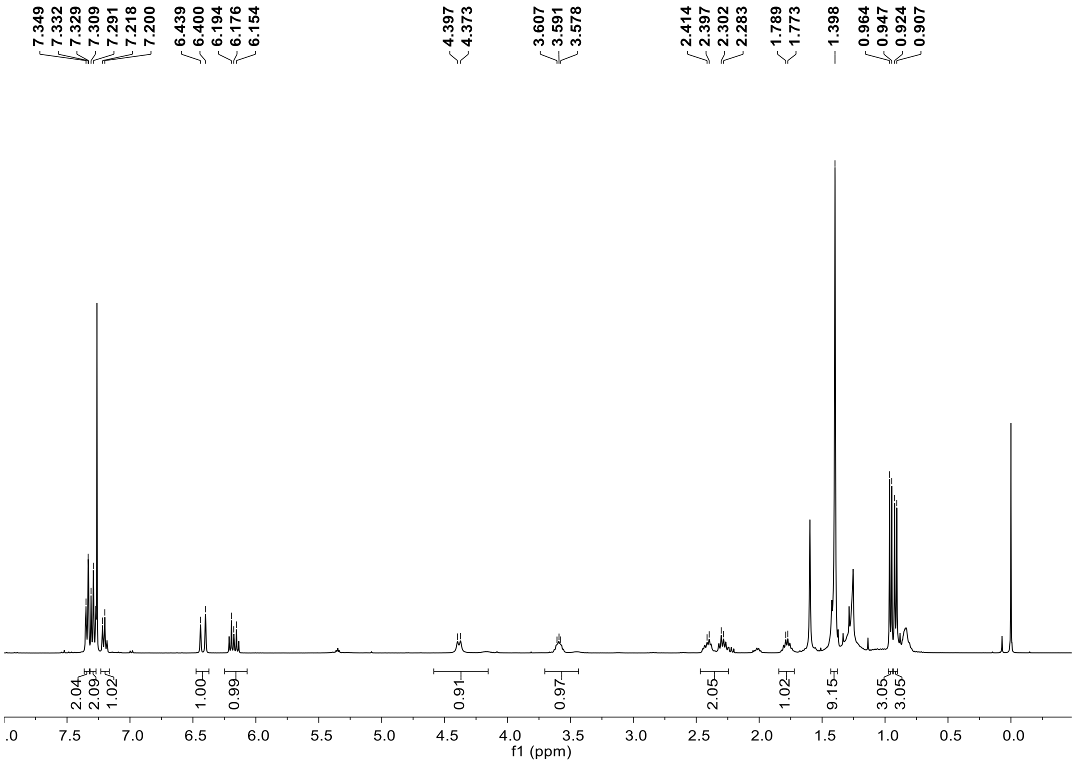

Supplementary Figure 80. ^{1}H NMR spectrum of (*S*)-**3N**

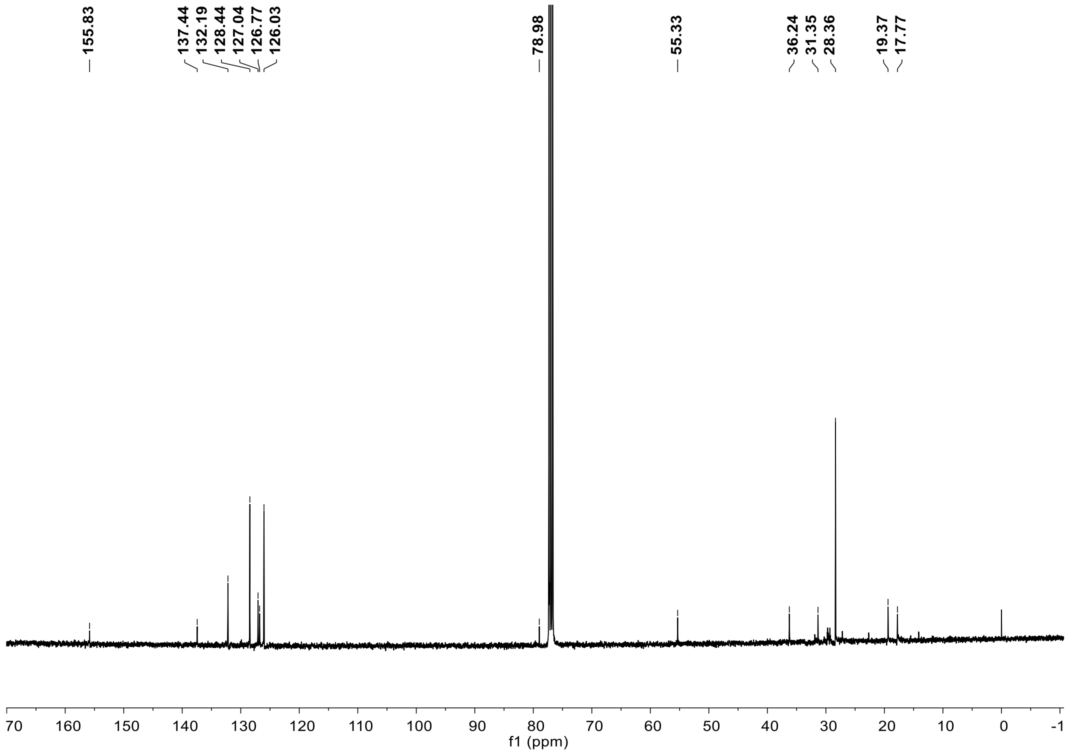

Supplementary Figure 81. ^{13}C NMR spectrum of (*S*)-**3N**

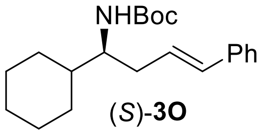

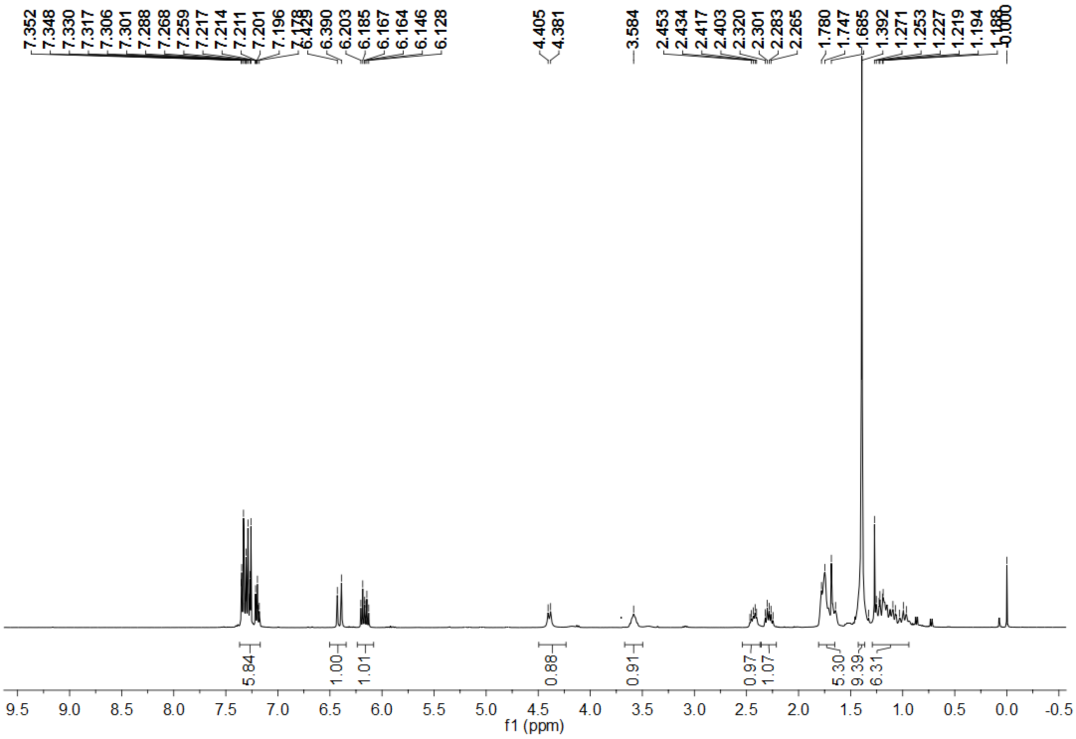

Supplementary Figure 82. ^{1}H NMR spectrum of (*S*)-**3O**

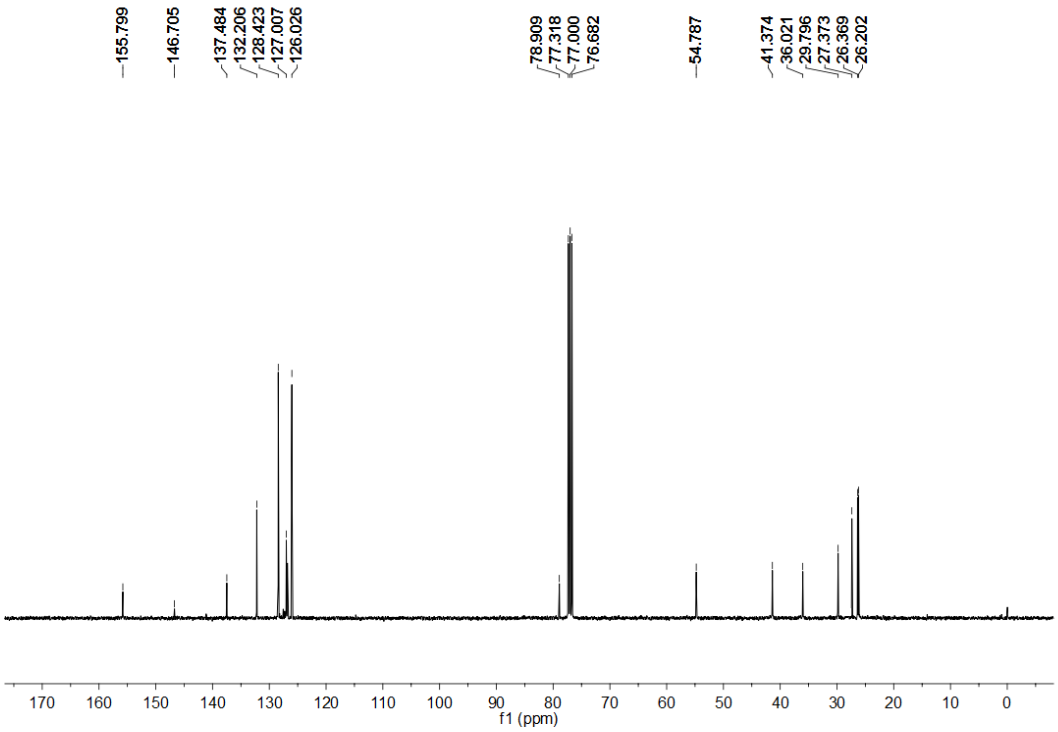

Supplementary Figure 83. ^{13}C NMR spectrum of (*S*)-**3O**

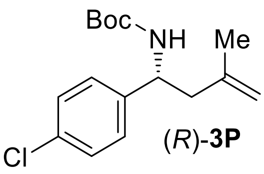

(*R*)-**3P**

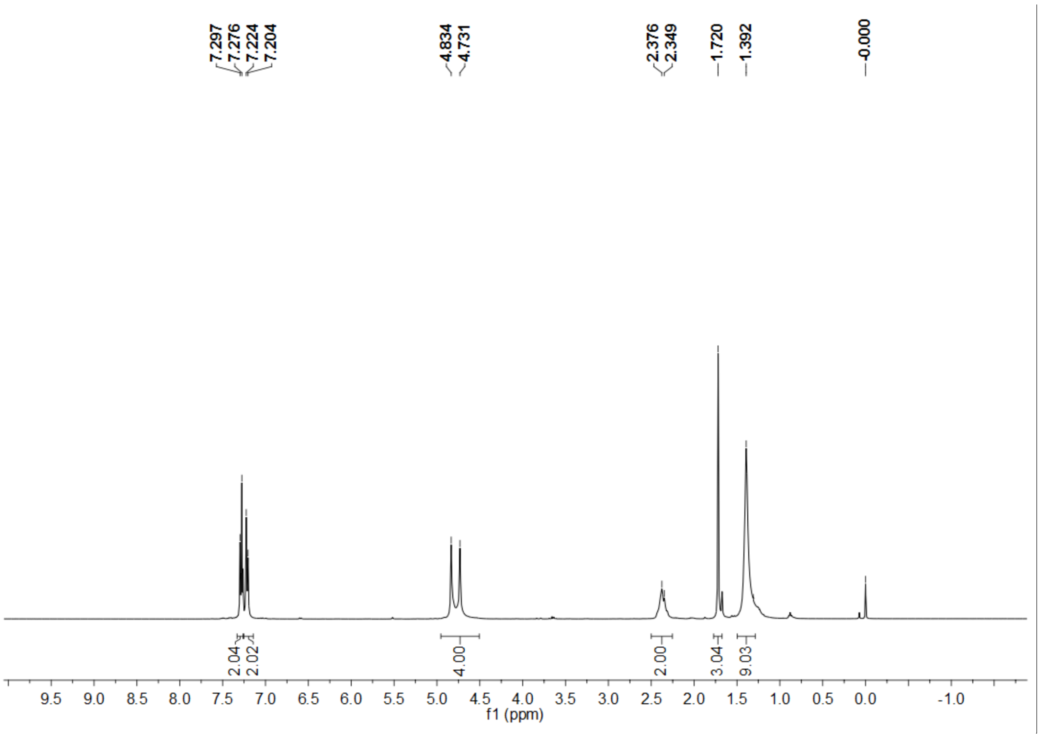

Supplementary Figure 84. ^{1}H NMR spectrum of (*R*)-**3P**

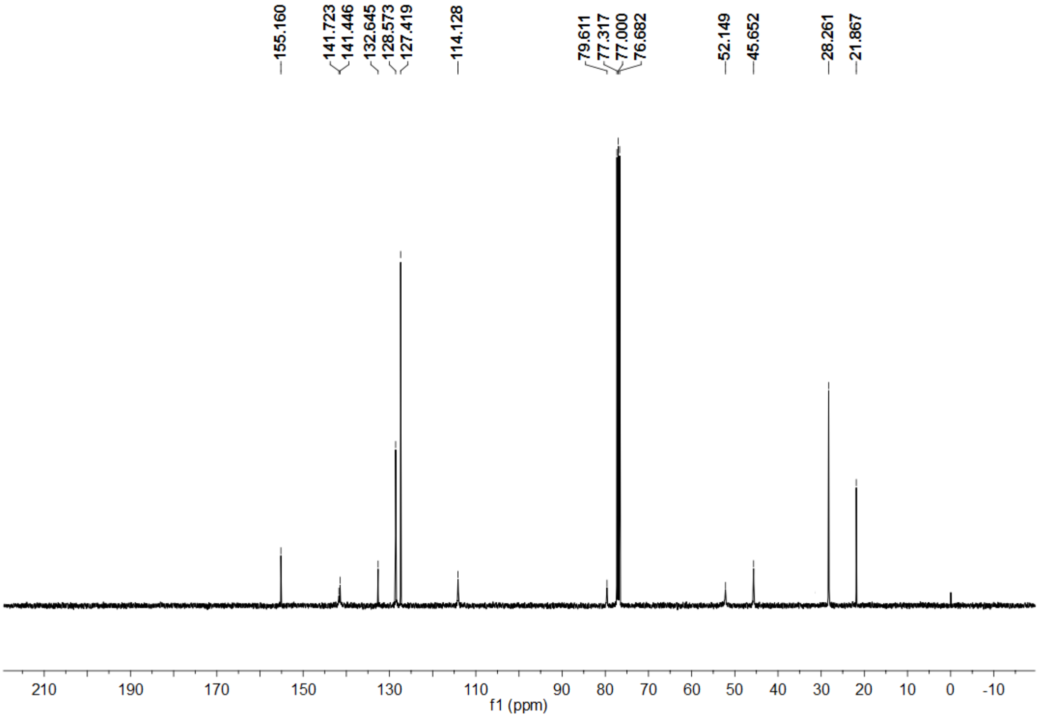

Supplementary Figure 85. ^{13}C NMR spectrum of (*R*)-**3P**

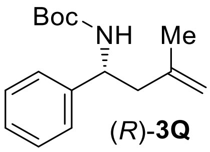

(*R*)-**3Q**

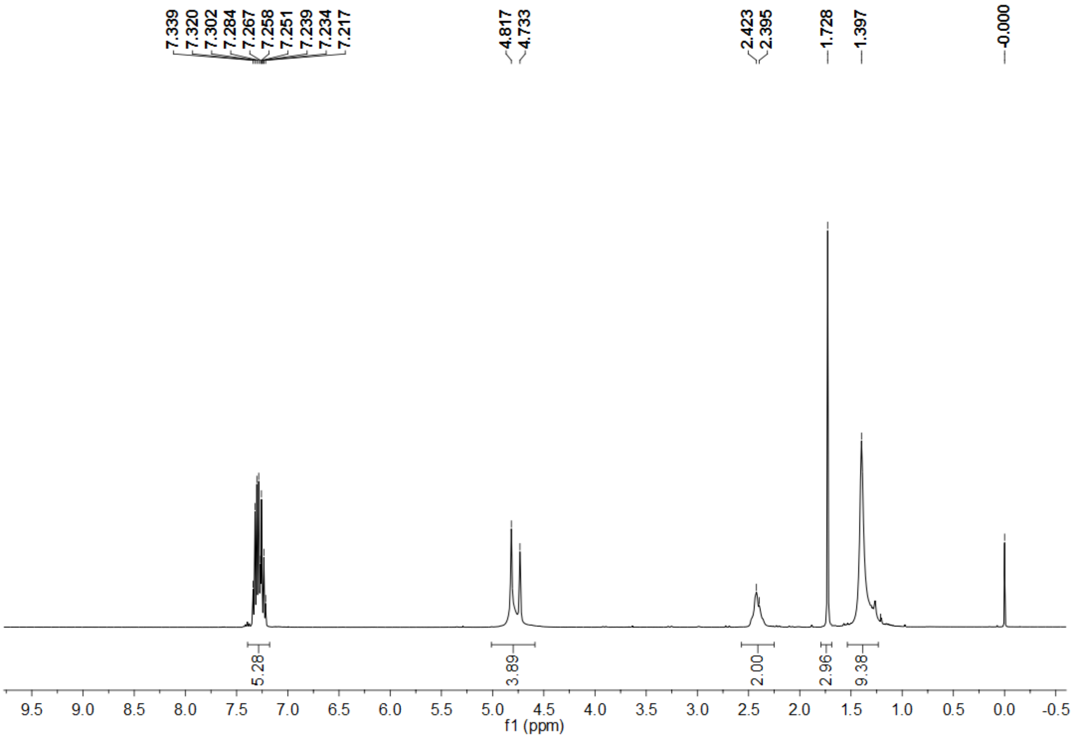

Supplementary Figure 86. ^{1}H NMR spectrum of (*R*)-**3Q**

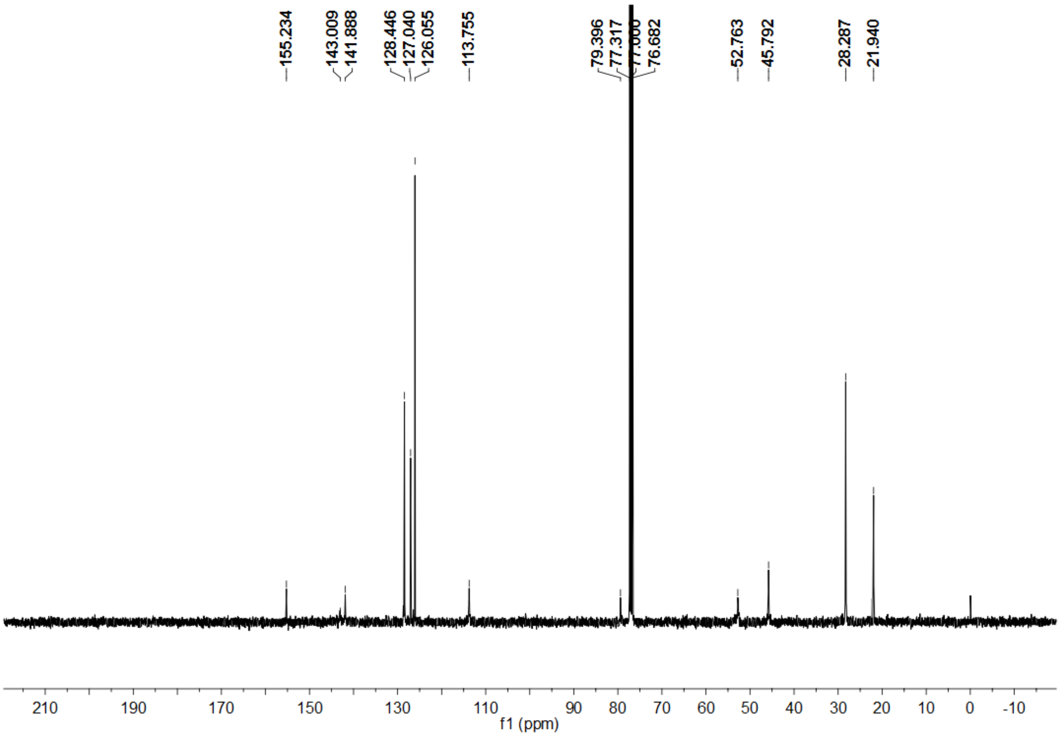

Supplementary Figure 87. ^{13}C NMR spectrum of (*R*)-**3Q**

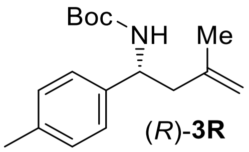

(*R*)-**3R**

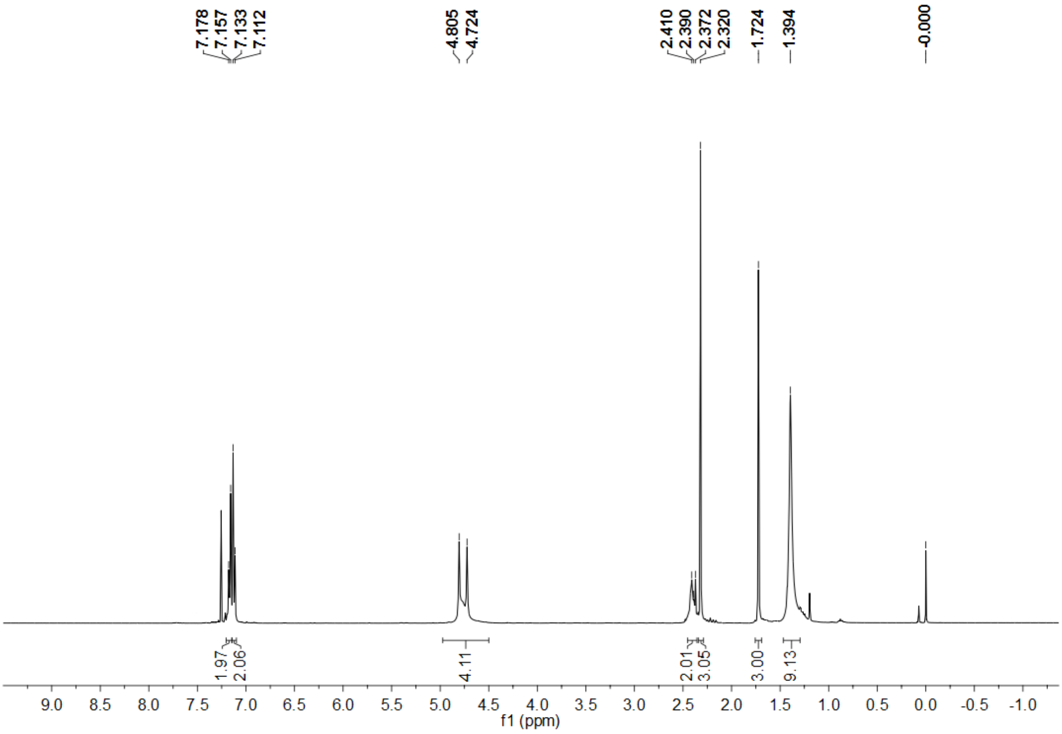

Supplementary Figure 88. ^{1}H NMR spectrum of (*R*)-**3R**

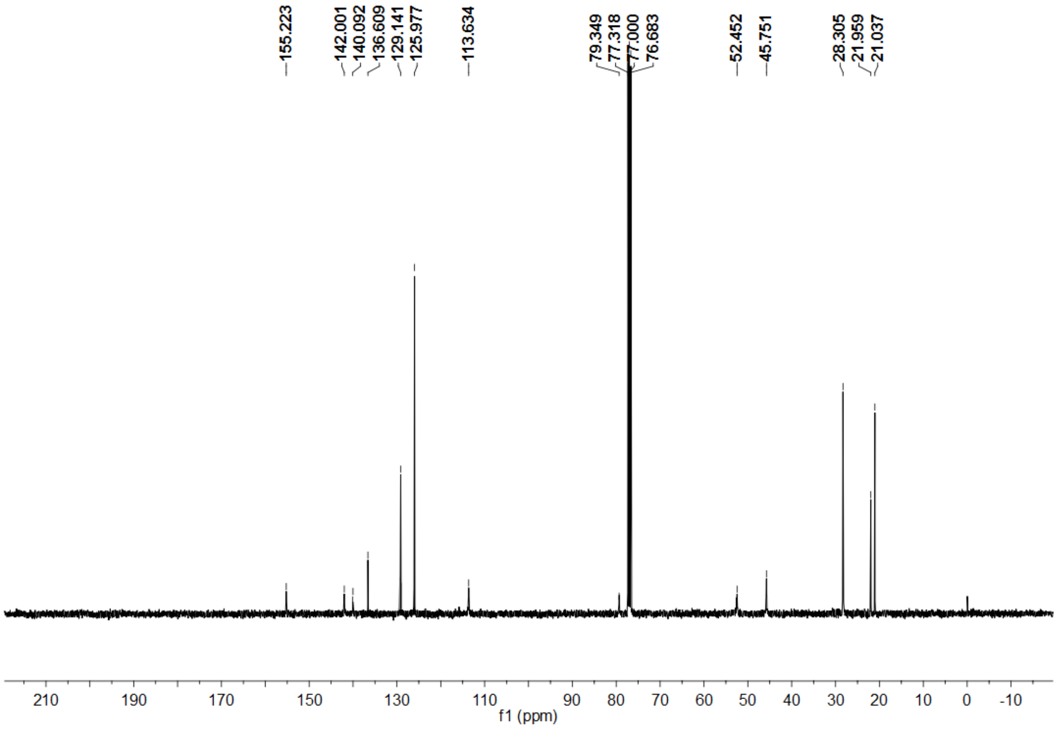

Supplementary Figure 89. ^{13}C NMR spectrum of (*R*)-**3R**

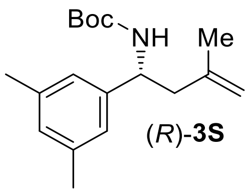

(*R*)-**3S**

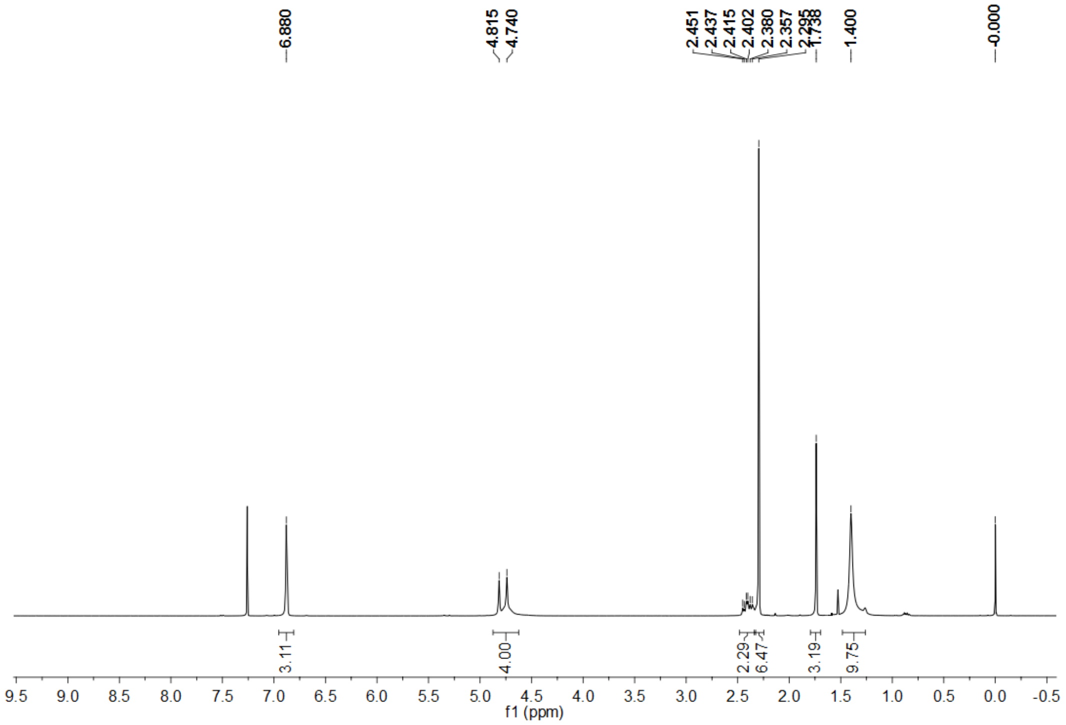

Supplementary Figure 90. ^{1}H NMR spectrum of (*R*)-**3S**

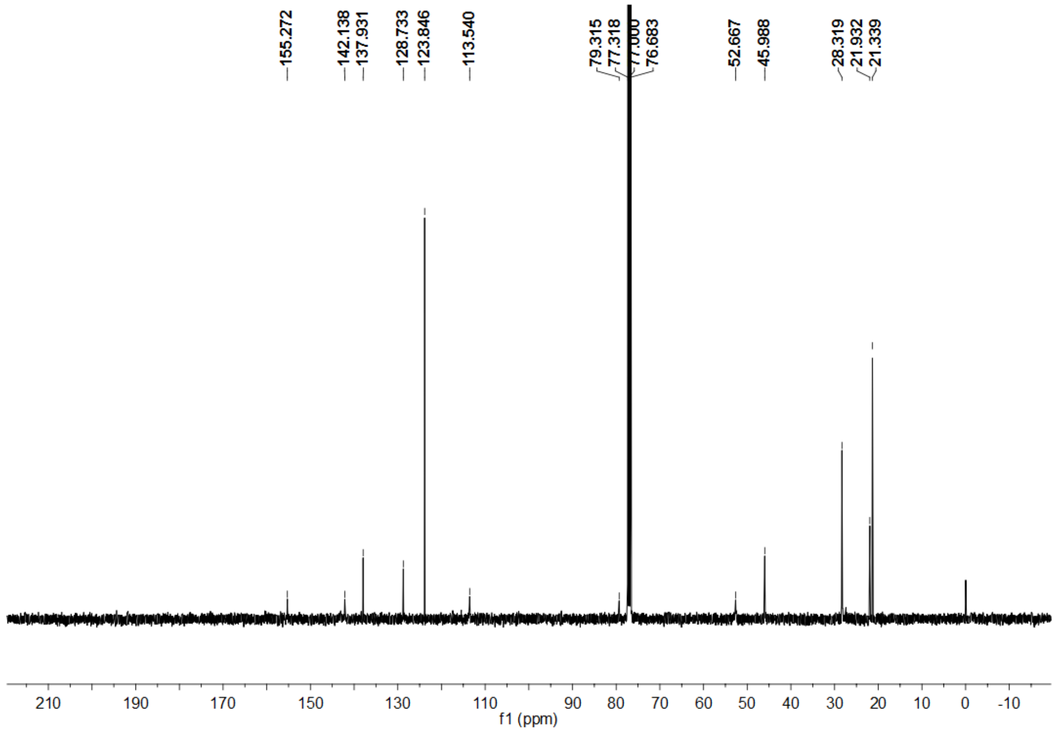

Supplementary Figure 91. ^{13}C NMR spectrum of (*R*)-**3S**

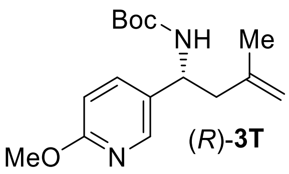

(*R*)-**3T**

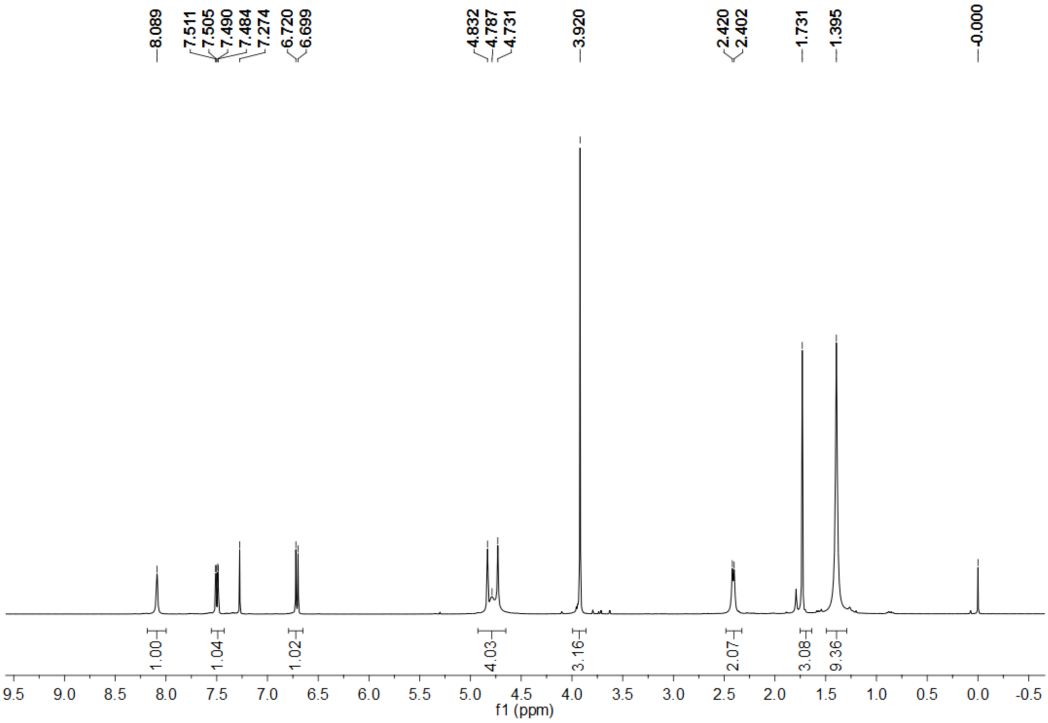

Supplementary Figure 92. ^{1}H NMR spectrum of (*R*)-**3T**

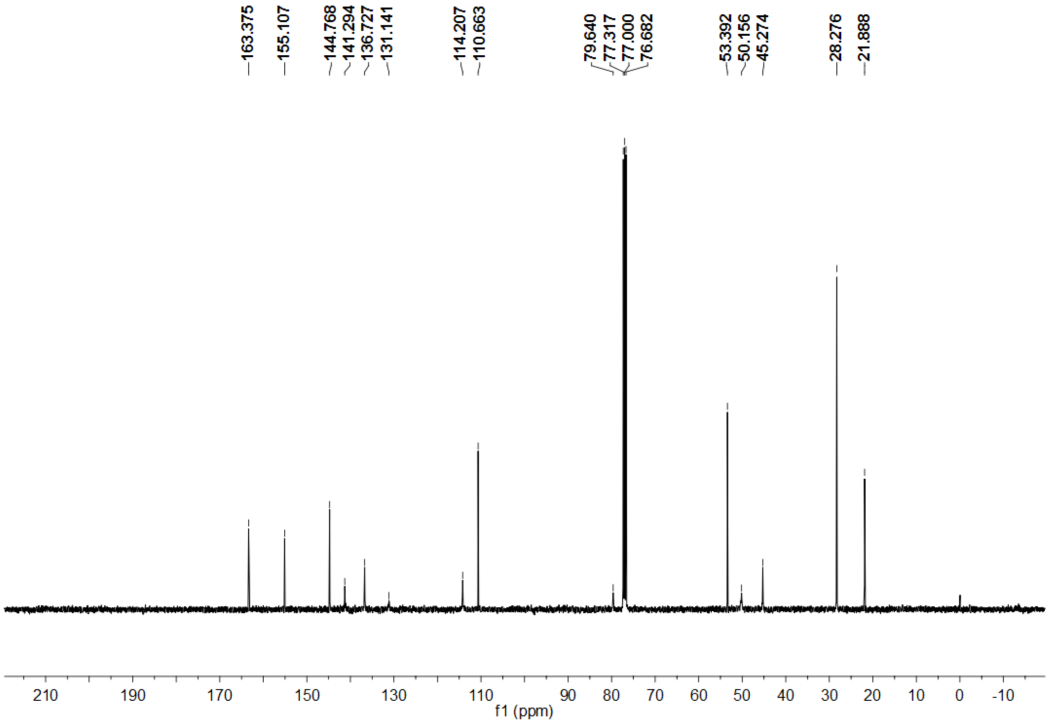

Supplementary Figure 93. ^{13}C NMR spectrum of (*R*)-**3T**

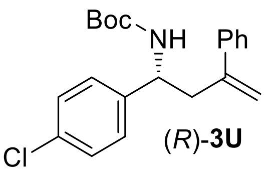

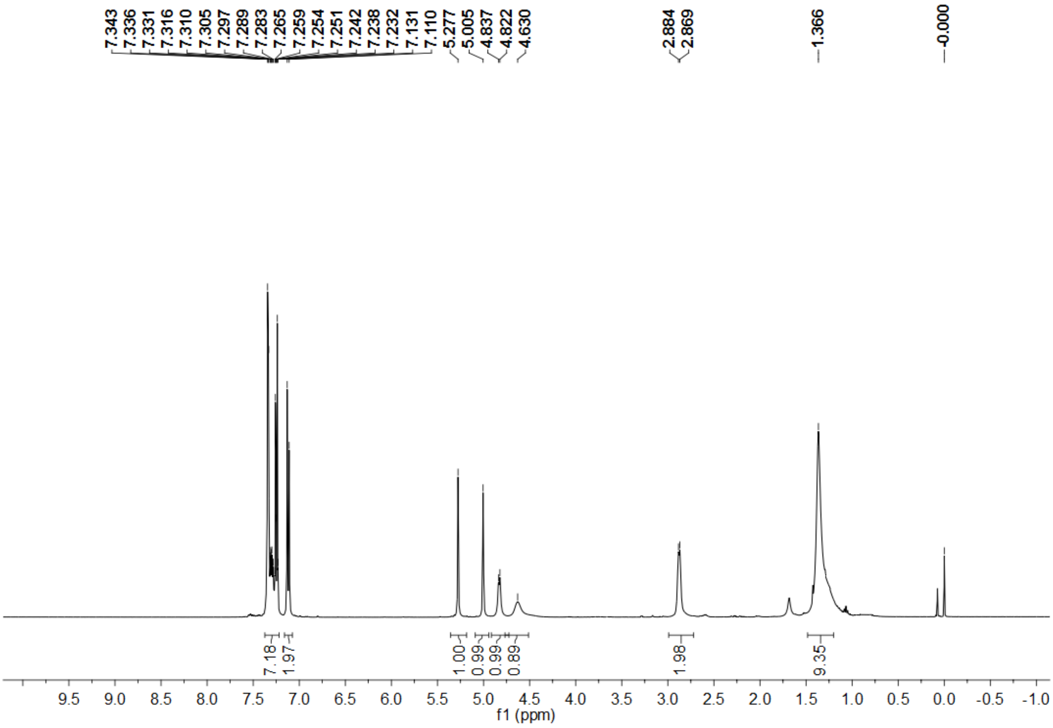

Supplementary Figure 94. ^{1}H NMR spectrum of (*R*)-**3U**

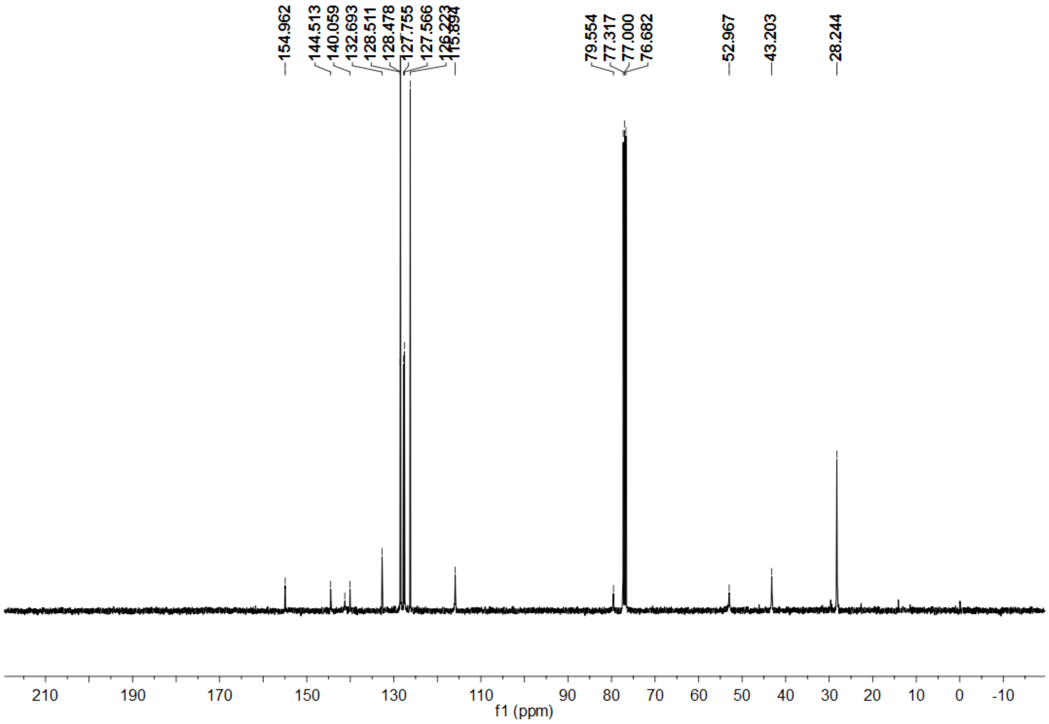

Supplementary Figure 95. ^{13}C NMR spectrum of (*R*)-**3U**

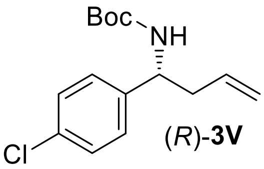

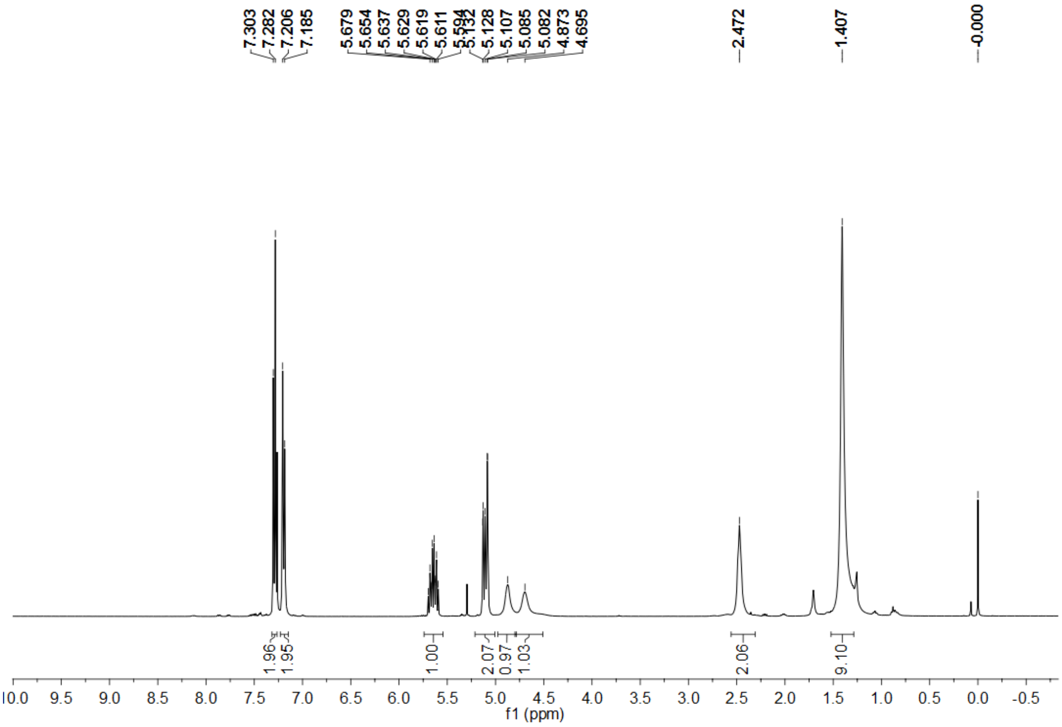

Supplementary Figure 96. ^{1}H NMR spectrum of (*R*)-**3V**

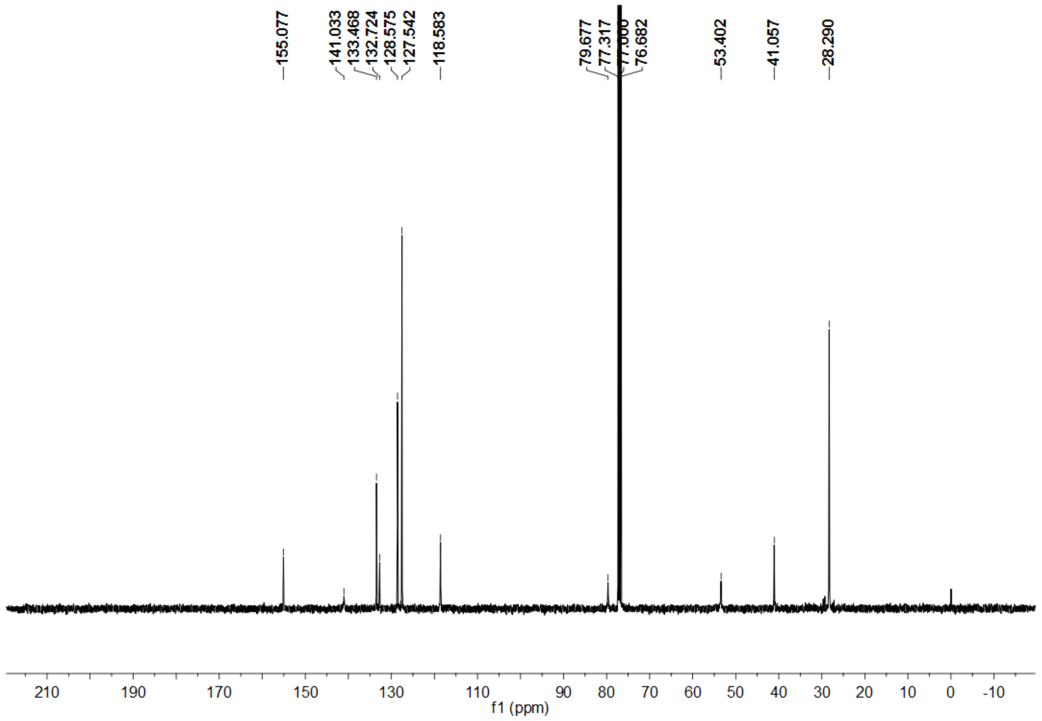

Supplementary Figure 97. ^{13}C NMR spectrum of (*R*)-**3V**

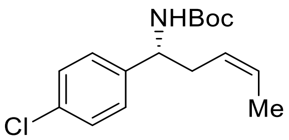

(*R*,*Z*)-**3s**

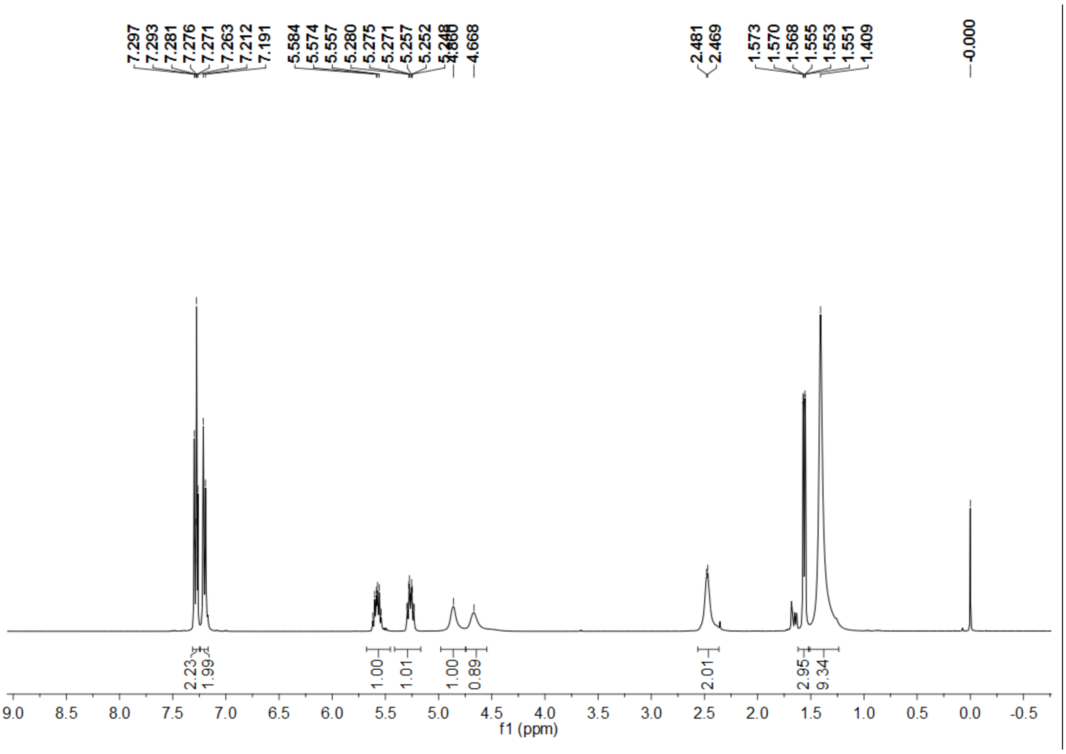

Supplementary Figure 98. ^{1}H NMR spectrum of (*R*,*Z*)-**3s**

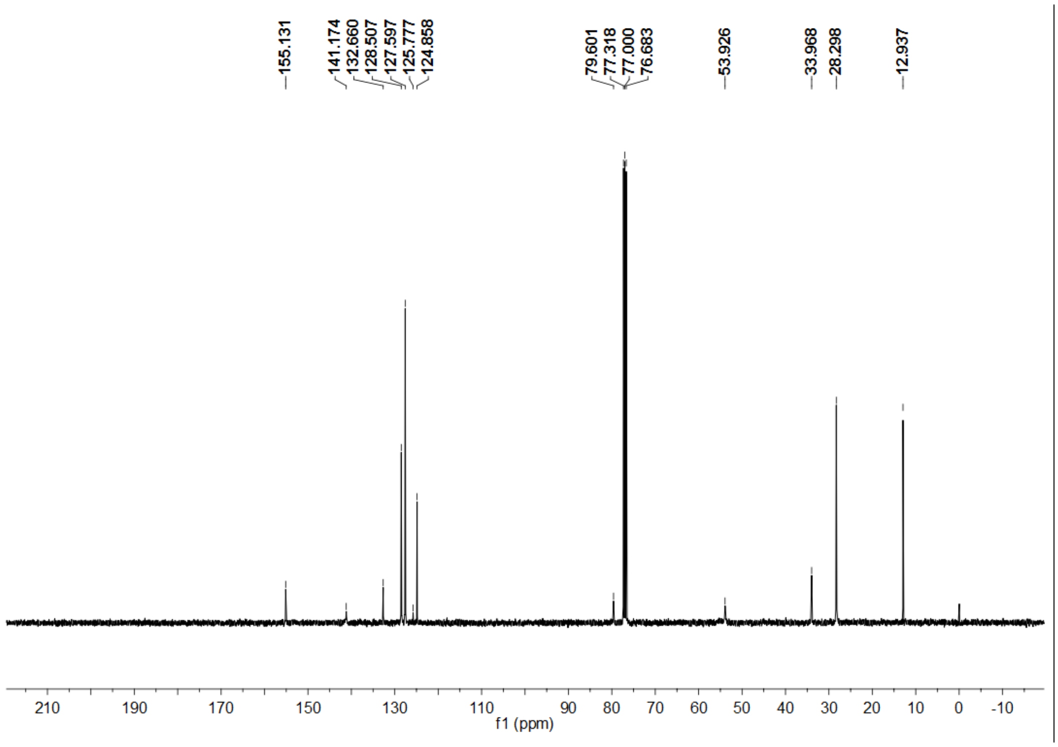

Supplementary Figure 99. ^{13}C NMR spectrum of (*R*,*Z*)-**3s**

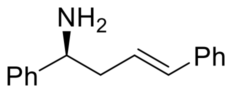

(*S*)-**5**

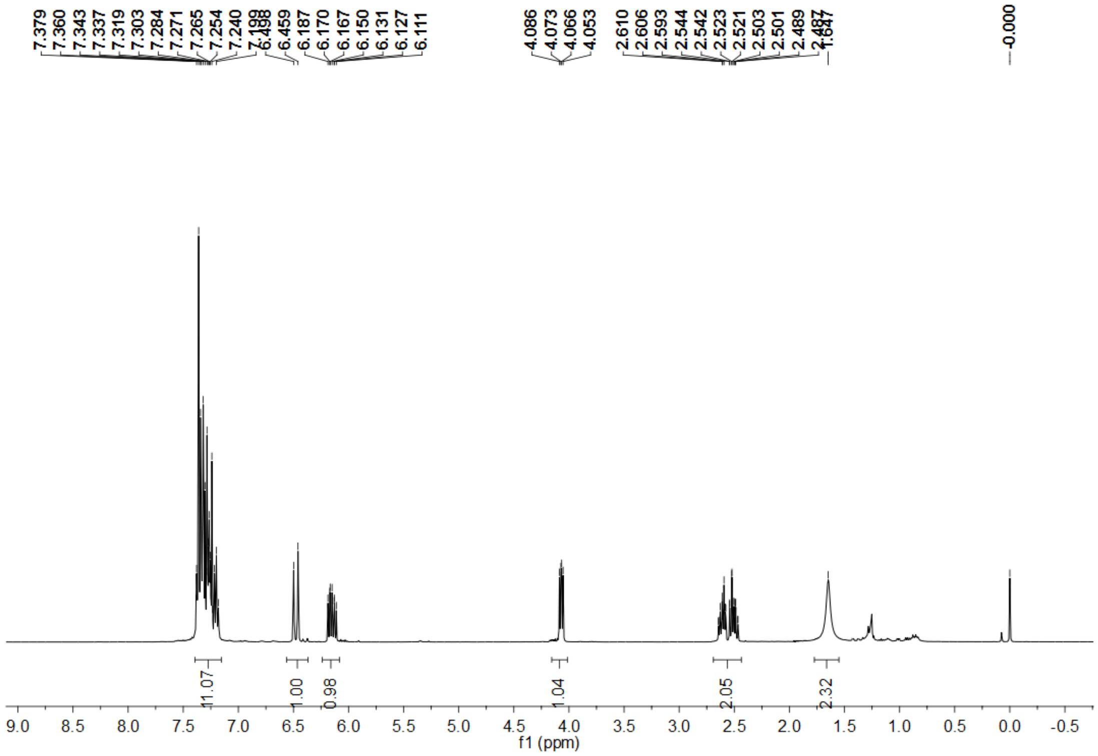

Supplementary Figure 100. ^{1}H NMR spectrum of (*S*)-**5**

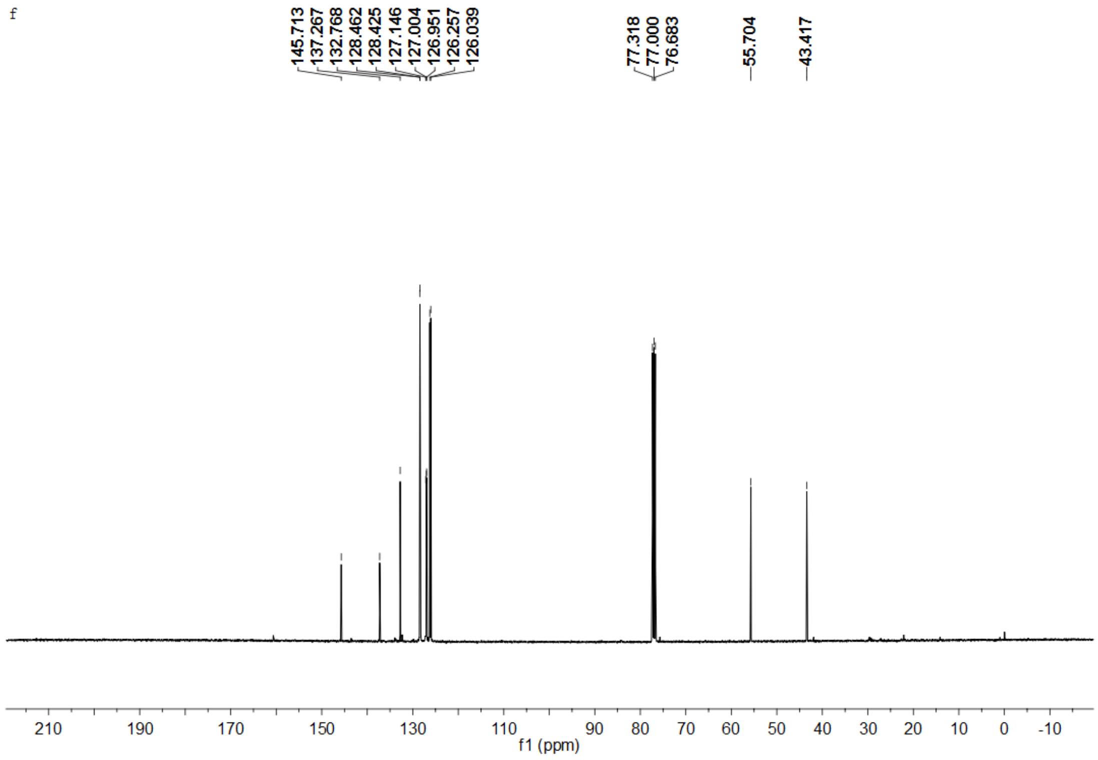

Supplementary Figure 101. ^{13}C NMR spectrum of (*S*)-**5**

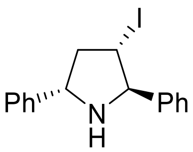

(2*R*,3*S*,5*S*)-**12**

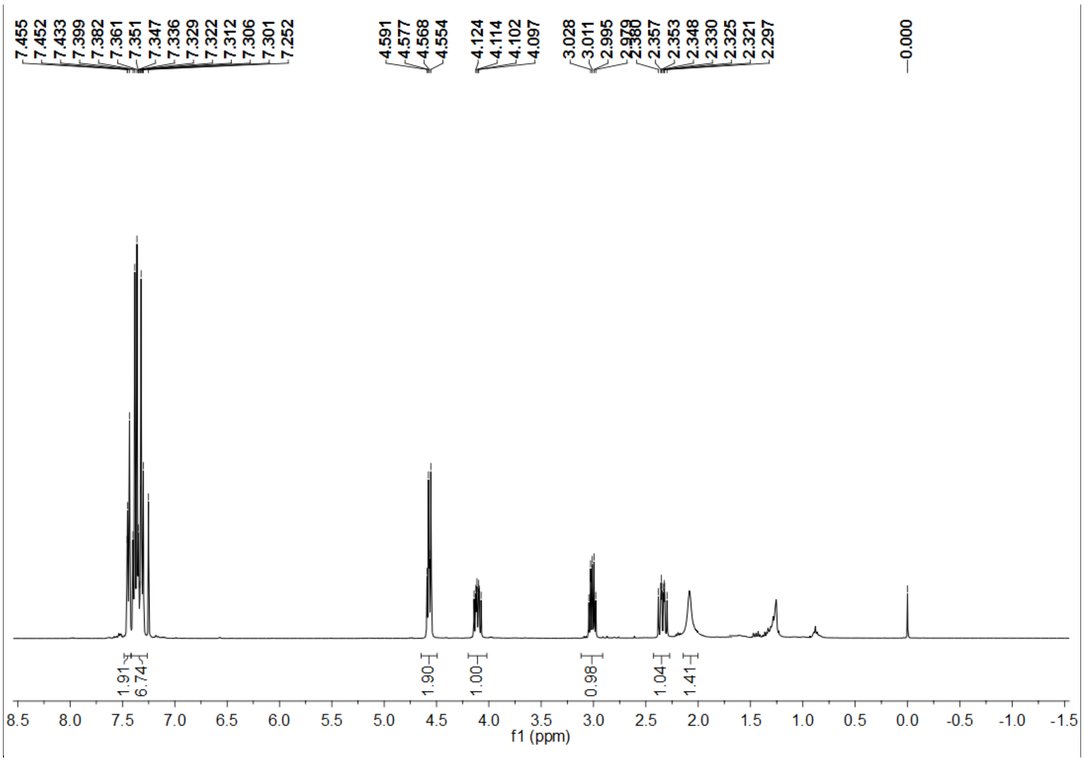

Supplementary Figure 102. ^{1}H NMR spectrum of (2*R*,3*S*,5*S*)-**5**

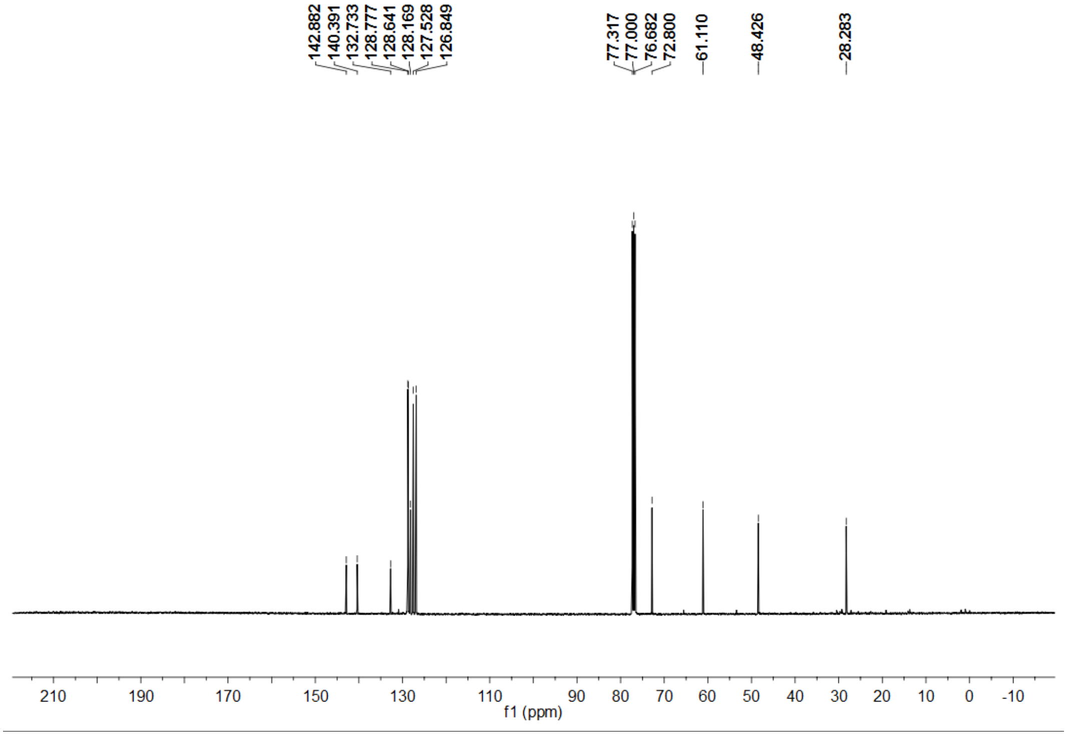

Supplementary Figure 103. ^{13}C NMR spectrum of (2*R*,3*S*,5*S*)-**5**

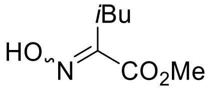

6

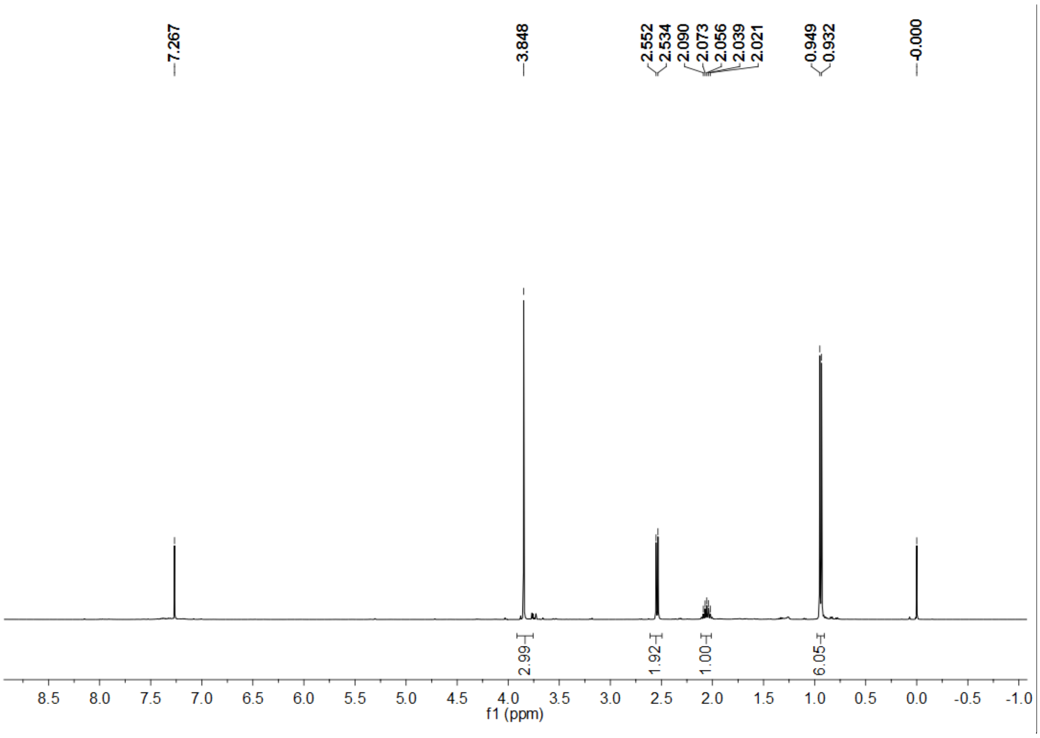

Supplementary Figure 104. ^{1}H NMR spectrum of **6**

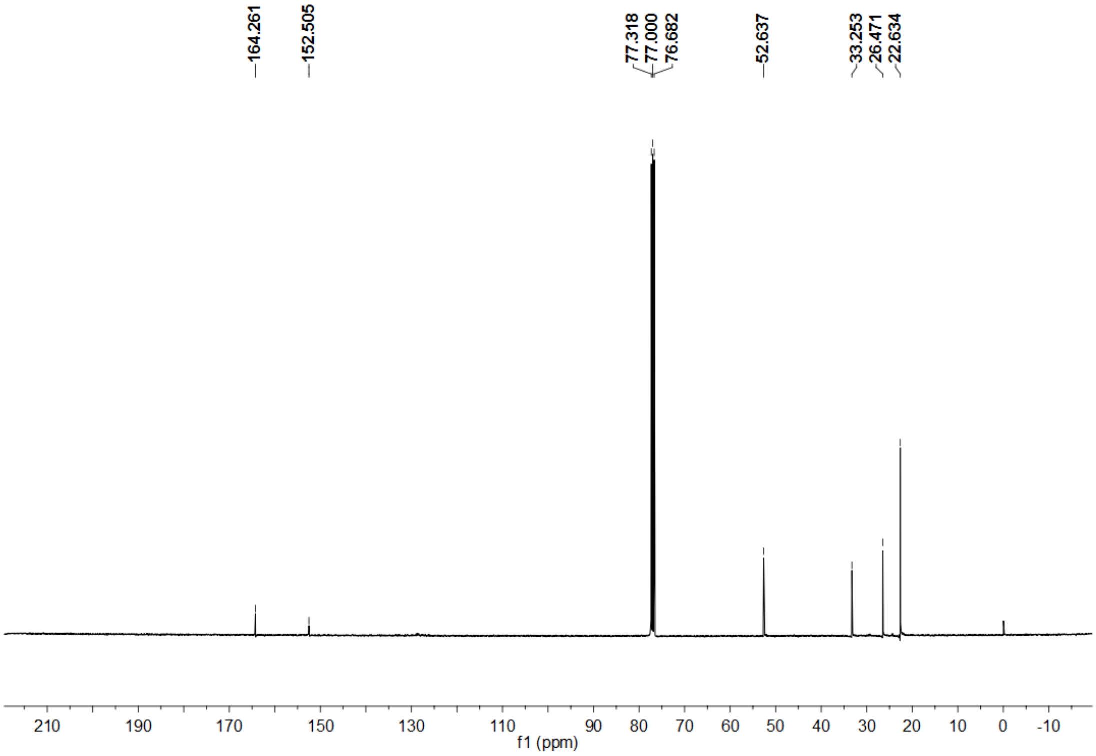

Supplementary Figure 105. ^{13}C NMR spectrum of **6**

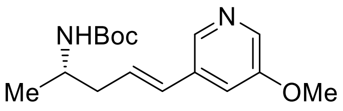

(*S*)-**10**

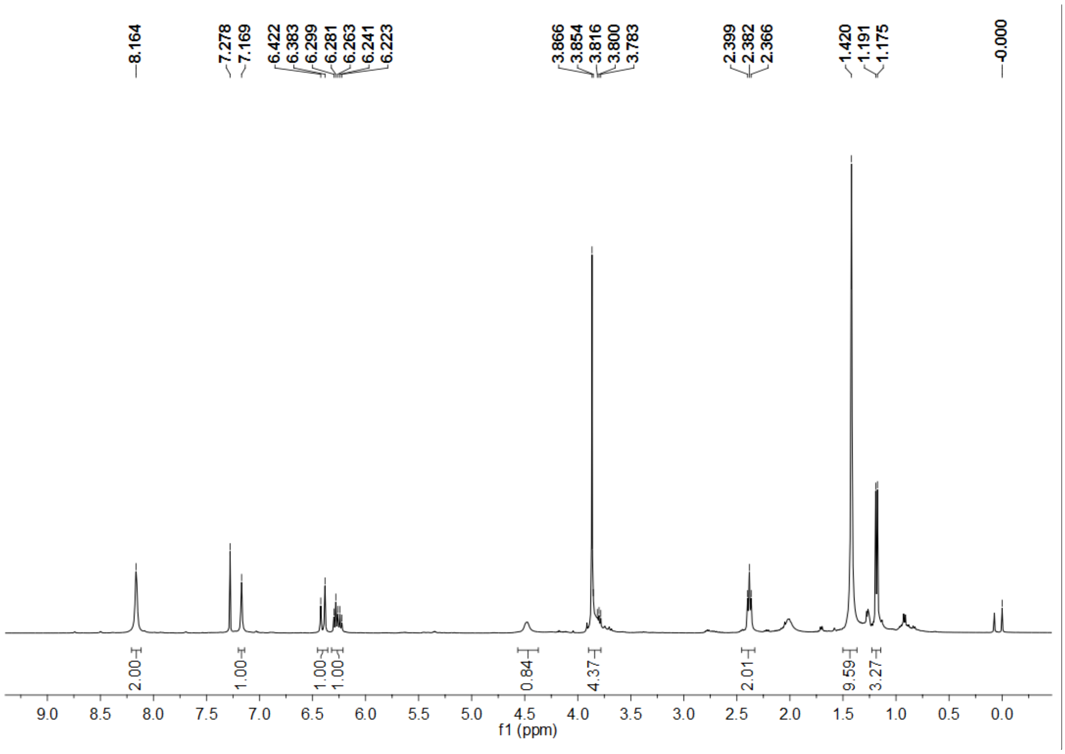

Supplementary Figure 106. ^{1}H NMR spectrum of (*S*)-**10**

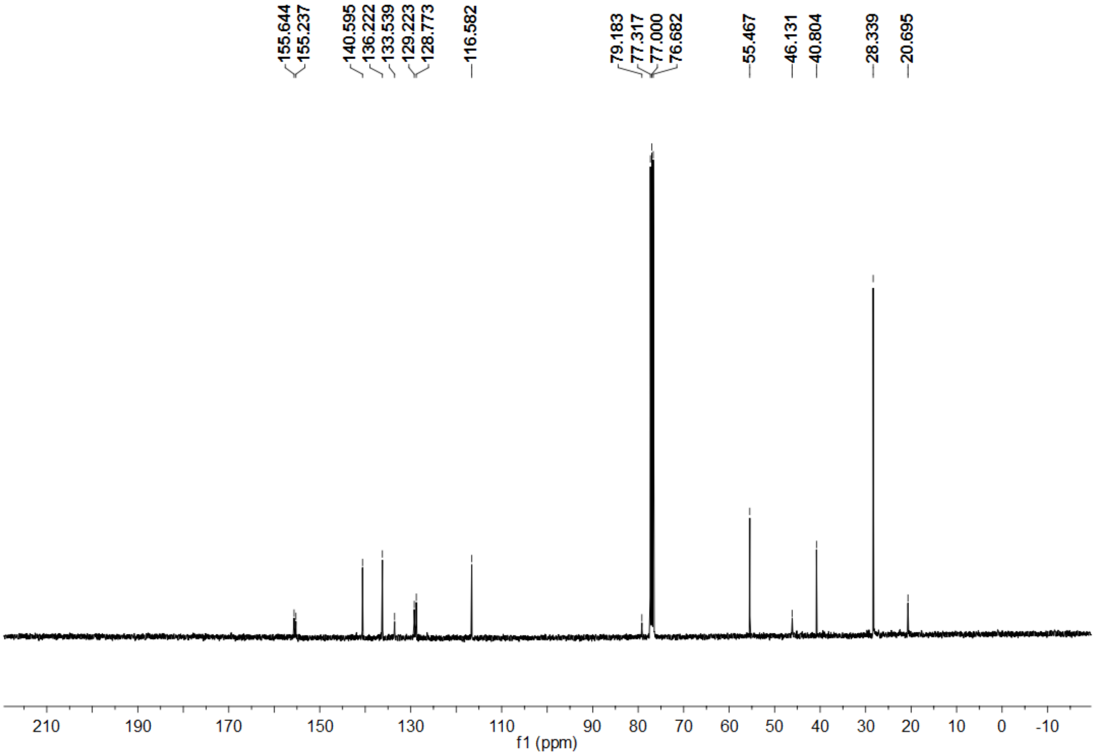

Supplementary Figure 107. ^{13}C NMR spectrum of (*S*)-**10**

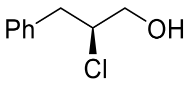

(*S*)-**14**

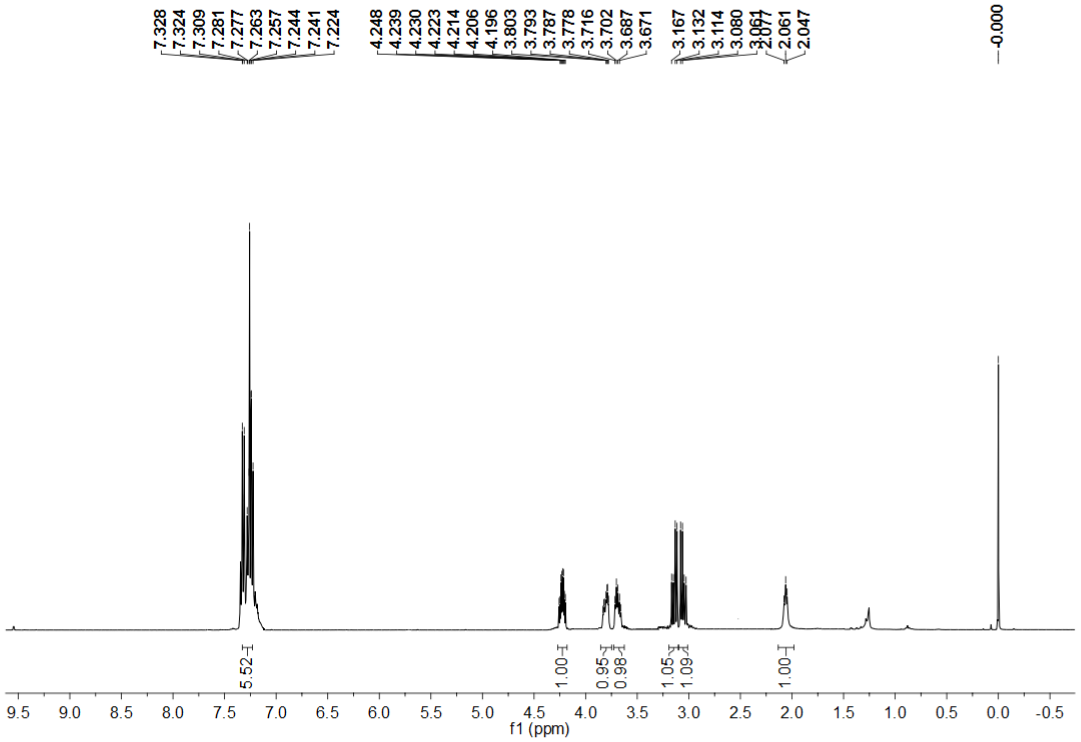

Supplementary Figure 108. ^{1}H NMR spectrum of (*S*)-**14**

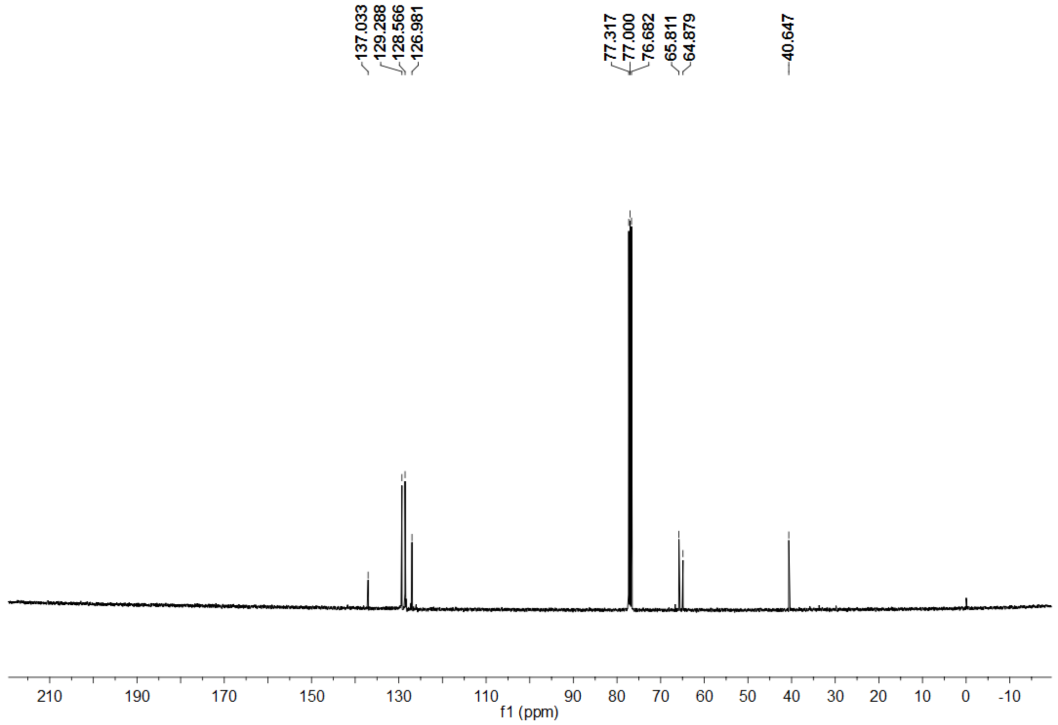

Supplementary Figure 109. ^{13}C NMR spectrum of (*S*)-**14**

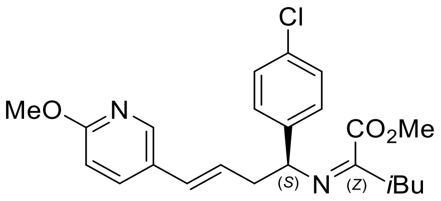

(*S*,*E*,*Z*)-**4q**

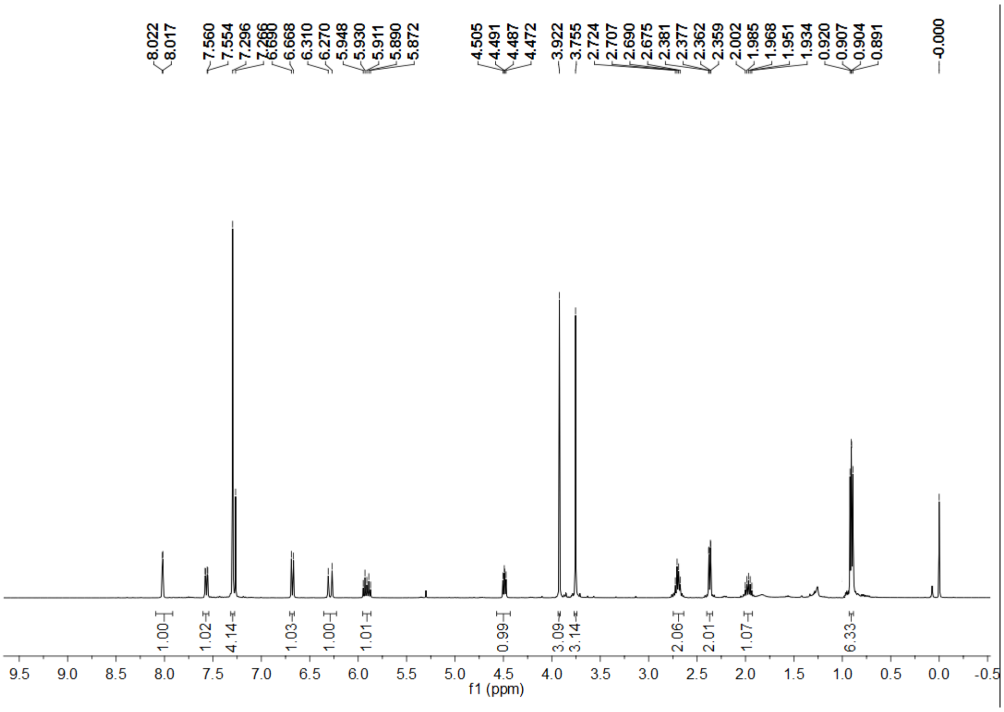

Supplementary Figure 110. ^{1}H NMR spectrum of (*S*,*E*,*Z*)-**4q**

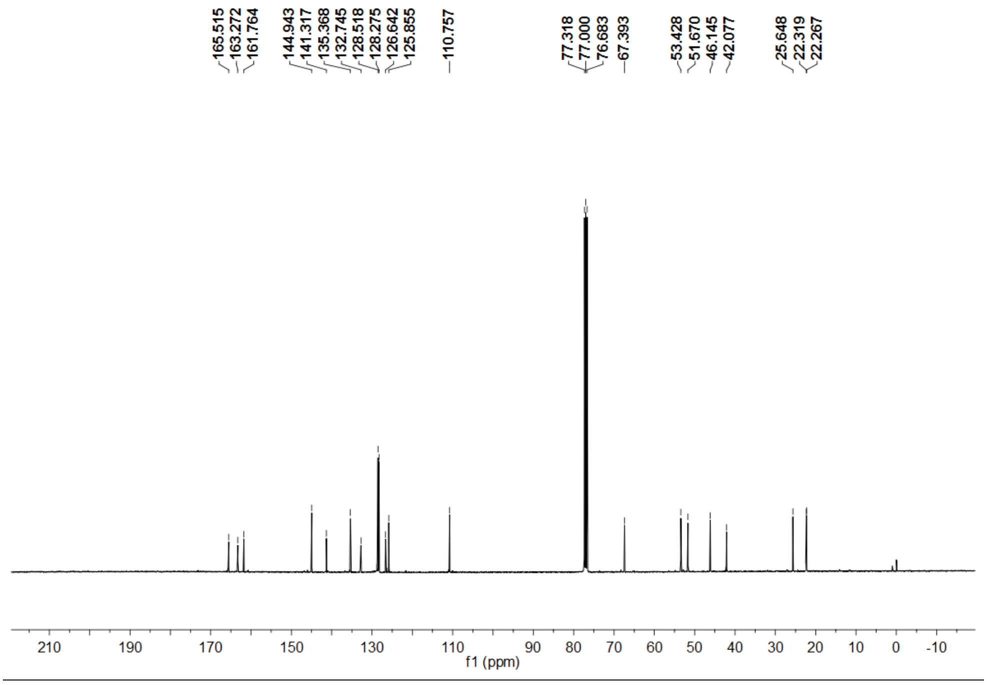

Supplementary Figure 111. ^{13}C NMR spectrum of (*S*,*E*,*Z*)-**4q**

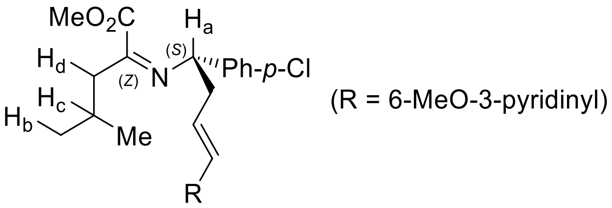

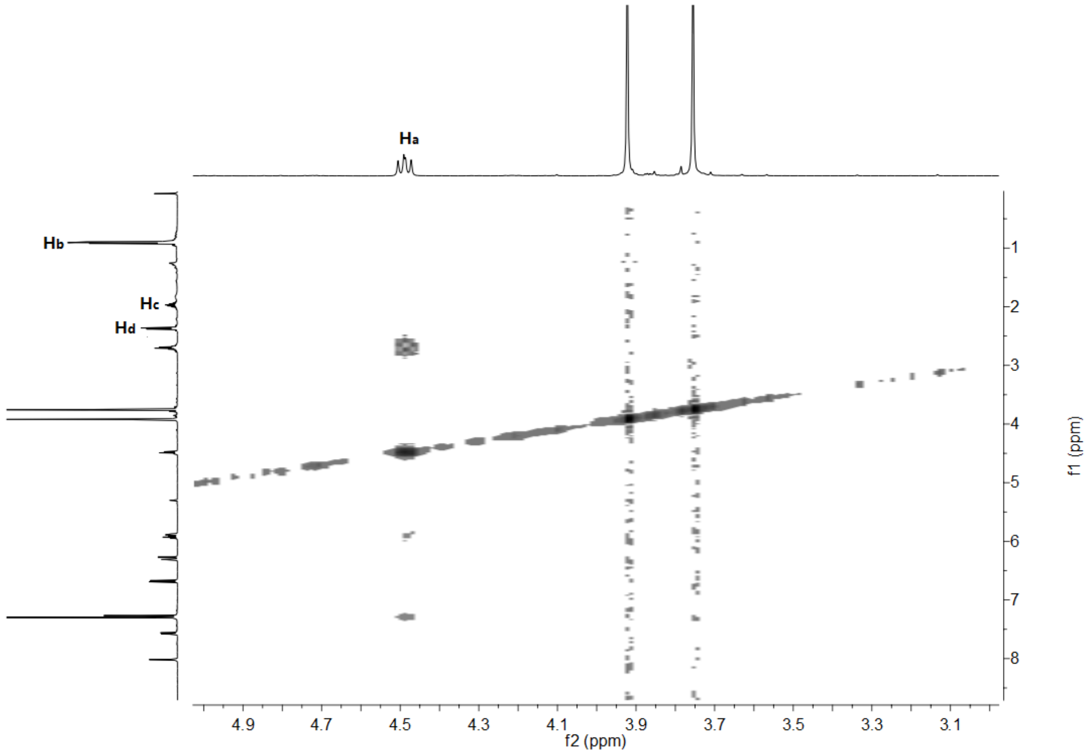

Supplementary Figure 112. NOESY spectrum of (*S*,*E*,*Z*)-**4q**

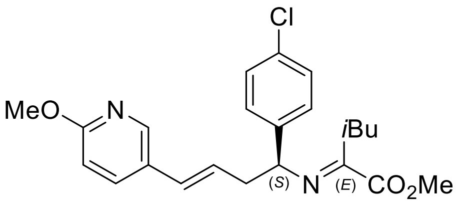

(*S*,*E*,*E*)-**4q**

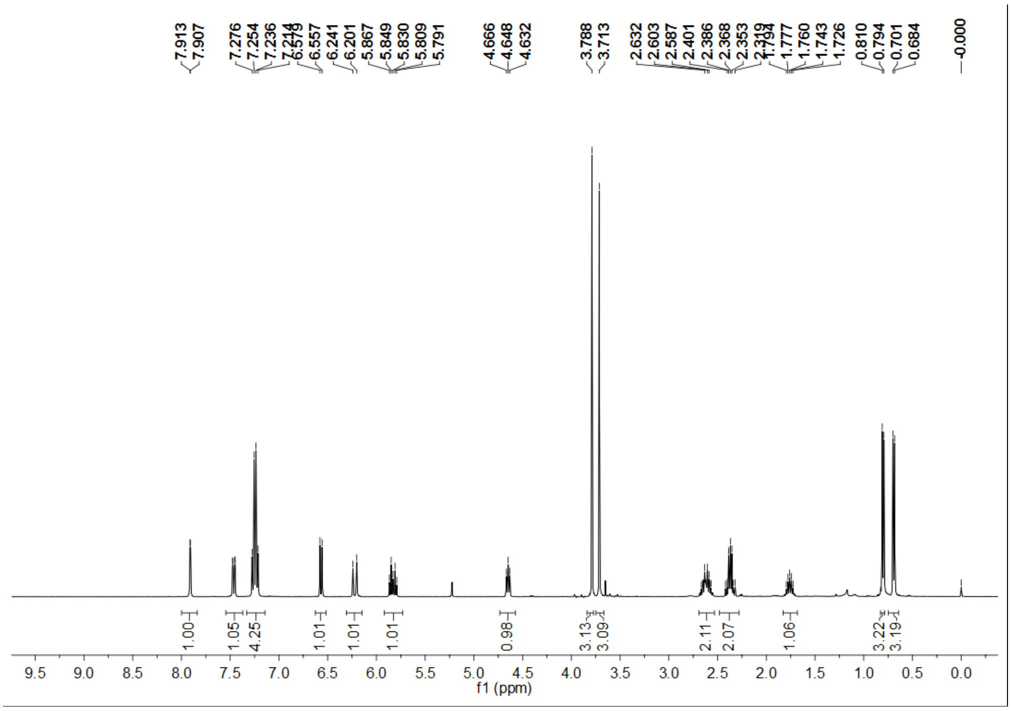

Supplementary Figure 113. ^{1}H NMR spectrum of (*S*,*E*,*E*)-**4q**

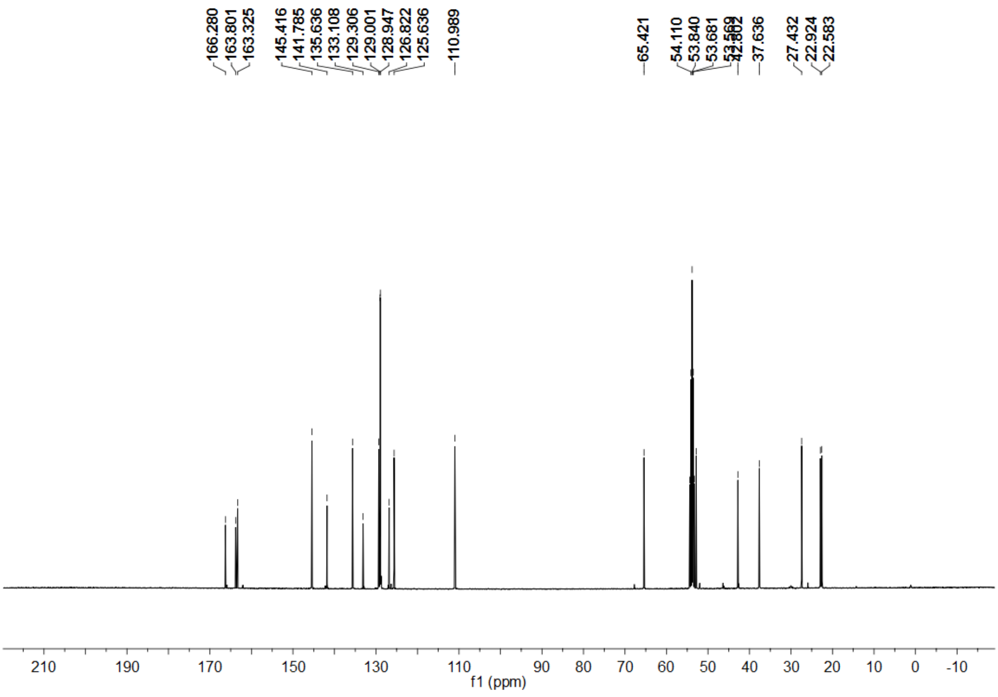

Supplementary Figure 114. ^{13}C NMR spectrum of (*S*,*E*,*E*)-**4q**

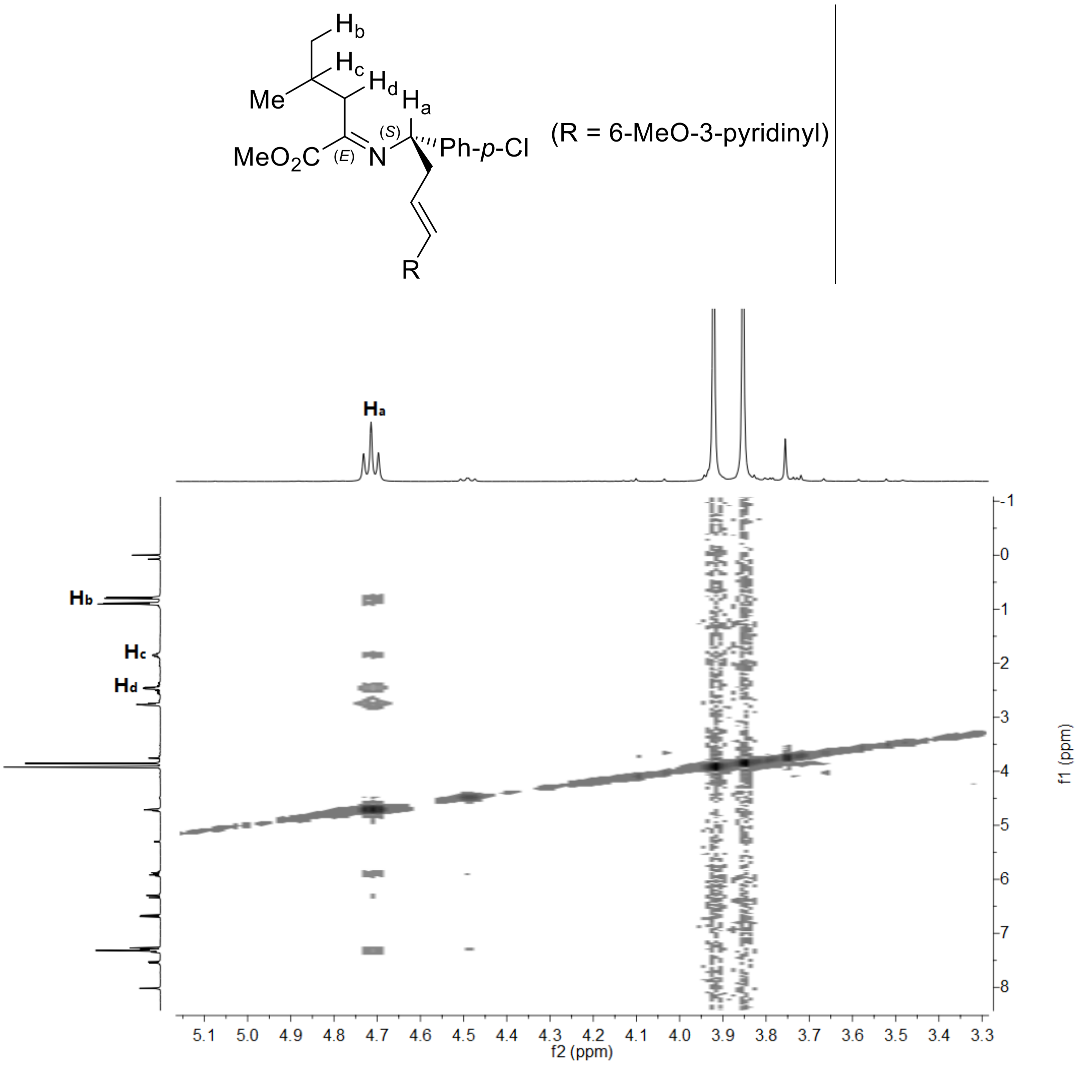

Supplementary Figure 115. NOESY spectrum of (*S*,*E*,*E*)-**4q**

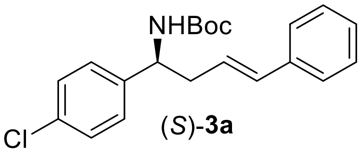

(*S*)-**3a**

Data File E:\DATA\ZQ\ZQ-10-33\ZQ-10-33 2017-12-13 17-44-19\SLM-7-88.D
Sample Name: ZQ-10-33-rac

```
=====================================================================
Acq. Operator   : SYSTEM                          Seq. Line :   1
Acq. Instrument : 1260                             Location :   45
Injection Date  : 12/13/2017 5:45:54 PM                 Inj :   1
                                                 Inj Volume : 5.000 µl
Acq. Method     : E:\DATA\ZQ\ZQ-10-33\ZQ-10-33 2017-12-13 17-44-19\OD-90-10-254NM-20MIN.M
Last changed    : 12/13/2017 5:44:19 PM by SYSTEM
Analysis Method : E:\DATA\ZQ\ZQ-10-33\ZQ-10-33 2017-12-13 17-44-19\OD-90-10-254NM-20MIN.M (
                  Sequence Method)
Last changed    : 4/11/2018 1:56:49 PM by SYSTEM
                  (modified after loading)
Additional Info : Peak(s) manually integrated
```

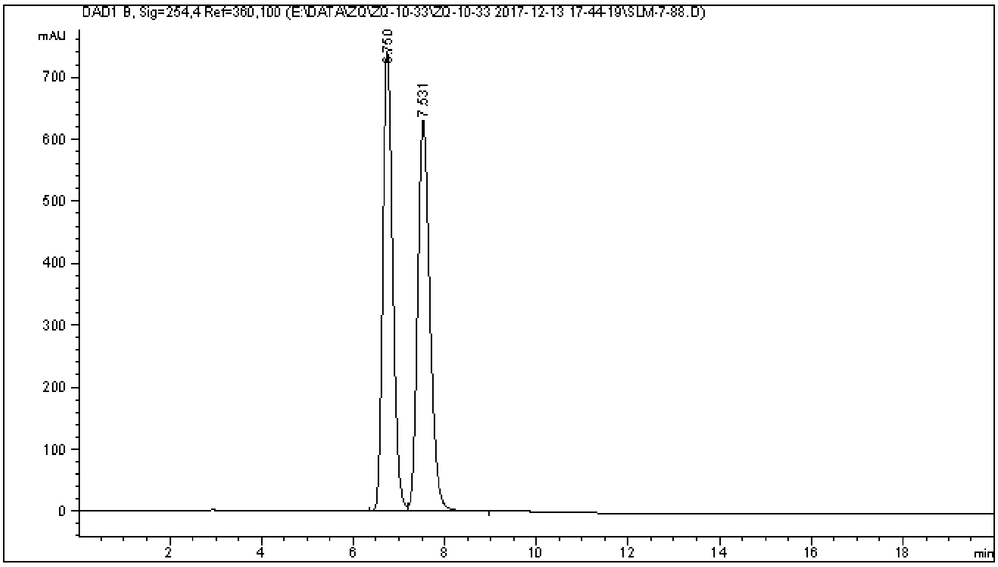


```
=====================================================================
                         Area Percent Report
=====================================================================

Sorted By             :      Signal
Multiplier            :      1.0000
Dilution              :      1.0000
Do not use Multiplier & Dilution Factor with ISTDs


Signal 1: DAD1 B, Sig=254,4 Ref=360,100
```

Peak #	RetTime [min]	Type	Width [min]	Area [mAU*s]	Height [mAU]	Area %
1	6.750	BV	0.2335	1.12050e4	740.54852	49.0770
2	7.531	VB	0.2835	1.16265e4	629.37653	50.9230
Totals :				2.28316e4	1369.92505	

1260 4/11/2018 1:56:56 PM SYSTEM

Supplementary Figure 116. HPLC spectrum of (*rac*)-**3a**

Data File E:\DATA\ZQ\ZQ-10-37\ZQ-10-37 2017-12-15 16-49-13\XSM-20171215.D
Sample Name: ZQ-10-37-DPEphos

===

Acq. Operator : SYSTEM Seq. Line : 1
Acq. Instrument : 1260 Location : 45
Injection Date : 12/15/2017 4:50:45 PM Inj : 1
Inj Volume : 5.000 µl
Acq. Method : E:\DATA\ZQ\ZQ-10-37\ZQ-10-37 2017-12-15 16-49-13\OD-90-10-254NM-10MIN.M
Last changed : 12/15/2017 4:49:13 PM by SYSTEM
Analysis Method : E:\DATA\ZQ\ZQ-10-37\ZQ-10-37 2017-12-15 16-49-13\OD-90-10-254NM-10MIN.M (Sequence Method)
Last changed : 4/11/2018 2:00:48 PM by SYSTEM
(modified after loading)
Additional Info : Peak(s) manually integrated

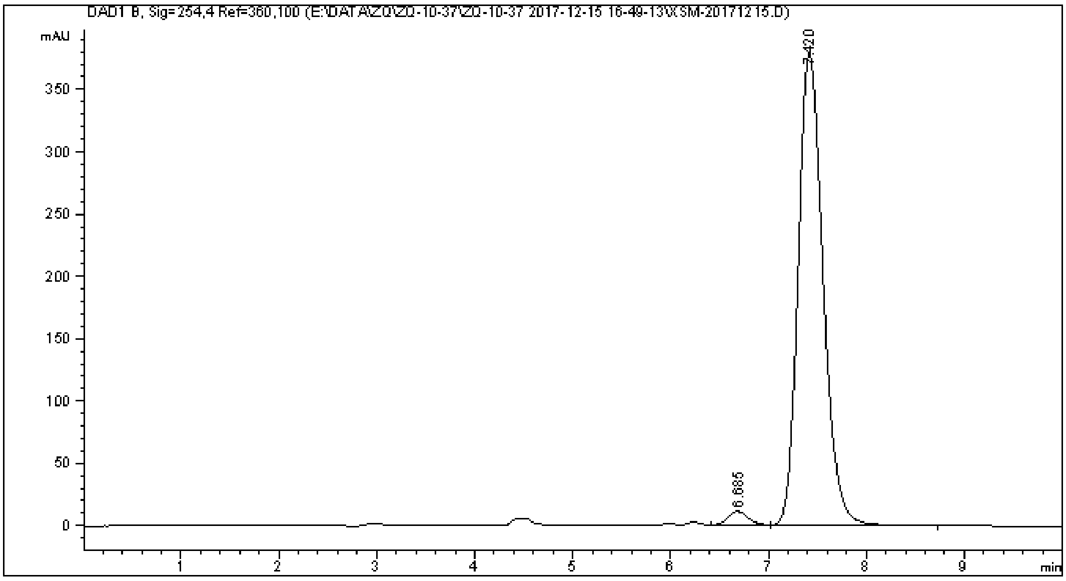

===

Area Percent Report

===

Sorted By : Signal
Multiplier : 1.0000
Dilution : 1.0000
Do not use Multiplier & Dilution Factor with ISTDs

Signal 1: DAD1 B, Sig=254,4 Ref=360,100

Peak #	RetTime [min]	Type	Width [min]	Area [mAU*s]	Height [mAU]	Area %
1	6.685	BV	0.2159	156.47008	11.06945	2.2882
2	7.420	VB	0.2716	6681.62793	379.17935	97.7118

Totals : 6838.09801 390.24880

1260 4/11/2018 2:00:58 PM SYSTEM Page 1 of 2

Supplementary Figure 117. HPLC spectrum of (*S*)-**3a**

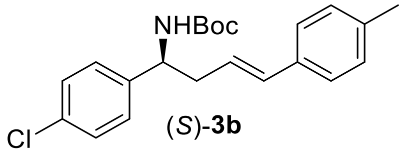

(*S*)-**3b**

Data File E:\DATA\ZQ-10-48\ZQ-10-48 2018-04-21 14-22-44\ZQ-10-48-rac.D
Sample Name: ZQ-10-48-rac

===

Acq. Operator : SYSTEM Seq. Line : 1
Acq. Instrument : 1260 Location : 2
Injection Date : 4/21/2018 2:24:14 PM Inj : 1
Inj Volume : 15.000 µl
Acq. Method : E:\DATA\ZQ-10-48\ZQ-10-48 2018-04-21 14-22-44\AD-90-10-254NM-20MIN-15uL.M
Last changed : 4/21/2018 2:22:44 PM by SYSTEM
Analysis Method : E:\DATA\ZQ-10-48\ZQ-10-48 2018-04-21 14-22-44\AD-90-10-254NM-20MIN-15uL.M (Sequence Method)
Last changed : 4/21/2018 2:39:16 PM by SYSTEM
(modified after loading)
Additional Info : Peak(s) manually integrated

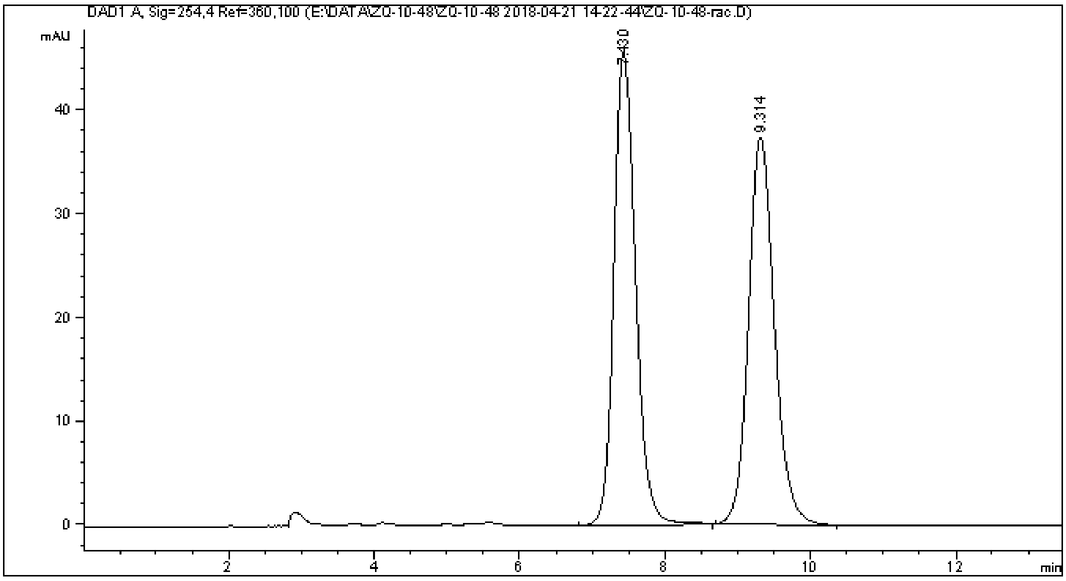

===

Area Percent Report

===

Sorted By : Signal
Multiplier : 1.0000
Dilution : 1.0000
Do not use Multiplier & Dilution Factor with ISTDs

Signal 1: DAD1 A, Sig=254,4 Ref=360,100

Peak #	RetTime [min]	Type	Width [min]	Area [mAU*s]	Height [mAU]	Area %
1	7.430	BB	0.3206	942.94366	45.53107	50.5378
2	9.314	BB	0.3781	922.87634	37.37675	49.4622

Totals : 1865.82001 82.90783

1260 4/21/2018 2:39:23 PM SYSTEM Page 1 of 2

Supplementary Figure 118. HPLC spectrum of (*rac*)-**3b**

Data File E:\DATA\ZQ\ZQ-10-48\ZQ-10-48 2018-01-02 15-31-08\WL-18-88.D
Sample Name: ZQ-10-48-opt

===

Acq. Operator : SYSTEM   Seq. Line : 1
Acq. Instrument : 1260   Location : 74
Injection Date : 1/2/2018 3:32:38 PM   Inj : 1
Inj Volume : 5.000 µl
Acq. Method : E:\DATA\ZQ\ZQ-10-48\ZQ-10-48 2018-01-02 15-31-08\AD-90-10-254NM-20MIN.M
Last changed : 1/2/2018 3:48:40 PM by SYSTEM
(modified after loading)
Analysis Method : E:\DATA\ZQ\ZQ-10-48\ZQ-10-48 2018-01-02 15-31-08\AD-90-10-254NM-20MIN.M (Sequence Method)
Last changed : 4/11/2018 2:48:04 PM by SYSTEM
(modified after loading)

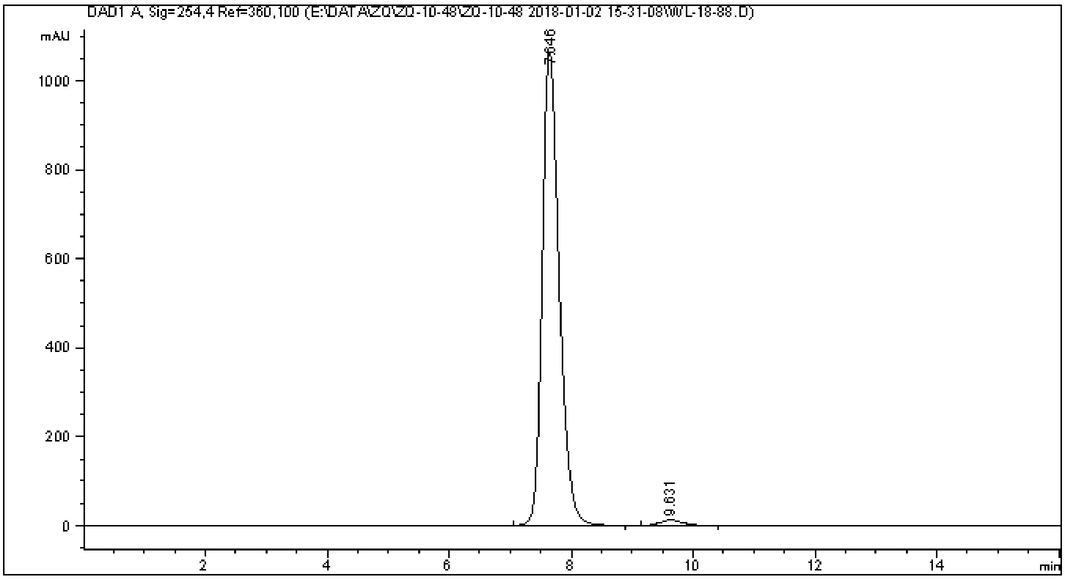

===

Area Percent Report

===

Sorted By : Signal
Multiplier : 1.0000
Dilution : 1.0000
Do not use Multiplier & Dilution Factor with ISTDs

Signal 1: DAD1 A, Sig=254,4 Ref=360,100

Peak #	RetTime [min]	Type	Width [min]	Area [mAU*s]	Height [mAU]	Area %
1	7.646	BB	0.2950	2.05856e4	1062.80750	98.6002
2	9.631	BB	0.3389	292.25519	12.01098	1.3998

Totals : 2.08779e4 1074.81848

Supplementary Figure 119. HPLC spectrum of (*S*)-**3b**

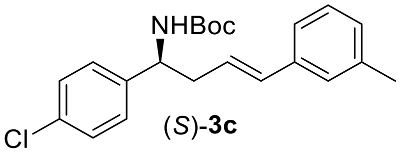

Data File E:\DATA\ZQ\ZQ-10-38\ZQ-10-38 2017-12-27 10-49-54\WL-18-88.D
Sample Name: ZQ-10-38-m-CH3-rac

```
=====================================================================
Acq. Operator   : SYSTEM                        Seq. Line :   1
Acq. Instrument : 1260                           Location :   71
Injection Date  : 12/27/2017 10:51:15 AM              Inj :   1
                                               Inj Volume : 1.000 µl
Acq. Method     : E:\DATA\ZQ\ZQ-10-38\ZQ-10-38 2017-12-27 10-49-54\OD-90-10-254NM-15MIN-1uL.M
Last changed    : 12/27/2017 10:49:54 AM by SYSTEM
Analysis Method : E:\DATA\ZQ\ZQ-10-38\ZQ-10-38 2017-12-27 10-49-54\OD-90-10-254NM-15MIN-1uL.M
                  (Sequence Method)
Last changed    : 4/11/2018 2:03:08 PM by SYSTEM
                  (modified after loading)
Additional Info : Peak(s) manually integrated
```

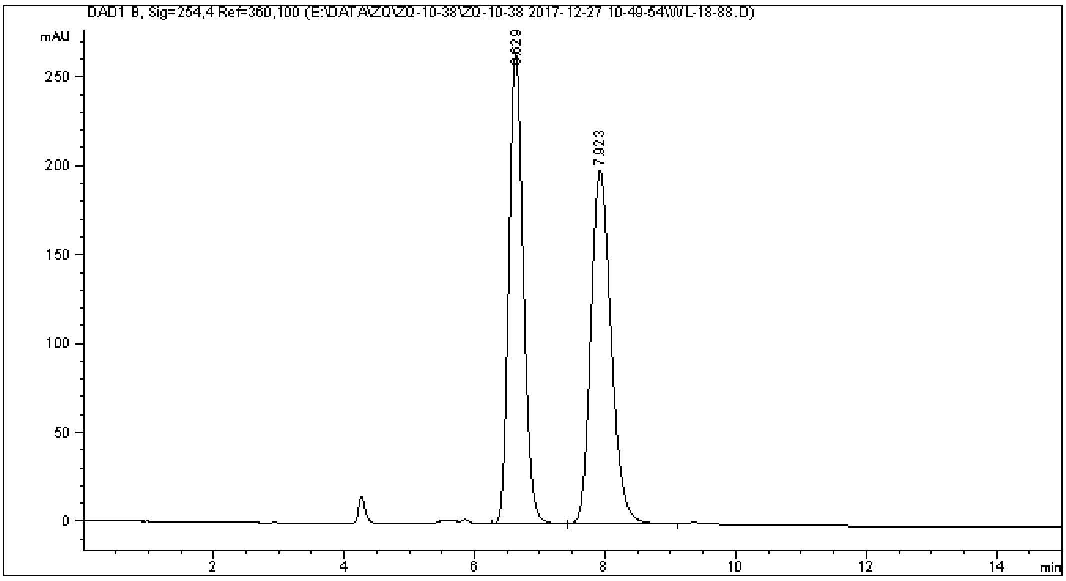


```
=====================================================================
                         Area Percent Report
=====================================================================

Sorted By             :      Signal
Multiplier            :      1.0000
Dilution              :      1.0000
Do not use Multiplier & Dilution Factor with ISTDs


Signal 1: DAD1 B, Sig=254,4 Ref=360,100

Peak RetTime Type  Width     Area      Height     Area
  #   [min]        [min]   [mAU*s]     [mAU]        %
----|-------|----|-------|----------|----------|--------|
   1   6.629 BB    0.2361 4044.97314  264.95731  48.5604
   2   7.923 BB    0.3303 4284.81152  198.89024  51.4396

Totals :                  8329.78467  463.84755
```


Supplementary Figure 120. HPLC spectrum of (*rac*)-**3c**

Data File E:\DATA\ZQ\ZQ-10-38\ZQ-10-38 2017-12-27 10-49-54\WL-18-881.D
Sample Name: ZQ-10-38-m-CH3-opt

===

Acq. Operator : SYSTEM Seq. Line : 2
Acq. Instrument : 1260 Location : 72
Injection Date : 12/27/2017 11:07:39 AM Inj : 1
Inj Volume : 1.000 µl
Acq. Method : E:\DATA\ZQ\ZQ-10-38\ZQ-10-38 2017-12-27 10-49-54\OD-90-10-254NM-15MIN-1uL.M
Last changed : 12/27/2017 10:49:54 AM by SYSTEM
Analysis Method : E:\DATA\ZQ\ZQ-10-38\ZQ-10-38 2017-12-27 10-49-54\OD-90-10-254NM-15MIN-1uL.M
(Sequence Method)
Last changed : 4/11/2018 2:03:08 PM by SYSTEM
(modified after loading)

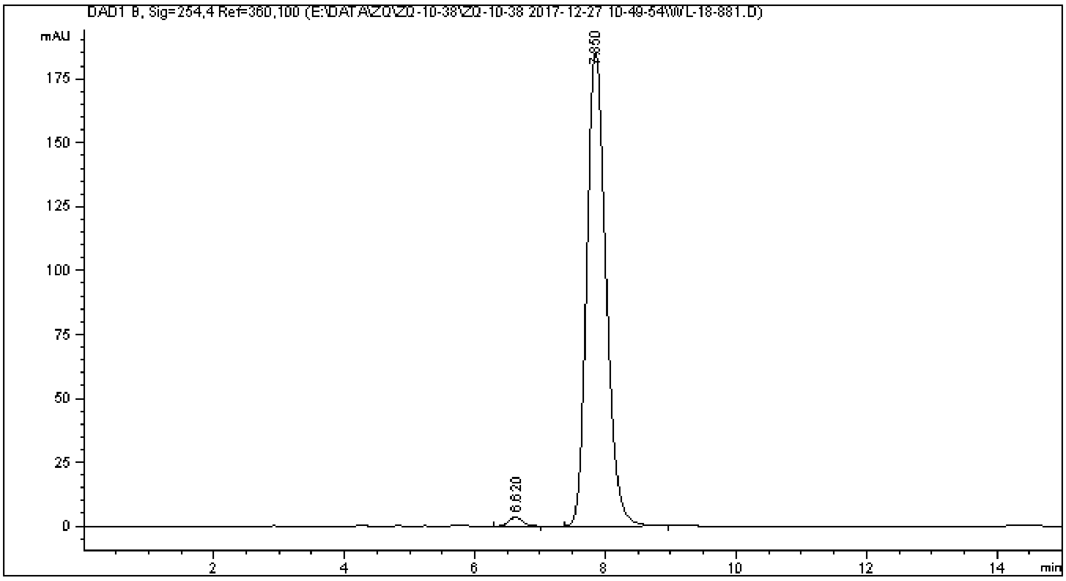

===

Area Percent Report

===

Sorted By : Signal
Multiplier : 1.0000
Dilution : 1.0000
Do not use Multiplier & Dilution Factor with ISTDs

Signal 1: DAD1 B, Sig=254,4 Ref=360,100

Peak #	RetTime [min]	Type	Width [min]	Area [mAU*s]	Height [mAU]	Area %
1	6.620	BB	0.2037	54.86750	3.66351	1.4273
2	7.850	BB	0.3138	3789.17847	185.08846	98.5727

Totals : 3844.04597 188.75197

===

1260 4/11/2018 2:05:06 PM SYSTEM Page 1 of 2

Supplementary Figure 121. HPLC spectrum of (*S*)-**3c**

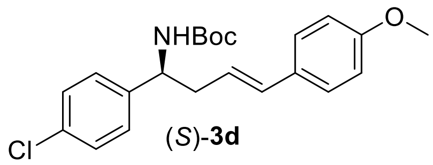

(*S*)-**3d**

```
Data File E:\DATA\WL\WL-18-86\WL-18-86ABCDE 2017-12-14 23-09-43\WL-18-862.D
Sample Name: WL-18-86B-rac

    =====================================================================
    Acq. Operator   : SYSTEM                        Seq. Line :   3
    Acq. Instrument : 1260                           Location :   23
    Injection Date  : 12/14/2017 11:43:48 PM              Inj :   1
                                                   Inj Volume : 5.000 µl
    Acq. Method     : E:\DATA\WL\WL-18-86\WL-18-86ABCDE 2017-12-14 23-09-43\ODH-90-10-1.0ML-ALL-
                      254NM-30MIN.M
    Last changed    : 12/14/2017 11:10:01 PM by SYSTEM
    Analysis Method : E:\DATA\WL\WL-18-86\WL-18-86ABCDE 2017-12-14 23-09-43\ODH-90-10-1.0ML-ALL-
                      254NM-30MIN.M (Sequence Method)
    Last changed    : 5/2/2018 8:26:38 PM by SYSTEM
                      (modified after loading)
    Additional Info : Peak(s) manually integrated
```

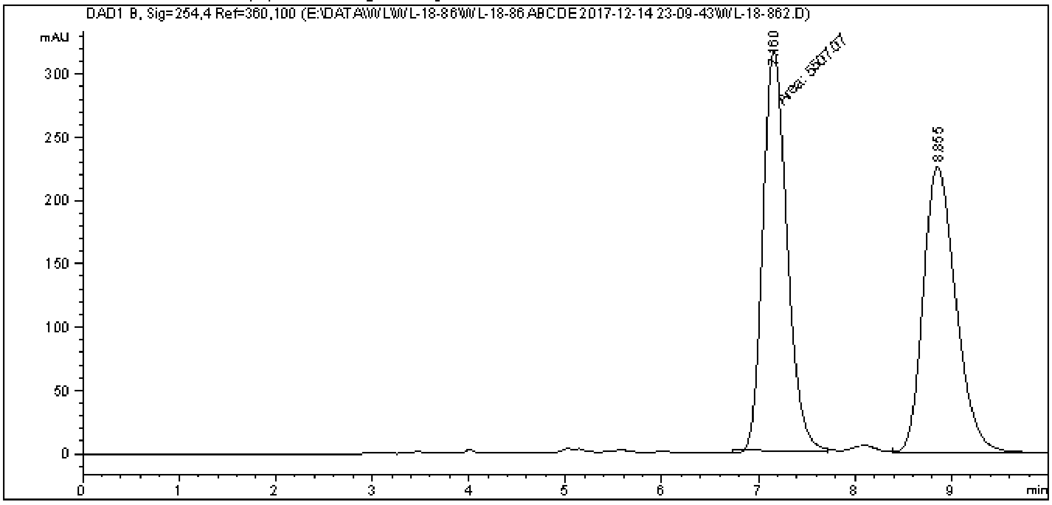


```
    =====================================================================
                             Area Percent Report
    =====================================================================

    Sorted By             :      Signal
    Multiplier            :      1.0000
    Dilution              :      1.0000
    Do not use Multiplier & Dilution Factor with ISTDs


    Signal 1: DAD1 B, Sig=254,4 Ref=360,100

    Peak RetTime Type  Width     Area      Height     Area
      #   [min]        [min]   [mAU*s]     [mAU]        %
    ----|-------|----|-------|----------|----------|--------|
       1   7.160 MM    0.2900 5507.07178  316.50110  50.9295
       2   8.855 BB    0.3599 5306.05078  226.00667  49.0705

    Totals :                  1.08131e4   542.50777


    =====================================================================
                             *** End of Report ***

1260 5/2/2018 8:26:42 PM SYSTEM                                        Page 1 of 1
```

Supplementary Figure 122. HPLC spectrum of (*rac*)-**3d**

Data File E:\DATA\WL\WL-18-86\WL-18-86ABCDE 2017-12-14 23-09-43\WL-18-861.D
Sample Name: WL-18-86B-opt

===

Acq. Operator : SYSTEM Seq. Line : 2
Acq. Instrument : 1260 Location : 22
Injection Date : 12/14/2017 11:27:26 PM Inj : 1
Inj Volume : 5.000 µl
Acq. Method : E:\DATA\WL\WL-18-86\WL-18-86ABCDE 2017-12-14 23-09-43\ODH-90-10-1.0ML-ALL-254NM-30MIN.M
Last changed : 12/14/2017 11:10:01 PM by SYSTEM
Analysis Method : E:\DATA\WL\WL-18-86\WL-18-86ABCDE 2017-12-14 23-09-43\ODH-90-10-1.0ML-ALL-254NM-30MIN.M (Sequence Method)
Last changed : 5/2/2018 8:26:38 PM by SYSTEM
(modified after loading)
Additional Info : Peak(s) manually integrated

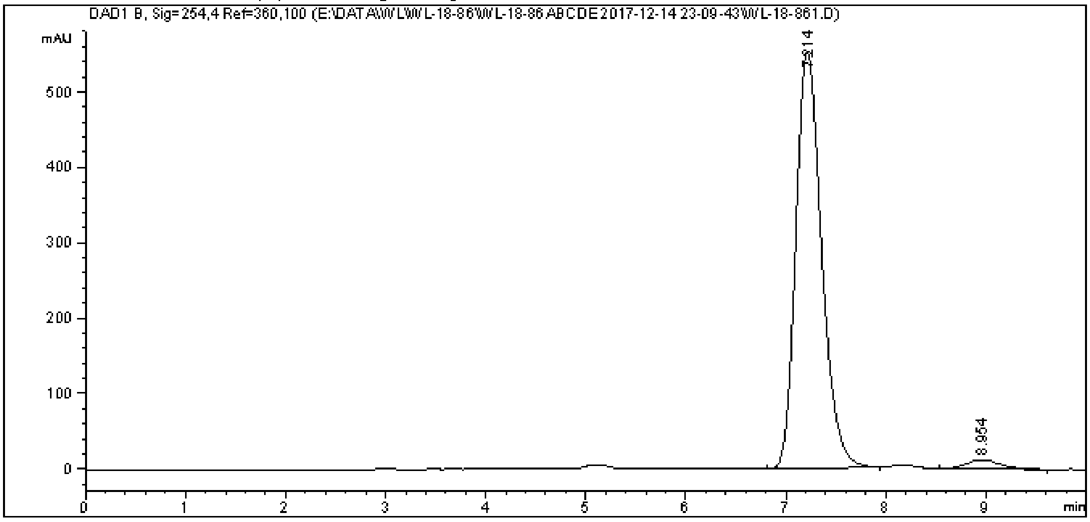

===

Area Percent Report

===

Sorted By : Signal
Multiplier : 1.0000
Dilution : 1.0000
Do not use Multiplier & Dilution Factor with ISTDs

Signal 1: DAD1 B, Sig=254,4 Ref=360,100

Peak #	RetTime [min]	Type	Width [min]	Area [mAU*s]	Height [mAU]	Area %
1	7.214	BB	0.2763	9911.16504	552.36609	97.2585
2	8.954	BB	0.3391	279.37354	12.11145	2.7415

Totals : 1.01905e4 564.47754

===

*** End of Report ***

1260 5/2/2018 8:27:07 PM SYSTEM Page 1 of 1

Supplementary Figure 123. HPLC spectrum of (*S*)-**3d**

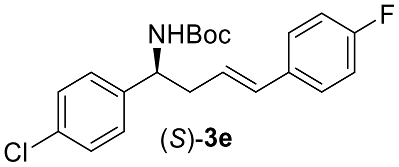

(*S*)-**3e**

Data File E:\DATA\WL\WL-18-86\WL-18-86ABCDE 2017-12-14 23-09-43\WL-18-865.D
Sample Name: WL-18-86E-rac

===

Acq. Operator : SYSTEM Seq. Line : 6
Acq. Instrument : 1260 Location : 26
Injection Date : 12/15/2017 12:33:13 AM Inj : 1
Inj Volume : 5.000 µl
Acq. Method : E:\DATA\WL\WL-18-86\WL-18-86ABCDE 2017-12-14 23-09-43\ODH-90-10-1.0ML-ALL-254NM-30MIN.M
Last changed : 12/14/2017 11:10:01 PM by SYSTEM
Analysis Method : E:\DATA\WL\WL-18-86\WL-18-86ABCDE 2017-12-14 23-09-43\ODH-90-10-1.0ML-ALL-254NM-30MIN.M (Sequence Method)
Last changed : 5/2/2018 8:26:38 PM by SYSTEM
(modified after loading)
Additional Info : Peak(s) manually integrated

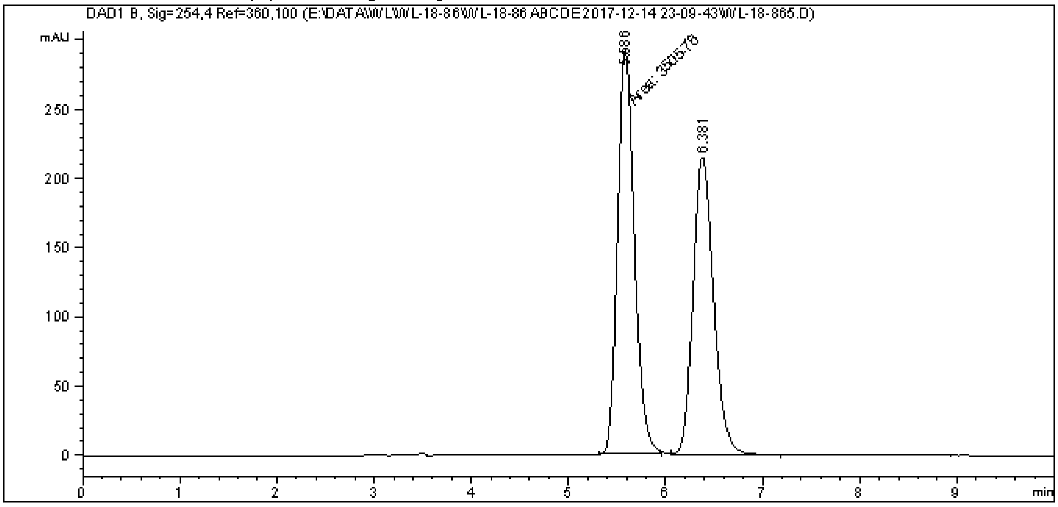

===

Area Percent Report

===

Sorted By : Signal
Multiplier : 1.0000
Dilution : 1.0000
Do not use Multiplier & Dilution Factor with ISTDs

Signal 1: DAD1 B, Sig=254,4 Ref=360,100

Peak #	RetTime [min]	Type	Width [min]	Area [mAU*s]	Height [mAU]	Area %
1	5.586	MM	0.2011	3505.75635	290.53647	52.4767
2	6.381	VB	0.2272	3174.84375	215.03854	47.5233

Totals : 6680.60010 505.57501

===

*** End of Report ***

1260 5/2/2018 8:29:29 PM SYSTEM Page 1 of 1

Supplementary Figure 124. HPLC spectrum of (*rac*)-**3e**

Data File E:\DATA\WL\WL-18-86\WL-18-86CDE 2017-12-26 12-02-46\WL-18-862.D
Sample Name: WL-18-86E-OPT

===

Acq. Operator : SYSTEM Seq. Line : 3
Acq. Instrument : 1260 Location : 3
Injection Date : 12/26/2017 1:06:54 PM Inj : 1
Inj Volume : 5.000 µl
Acq. Method : E:\DATA\WL\WL-18-86\WL-18-86CDE 2017-12-26 12-02-46\ODH-90-10-1.0ML-ALL-254NM-30MIN.M
Last changed : 12/26/2017 12:02:46 PM by SYSTEM
Analysis Method : E:\DATA\WL\WL-18-86\WL-18-86CDE 2017-12-26 12-02-46\ODH-90-10-1.0ML-ALL-254NM-30MIN.M (Sequence Method)
Last changed : 5/2/2018 8:30:34 PM by SYSTEM
(modified after loading)
Additional Info : Peak(s) manually integrated

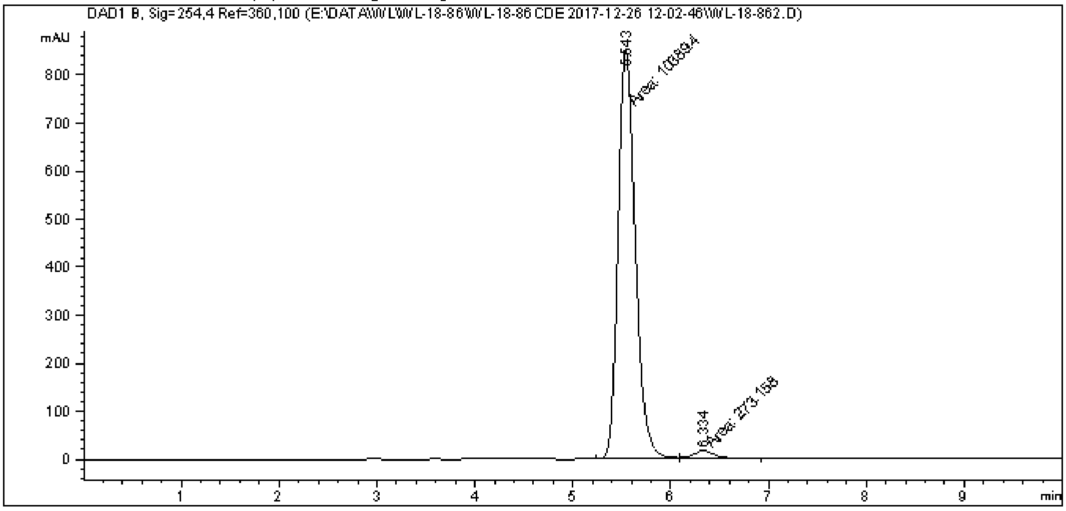

===

Area Percent Report

===

Sorted By : Signal
Multiplier : 1.0000
Dilution : 1.0000
Do not use Multiplier & Dilution Factor with ISTDs

Signal 1: DAD1 B, Sig=254,4 Ref=360,100

Peak #	RetTime [min]	Type	Width [min]	Area [mAU*s]	Height [mAU]	Area %
1	5.543	MF	0.2037	1.03894e4	850.18860	97.4382
2	6.334	FM	0.2716	273.15820	16.76173	2.5618

Totals : 1.06626e4 866.95033

===

*** End of Report ***

1260 5/2/2018 8:31:12 PM SYSTEM Page 1 of 1

Supplementary Figure 125. HPLC spectrum of (*S*)-**3e**

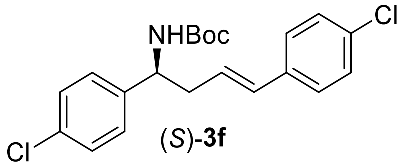

(*S*)-**3f**

```
Data File E:\DATA\WL\WL-18-86\WL-18-86ABCDE 2017-12-14 23-09-43\WL-18-864.D
Sample Name: WL-18-86D-rac

    =====================================================================
    Acq. Operator   : SYSTEM                        Seq. Line :    5
    Acq. Instrument : 1260                           Location :   25
    Injection Date  : 12/15/2017 12:16:48 AM              Inj :    1
                                                   Inj Volume : 5.000 µl
    Acq. Method     : E:\DATA\WL\WL-18-86\WL-18-86ABCDE 2017-12-14 23-09-43\ODH-90-10-1.0ML-ALL-
                      254NM-30MIN.M
    Last changed    : 12/14/2017 11:10:01 PM by SYSTEM
    Analysis Method : E:\DATA\WL\WL-18-86\WL-18-86ABCDE 2017-12-14 23-09-43\ODH-90-10-1.0ML-ALL-
                      254NM-30MIN.M (Sequence Method)
    Last changed    : 5/2/2018 8:26:38 PM by SYSTEM
                      (modified after loading)
    Additional Info : Peak(s) manually integrated
```

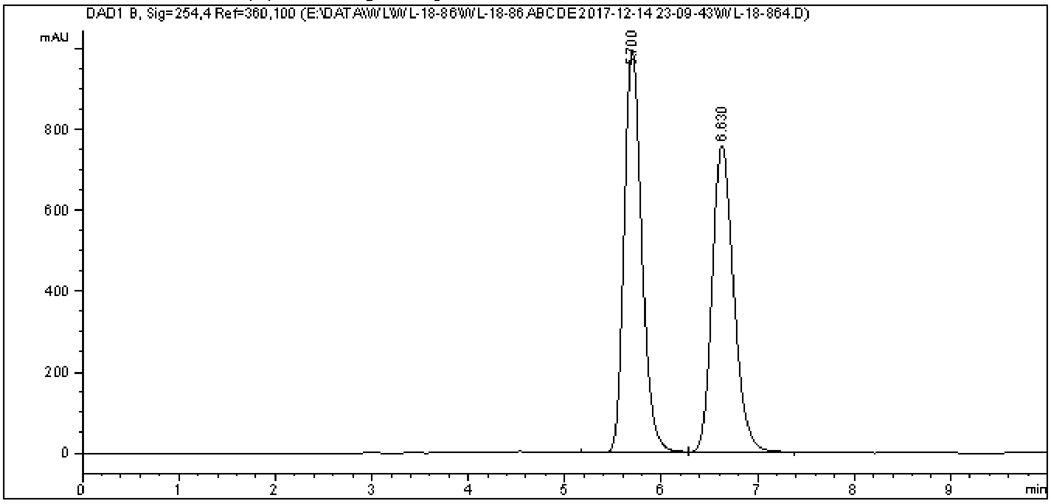


```
    =====================================================================
                             Area Percent Report
    =====================================================================

    Sorted By             :      Signal
    Multiplier            :      1.0000
    Dilution              :      1.0000
    Do not use Multiplier & Dilution Factor with ISTDs


    Signal 1: DAD1 B, Sig=254,4 Ref=360,100

    Peak RetTime Type  Width     Area      Height     Area
      #   [min]        [min]   [mAU*s]     [mAU]        %
    ----|-------|----|-------|----------|----------|--------|
       1   5.700 VB R  0.1960 1.26089e4   993.21906  51.8703
       2   6.630 BB    0.2393 1.16996e4   757.24414  48.1297

    Totals :                  2.43086e4  1750.46320


    =====================================================================
                             *** End of Report ***

1260 5/2/2018 8:28:48 PM SYSTEM                                      Page 1 of 1
```

Supplementary Figure 126. HPLC spectrum of (*rac*)-**3f**

```
Data File E:\DATA\WL\WL-18-86\WL-18-86CDE 2017-12-26 12-02-46\WL-18-861.D
Sample Name: WL-18-86D-OPT

    =====================================================================
    Acq. Operator   : SYSTEM                        Seq. Line :    2
    Acq. Instrument : 1260                           Location :    2
    Injection Date  : 12/26/2017 12:35:30 PM              Inj :    1
                                                   Inj Volume : 5.000 µl
    Acq. Method     : E:\DATA\WL\WL-18-86\WL-18-86CDE 2017-12-26 12-02-46\ODH-90-10-1.0ML-ALL-
                      254NM-30MIN.M
    Last changed    : 12/26/2017 12:02:46 PM by SYSTEM
    Analysis Method : E:\DATA\WL\WL-18-86\WL-18-86CDE 2017-12-26 12-02-46\ODH-90-10-1.0ML-ALL-
                      254NM-30MIN.M (Sequence Method)
    Last changed    : 5/2/2018 8:30:34 PM by SYSTEM
                      (modified after loading)
    Additional Info : Peak(s) manually integrated
```

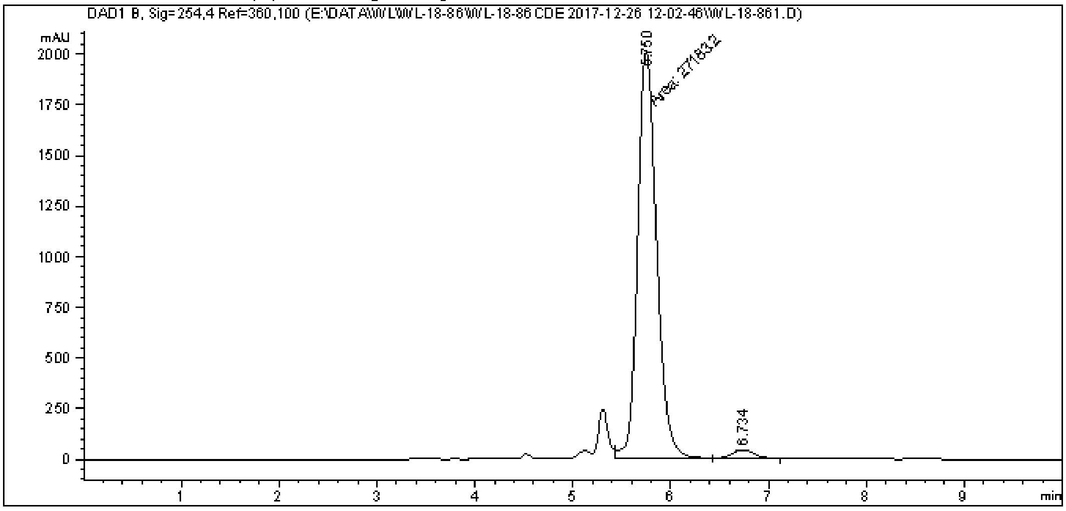


```
    =====================================================================
                             Area Percent Report
    =====================================================================

    Sorted By            :      Signal
    Multiplier           :      1.0000
    Dilution             :      1.0000
    Do not use Multiplier & Dilution Factor with ISTDs


    Signal 1: DAD1 B, Sig=254,4 Ref=360,100

    Peak RetTime Type  Width     Area      Height     Area
      #   [min]        [min]   [mAU*s]     [mAU]        %
    ----|-------|----|-------|----------|----------|--------|
       1   5.750 FM    0.2249 2.71832e4  2014.48145  97.6034
       2   6.734 BB    0.2413  667.47791   42.70662   2.3966

    Totals :                  2.78507e4  2057.18806


    =====================================================================
                              *** End of Report ***

1260 5/2/2018 8:31:01 PM SYSTEM                                          Page 1 of 1
```

Supplementary Figure 127. HPLC spectrum of (*S*)-**3f**

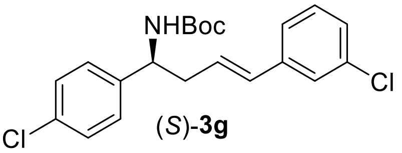

(*S*)-**3g**

Data File E:\DATA\WL\WL-18-88\WL-18-88B-RAC 2017-12-22 00-31-32\WL-18-882.D
Sample Name: WL-18-88C-RAC

===

Acq. Operator : SYSTEM Seq. Line : 3
Acq. Instrument : 1260 Location : 9
Injection Date : 12/22/2017 1:06:10 AM Inj : 1
Inj Volume : 5.000 µl
Acq. Method : E:\DATA\WL\WL-18-88\WL-18-88B-RAC 2017-12-22 00-31-32\ADH-90-10-1.0ML-ALL-210NM-30MIN.M
Last changed : 12/22/2017 12:48:10 AM by SYSTEM
Analysis Method : E:\DATA\WL\WL-18-88\WL-18-88B-RAC 2017-12-22 00-31-32\ADH-90-10-1.0ML-ALL-210NM-30MIN.M (Sequence Method)
Last changed : 5/2/2018 9:03:14 PM by SYSTEM
(modified after loading)
Additional Info : Peak(s) manually integrated

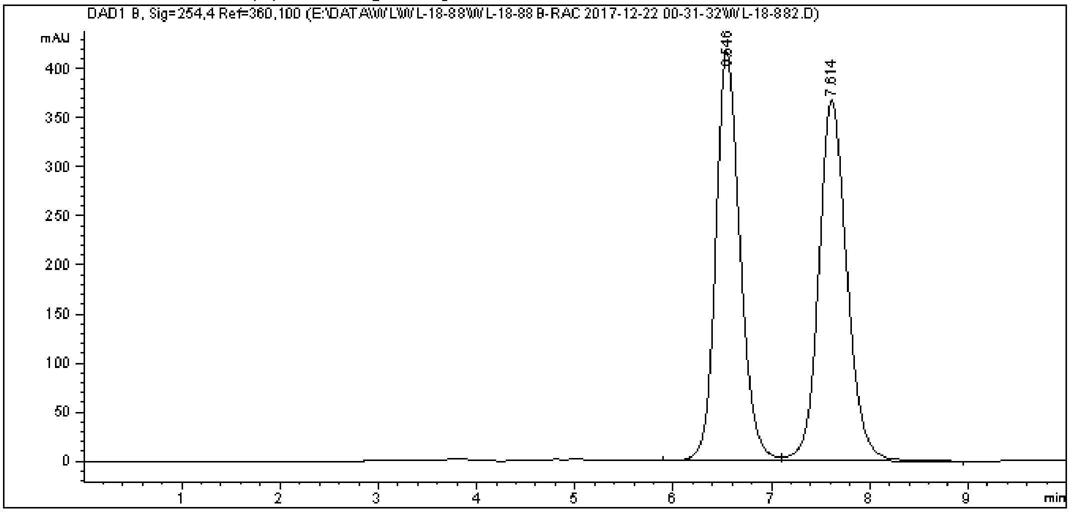

===

Area Percent Report

===

Sorted By : Signal
Multiplier : 1.0000
Dilution : 1.0000
Do not use Multiplier & Dilution Factor with ISTDs

Signal 1: DAD1 B, Sig=254,4 Ref=360,100

Peak #	RetTime [min]	Type	Width [min]	Area [mAU*s]	Height [mAU]	Area %
1	6.546	BV	0.2636	7193.07422	418.37674	49.4998
2	7.614	VB	0.3044	7338.44580	368.40784	50.5002
Totals :				1.45315e4	786.78458	

===

*** End of Report ***

1260 5/2/2018 9:03:28 PM SYSTEM Page 1 of 1

Supplementary Figure 128. HPLC spectrum of (*rac*)-**3g**

Data File E:\DATA\WL\WL-18-86\WL-18-86FH-OPT 2017-12-29 16-19-14\WL-18-86.D
Sample Name: WL-18-86F-OPT

===

Acq. Operator : SYSTEM   Seq. Line : 1
Acq. Instrument : 1260   Location : 93
Injection Date : 12/29/2017 4:20:38 PM   Inj : 1
Inj Volume : 5.000 µl
Acq. Method : E:\DATA\WL\WL-18-86\WL-18-86FH-OPT 2017-12-29 16-19-14\ODH-90-10-1.0ML-ALL-254NM-30MIN.M
Last changed : 12/29/2017 4:19:26 PM by SYSTEM
(modified after loading)
Analysis Method : E:\DATA\WL\WL-18-86\WL-18-86FH-OPT 2017-12-29 16-19-14\ODH-90-10-1.0ML-ALL-254NM-30MIN.M (Sequence Method)
Last changed : 5/2/2018 8:35:45 PM by SYSTEM
(modified after loading)
Additional Info : Peak(s) manually integrated

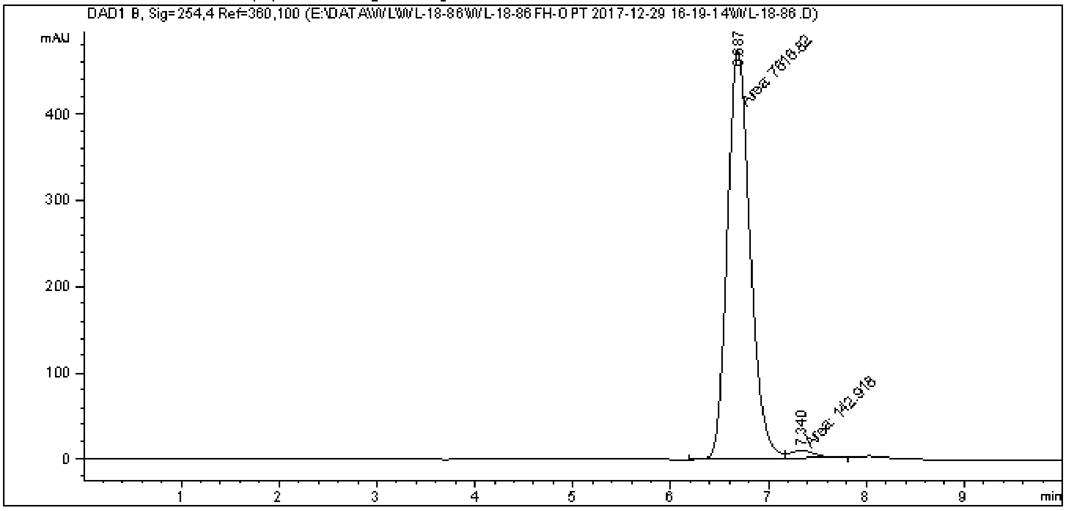

===

Area Percent Report

===

Sorted By : Signal
Multiplier : 1.0000
Dilution : 1.0000
Do not use Multiplier & Dilution Factor with ISTDs

Signal 1: DAD1 B, Sig=254,4 Ref=360,100

Peak #	RetTime [min]	Type	Width [min]	Area [mAU*s]	Height [mAU]	Area %
1	6.687	MF	0.2686	7616.81934	472.62332	98.1582
2	7.340	FM	0.2900	142.91833	8.21292	1.8418

Totals : 7759.73767 480.83624

===

1260 5/2/2018 8:35:48 PM SYSTEM   Page 1 of 2

Supplementary Figure 129. HPLC spectrum of (*S*)-**3g**

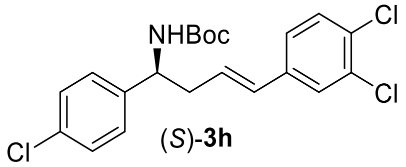

Data File E:\DATA\WL\WL-18-86\WL-18-86A 2017-12-11 21-48-16\WL-18-86.D
Sample Name: WL-18-86A

===

Acq. Operator : SYSTEM Seq. Line : 1
Acq. Instrument : 1260 Location : 49
Injection Date : 12/11/2017 9:49:42 PM Inj : 1
Inj Volume : 5.000 µl
Acq. Method : E:\DATA\WL\WL-18-86\WL-18-86A 2017-12-11 21-48-16\ODH-90-10-1.0ML-ALL-254NM -30MIN.M
Last changed : 12/11/2017 9:48:16 PM by SYSTEM
Analysis Method : E:\DATA\WL\WL-18-86\WL-18-86A 2017-12-11 21-48-16\ODH-90-10-1.0ML-ALL-254NM -30MIN.M (Sequence Method)
Last changed : 5/2/2018 8:23:24 PM by SYSTEM
(modified after loading)
Additional Info : Peak(s) manually integrated

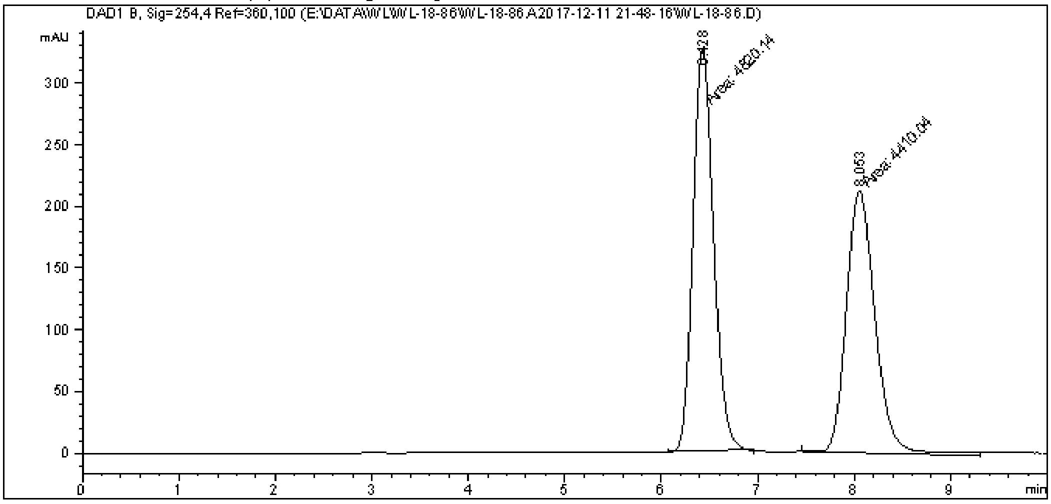

===

Area Percent Report

===

Sorted By : Signal
Multiplier : 1.0000
Dilution : 1.0000
Do not use Multiplier & Dilution Factor with ISTDs

Signal 1: DAD1 B, Sig=254,4 Ref=360,100

Peak #	RetTime [min]	Type	Width [min]	Area [mAU*s]	Height [mAU]	Area %
1	6.428	MM	0.2474	4820.14111	324.68561	52.2215
2	8.053	MM	0.3460	4410.04492	212.41692	47.7785

Totals : 9230.18604 537.10252

===

*** End of Report ***

1260 5/2/2018 8:23:29 PM SYSTEM Page 1 of 1

Supplementary Figure 130. HPLC spectrum of (*rac*)-**3h**

Data File E:\DATA\WL\WL-18-86\WL-18-86ABCDE 2017-12-14 23-09-43\WL-18-86.D
Sample Name: WL-18-86A-opt

===

Acq. Operator : SYSTEM Seq. Line : 1
Acq. Instrument : 1260 Location : 21
Injection Date : 12/14/2017 11:11:04 PM Inj : 1
Inj Volume : 5.000 µl
Acq. Method : E:\DATA\WL\WL-18-86\WL-18-86ABCDE 2017-12-14 23-09-43\ODH-90-10-1.0ML-ALL-254NM-30MIN.M
Last changed : 12/14/2017 11:10:01 PM by SYSTEM
(modified after loading)
Analysis Method : E:\DATA\WL\WL-18-86\WL-18-86ABCDE 2017-12-14 23-09-43\ODH-90-10-1.0ML-ALL-254NM-30MIN.M (Sequence Method)
Last changed : 5/2/2018 8:25:52 PM by SYSTEM
(modified after loading)
Additional Info : Peak(s) manually integrated

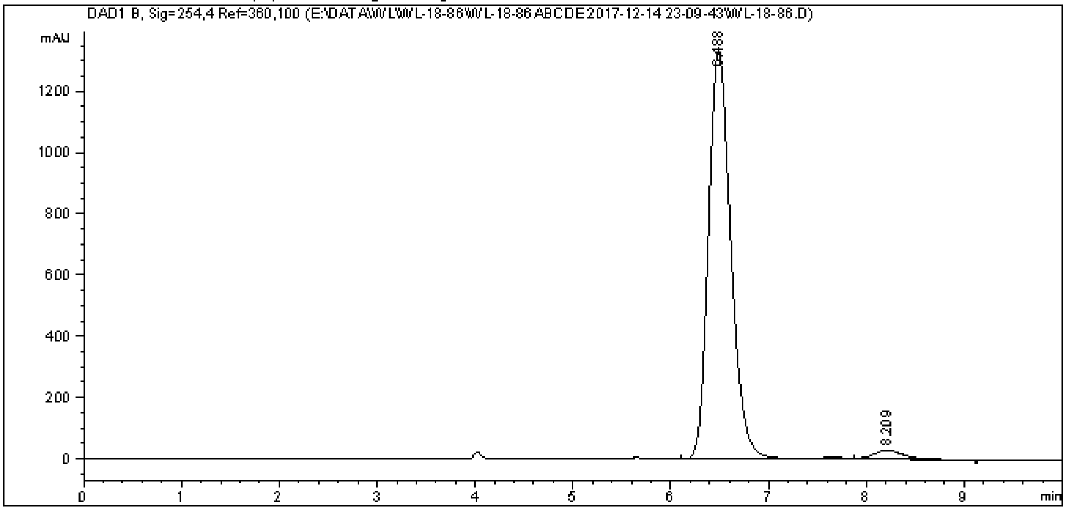

===

Area Percent Report

===

Sorted By : Signal
Multiplier : 1.0000
Dilution : 1.0000
Do not use Multiplier & Dilution Factor with ISTDs

Signal 1: DAD1 B, Sig=254,4 Ref=360,100

Peak #	RetTime [min]	Type	Width [min]	Area [mAU*s]	Height [mAU]	Area %
1	6.488	BV R	0.2438	2.11906e4	1332.72754	97.2753
2	8.209	VB E	0.3253	593.55768	28.12059	2.7247

Totals : 2.17841e4 1360.84813

===

1260 5/2/2018 8:25:55 PM SYSTEM Page 1 of 2

Supplementary Figure 131. HPLC spectrum of (*S*)-**3h**

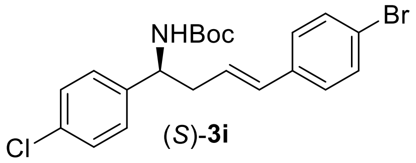

(*S*)-**3i**

```
Data File E:\DATA\WL\WL-18-86\WL-18-86ABCDE 2017-12-14 23-09-43\WL-18-863.D
Sample Name: WL-18-86C-rac

    =================================================================
    Acq. Operator   : SYSTEM                      Seq. Line :   4
    Acq. Instrument : 1260                          Location :   24
    Injection Date  : 12/15/2017 12:00:17 AM             Inj :   1
                                               Inj Volume : 5.000 µl
    Acq. Method     : E:\DATA\WL\WL-18-86\WL-18-86ABCDE 2017-12-14 23-09-43\ODH-90-10-1.0ML-ALL-
                      254NM-30MIN.M
    Last changed    : 12/14/2017 11:10:01 PM by SYSTEM
    Analysis Method : E:\DATA\WL\WL-18-86\WL-18-86ABCDE 2017-12-14 23-09-43\ODH-90-10-1.0ML-ALL-
                      254NM-30MIN.M (Sequence Method)
    Last changed    : 5/2/2018 8:26:38 PM by SYSTEM
                      (modified after loading)
    Additional Info : Peak(s) manually integrated
```

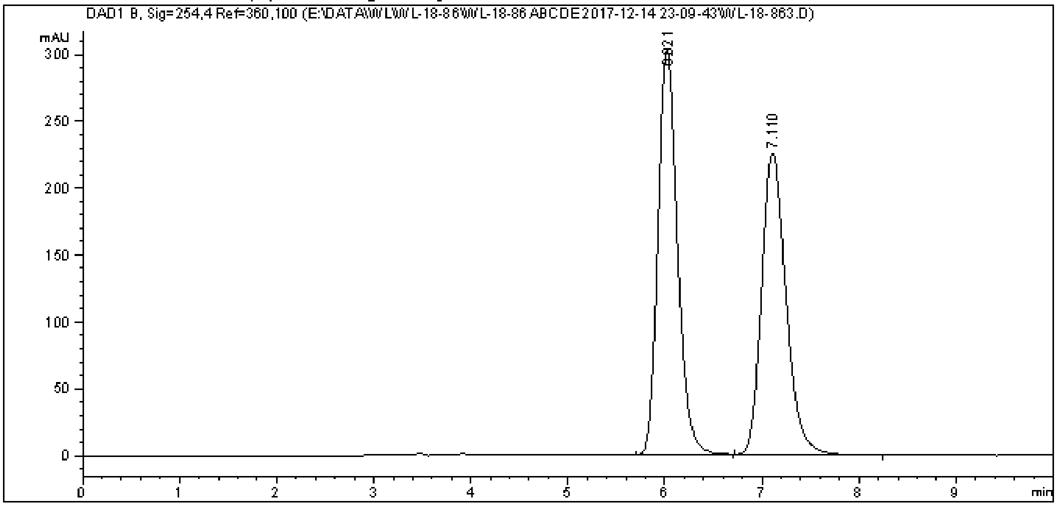


```
    =================================================================
                          Area Percent Report
    =================================================================

    Sorted By             :      Signal
    Multiplier            :      1.0000
    Dilution              :      1.0000
    Do not use Multiplier & Dilution Factor with ISTDs


    Signal 1: DAD1 B, Sig=254,4 Ref=360,100

    Peak RetTime Type  Width     Area      Height     Area
      #   [min]        [min]   [mAU*s]     [mAU]        %
    ----|-------|----|-------|----------|----------|--------|
       1   6.021 BB    0.2164 4234.55371  302.29507  51.7415
       2   7.110 BB    0.2677 3949.50415  226.20853  48.2585

    Totals :                  8184.05786  528.50360


    =================================================================
                          *** End of Report ***
```


Supplementary Figure 132. HPLC spectrum of (*rac*)-**3i**

Data File E:\DATA\WL\WL-18-86\WL-18-86CDE 2017-12-26 12-02-46\WL-18-86.D
Sample Name: WL-18-86C-OPT

===

Acq. Operator : SYSTEM   Seq. Line : 1
Acq. Instrument : 1260   Location : 1
Injection Date : 12/26/2017 12:04:08 PM   Inj : 1
Inj Volume : 5.000 µl
Acq. Method : E:\DATA\WL\WL-18-86\WL-18-86CDE 2017-12-26 12-02-46\ODH-90-10-1.0ML-ALL-254NM-30MIN.M
Last changed : 12/26/2017 12:02:46 PM by SYSTEM
Analysis Method : E:\DATA\WL\WL-18-86\WL-18-86CDE 2017-12-26 12-02-46\ODH-90-10-1.0ML-ALL-254NM-30MIN.M (Sequence Method)
Last changed : 5/2/2018 8:30:34 PM by SYSTEM
(modified after loading)
Additional Info : Peak(s) manually integrated

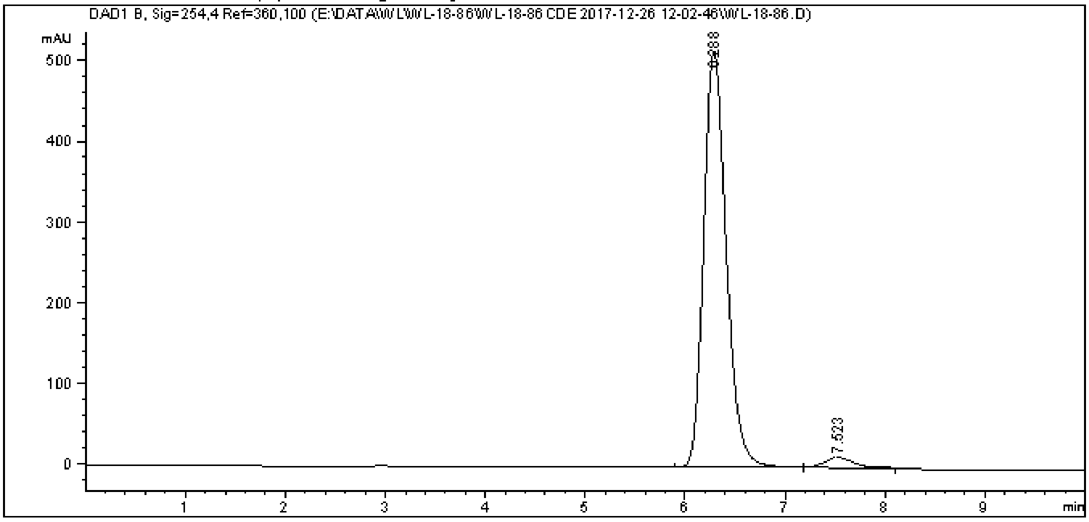

===

Area Percent Report

===

Sorted By : Signal
Multiplier : 1.0000
Dilution : 1.0000
Do not use Multiplier & Dilution Factor with ISTDs

Signal 1: DAD1 B, Sig=254,4 Ref=360,100

Peak #	RetTime [min]	Type	Width [min]	Area [mAU*s]	Height [mAU]	Area %
1	6.288	BB	0.2418	8052.49463	514.00195	96.7657
2	7.523	BB	0.3190	269.14597	12.60950	3.2343

Totals : 8321.64059 526.61145

===

*** End of Report ***

1260 5/2/2018 8:30:39 PM SYSTEM   Page 1 of 1

Supplementary Figure 133. HPLC spectrum of (*S*)-**3i**

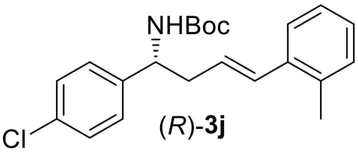

(*R*)-**3j**

Data File E:\DATA\ZQ\ZQ-10-59\ZQ-10-59 2018-01-18 14-58-07\SLM-8-371.D
Sample Name: ZQ-10-58-o-CH3-rac

```
=====================================================================
Acq. Operator   : SYSTEM                        Seq. Line :   2
Acq. Instrument : 1260                           Location :   74
Injection Date  : 1/18/2018 3:31:03 PM                Inj :   1
                                               Inj Volume : 5.000 µl
Acq. Method     : E:\DATA\ZQ\ZQ-10-59\ZQ-10-59 2018-01-18 14-58-07\OD-90-10-254NM-30MIN.M
Last changed    : 1/18/2018 3:41:36 PM by SYSTEM
                  (modified after loading)
Analysis Method : E:\DATA\ZQ\ZQ-10-59\ZQ-10-59 2018-01-18 14-58-07\OD-90-10-254NM-30MIN.M (
                  Sequence Method)
Last changed    : 4/11/2018 2:56:53 PM by SYSTEM
                  (modified after loading)
Additional Info : Peak(s) manually integrated
```

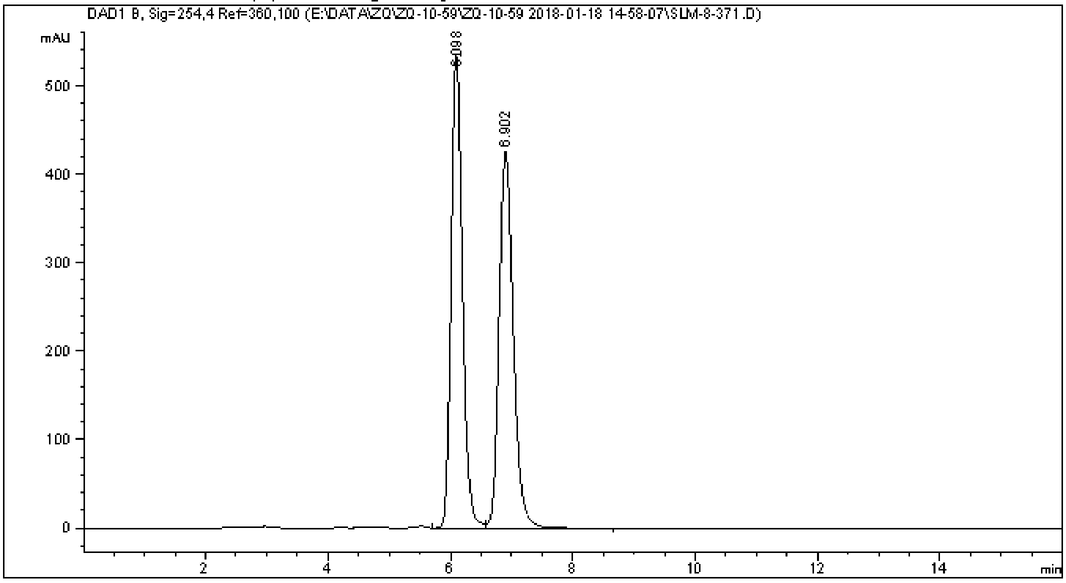


```
=====================================================================
                         Area Percent Report
=====================================================================

Sorted By            :      Signal
Multiplier           :      1.0000
Dilution             :      1.0000
Do not use Multiplier & Dilution Factor with ISTDs


Signal 1: DAD1 B, Sig=254,4 Ref=360,100

Peak RetTime Type  Width     Area      Height     Area
  #   [min]        [min]   [mAU*s]     [mAU]        %
----|-------|----|-------|----------|----------|--------|
   1   6.098 BV    0.1974 6897.25879  535.13696  50.1463
   2   6.902 VB    0.2456 6857.00488  426.39203  49.8537

Totals :                  1.37543e4   961.52899
```

1260 4/11/2018 2:56:57 PM SYSTEM Page 1 of 2

Supplementary Figure 134. HPLC spectrum of (*rac*)-**3j**

Data File E:\DATA\ZQ\ZQ-10-59\ZQ-10-59 2018-01-18 14-58-07\SLM-8-373.D
Sample Name: ZQ-10-58-o-CH3-You

===

Acq. Operator : SYSTEM Seq. Line : 4
Acq. Instrument : 1260 Location : 78
Injection Date : 1/18/2018 4:11:01 PM Inj : 1
Inj Volume : 5.000 µl
Acq. Method : E:\DATA\ZQ\ZQ-10-59\ZQ-10-59 2018-01-18 14-58-07\OD-90-10-254NM-30MIN.M
Last changed : 1/18/2018 4:22:33 PM by SYSTEM
(modified after loading)
Analysis Method : E:\DATA\ZQ\ZQ-10-59\ZQ-10-59 2018-01-18 14-58-07\OD-90-10-254NM-30MIN.M (
Sequence Method)
Last changed : 4/11/2018 2:56:53 PM by SYSTEM
(modified after loading)
Additional Info : Peak(s) manually integrated

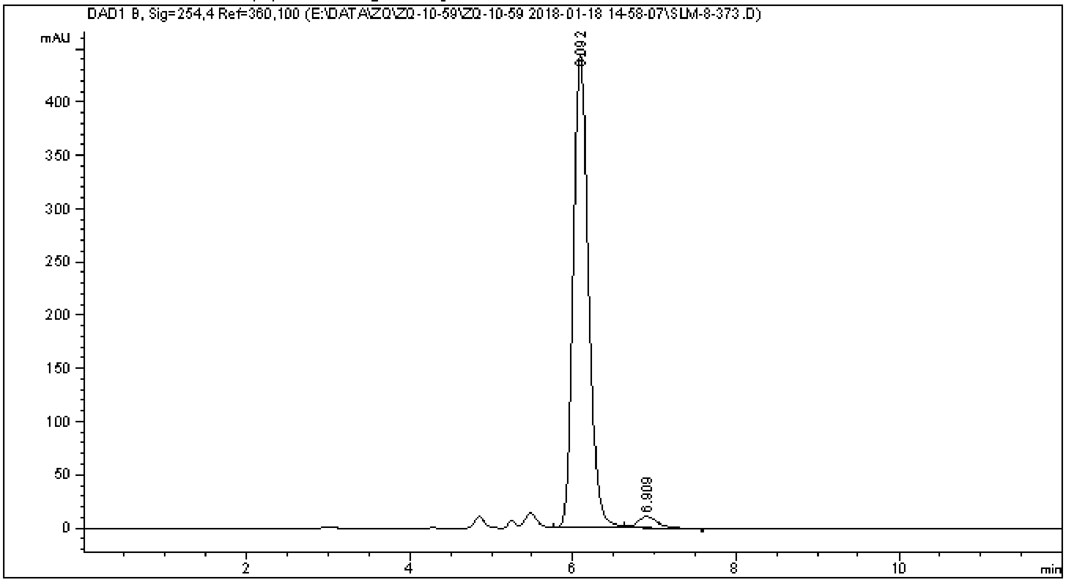

===

Area Percent Report

===

Sorted By : Signal
Multiplier : 1.0000
Dilution : 1.0000
Do not use Multiplier & Dilution Factor with ISTDs

Signal 1: DAD1 B, Sig=254,4 Ref=360,100

Peak #	RetTime [min]	Type	Width [min]	Area [mAU*s]	Height [mAU]	Area %
1	6.092	BV R	0.1997	5778.07129	444.45648	97.1714
2	6.909	VB E	0.2406	168.19788	10.57043	2.8286

Totals : 5946.26917 455.02691

1260 4/11/2018 2:58:22 PM SYSTEM Page 1 of 2

Supplementary Figure 135. HPLC spectrum of (*R*)-**3j**

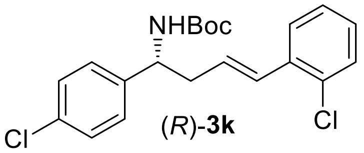

(*R*)-**3k**

Data File E:\DATA\ZQ\ZQ-10-67\ZQ-10-67 2018-01-25 12-10-41\SC-7-109.D
Sample Name: ZQ-10-67-rac

===

Acq. Operator : SYSTEM Seq. Line : 1
Acq. Instrument : 1260 Location : 64
Injection Date : 1/25/2018 12:12:06 PM Inj : 1
Inj Volume : 1.000 µl
Acq. Method : E:\DATA\ZQ\ZQ-10-67\ZQ-10-67 2018-01-25 12-10-41\IE-95-5-254NM-1uL-20MIN.M
Last changed : 1/25/2018 12:10:41 PM by SYSTEM
Analysis Method : E:\DATA\ZQ\ZQ-10-67\ZQ-10-67 2018-01-25 12-10-41\IE-95-5-254NM-1uL-20MIN.M
(Sequence Method)
Last changed : 4/11/2018 3:03:37 PM by SYSTEM
(modified after loading)

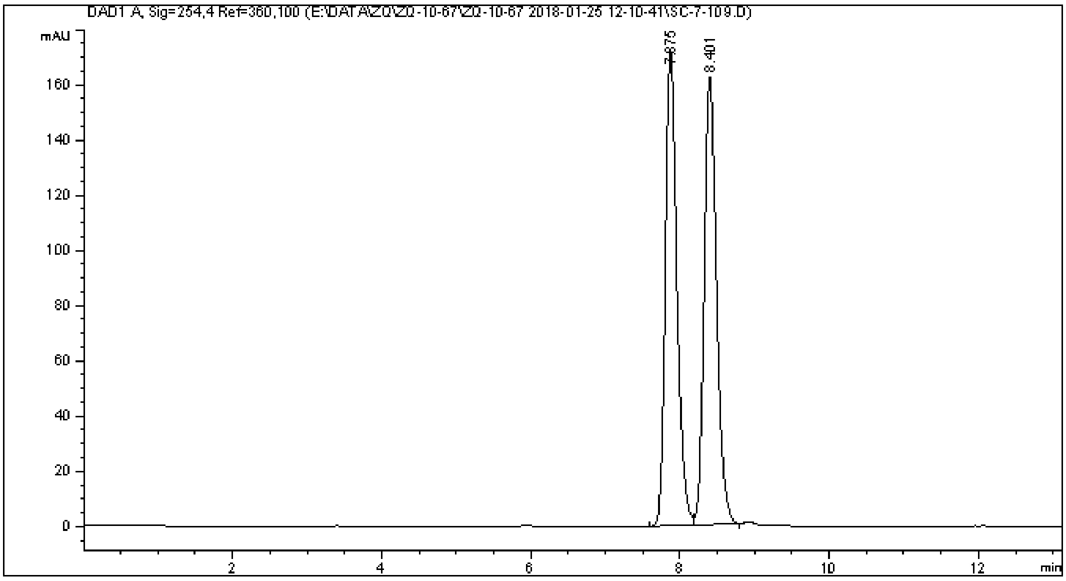

===

Area Percent Report

===

Sorted By : Signal
Multiplier : 1.0000
Dilution : 1.0000
Do not use Multiplier & Dilution Factor with ISTDs

Signal 1: DAD1 A, Sig=254,4 Ref=360,100

Peak #	RetTime [min]	Type	Width [min]	Area [mAU*s]	Height [mAU]	Area %
1	7.875	BV	0.1643	1856.25244	171.40465	49.8435
2	8.401	VB	0.1753	1867.90637	162.29251	50.1565

Totals : 3724.15881 333.69716

===

1260 4/11/2018 3:03:51 PM SYSTEM Page 1 of 2

Supplementary Figure 136. HPLC spectrum of (*rac*)-**3k**

```
Data File E:\DATA\ZQ\ZQ-10-67\ZQ-10-67 2018-01-26 22-49-40\CC-4-17B-OPT.D
Sample Name: ZQ-10-67-opt

    =====================================================================
    Acq. Operator   : SYSTEM                        Seq. Line :   1
    Acq. Instrument : 1260                           Location :   65
    Injection Date  : 1/26/2018 10:51:12 PM               Inj :   1
                                                   Inj Volume : 1.000 µl
    Acq. Method     : E:\DATA\ZQ\ZQ-10-67\ZQ-10-67 2018-01-26 22-49-40\IE-95-5-254NM-1uL-20MIN.M
    Last changed    : 1/26/2018 10:49:40 PM by SYSTEM
    Analysis Method : E:\DATA\ZQ\ZQ-10-67\ZQ-10-67 2018-01-26 22-49-40\IE-95-5-254NM-1uL-20MIN.M
                      (Sequence Method)
    Last changed    : 4/11/2018 3:06:09 PM by SYSTEM
                      (modified after loading)
```

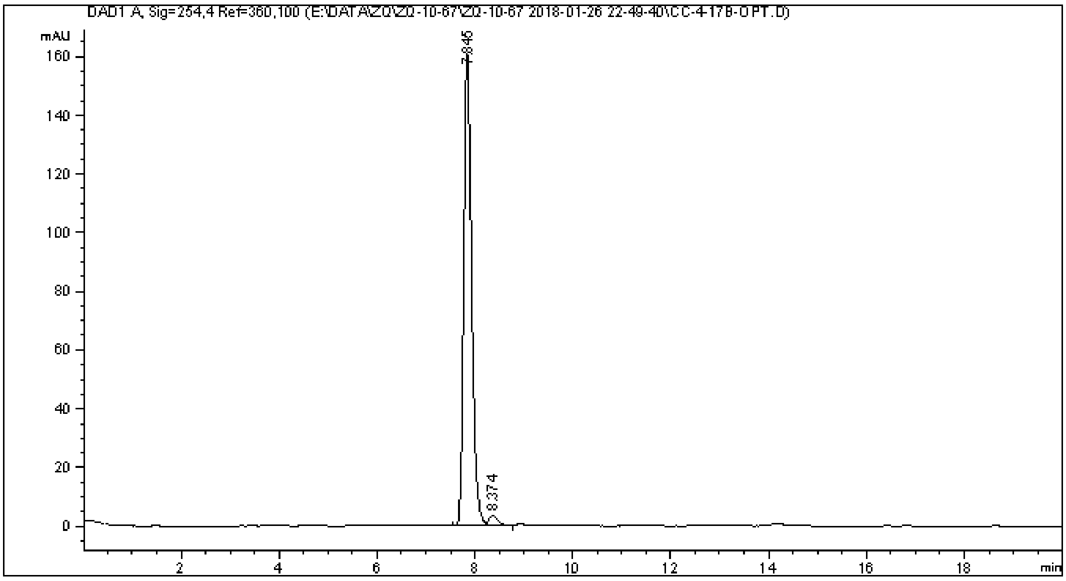


```
    =====================================================================
                             Area Percent Report
    =====================================================================

    Sorted By             :      Signal
    Multiplier            :      1.0000
    Dilution              :      1.0000
    Do not use Multiplier & Dilution Factor with ISTDs


    Signal 1: DAD1 A, Sig=254,4 Ref=360,100

    Peak RetTime Type  Width     Area      Height     Area
      #   [min]        [min]   [mAU*s]     [mAU]        %
    ----|-------|----|-------|----------|----------|--------|
       1   7.845 BV R  0.1679 1780.76160  161.15404  97.4784
       2   8.374 VB E  0.1981   46.06591    3.44396   2.5216

    Totals :                  1826.82751  164.59800


    =====================================================================
```

1260 4/11/2018 3:06:13 PM SYSTEM Page 1 of 2

Supplementary Figure 137. HPLC spectrum of (*R*)-**3k**

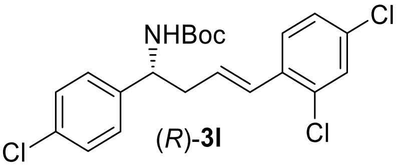

(*R*)-**3l**

Data File E:\DATA\ZQ\ZQ-10-66\ZQ-10-66 2018-01-25 14-09-54\SC-7-109.D
Sample Name: ZQ-10-66-rac

===
Acq. Operator : SYSTEM Seq. Line : 1
Acq. Instrument : 1260 Location : 63
Injection Date : 1/25/2018 2:11:17 PM Inj : 1
Inj Volume : 1.000 µl
Acq. Method : E:\DATA\ZQ\ZQ-10-66\ZQ-10-66 2018-01-25 14-09-54\OD-90-10-254NM-10MIN-1uL.M
Last changed : 1/25/2018 2:09:54 PM by SYSTEM
Analysis Method : E:\DATA\ZQ\ZQ-10-66\ZQ-10-66 2018-01-25 14-09-54\OD-90-10-254NM-10MIN-1uL.M
(Sequence Method)
Last changed : 4/11/2018 3:07:17 PM by SYSTEM
(modified after loading)

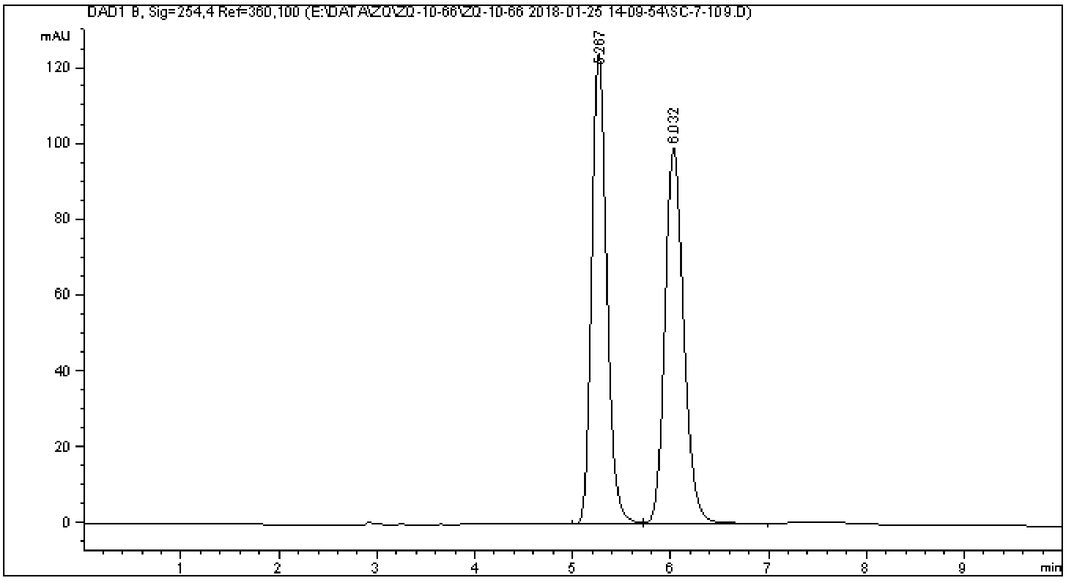

===
Area Percent Report
===

Sorted By : Signal
Multiplier : 1.0000
Dilution : 1.0000
Do not use Multiplier & Dilution Factor with ISTDs

Signal 1: DAD1 B, Sig=254,4 Ref=360,100

Peak #	RetTime [min]	Type	Width [min]	Area [mAU*s]	Height [mAU]	Area %
1	5.267	BV	0.1651	1332.84814	124.26910	50.0070
2	6.032	VB	0.2069	1332.47253	99.11027	49.9930

Totals : 2665.32068 223.37936

===

1260 4/11/2018 3:07:21 PM SYSTEM Page 1 of 2

Supplementary Figure 138. HPLC spectrum of (*rac*)-**3l**

Data File E:\DATA\ZQ\ZQ-10-66\ZQ-10-66 2018-01-25 14-09-54\SC-7-1091.D
Sample Name: ZQ-10-66-opt

```
=====================================================================
Acq. Operator   : SYSTEM                        Seq. Line :   2
Acq. Instrument : 1260                           Location :   65
Injection Date  : 1/25/2018 2:22:45 PM                Inj :   1
                                               Inj Volume : 1.000 µl
Acq. Method     : E:\DATA\ZQ\ZQ-10-66\ZQ-10-66 2018-01-25 14-09-54\OD-90-10-254NM-10MIN-1uL.M
Last changed    : 1/25/2018 2:09:54 PM by SYSTEM
Analysis Method : E:\DATA\ZQ\ZQ-10-66\ZQ-10-66 2018-01-25 14-09-54\OD-90-10-254NM-10MIN-1uL.M
                  (Sequence Method)
Last changed    : 4/11/2018 3:09:01 PM by SYSTEM
                  (modified after loading)
Additional Info : Peak(s) manually integrated
```

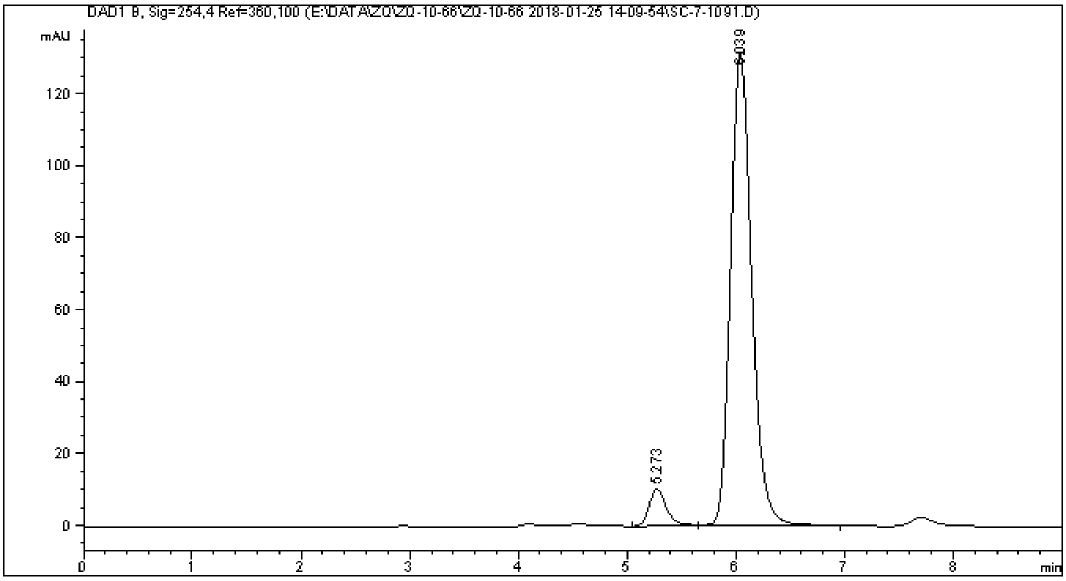


```
=====================================================================
                         Area Percent Report
=====================================================================

Sorted By             :      Signal
Multiplier            :      1.0000
Dilution              :      1.0000
Do not use Multiplier & Dilution Factor with ISTDs


Signal 1: DAD1 B, Sig=254,4 Ref=360,100

Peak RetTime Type  Width     Area      Height     Area
  #   [min]        [min]   [mAU*s]     [mAU]        %
----|-------|----|-------|----------|----------|--------|
   1   5.273 BB    0.1660  112.10761   10.29635   5.9435
   2   6.039 BB    0.2075 1774.12268  131.44487  94.0565

Totals :                  1886.23029  141.74122
```


Supplementary Figure 139. HPLC spectrum of (*R*)-**3l**

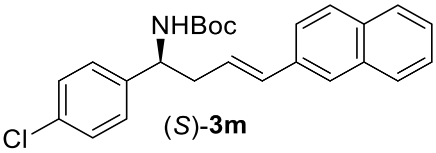

(*S*)-**3m**

```
Data File E:\DATA\ZQ\ZQ-10-38\ZQ-10-38 2017-12-27 15-50-03\cc-3-46a-opt.D
Sample Name: ZQ-10-38-nap-rac

    =====================================================================
    Acq. Operator   : SYSTEM                        Seq. Line :    1
    Acq. Instrument : 1260                           Location :   73
    Injection Date  : 12/27/2017 3:51:23 PM               Inj :    1
                                                   Inj Volume : 5.000 µl
    Acq. Method     : E:\DATA\ZQ\ZQ-10-38\ZQ-10-38 2017-12-27 15-50-03\AD-90-10-254NM-20MIN.M
    Last changed    : 12/27/2017 4:12:09 PM by SYSTEM
                      (modified after loading)
    Analysis Method : E:\DATA\ZQ\ZQ-10-38\ZQ-10-38 2017-12-27 15-50-03\AD-90-10-254NM-20MIN.M (
                      Sequence Method)
    Last changed    : 4/11/2018 2:08:17 PM by SYSTEM
                      (modified after loading)
    Additional Info : Peak(s) manually integrated
```

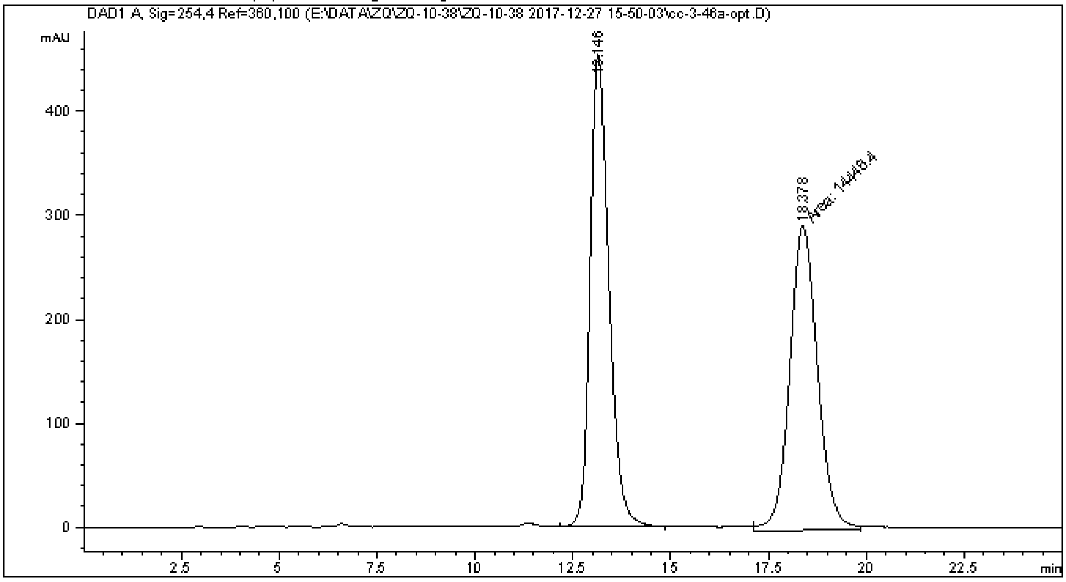


```
    =====================================================================
                             Area Percent Report
    =====================================================================

    Sorted By             :      Signal
    Multiplier            :      1.0000
    Dilution              :      1.0000
    Do not use Multiplier & Dilution Factor with ISTDs


    Signal 1: DAD1 A, Sig=254,4 Ref=360,100
```

Peak #	RetTime [min]	Type	Width [min]	Area [mAU*s]	Height [mAU]	Area %
1	13.146	BB	0.5073	1.51075e4	453.97009	51.1185
2	18.378	MM	0.8232	1.44464e4	292.49023	48.8815
Totals :				2.95539e4	746.46033	

Supplementary Figure 140. HPLC spectrum of (*rac*)-**3m**

Data File E:\DATA\ZQ\ZQ-10-38\ZQ-10-38 2017-12-27 16-23-25\cc-3-46a-opt.D
Sample Name: ZQ-10-38-nap-opt

```
=====================================================================
Acq. Operator   : SYSTEM                          Seq. Line :    1
Acq. Instrument : 1260                             Location :   74
Injection Date  : 12/27/2017 4:24:52 PM                 Inj :    1
                                                 Inj Volume : 5.000 µl
Acq. Method     : E:\DATA\ZQ\ZQ-10-38\ZQ-10-38 2017-12-27 16-23-25\AD-90-10-254NM-25MIN.M
Last changed    : 12/27/2017 4:23:25 PM by SYSTEM
Analysis Method : E:\DATA\ZQ\ZQ-10-38\ZQ-10-38 2017-12-27 16-23-25\AD-90-10-254NM-25MIN.M (
                  Sequence Method)
Last changed    : 4/11/2018 2:09:37 PM by SYSTEM
                  (modified after loading)
Additional Info : Peak(s) manually integrated
```

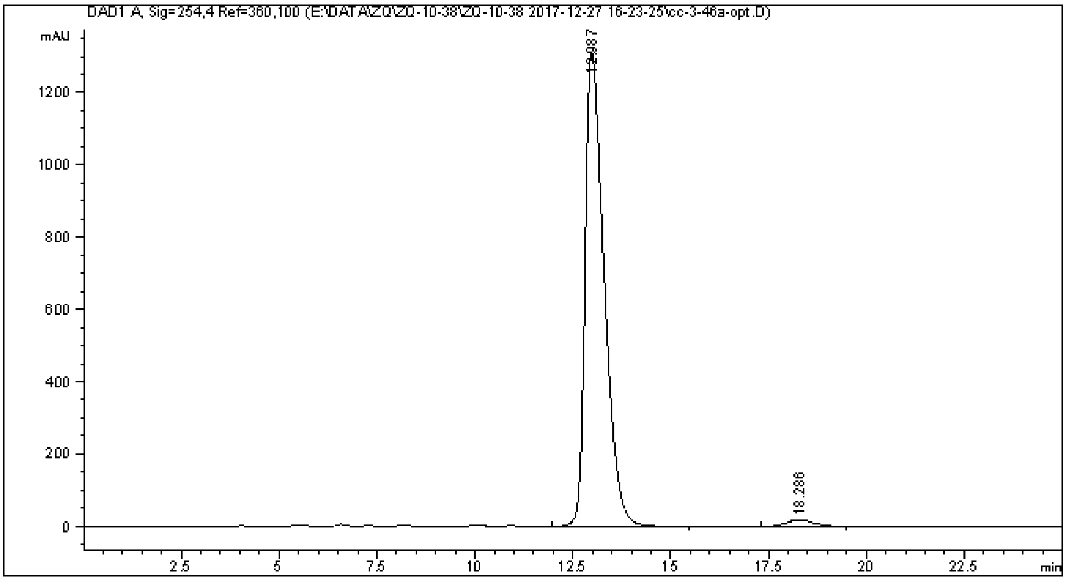


```
=====================================================================
                         Area Percent Report
=====================================================================

Sorted By             :      Signal
Multiplier            :      1.0000
Dilution              :      1.0000
Do not use Multiplier & Dilution Factor with ISTDs


Signal 1: DAD1 A, Sig=254,4 Ref=360,100

Peak RetTime Type  Width     Area      Height     Area
  #   [min]        [min]   [mAU*s]     [mAU]        %
----|-------|----|-------|----------|----------|--------|
   1  12.987 BB    0.5016 4.43058e4  1309.92896  98.2160
   2  18.286 BB    0.5532  804.75214   17.31563   1.7840

Totals :                  4.51106e4  1327.24459
```


Supplementary Figure 141. HPLC spectrum of (*S*)-**3m**

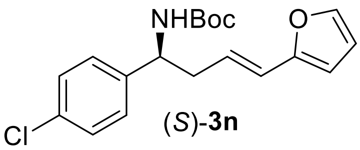

(*S*)-**3n**

```
Data File E:\DATA\WL\WL-18-86\WL-18-86HIJ-RAC 2017-12-19 17-16-59\WL-18-86.D
Sample Name: WL-18-86H-RAC

    ==================================================================
    Acq. Operator   : SYSTEM                          Seq. Line :   1
    Acq. Instrument : 1260                             Location :   31
    Injection Date  : 12/19/2017 5:18:27 PM                 Inj :   1
                                                     Inj Volume : 5.000 µl
    Acq. Method     : E:\DATA\WL\WL-18-86\WL-18-86HIJ-RAC 2017-12-19 17-16-59\ODH-90-10-1.0ML-ALL
                      -254NM-30MIN.M
    Last changed    : 12/19/2017 5:33:14 PM by SYSTEM
                      (modified after loading)
    Analysis Method : E:\DATA\WL\WL-18-86\WL-18-86HIJ-RAC 2017-12-19 17-16-59\ODH-90-10-1.0ML-ALL
                      -254NM-30MIN.M (Sequence Method)
    Last changed    : 5/2/2018 8:37:57 PM by SYSTEM
                      (modified after loading)
    Additional Info : Peak(s) manually integrated
```

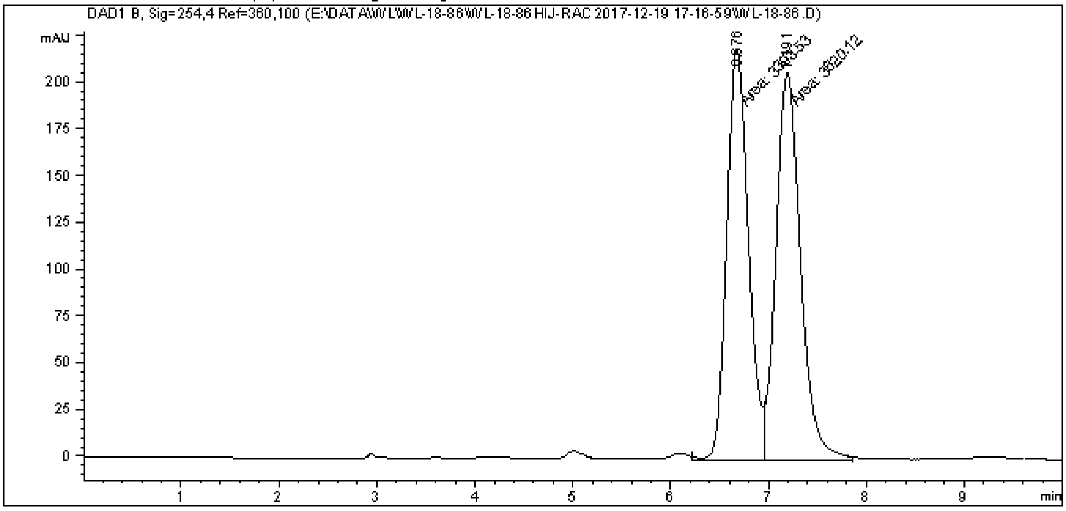


```
    ==================================================================
                         Area Percent Report
    ==================================================================

    Sorted By             :      Signal
    Multiplier            :      1.0000
    Dilution              :      1.0000
    Do not use Multiplier & Dilution Factor with ISTDs


    Signal 1: DAD1 B, Sig=254,4 Ref=360,100

    Peak RetTime Type  Width     Area      Height     Area
      #   [min]        [min]   [mAU*s]     [mAU]        %
    ----|-------|----|-------|----------|----------|--------|
       1   6.676 MF    0.2584 3393.52856  218.83951  48.3846
       2   7.191 FM    0.2914 3620.12476  207.07100  51.6154

    Totals :                  7013.65332  425.91051

    ==================================================================
```


Supplementary Figure 142. HPLC spectrum of (*rac*)-**3n**

Data File E:\DATA\WL\WL-18-86\WL-18-86FH-OPT 2017-12-29 16-19-14\WL-18-861.D
Sample Name: WL-18-86H-OPT

===

```
Acq. Operator   : SYSTEM                        Seq. Line :    2
Acq. Instrument : 1260                           Location :   94
Injection Date  : 12/29/2017 4:37:00 PM               Inj :    1
                                               Inj Volume : 5.000 µl
Acq. Method     : E:\DATA\WL\WL-18-86\WL-18-86FH-OPT 2017-12-29 16-19-14\ODH-90-10-1.0ML-ALL-
                  254NM-30MIN.M
Last changed    : 12/29/2017 4:19:26 PM by SYSTEM
Analysis Method : E:\DATA\WL\WL-18-86\WL-18-86FH-OPT 2017-12-29 16-19-14\ODH-90-10-1.0ML-ALL-
                  254NM-30MIN.M (Sequence Method)
Last changed    : 5/2/2018 8:35:45 PM by SYSTEM
                  (modified after loading)
Additional Info : Peak(s) manually integrated
```

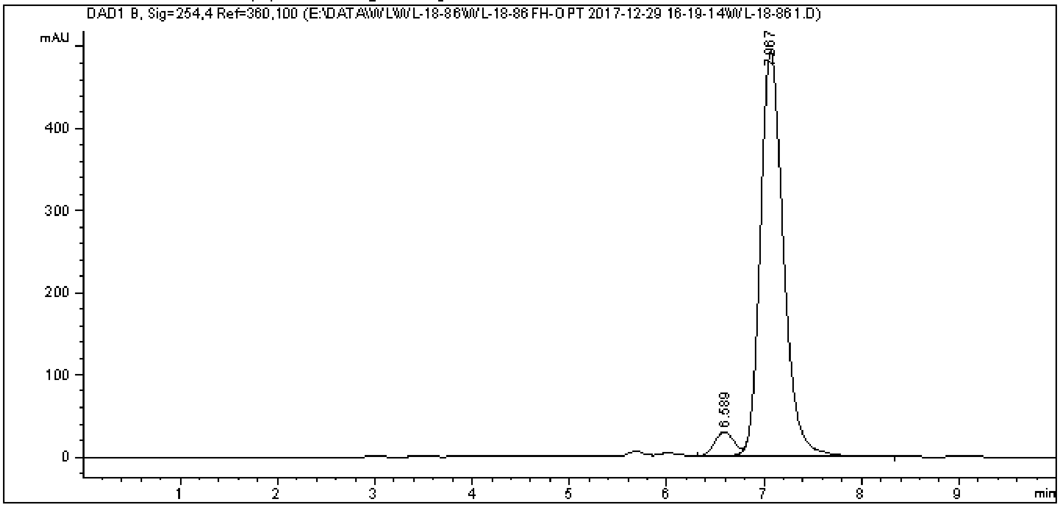

===
Area Percent Report
===

```
Sorted By          :      Signal
Multiplier         :      1.0000
Dilution           :      1.0000
Do not use Multiplier & Dilution Factor with ISTDs
```

Signal 1: DAD1 B, Sig=254,4 Ref=360,100

Peak #	RetTime [min]	Type	Width [min]	Area [mAU*s]	Height [mAU]	Area %
1	6.589	BV E	0.2090	395.03668	29.35155	4.6280
2	7.067	VB R	0.2531	8140.69873	494.31427	95.3720

Totals : 8535.73541 523.66582

===
*** End of Report ***

1260 5/2/2018 8:36:11 PM SYSTEM Page 1 of 1

Supplementary Figure 143. HPLC spectrum of (*S*)-**3n**

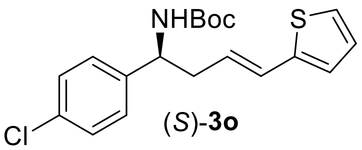

(*S*)-**3o**

```
Data File E:\DATA\WL\WL-18-86\WL-18-86HIJ-RAC 2017-12-19 17-16-59\WL-18-861.D
Sample Name: WL-18-86I-RAC

    =====================================================================
    Acq. Operator   : SYSTEM                        Seq. Line :   2
    Acq. Instrument : 1260                           Location :   32
    Injection Date  : 12/19/2017 5:34:54 PM               Inj :   1
                                                   Inj Volume : 5.000 µl
    Acq. Method     : E:\DATA\WL\WL-18-86\WL-18-86HIJ-RAC 2017-12-19 17-16-59\ODH-90-10-1.0ML-ALL
                      -254NM-30MIN.M
    Last changed    : 12/19/2017 5:33:14 PM by SYSTEM
    Analysis Method : E:\DATA\WL\WL-18-86\WL-18-86HIJ-RAC 2017-12-19 17-16-59\ODH-90-10-1.0ML-ALL
                      -254NM-30MIN.M (Sequence Method)
    Last changed    : 5/2/2018 8:38:17 PM by SYSTEM
                      (modified after loading)
    Additional Info : Peak(s) manually integrated
```

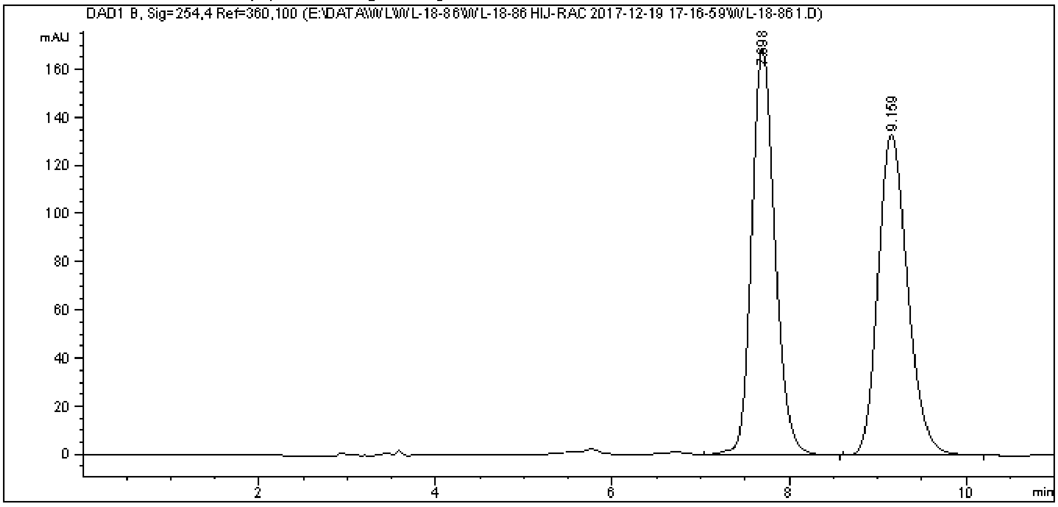


```
    =====================================================================
                             Area Percent Report
    =====================================================================

    Sorted By             :      Signal
    Multiplier            :      1.0000
    Dilution              :      1.0000
    Do not use Multiplier & Dilution Factor with ISTDs


    Signal 1: DAD1 B, Sig=254,4 Ref=360,100

    Peak RetTime Type  Width     Area      Height     Area
      #   [min]        [min]   [mAU*s]     [mAU]        %
    ----|-------|----|-------|----------|----------|--------|
       1   7.698 BB    0.2837 3092.12280  168.05588  50.0169
       2   9.159 BB    0.3572 3090.02808  132.90730  49.9831

    Totals :                  6182.15088  300.96318


    =====================================================================
                             *** End of Report ***

1260 5/2/2018 8:38:20 PM SYSTEM                                          Page 1 of 1
```

Supplementary Figure 144. HPLC spectrum of (*rac*)-**3o**

Data File E:\DATA\WL\WL-18-86\WL-18-86IJ-OPT 2018-01-03 00-44-31\WL-18-86.D
Sample Name: WL-18-86I-OPT

===

Acq. Operator : SYSTEM Seq. Line : 1
Acq. Instrument : 1260 Location : 11
Injection Date : 1/3/2018 12:45:52 AM Inj : 1
Inj Volume : 5.000 µl
Acq. Method : E:\DATA\WL\WL-18-86\WL-18-86IJ-OPT 2018-01-03 00-44-31\ODH-90-10-1.0ML-ALL-254NM-30MIN.M
Last changed : 1/3/2018 12:59:55 AM by SYSTEM
(modified after loading)
Analysis Method : E:\DATA\WL\WL-18-86\WL-18-86IJ-OPT 2018-01-03 00-44-31\ODH-90-10-1.0ML-ALL-254NM-30MIN.M (Sequence Method)
Last changed : 5/2/2018 8:40:37 PM by SYSTEM
(modified after loading)
Additional Info : Peak(s) manually integrated

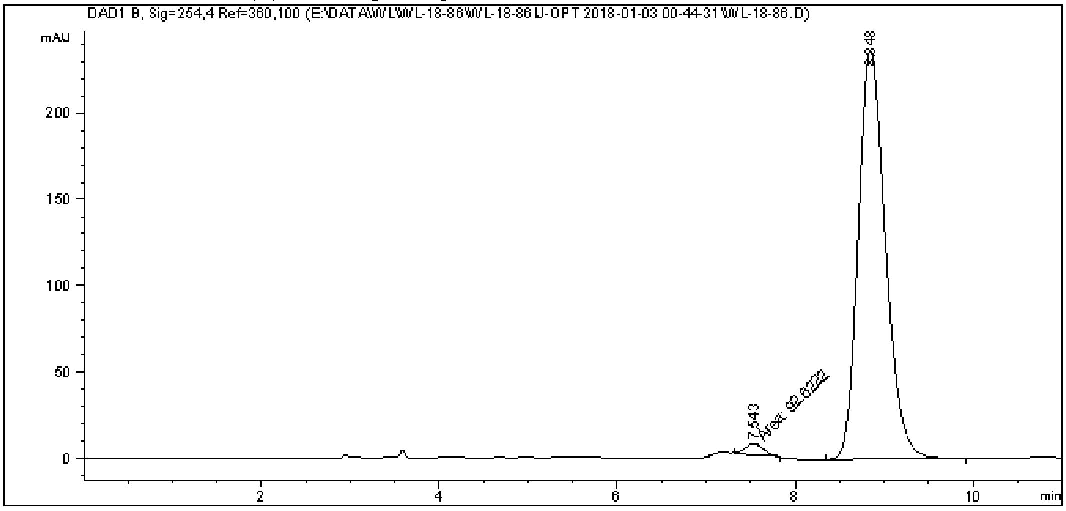

===

Area Percent Report

===

Sorted By : Signal
Multiplier : 1.0000
Dilution : 1.0000
Do not use Multiplier & Dilution Factor with ISTDs

Signal 1: DAD1 B, Sig=254,4 Ref=360,100

Peak #	RetTime [min]	Type	Width [min]	Area [mAU*s]	Height [mAU]	Area %
1	7.543	MM	0.2432	92.62218	6.34824	1.8041
2	8.848	BB	0.3275	5041.41699	236.64812	98.1959

Totals : 5134.03917 242.99636

===

1260 5/2/2018 8:40:40 PM SYSTEM Page 1 of 2

Supplementary Figure 145. HPLC spectrum of (*S*)-**3o**

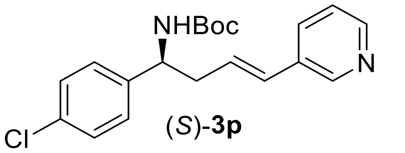

(*S*)-**3p**

Data File E:\DATA\ZQ\ZQ-10-47\ZQ-10-47 2018-01-09 16-03-15\WL-18-88M-RAC2.D
Sample Name: ZQ-10-54-3-P

```
=====================================================================
Acq. Operator   : SYSTEM                        Seq. Line :    3
Acq. Instrument : 1260                           Location :   74
Injection Date  : 1/9/2018 5:07:31 PM                 Inj :    1
                                               Inj Volume : 5.000 µl
Acq. Method     : E:\DATA\ZQ\ZQ-10-47\ZQ-10-47 2018-01-09 16-03-15\OD-90-10-254NM-30MIN.M
Last changed    : 1/9/2018 4:03:15 PM by SYSTEM
Analysis Method : E:\DATA\ZQ\ZQ-10-47\ZQ-10-47 2018-01-09 16-03-15\OD-90-10-254NM-30MIN.M (
                  Sequence Method)
Last changed    : 4/11/2018 2:40:15 PM by SYSTEM
                  (modified after loading)
Additional Info : Peak(s) manually integrated
```

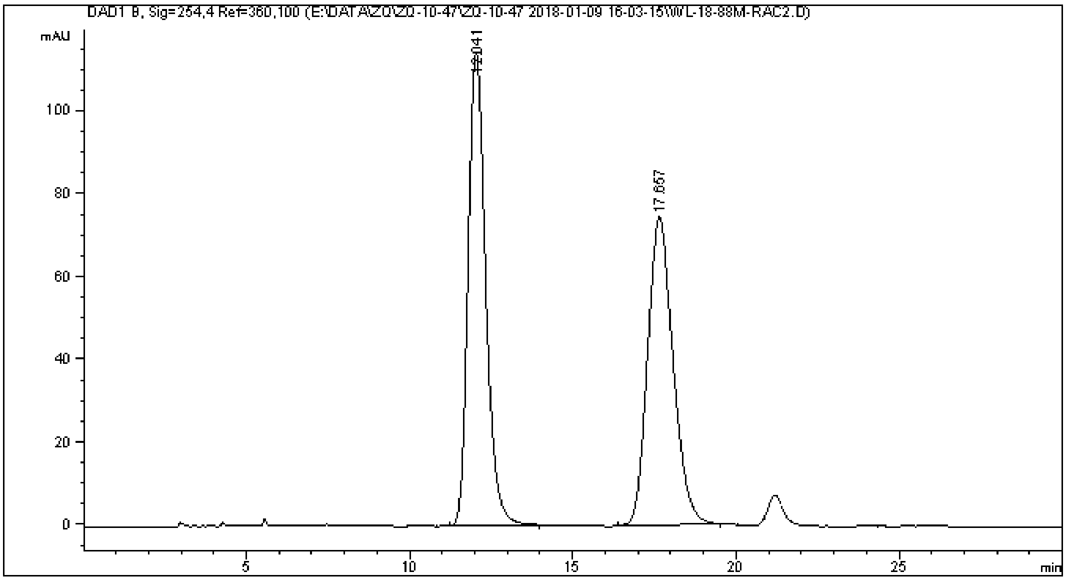


```
=====================================================================
                         Area Percent Report
=====================================================================

Sorted By             :      Signal
Multiplier            :      1.0000
Dilution              :      1.0000
Do not use Multiplier & Dilution Factor with ISTDs


Signal 1: DAD1 B, Sig=254,4 Ref=360,100

Peak RetTime Type  Width     Area      Height     Area
  #   [min]        [min]   [mAU*s]     [mAU]        %
----|-------|----|-------|----------|----------|--------|
   1  12.041 BB    0.5685 4273.55859  114.22523  51.5100
   2  17.657 BB    0.7504 4022.99683   74.45358  48.4900

Totals :                  8296.55542  188.67881
```


Supplementary Figure 146. HPLC spectrum of (*rac*)-**3p**

Data File E:\DATA\ZQ\ZQ-10-47\ZQ-10-47 2018-01-09 18-53-07\WL-18-88M-RAC1.D
Sample Name: ZQ-10-54-3-P

===

Acq. Operator : SYSTEM Seq. Line : 2
Acq. Instrument : 1260 Location : 79
Injection Date : 1/9/2018 7:16:09 PM Inj : 1
Inj Volume : 5.000 µl
Acq. Method : E:\DATA\ZQ\ZQ-10-47\ZQ-10-47 2018-01-09 18-53-07\OD-90-10-254NM-20MIN.M
Last changed : 1/9/2018 7:18:11 PM by SYSTEM
(modified after loading)
Analysis Method : E:\DATA\ZQ\ZQ-10-47\ZQ-10-47 2018-01-09 18-53-07\OD-90-10-254NM-20MIN.M (
Sequence Method)
Last changed : 4/11/2018 2:43:22 PM by SYSTEM
(modified after loading)
Additional Info : Peak(s) manually integrated

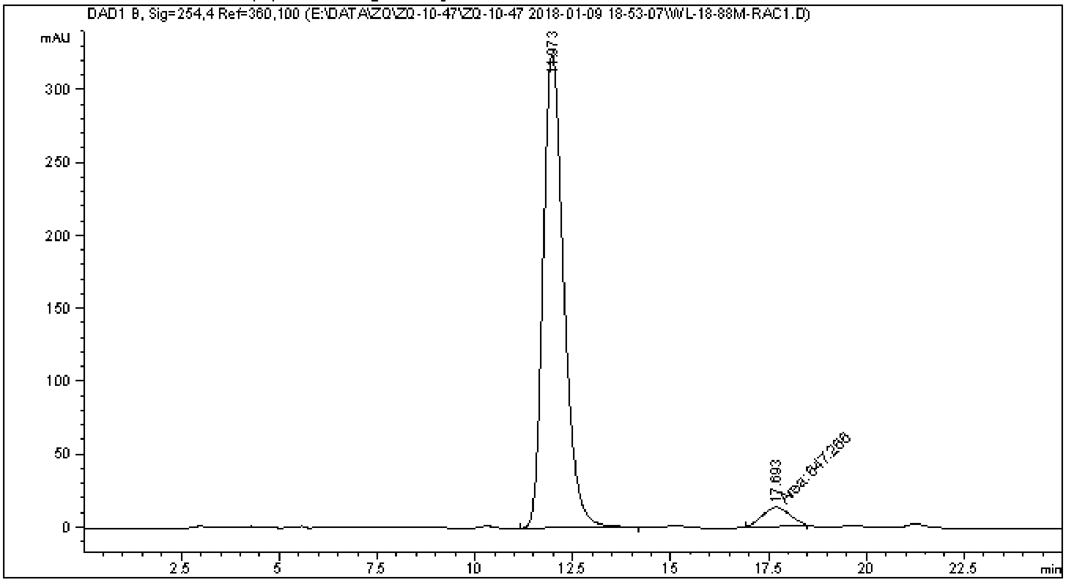

===

Area Percent Report

===

Sorted By : Signal
Multiplier : 1.0000
Dilution : 1.0000
Do not use Multiplier & Dilution Factor with ISTDs

Signal 1: DAD1 B, Sig=254,4 Ref=360,100

Peak #	RetTime [min]	Type	Width [min]	Area [mAU*s]	Height [mAU]	Area %
1	11.973	BB	0.5676	1.18973e4	324.61740	94.8403
2	17.693	MM	0.8249	647.26581	13.07803	5.1597

Totals : 1.25446e4 337.69543

Supplementary Figure 147. HPLC spectrum of (*S*)-**3p**

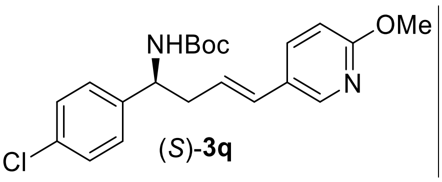

(*S*)-**3q**

Data File E:\DATA\WL\WL-18-86\WL-18-86HIJ-RAC 2017-12-19 17-16-59\WL-18-862.D
Sample Name: WL-18-86J-RAC

===
Acq. Operator : SYSTEM Seq. Line : 3
Acq. Instrument : 1260 Location : 33
Injection Date : 12/19/2017 5:51:21 PM Inj : 1
Inj Volume : 5.000 µl
Acq. Method : E:\DATA\WL\WL-18-86\WL-18-86HIJ-RAC 2017-12-19 17-16-59\ODH-90-10-1.0ML-ALL-254NM-30MIN.M
Last changed : 12/19/2017 5:33:14 PM by SYSTEM
Analysis Method : E:\DATA\WL\WL-18-86\WL-18-86HIJ-RAC 2017-12-19 17-16-59\ODH-90-10-1.0ML-ALL-254NM-30MIN.M (Sequence Method)
Last changed : 5/2/2018 8:38:17 PM by SYSTEM
(modified after loading)
Additional Info : Peak(s) manually integrated

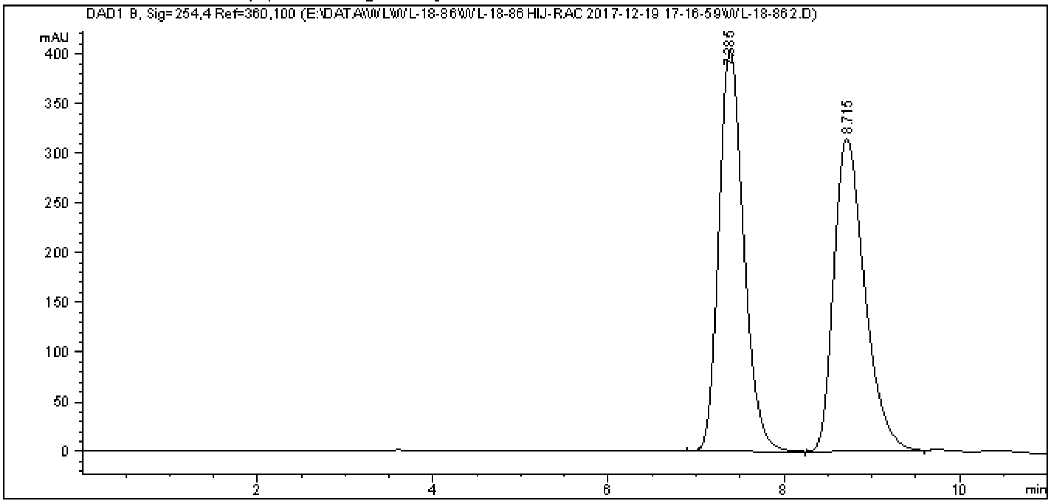

===
Area Percent Report
===

Sorted By : Signal
Multiplier : 1.0000
Dilution : 1.0000
Do not use Multiplier & Dilution Factor with ISTDs

Signal 1: DAD1 B, Sig=254,4 Ref=360,100

Peak #	RetTime [min]	Type	Width [min]	Area [mAU*s]	Height [mAU]	Area %
1	7.385	BB	0.3026	7966.70313	403.12137	50.9309
2	8.715	BB	0.3770	7675.47900	314.23761	49.0691
Totals :				1.56422e4	717.35898	

===
*** End of Report ***

1260 5/2/2018 8:38:27 PM SYSTEM Page 1 of 1

Supplementary Figure 148. HPLC spectrum of (*rac*)-**3q**

Data File E:\DATA\WL\WL-18-86\WL-18-86IJ-OPT 2018-01-03 00-44-31\WL-18-861.D
Sample Name: WL-18-86J-OPT

===

Acq. Operator : SYSTEM Seq. Line : 2
Acq. Instrument : 1260 Location : 12
Injection Date : 1/3/2018 1:02:15 AM Inj : 1
Inj Volume : 5.000 µl
Acq. Method : E:\DATA\WL\WL-18-86\WL-18-86IJ-OPT 2018-01-03 00-44-31\ODH-90-10-1.0ML-ALL-254NM-30MIN.M
Last changed : 1/3/2018 12:59:55 AM by SYSTEM
Analysis Method : E:\DATA\WL\WL-18-86\WL-18-86IJ-OPT 2018-01-03 00-44-31\ODH-90-10-1.0ML-ALL-254NM-30MIN.M (Sequence Method)
Last changed : 5/2/2018 8:40:37 PM by SYSTEM
(modified after loading)
Additional Info : Peak(s) manually integrated

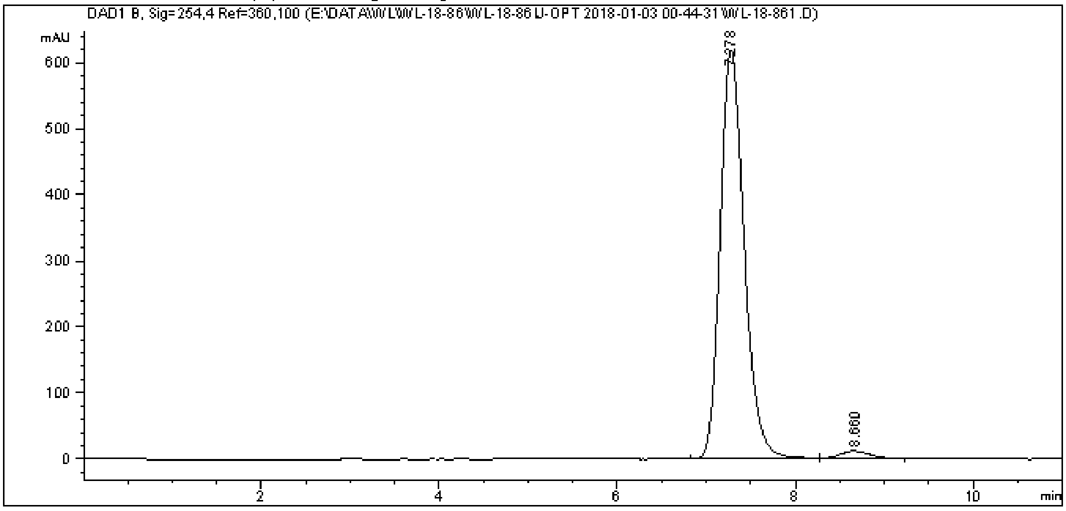

===

Area Percent Report

===

Sorted By : Signal
Multiplier : 1.0000
Dilution : 1.0000
Do not use Multiplier & Dilution Factor with ISTDs

Signal 1: DAD1 B, Sig=254,4 Ref=360,100

Peak #	RetTime [min]	Type	Width [min]	Area [mAU*s]	Height [mAU]	Area %
1	7.278	BB	0.2909	1.16005e4	618.15558	97.9041
2	8.660	BB	0.3040	248.34328	10.93409	2.0959

Totals : 1.18488e4 629.08967

===

*** End of Report ***

1260 5/2/2018 8:40:54 PM SYSTEM Page 1 of 1

Supplementary Figure 149. HPLC spectrum of (*S*)-**3q**

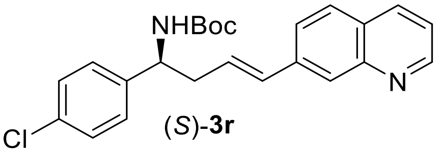

(*S*)-**3r**

Data File E:\DATA\ZQ\ZQ-10-59\ZQ-10-59 2018-01-18 14-58-07\SLM-8-37.D
Sample Name: ZQ-10-53-6Q-rac

===

Acq. Operator : SYSTEM Seq. Line : 1
Acq. Instrument : 1260 Location : 73
Injection Date : 1/18/2018 2:59:33 PM Inj : 1
Inj Volume : 5.000 µl
Acq. Method : E:\DATA\ZQ\ZQ-10-59\ZQ-10-59 2018-01-18 14-58-07\OD-90-10-254NM-30MIN.M
Last changed : 1/18/2018 2:58:07 PM by SYSTEM
Analysis Method : E:\DATA\ZQ\ZQ-10-59\ZQ-10-59 2018-01-18 14-58-07\OD-90-10-254NM-30MIN.M (Sequence Method)
Last changed : 4/11/2018 2:49:28 PM by SYSTEM
(modified after loading)
Additional Info : Peak(s) manually integrated

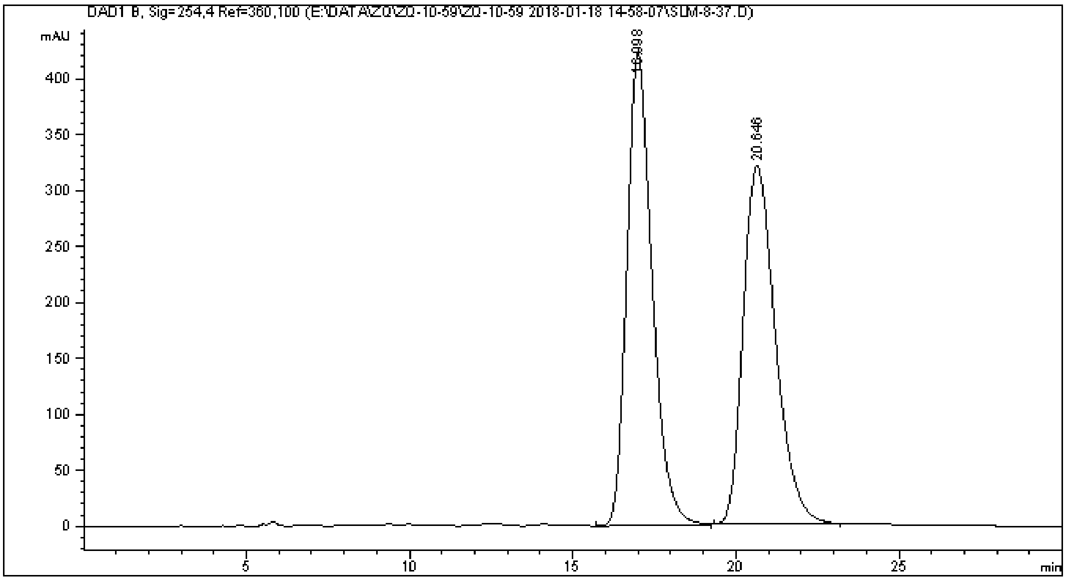

===

Area Percent Report

===

Sorted By : Signal
Multiplier : 1.0000
Dilution : 1.0000
Do not use Multiplier & Dilution Factor with ISTDs

Signal 1: DAD1 B, Sig=254,4 Ref=360,100

Peak #	RetTime [min]	Type	Width [min]	Area [mAU*s]	Height [mAU]	Area %
1	16.998	BB	0.7744	2.27096e4	422.27744	50.8277
2	20.646	BB	1.0224	2.19699e4	320.24136	49.1723

Totals : 4.46795e4 742.51880

1260 4/11/2018 2:49:31 PM SYSTEM Page 1 of 2

Supplementary Figure 150. HPLC spectrum of (*rac*)-**3r**

Data File E:\DATA\ZQ\ZQ-10-59\ZQ-10-59 2018-01-18 13-09-29\SLM-8-37.D
Sample Name: ZQ-10-59-6Q-opt

===

Acq. Operator : SYSTEM Seq. Line : 1
Acq. Instrument : 1260 Location : 71
Injection Date : 1/18/2018 1:10:53 PM Inj : 1
Inj Volume : 5.000 µl
Acq. Method : E:\DATA\ZQ\ZQ-10-59\ZQ-10-59 2018-01-18 13-09-29\OD-90-10-254NM-30MIN.M
Last changed : 1/18/2018 1:11:59 PM by SYSTEM
(modified after loading)
Analysis Method : E:\DATA\ZQ\ZQ-10-59\ZQ-10-59 2018-01-18 13-09-29\OD-90-10-254NM-30MIN.M (
Sequence Method)
Last changed : 4/11/2018 2:51:28 PM by SYSTEM
(modified after loading)
Additional Info : Peak(s) manually integrated

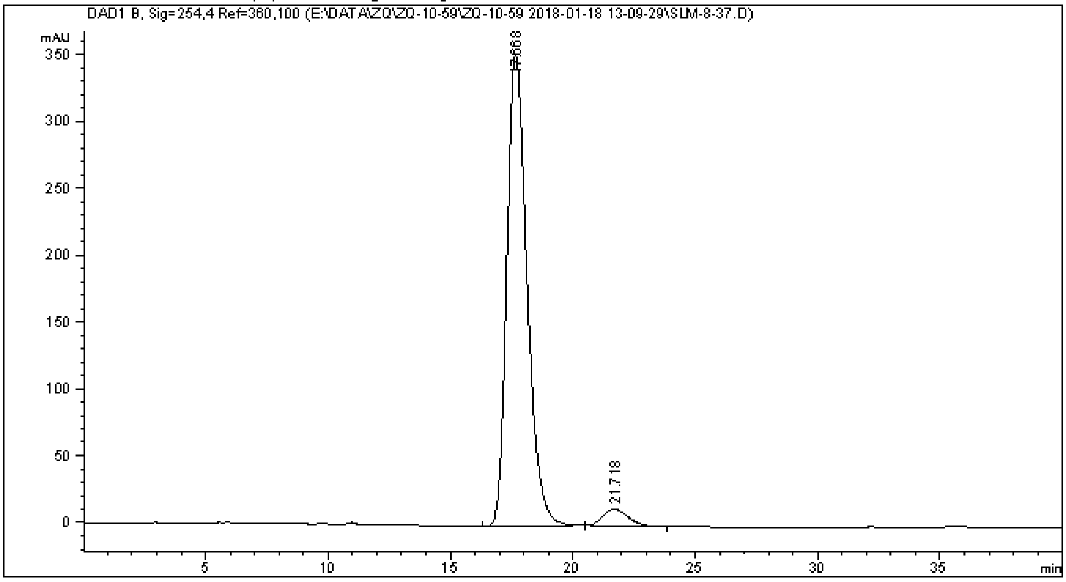

===

Area Percent Report

===

Sorted By : Signal
Multiplier : 1.0000
Dilution : 1.0000
Do not use Multiplier & Dilution Factor with ISTDs

Signal 1: DAD1 B, Sig=254,4 Ref=360,100

Peak #	RetTime [min]	Type	Width [min]	Area [mAU*s]	Height [mAU]	Area %
1	17.668	BB	0.8773	2.01842e4	352.91364	95.7065
2	21.718	BB	0.8388	905.48761	12.80782	4.2935

Totals : 2.10897e4 365.72145

Supplementary Figure 151. HPLC spectrum of (*S*)-**3r**

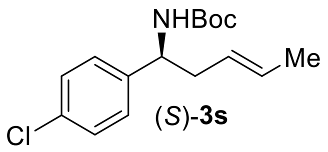

(*S*)-**3s**

Data File E:\DATA\WL\WL-18-86\WL-18-86M-RAC-AD 2018-04-16 20-31-26\WL-18-86.D
Sample Name: WL-18-86M-RAC

===

```
Acq. Operator   : SYSTEM                          Seq. Line :   1
Acq. Instrument : 1260                             Location :   1
Injection Date  : 4/16/2018 8:32:56 PM                  Inj :   1
                                                 Inj Volume : 5.000 µl
Acq. Method     : E:\DATA\WL\WL-18-86\WL-18-86M-RAC-AD 2018-04-16 20-31-26\ADH-97-3-1.0ML-ALL
                  -254NM-30MIN1.M
Last changed    : 4/16/2018 8:31:27 PM by SYSTEM
Analysis Method : E:\DATA\WL\WL-18-86\WL-18-86M-RAC-AD 2018-04-16 20-31-26\ADH-97-3-1.0ML-ALL
                  -254NM-30MIN1.M (Sequence Method)
Last changed    : 5/2/2018 8:54:51 PM by SYSTEM
                  (modified after loading)
Additional Info : Peak(s) manually integrated
```

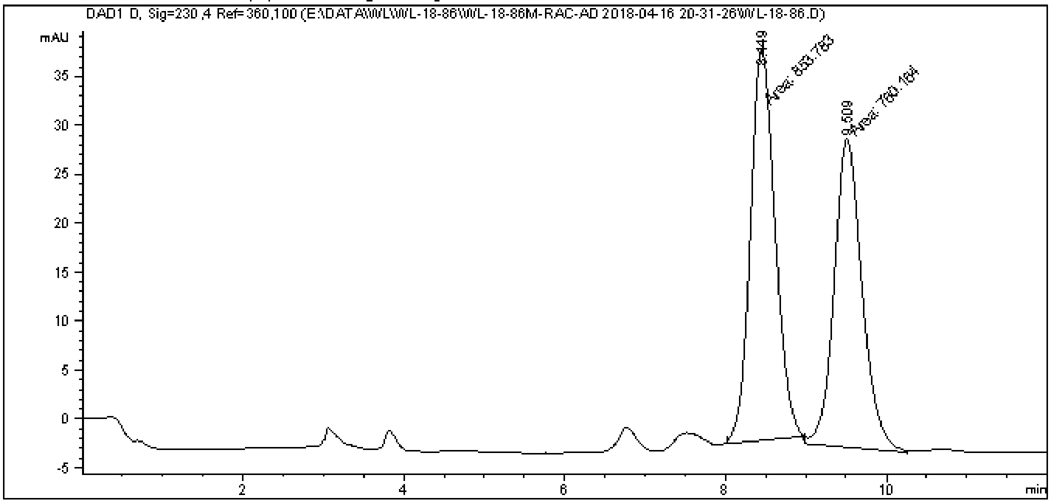

===

Area Percent Report

===

```
Sorted By             :      Signal
Multiplier            :      1.0000
Dilution              :      1.0000
Do not use Multiplier & Dilution Factor with ISTDs
```

Signal 1: DAD1 D, Sig=230,4 Ref=360,100

Peak #	RetTime [min]	Type	Width [min]	Area [mAU*s]	Height [mAU]	Area %
1	8.449	MM	0.3574	853.78326	39.81672	52.9003
2	9.509	MM	0.4029	760.16449	31.44429	47.0997

Totals : 1613.94775 71.26101

===

*** End of Report ***

1260 5/2/2018 8:54:53 PM SYSTEM Page 1 of 1

Supplementary Figure 152. HPLC spectrum of (*rac*)-**3s**

Data File E:\DATA\WL\WL-18-86\WL-18-86M-OPT-AD 2018-04-16 21-59-06\WL-18-86.D
Sample Name: WL-18-86M-OPT

===

Acq. Operator : SYSTEM Seq. Line : 1
Acq. Instrument : 1260 Location : 2
Injection Date : 4/16/2018 10:00:28 PM Inj : 1
Inj Volume : 5.000 µl
Acq. Method : E:\DATA\WL\WL-18-86\WL-18-86M-OPT-AD 2018-04-16 21-59-06\ADH-97-3-1.0ML-ALL-254NM-30MIN1.M
Last changed : 4/16/2018 9:59:06 PM by SYSTEM
Analysis Method : E:\DATA\WL\WL-18-86\WL-18-86M-OPT-AD 2018-04-16 21-59-06\ADH-97-3-1.0ML-ALL-254NM-30MIN1.M (Sequence Method)
Last changed : 5/2/2018 8:54:00 PM by SYSTEM
(modified after loading)
Additional Info : Peak(s) manually integrated

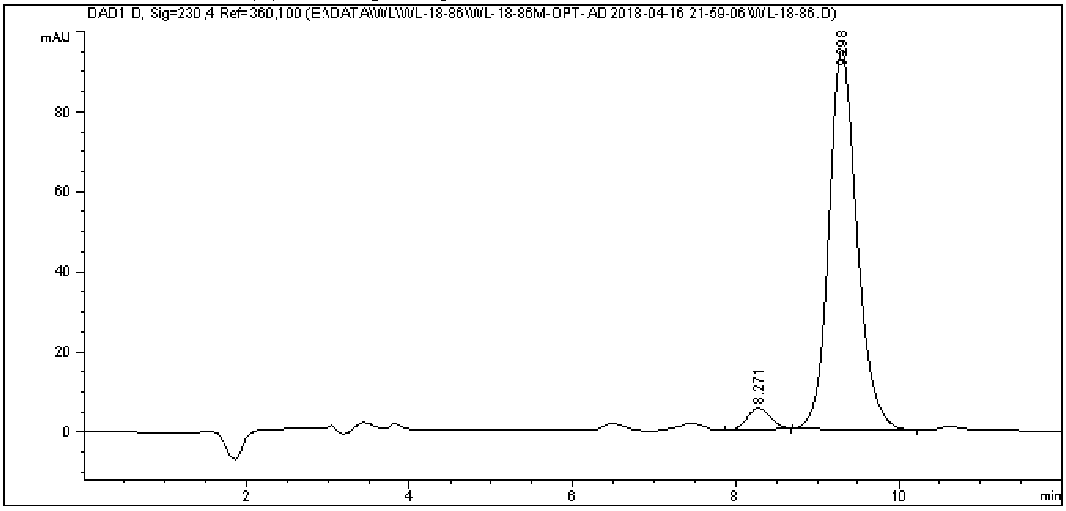

===

Area Percent Report

===

Sorted By : Signal
Multiplier : 1.0000
Dilution : 1.0000
Do not use Multiplier & Dilution Factor with ISTDs

Signal 1: DAD1 D, Sig=230,4 Ref=360,100

Peak #	RetTime [min]	Type	Width [min]	Area [mAU*s]	Height [mAU]	Area %
1	8.271	BB	0.2883	105.30482	5.33407	4.4239
2	9.298	BB	0.3675	2275.03735	94.62646	95.5761
Totals :				2380.34217	99.96053	

===

*** End of Report ***

1260 5/2/2018 8:54:02 PM SYSTEM Page 1 of 1

Supplementary Figure 153. HPLC spectrum of (*S*)-**3s**

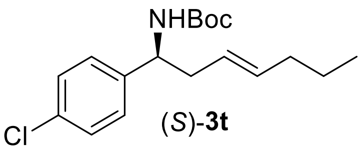

(*S*)-**3t**

```
Data File E:\DATA\WL\WL-18-86\WL-18-86-NH2-RAC 2018-02-01 16-21-44\WL-18-862.D
Sample Name: WL-18-86L-NH2-RAC

    =====================================================================
    Acq. Operator   : SYSTEM                          Seq. Line :   3
    Acq. Instrument : 1260                             Location :   82
    Injection Date  : 2/1/2018 4:42:30 PM                   Inj :   1
                                                     Inj Volume : 5.000 µl
    Acq. Method     : E:\DATA\WL\WL-18-86\WL-18-86-NH2-rac 2018-02-01 16-21-44\ODH-90-10-1.0ML-
                      ALL-254NM-30MIN.M
    Last changed    : 2/1/2018 5:10:24 PM by SYSTEM
                      (modified after loading)
    Analysis Method : E:\DATA\WL\WL-18-86\WL-18-86-NH2-rac 2018-02-01 16-21-44\ODH-90-10-1.0ML-
                      ALL-254NM-30MIN.M (Sequence Method)
    Last changed    : 5/2/2018 8:44:42 PM by SYSTEM
                      (modified after loading)
    Additional Info : Peak(s) manually integrated
```

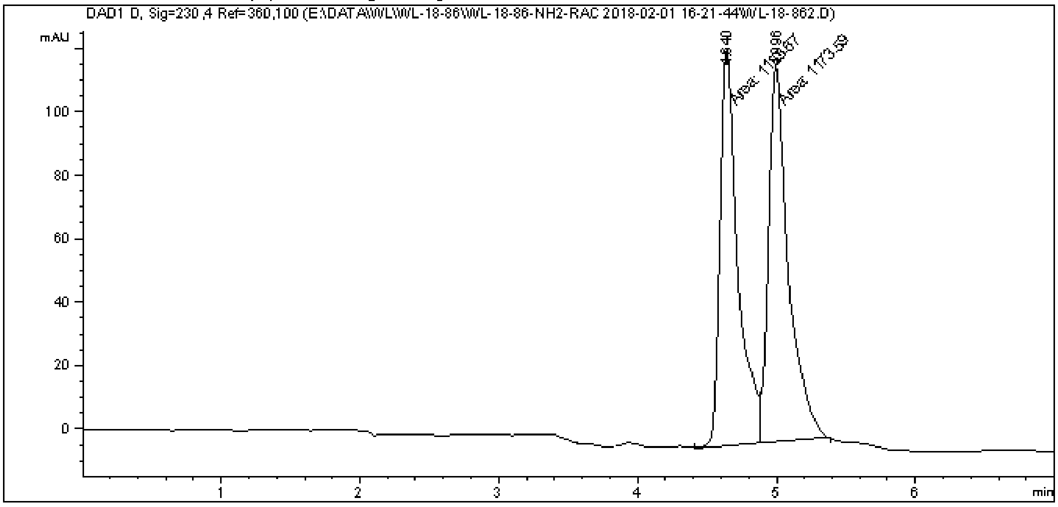


```
    =====================================================================
                             Area Percent Report
    =====================================================================

    Sorted By             :      Signal
    Multiplier            :      1.0000
    Dilution              :      1.0000
    Do not use Multiplier & Dilution Factor with ISTDs


    Signal 1: DAD1 D, Sig=230,4 Ref=360,100
```

Peak #	RetTime [min]	Type	Width [min]	Area [mAU*s]	Height [mAU]	Area %
1	4.640	MF	0.1505	1125.66711	124.66203	48.9580
2	4.996	FM	0.1660	1173.58508	117.86191	51.0420

Totals : 2299.25220 242.52394

===

1260 5/2/2018 8:44:45 PM SYSTEM Page 1 of 2

Supplementary Figure 154. HPLC spectrum of (*rac*)-**3t**

Data File E:\DATA\WL\WL-18-86\ALKYL CARBONATES 2018-04-12 21-51-28\WL-18-861.D
Sample Name: wl-18-86L-S+L

===

Acq. Operator : SYSTEM   Seq. Line : 2
Acq. Instrument : 1260   Location : 4
Injection Date : 4/12/2018 10:09:32 PM   Inj : 1
                                          Inj Volume : 5.000 µl
Acq. Method : E:\DATA\WL\WL-18-86\Alkyl Carbonates 2018-04-12 21-51-28\ODH-90-10-1.0ML-ALL-254NM-30MIN.M
Last changed : 4/12/2018 10:25:41 PM by SYSTEM
(modified after loading)
Analysis Method : E:\DATA\WL\WL-18-86\Alkyl Carbonates 2018-04-12 21-51-28\ODH-90-10-1.0ML-ALL-254NM-30MIN.M (Sequence Method)
Last changed : 5/2/2018 8:48:02 PM by SYSTEM
(modified after loading)
Additional Info : Peak(s) manually integrated

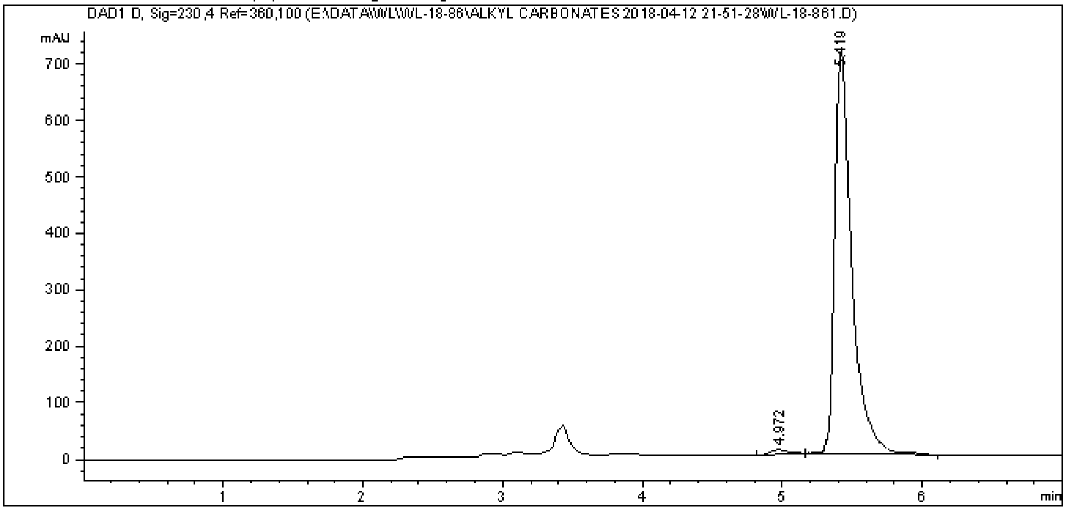

===

Area Percent Report

===

Sorted By : Signal
Multiplier : 1.0000
Dilution : 1.0000
Do not use Multiplier & Dilution Factor with ISTDs

Signal 1: DAD1 D, Sig=230,4 Ref=360,100

Peak #	RetTime [min]	Type	Width [min]	Area [mAU*s]	Height [mAU]	Area %
1	4.972	BV R	0.1345	74.89253	8.77698	1.1924
2	5.419	VV R	0.1271	6205.86035	713.16821	98.8076

Totals : 6280.75288 721.94520

===

1260 5/2/2018 8:49:07 PM SYSTEM   Page 1 of 2

Supplementary Figure 155. HPLC spectrum of (*S*)-**3t**

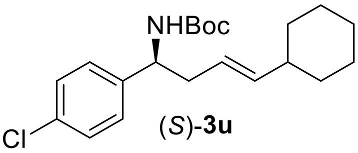

Data File E:\DATA\ZQ\ZQ-10-68\ZQ-10-68 2018-01-26 22-03-29\CC-4-17B-OPT.D
Sample Name: ZQ-10-68-rac

```
=====================================================================
Acq. Operator   : SYSTEM                        Seq. Line :   1
Acq. Instrument : 1260                           Location :   64
Injection Date  : 1/26/2018 10:05:02 PM               Inj :   1
                                               Inj Volume : 5.000 µl
Acq. Method     : E:\DATA\ZQ\ZQ-10-68\ZQ-10-68 2018-01-26 22-03-29\OD-97-3-210NM-20MIN-5uL.M
Last changed    : 1/26/2018 10:03:29 PM by SYSTEM
Analysis Method : E:\DATA\ZQ\ZQ-10-68\ZQ-10-68 2018-01-26 22-03-29\OD-97-3-210NM-20MIN-5uL.M
                  (Sequence Method)
Last changed    : 5/5/2018 11:48:41 AM by SYSTEM
                  (modified after loading)
Additional Info : Peak(s) manually integrated
```

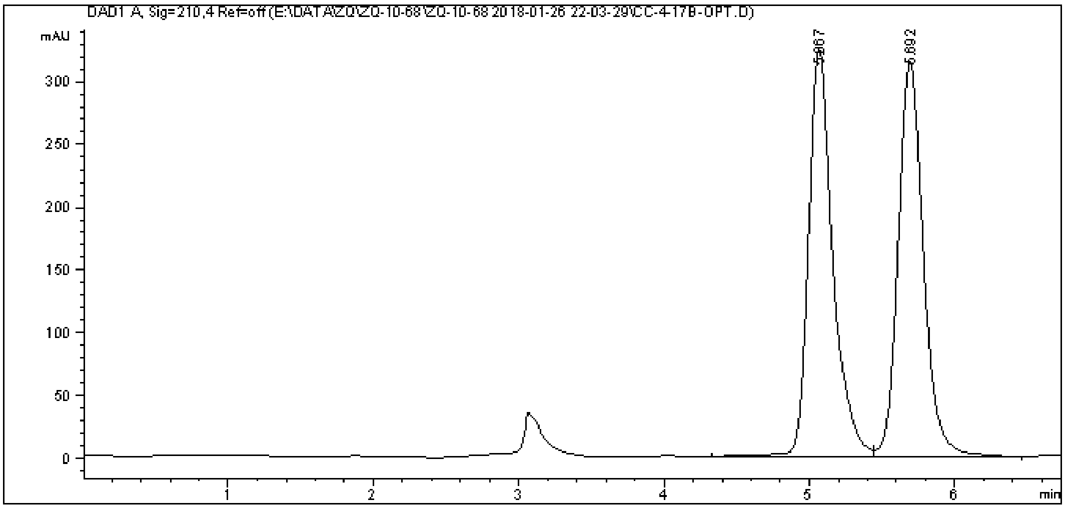


```
=====================================================================
                         Area Percent Report
=====================================================================

Sorted By             :      Signal
Multiplier            :      1.0000
Dilution              :      1.0000
Do not use Multiplier & Dilution Factor with ISTDs


Signal 1: DAD1 A, Sig=210,4 Ref=off

Peak RetTime Type  Width     Area      Height     Area
  #   [min]        [min]   [mAU*s]     [mAU]        %
----|-------|----|-------|----------|----------|--------|
   1   5.067 BV    0.1743 3785.05786  323.99088  49.9613
   2   5.692 VB    0.1848 3790.91846  314.11087  50.0387

Totals :                  7575.97632  638.10175


=====================================================================
                          *** End of Report ***
```


Supplementary Figure 156. HPLC spectrum of (*rac*)-**3u**

```
Data File E:\DATA\WL\WL-18-86\CROTYL-OPT-CRYSTALIZATION 2018-04-16 20-19-07\WL-18-86.D
Sample Name: crotyl-opt

    =====================================================================
    Acq. Operator   : SYSTEM                        Seq. Line :    1
    Acq. Instrument : 1260                           Location :    6
    Injection Date  : 4/16/2018 8:20:34 PM                Inj :    1
                                                    Inj Volume : 5.000 µl
    Acq. Method     : E:\DATA\WL\WL-18-86\crotyl-opt-crystalization 2018-04-16 20-19-07\ODH-97-3-
                      1.0ML-ALL-254NM-30MIN.M
    Last changed    : 4/16/2018 8:21:18 PM by SYSTEM
                      (modified after loading)
    Analysis Method : E:\DATA\WL\WL-18-86\crotyl-opt-crystalization 2018-04-16 20-19-07\ODH-97-3-
                      1.0ML-ALL-254NM-30MIN.M (Sequence Method)
    Last changed    : 5/2/2018 8:57:33 PM by SYSTEM
                      (modified after loading)
    Additional Info : Peak(s) manually integrated
```

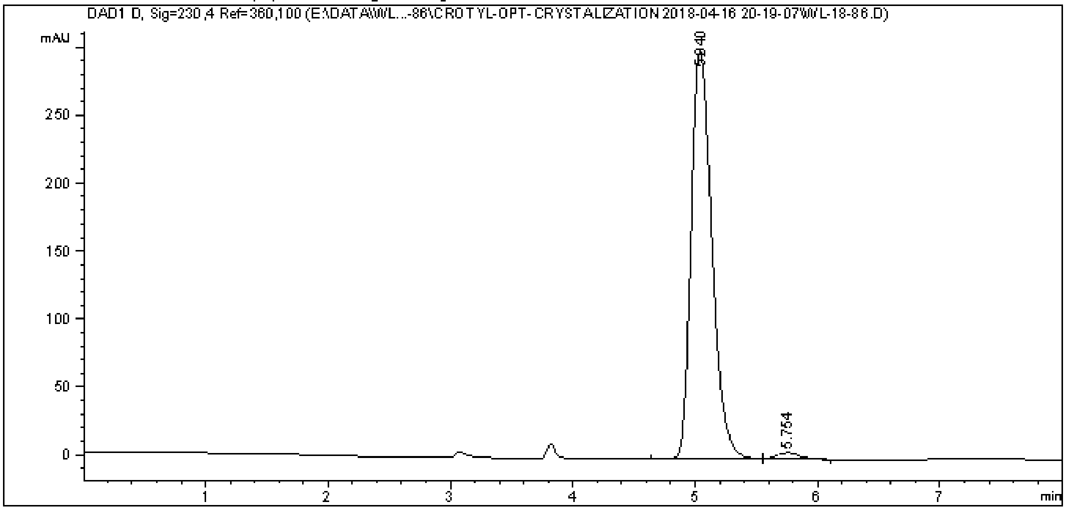


```
    =====================================================================
                             Area Percent Report
    =====================================================================

    Sorted By             :      Signal
    Multiplier            :      1.0000
    Dilution              :      1.0000
    Do not use Multiplier & Dilution Factor with ISTDs


    Signal 1: DAD1 D, Sig=230,4 Ref=360,100

    Peak RetTime Type  Width     Area      Height     Area
      #   [min]        [min]   [mAU*s]     [mAU]        %
    ----|-------|----|-------|----------|----------|--------|
       1   5.040 BB    0.1859 3544.73584  299.96707  98.5125
       2   5.754 BB    0.1880   53.52421    4.39614   1.4875

    Totals :                  3598.26005  304.36321


    =====================================================================
```


Supplementary Figure 157. HPLC spectrum of (*S*)-**3u**

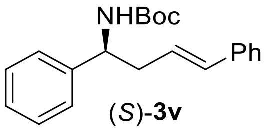

(*S*)-**3v**

Data File E:\DATA\WL\WL-18-88\WL-18-88EFGH-RAC 2017-12-26 15-23-42\WL-18-884.D
Sample Name: WL-18-88E

===

Acq. Operator : SYSTEM Seq. Line : 5
Acq. Instrument : 1260 Location : 4
Injection Date : 12/26/2017 5:30:51 PM Inj : 1
Inj Volume : 5.000 µl
Acq. Method : E:\DATA\WL\WL-18-88\WL-18-88EFGH-RAC 2017-12-26 15-23-42\AdH-90-10-254NM-25min.M
Last changed : 12/26/2017 3:23:42 PM by SYSTEM
Analysis Method : E:\DATA\WL\WL-18-88\WL-18-88EFGH-RAC 2017-12-26 15-23-42\AdH-90-10-254NM-25min.M (Sequence Method)
Last changed : 5/2/2018 9:10:55 PM by SYSTEM
(modified after loading)
Additional Info : Peak(s) manually integrated

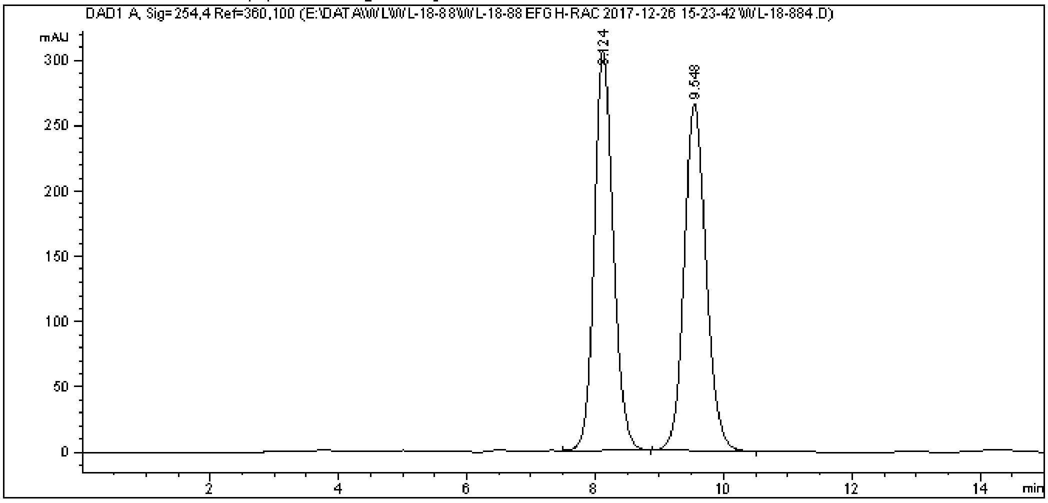

===

Area Percent Report

===

Sorted By : Signal
Multiplier : 1.0000
Dilution : 1.0000
Do not use Multiplier & Dilution Factor with ISTDs

Signal 1: DAD1 A, Sig=254,4 Ref=360,100

Peak #	RetTime [min]	Type	Width [min]	Area [mAU*s]	Height [mAU]	Area %
1	8.124	BB	0.3151	6308.34619	306.51801	50.0519
2	9.548	BB	0.3624	6295.25342	265.75354	49.9481

Totals : 1.26036e4 572.27155

===

*** End of Report ***

1260 5/2/2018 9:10:58 PM SYSTEM Page 1 of 1

Supplementary Figure 158. HPLC spectrum of (*rac*)-**3v**

Data File E:\DATA\WL\WL-18-88\WL-18-88DEFG-OPT 2018-01-03 18-53-47\WL-18-881.D
Sample Name: WL-18-88E-OPT

```
=====================================================================
Acq. Operator   : SYSTEM                        Seq. Line :   2
Acq. Instrument : 1260                           Location :   42
Injection Date  : 1/3/2018 7:11:42 PM                 Inj :   1
                                               Inj Volume : 5.000 µl
Acq. Method     : E:\DATA\WL\WL-18-88\WL-18-88DEFG-OPT 2018-01-03 18-53-47\AdH-90-10-254NM-
                  25min.M
Last changed    : 1/3/2018 6:54:05 PM by SYSTEM
Analysis Method : E:\DATA\WL\WL-18-88\WL-18-88DEFG-OPT 2018-01-03 18-53-47\AdH-90-10-254NM-
                  25min.M (Sequence Method)
Last changed    : 5/2/2018 9:17:13 PM by SYSTEM
                  (modified after loading)
Additional Info : Peak(s) manually integrated
```

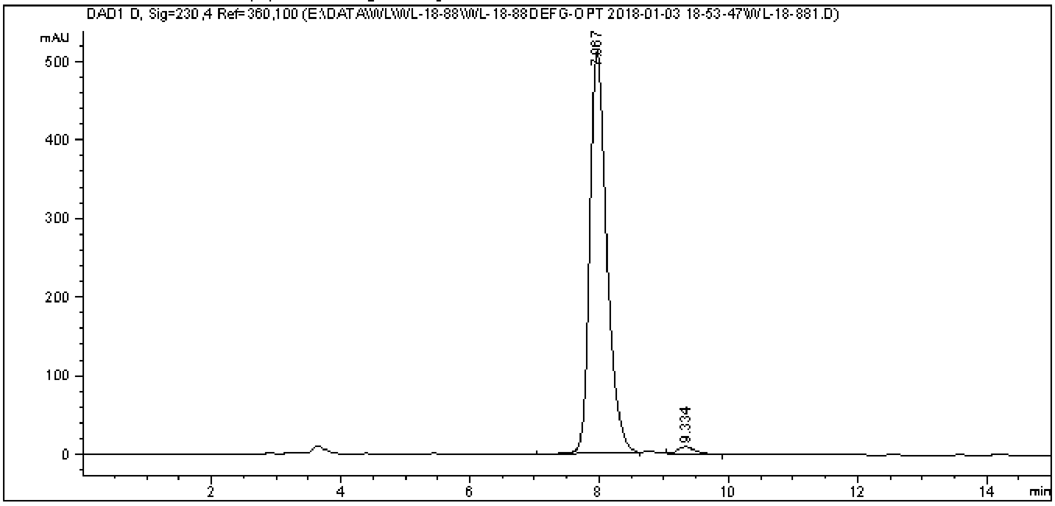


```
=====================================================================
                         Area Percent Report
=====================================================================

Sorted By             :      Signal
Multiplier            :      1.0000
Dilution              :      1.0000
Do not use Multiplier & Dilution Factor with ISTDs


Signal 1: DAD1 D, Sig=230,4 Ref=360,100

Peak RetTime Type  Width     Area      Height     Area
  #   [min]        [min]   [mAU*s]     [mAU]        %
----|-------|----|-------|----------|----------|--------|
   1   7.967 BB    0.2771 9347.10449  511.79199  98.3257
   2   9.334 BB    0.2775  159.16798    8.46116   1.6743

Totals :                  9506.27248  520.25315


=====================================================================
                          *** End of Report ***
```

1260 5/2/2018 9:17:16 PM SYSTEM Page 1 of 1

Supplementary Figure 159. HPLC spectrum of (*S*)-**3v**

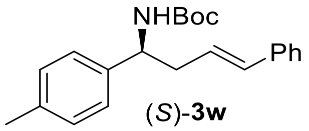

(*S*)-**3w**

Data File E:\DATA\WL\WL-18-88\WL-18-88B-RAC 2017-12-22 00-31-32\WL-18-88.D
Sample Name: WL-18-88B-RAC

```
=====================================================================
Acq. Operator   : SYSTEM                          Seq. Line :   1
Acq. Instrument : 1260                             Location :   8
Injection Date  : 12/22/2017 12:33:07 AM                Inj :   1
                                                 Inj Volume : 5.000 µl
Acq. Method     : E:\DATA\WL\WL-18-88\WL-18-88B-RAC 2017-12-22 00-31-32\ADH-90-10-1.0ML-ALL-
                  210NM-30MIN.M
Last changed    : 12/22/2017 12:48:10 AM by SYSTEM
                  (modified after loading)
Analysis Method : E:\DATA\WL\WL-18-88\WL-18-88B-RAC 2017-12-22 00-31-32\ADH-90-10-1.0ML-ALL-
                  210NM-30MIN.M (Sequence Method)
Last changed    : 5/2/2018 9:03:14 PM by SYSTEM
                  (modified after loading)
Additional Info : Peak(s) manually integrated
```

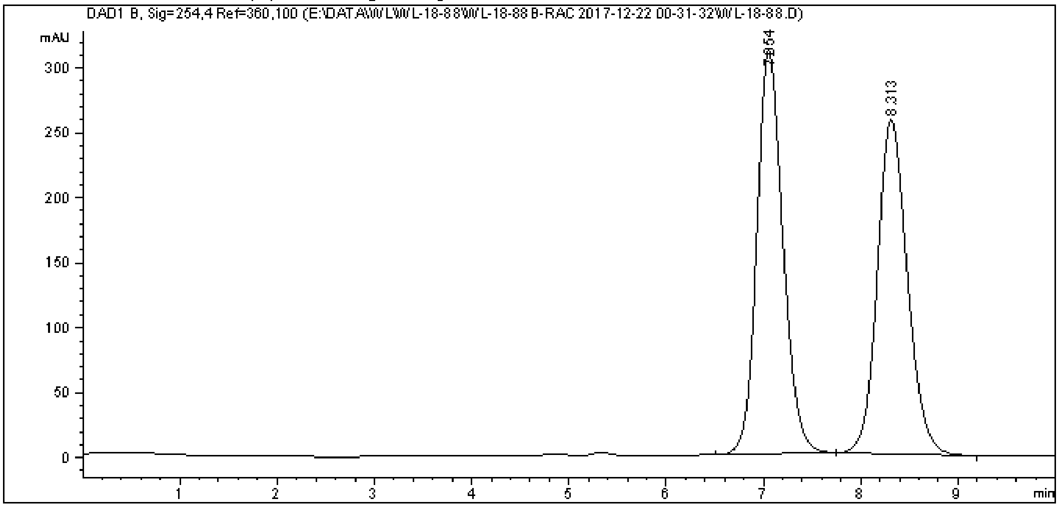


```
=====================================================================
                         Area Percent Report
=====================================================================

Sorted By             :      Signal
Multiplier            :      1.0000
Dilution              :      1.0000
Do not use Multiplier & Dilution Factor with ISTDs


Signal 1: DAD1 B, Sig=254,4 Ref=360,100
```

Peak #	RetTime [min]	Type	Width [min]	Area [mAU*s]	Height [mAU]	Area %
1	7.054	BB	0.2857	5795.20996	310.52237	51.1445
2	8.313	BB	0.3309	5535.85059	257.40891	48.8555

Totals : 1.13311e4 567.93127

===

1260 5/2/2018 9:03:17 PM SYSTEM

Page 1 of 2

Supplementary Figure 160. HPLC spectrum of (*rac*)-**3w**

Data File E:\DATA\WL\WL-18-88\23-OPT 2017-12-29 15-03-16\WL-18-88.D
Sample Name: 2

```
=====================================================================
Acq. Operator   : SYSTEM                        Seq. Line :    1
Acq. Instrument : 1260                           Location :    2
Injection Date  : 12/29/2017 3:04:40 PM               Inj :    1
                                              Inj Volume : 5.000 µl
Acq. Method     : E:\DATA\WL\WL-18-88\23-OPT 2017-12-29 15-03-16\AdH-90-10-254NM-25min.M
Last changed    : 12/29/2017 3:03:32 PM by SYSTEM
                  (modified after loading)
Analysis Method : E:\DATA\WL\WL-18-88\23-OPT 2017-12-29 15-03-16\AdH-90-10-254NM-25min.M (
                  Sequence Method)
Last changed    : 5/2/2018 9:08:00 PM by SYSTEM
                  (modified after loading)
Additional Info : Peak(s) manually integrated
```

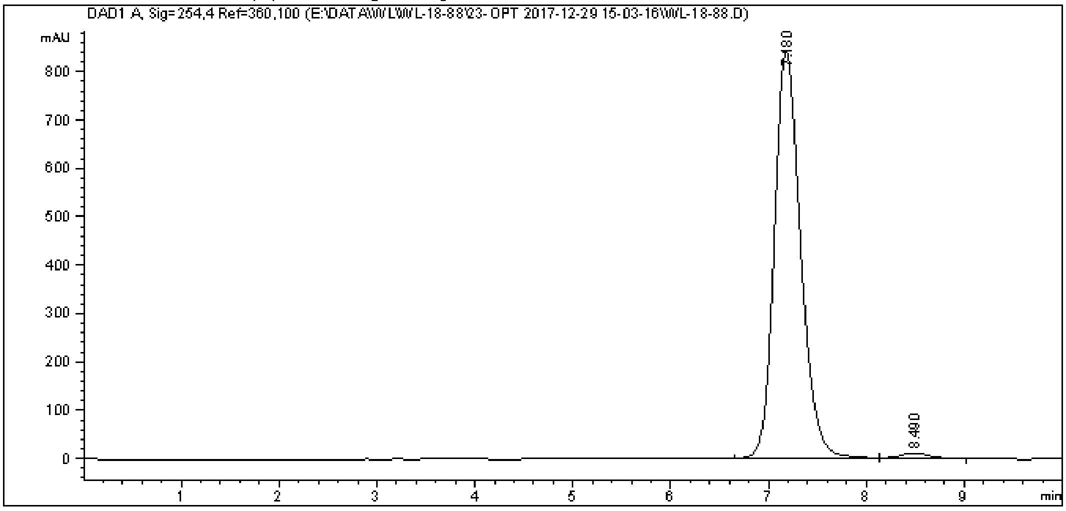


```
=====================================================================
                         Area Percent Report
=====================================================================

Sorted By             :      Signal
Multiplier            :      1.0000
Dilution              :      1.0000
Do not use Multiplier & Dilution Factor with ISTDs


Signal 1: DAD1 A, Sig=254,4 Ref=360,100

Peak RetTime Type  Width     Area      Height     Area
  #   [min]        [min]   [mAU*s]     [mAU]        %
----|-------|----|-------|----------|----------|--------|
   1   7.180 BB    0.2856 1.57184e4  842.71936  98.6382
   2   8.490 BB    0.3114  217.01131   10.44407   1.3618

Totals :                  1.59355e4  853.16343


=====================================================================
                          *** End of Report ***
```

1260 5/2/2018 9:08:02 PM SYSTEM Page 1 of 1

Supplementary Figure 161. HPLC spectrum of (*S*)-**3w**

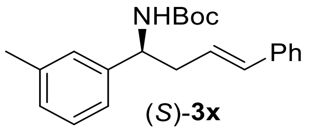

Data File E:\DATA\WL\WL-18-88\WL-18-88B-RAC 2017-12-22 00-31-32\WL-18-882.D
Sample Name: WL-18-88C-RAC

===

Acq. Operator : SYSTEM   Seq. Line : 3
Acq. Instrument : 1260   Location : 9
Injection Date : 12/22/2017 1:06:10 AM   Inj : 1
Inj Volume : 5.000 µl
Acq. Method : E:\DATA\WL\WL-18-88\WL-18-88B-RAC 2017-12-22 00-31-32\ADH-90-10-1.0ML-ALL-210NM-30MIN.M
Last changed : 12/22/2017 12:48:10 AM by SYSTEM
Analysis Method : E:\DATA\WL\WL-18-88\WL-18-88B-RAC 2017-12-22 00-31-32\ADH-90-10-1.0ML-ALL-210NM-30MIN.M (Sequence Method)
Last changed : 5/2/2018 9:03:14 PM by SYSTEM
(modified after loading)
Additional Info : Peak(s) manually integrated

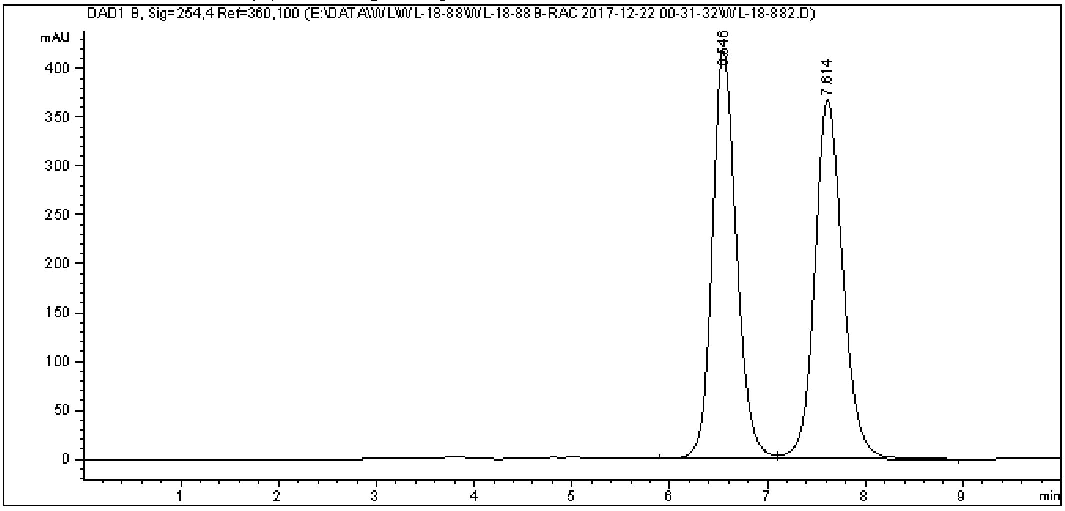

===

Area Percent Report

===

Sorted By : Signal
Multiplier : 1.0000
Dilution : 1.0000
Do not use Multiplier & Dilution Factor with ISTDs

Signal 1: DAD1 B, Sig=254,4 Ref=360,100

Peak #	RetTime [min]	Type	Width [min]	Area [mAU*s]	Height [mAU]	Area %
1	6.546	BV	0.2636	7193.07422	418.37674	49.4998
2	7.614	VB	0.3044	7338.44580	368.40784	50.5002

Totals : 1.45315e4 786.78458

===

*** End of Report ***

1260 5/2/2018 9:03:28 PM SYSTEM   Page 1 of 1

Supplementary Figure 162. HPLC spectrum of (*rac*)-**3x**

Data File E:\DATA\WL\WL-18-88\23-OPT 2017-12-29 15-03-16\WL-18-881.D
Sample Name: 3

===

```
Acq. Operator   : SYSTEM                          Seq. Line :   2
Acq. Instrument : 1260                             Location :   3
Injection Date  : 12/29/2017 3:21:07 PM                 Inj :   1
                                                Inj Volume : 5.000 µl
Acq. Method     : E:\DATA\WL\WL-18-88\23-OPT 2017-12-29 15-03-16\AdH-90-10-254NM-25min.M
Last changed    : 12/29/2017 3:03:32 PM by SYSTEM
Analysis Method : E:\DATA\WL\WL-18-88\23-OPT 2017-12-29 15-03-16\AdH-90-10-254NM-25min.M (
                  Sequence Method)
Last changed    : 5/2/2018 9:08:00 PM by SYSTEM
                  (modified after loading)
Additional Info : Peak(s) manually integrated
```

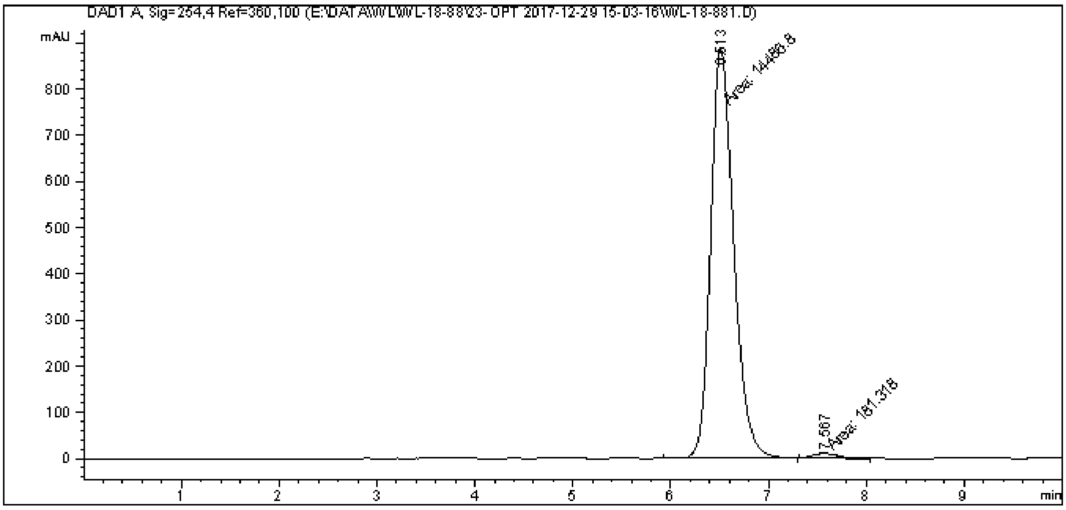

===

Area Percent Report

===

Sorted By : Signal
Multiplier : 1.0000
Dilution : 1.0000
Do not use Multiplier & Dilution Factor with ISTDs

Signal 1: DAD1 A, Sig=254,4 Ref=360,100

Peak #	RetTime [min]	Type	Width [min]	Area [mAU*s]	Height [mAU]	Area %
1	6.513	MM	0.2731	1.44868e4	884.13330	98.7639
2	7.567	MM	0.2948	181.31848	10.25043	1.2361

Totals : 1.46681e4 894.38373

===

*** End of Report ***

1260 5/2/2018 9:09:24 PM SYSTEM Page 1 of 1

Supplementary Figure 163. HPLC spectrum of (*S*)-**3x**

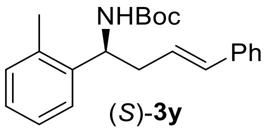

(*S*)-**3y**

Data File E:\DATA\WL\WL-18-88\WL-18-88EFGH-RAC 2017-12-26 15-23-42\WL-18-886.D
Sample Name: WL-18-88G

===
Acq. Operator : SYSTEM Seq. Line : 7
Acq. Instrument : 1260 Location : 6
Injection Date : 12/26/2017 6:23:47 PM Inj : 1
Inj Volume : 5.000 µl
Acq. Method : E:\DATA\WL\WL-18-88\WL-18-88EFGH-RAC 2017-12-26 15-23-42\AdH-90-10-254NM-25min.M
Last changed : 12/26/2017 3:23:42 PM by SYSTEM
Analysis Method : E:\DATA\WL\WL-18-88\WL-18-88EFGH-RAC 2017-12-26 15-23-42\AdH-90-10-254NM-25min.M (Sequence Method)
Last changed : 5/2/2018 9:11:08 PM by SYSTEM
Additional Info : Peak(s) manually integrated

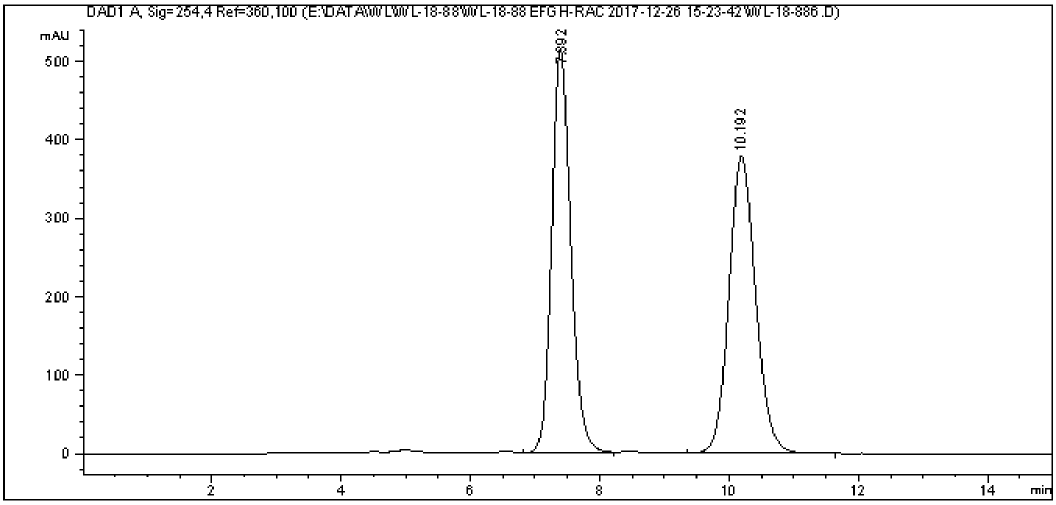

===
Area Percent Report
===

Sorted By : Signal
Multiplier : 1.0000
Dilution : 1.0000
Do not use Multiplier & Dilution Factor with ISTDs

Signal 1: DAD1 A, Sig=254,4 Ref=360,100

Peak #	RetTime [min]	Type	Width [min]	Area [mAU*s]	Height [mAU]	Area %
1	7.392	BB	0.3140	1.05327e4	514.12512	49.1957
2	10.192	BB	0.4444	1.08771e4	378.29578	50.8043

Totals : 2.14097e4 892.42090

===
*** End of Report ***

1260 5/2/2018 9:12:56 PM SYSTEM Page 1 of 1

Supplementary Figure 164. HPLC spectrum of (*rac*)-**3y**

Data File E:\DATA\WL\WL-18-88\WL-18-88DEFG-OPT 2018-01-03 18-53-47\WL-18-883.D
Sample Name: WL-18-88G-OPT

===

```
Acq. Operator   : SYSTEM                          Seq. Line :    4
Acq. Instrument : 1260                             Location :   44
Injection Date  : 1/3/2018 7:44:40 PM                   Inj :    1
                                                 Inj Volume : 5.000 µl
Acq. Method     : E:\DATA\WL\WL-18-88\WL-18-88DEFG-OPT 2018-01-03 18-53-47\AdH-90-10-254NM-
                  25min.M
Last changed    : 1/3/2018 6:54:05 PM by SYSTEM
Analysis Method : E:\DATA\WL\WL-18-88\WL-18-88DEFG-OPT 2018-01-03 18-53-47\AdH-90-10-254NM-
                  25min.M (Sequence Method)
Last changed    : 5/2/2018 9:17:13 PM by SYSTEM
                  (modified after loading)
Additional Info : Peak(s) manually integrated
```

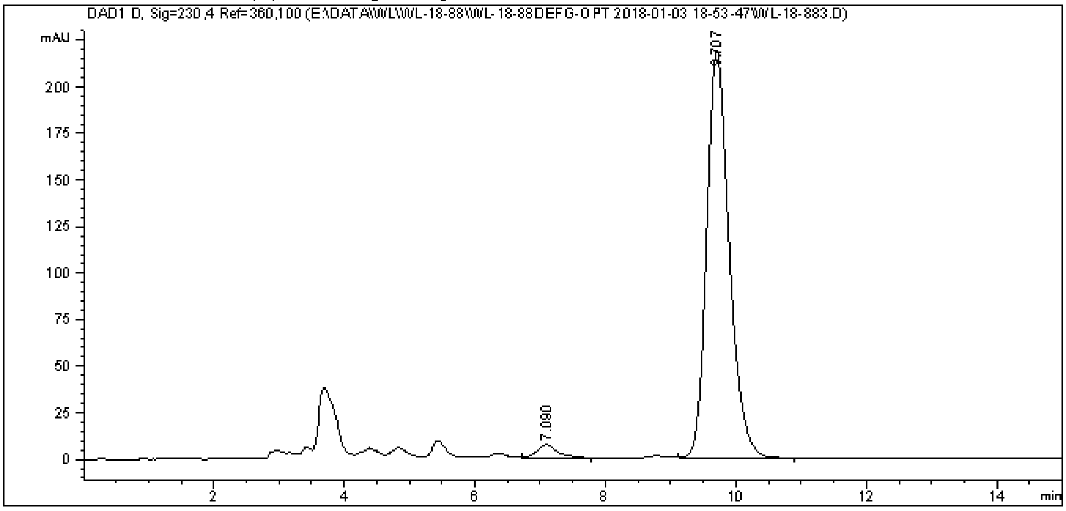

===

Area Percent Report

===

```
Sorted By            :      Signal
Multiplier           :      1.0000
Dilution             :      1.0000
Do not use Multiplier & Dilution Factor with ISTDs
```

Signal 1: DAD1 D, Sig=230,4 Ref=360,100

Peak #	RetTime [min]	Type	Width [min]	Area [mAU*s]	Height [mAU]	Area %
1	7.090	BB	0.2937	149.88013	6.94831	2.7835
2	9.707	BB	0.3634	5234.63281	218.58333	97.2165

Totals : 5384.51294 225.53163

===

*** End of Report ***

1260 5/2/2018 9:18:17 PM SYSTEM Page 1 of 1

Supplementary Figure 165. HPLC spectrum of (*S*)-**3y**

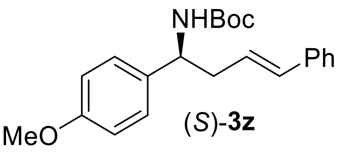

(*S*)-**3z**

Data File E:\DATA\WL\WL-18-88\WL-18-88EFGH-RAC 2017-12-26 15-23-42\WL-18-885.D
Sample Name: WL-18-88F

===

Acq. Operator : SYSTEM Seq. Line : 6
Acq. Instrument : 1260 Location : 5
Injection Date : 12/26/2017 5:57:22 PM Inj : 1
Inj Volume : 5.000 µl
Acq. Method : E:\DATA\WL\WL-18-88\WL-18-88EFGH-RAC 2017-12-26 15-23-42\AdH-90-10-254NM-25min.M
Last changed : 12/26/2017 3:23:42 PM by SYSTEM
Analysis Method : E:\DATA\WL\WL-18-88\WL-18-88EFGH-RAC 2017-12-26 15-23-42\AdH-90-10-254NM-25min.M (Sequence Method)
Last changed : 5/2/2018 9:11:08 PM by SYSTEM
Additional Info : Peak(s) manually integrated

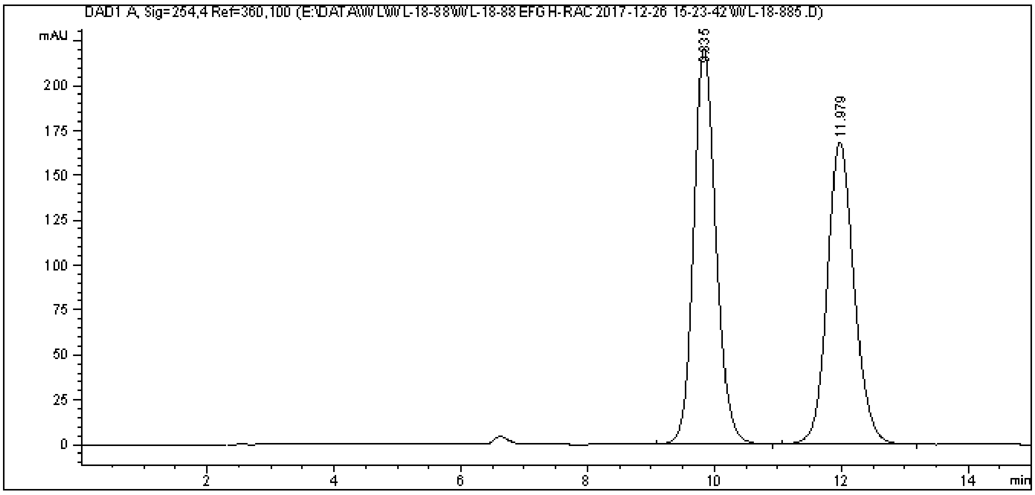

===

Area Percent Report

===

Sorted By : Signal
Multiplier : 1.0000
Dilution : 1.0000
Do not use Multiplier & Dilution Factor with ISTDs

Signal 1: DAD1 A, Sig=254,4 Ref=360,100

Peak #	RetTime [min]	Type	Width [min]	Area [mAU*s]	Height [mAU]	Area %
1	9.835	BB	0.3686	5378.60303	220.50105	51.9339
2	11.979	BB	0.4531	4978.02637	168.31290	48.0661

Totals : 1.03566e4 388.81395

===

*** End of Report ***

1260 5/2/2018 9:15:55 PM SYSTEM Page 1 of 1

Supplementary Figure 166. HPLC spectrum of (*rac*)-**3z**

Data File E:\DATA\WL\WL-18-88\WL-18-88DEFG-OPT 2018-01-03 18-53-47\WL-18-882.D
Sample Name: WL-18-88F-OPT

===

Acq. Operator : SYSTEM Seq. Line : 3
Acq. Instrument : 1260 Location : 43
Injection Date : 1/3/2018 7:28:07 PM Inj : 1
Inj Volume : 5.000 µl
Acq. Method : E:\DATA\WL\WL-18-88\WL-18-88DEFG-OPT 2018-01-03 18-53-47\AdH-90-10-254NM-25min.M
Last changed : 1/3/2018 6:54:05 PM by SYSTEM
Analysis Method : E:\DATA\WL\WL-18-88\WL-18-88DEFG-OPT 2018-01-03 18-53-47\AdH-90-10-254NM-25min.M (Sequence Method)
Last changed : 5/2/2018 9:17:13 PM by SYSTEM
(modified after loading)
Additional Info : Peak(s) manually integrated

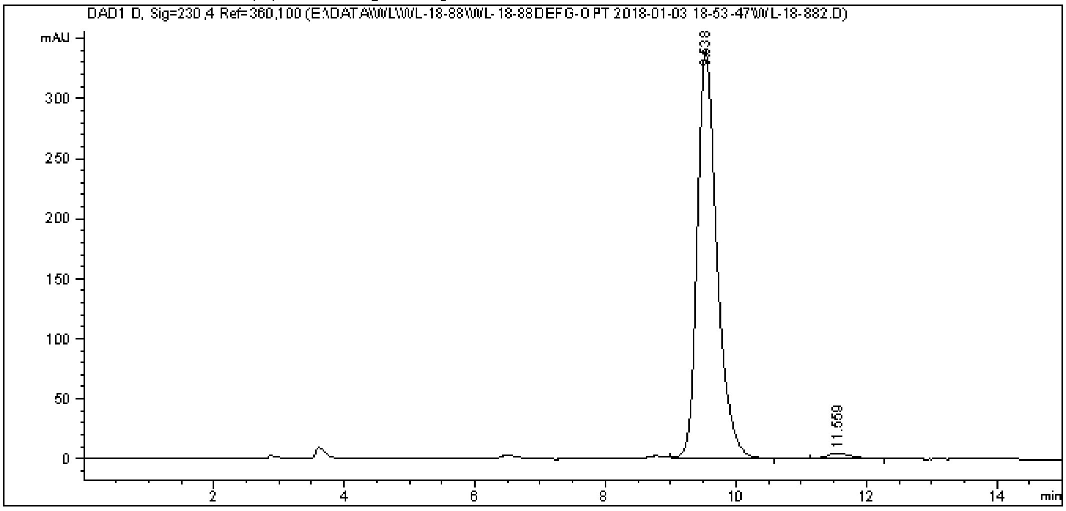

===

Area Percent Report

===

Sorted By : Signal
Multiplier : 1.0000
Dilution : 1.0000
Do not use Multiplier & Dilution Factor with ISTDs

Signal 1: DAD1 D, Sig=230,4 Ref=360,100

Peak #	RetTime [min]	Type	Width [min]	Area [mAU*s]	Height [mAU]	Area %
1	9.538	BB	0.3173	7100.23242	339.03854	98.2971
2	11.559	BB	0.3047	123.00558	4.91991	1.7029

Totals : 7223.23801 343.95846

===

*** End of Report ***

1260 5/2/2018 9:17:55 PM SYSTEM Page 1 of 1

Supplementary Figure 167. HPLC spectrum of (*S*)-**3z**

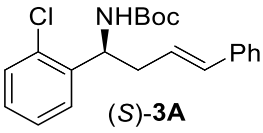

(*S*)-**3A**

```
Data File E:\DATA\ZQ\ZQ-10-47\ZQ-10-47 2018-01-08 15-32-05\CC-3-70-OPT1.D
Sample Name: ZQ-10-47-oCl-rac

    ====================================================================
    Acq. Operator   : SYSTEM                        Seq. Line :   2
    Acq. Instrument : 1260                           Location :   74
    Injection Date  : 1/8/2018 3:49:04 PM                 Inj :   1
                                                   Inj Volume : 5.000 µl
    Acq. Method     : E:\DATA\ZQ\ZQ-10-47\ZQ-10-47 2018-01-08 15-32-05\OJH-90-10-1.0ML-ALL-254NM-
                      30MIN.M
    Last changed    : 1/8/2018 4:08:50 PM by SYSTEM
                      (modified after loading)
    Analysis Method : E:\DATA\ZQ\ZQ-10-47\ZQ-10-47 2018-01-08 15-32-05\OJH-90-10-1.0ML-ALL-254NM-
                      30MIN.M (Sequence Method)
    Last changed    : 4/11/2018 3:19:52 PM by SYSTEM
                      (modified after loading)
    Additional Info : Peak(s) manually integrated
```

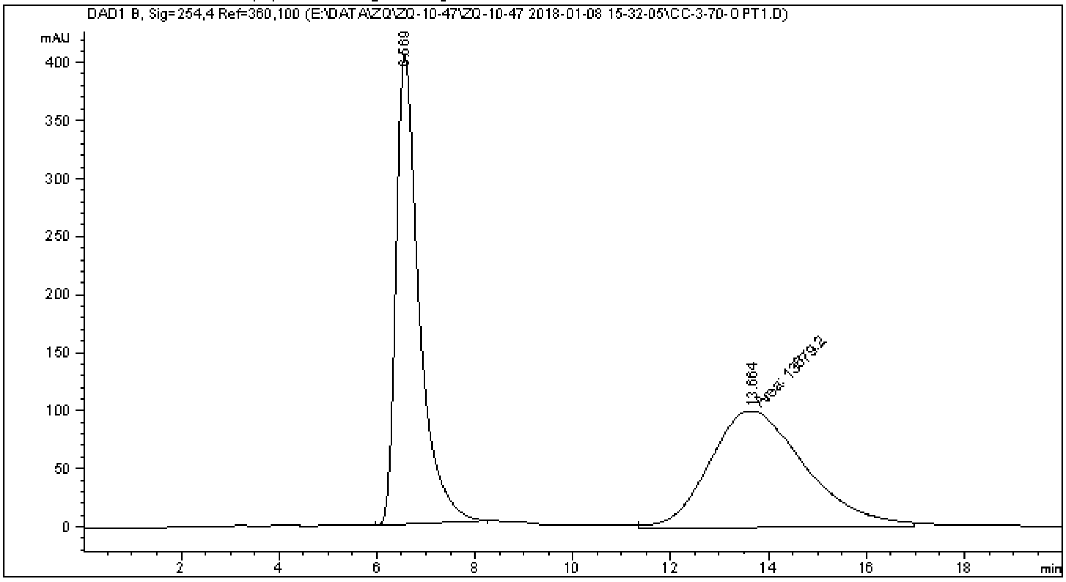


```
    ====================================================================
                            Area Percent Report
    ====================================================================

    Sorted By             :      Signal
    Multiplier            :      1.0000
    Dilution              :      1.0000
    Do not use Multiplier & Dilution Factor with ISTDs


    Signal 1: DAD1 B, Sig=254,4 Ref=360,100
```

Peak #	RetTime [min]	Type	Width [min]	Area [mAU*s]	Height [mAU]	Area %
1	6.569	BB	0.4907	1.33217e4	405.01218	49.3379
2	13.664	MM	2.2686	1.36792e4	100.49751	50.6621
Totals :				2.70009e4	505.50969	

Supplementary Figure 168. HPLC spectrum of (*rac*)-**3A**

```
Data File E:\DATA\ZQ\ZQ-10-47\ZQ-10-47 2018-01-08 15-32-05\CC-3-70-OPT2.D
Sample Name: o-Cl-opt

    =====================================================================
    Acq. Operator   : SYSTEM                        Seq. Line :    3
    Acq. Instrument : 1260                           Location :   76
    Injection Date  : 1/8/2018 4:10:36 PM                 Inj :    1
                                                   Inj Volume : 5.000 µl
    Acq. Method     : E:\DATA\ZQ\ZQ-10-47\ZQ-10-47 2018-01-08 15-32-05\OJH-90-10-1.0ML-ALL-254NM-
                      30MIN.M
    Last changed    : 1/8/2018 4:08:50 PM by SYSTEM
    Analysis Method : E:\DATA\ZQ\ZQ-10-47\ZQ-10-47 2018-01-08 15-32-05\OJH-90-10-1.0ML-ALL-254NM-
                      30MIN.M (Sequence Method)
    Last changed    : 4/11/2018 3:15:21 PM by SYSTEM
                      (modified after loading)
    Additional Info : Peak(s) manually integrated
```

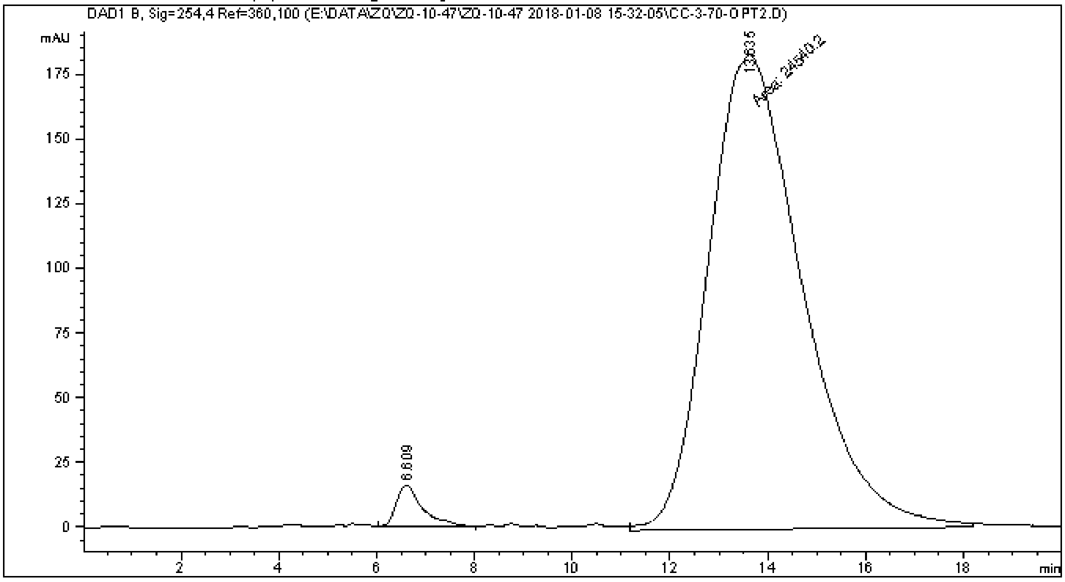


```
    =====================================================================
                         Area Percent Report
    =====================================================================

    Sorted By           :      Signal
    Multiplier          :      1.0000
    Dilution            :      1.0000
    Do not use Multiplier & Dilution Factor with ISTDs


    Signal 1: DAD1 B, Sig=254,4 Ref=360,100
```

Peak #	RetTime [min]	Type	Width [min]	Area [mAU*s]	Height [mAU]	Area %
1	6.609	BB	0.4792	592.15137	15.91209	2.3561
2	13.635	MM	2.2282	2.45402e4	183.55688	97.6439

Totals : 2.51324e4 199.46897

Supplementary Figure 169. HPLC spectrum of (*S*)-**3A**

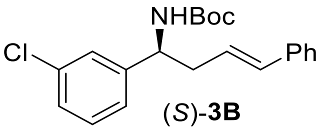

(*S*)-**3B**

Data File E:\DATA\WL\WL-18-88\WL-18-88H-RAC 2017-12-26 21-25-37\WL-18-883.D
Sample Name: WL-18-88H

===
Acq. Operator : SYSTEM Seq. Line : 4
Acq. Instrument : 1260 Location : 7
Injection Date : 12/26/2017 10:31:36 PM Inj : 1
Inj Volume : 5.000 µl
Acq. Method : E:\DATA\WL\WL-18-88\WL-18-88H-RAC 2017-12-26 21-25-37\OJH-90-10-1.0ML-ALL-254NM-30MIN.M
Last changed : 12/26/2017 9:25:37 PM by SYSTEM
Analysis Method : E:\DATA\WL\WL-18-88\WL-18-88H-RAC 2017-12-26 21-25-37\OJH-90-10-1.0ML-ALL-254NM-30MIN.M (Sequence Method)
Last changed : 5/2/2018 9:19:50 PM by SYSTEM
(modified after loading)
Additional Info : Peak(s) manually integrated

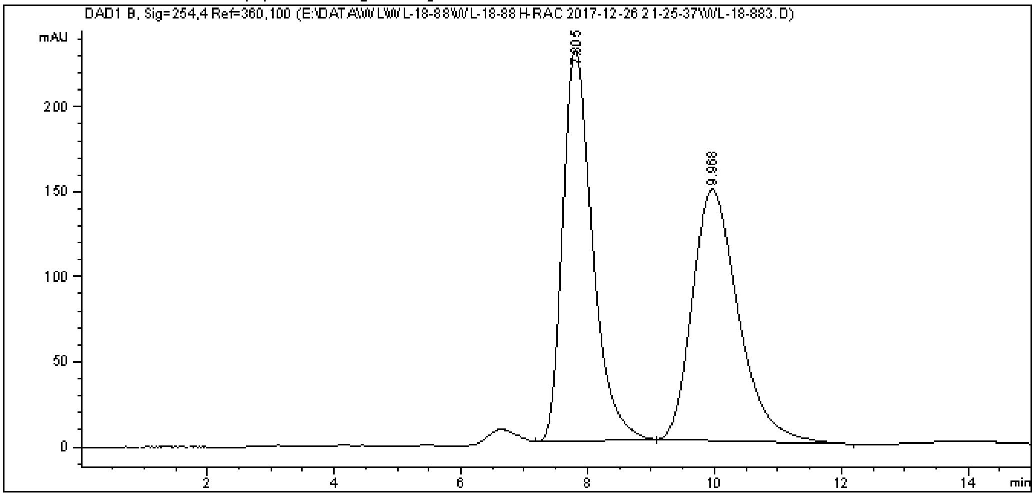

===
Area Percent Report
===

Sorted By : Signal
Multiplier : 1.0000
Dilution : 1.0000
Do not use Multiplier & Dilution Factor with ISTDs

Signal 1: DAD1 B, Sig=254,4 Ref=360,100

Peak #	RetTime [min]	Type	Width [min]	Area [mAU*s]	Height [mAU]	Area %
1	7.805	BB	0.4955	7505.48926	230.11427	50.1348
2	9.968	BB	0.7414	7465.13232	147.75601	49.8652
Totals :				1.49706e4	377.87029	

===
*** End of Report ***

1260 5/2/2018 9:19:54 PM SYSTEM Page 1 of 1

Supplementary Figure 170. HPLC spectrum of (*rac*)-**3B**

Data File E:\DATA\WL\WL-18-88\WL-18-88H-OPT 2018-01-05 12-07-10\WL-18-881.D
Sample Name: WL-18-88H-OPT

===

Acq. Operator : SYSTEM Seq. Line : 2
Acq. Instrument : 1260 Location : 47
Injection Date : 1/5/2018 12:24:35 PM Inj : 1
Inj Volume : 5.000 µl
Acq. Method : E:\DATA\WL\WL-18-88\WL-18-88H-OPT 2018-01-05 12-07-10\OJH-90-10-1.0ML-ALL-254NM-30MIN.M
Last changed : 1/5/2018 12:07:20 PM by SYSTEM
Analysis Method : E:\DATA\WL\WL-18-88\WL-18-88H-OPT 2018-01-05 12-07-10\OJH-90-10-1.0ML-ALL-254NM-30MIN.M (Sequence Method)
Last changed : 5/2/2018 9:20:31 PM by SYSTEM
(modified after loading)
Additional Info : Peak(s) manually integrated

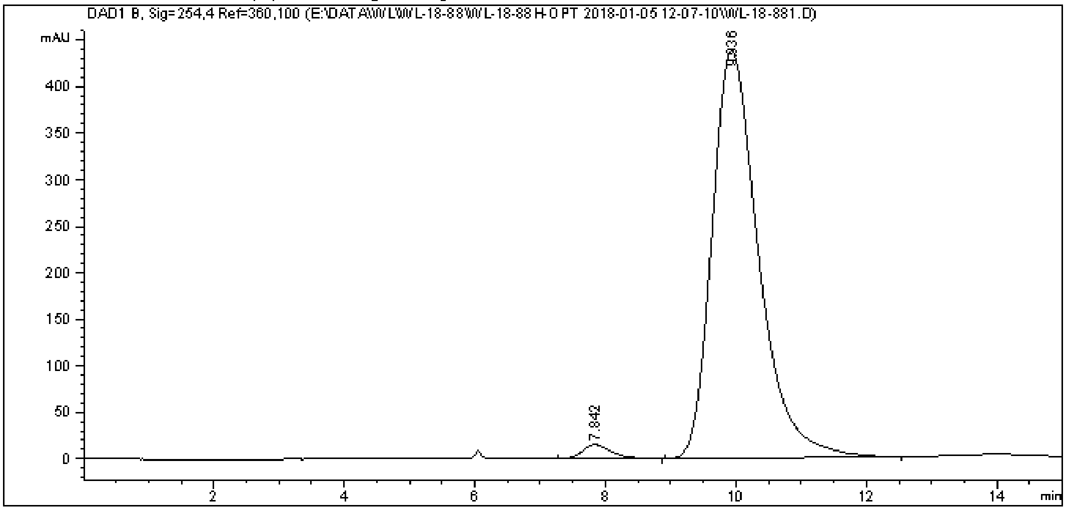

===

Area Percent Report

===

Sorted By : Signal
Multiplier : 1.0000
Dilution : 1.0000
Do not use Multiplier & Dilution Factor with ISTDs

Signal 1: DAD1 B, Sig=254,4 Ref=360,100

Peak #	RetTime [min]	Type	Width [min]	Area [mAU*s]	Height [mAU]	Area %
1	7.842	BB	0.4241	465.73828	15.04795	2.0999
2	9.936	BB	0.7508	2.17135e4	437.63889	97.9001

Totals : 2.21792e4 452.68684

===

*** End of Report ***

1260 5/2/2018 9:20:34 PM SYSTEM Page 1 of 1

Supplementary Figure 171. HPLC spectrum of (*S*)-**3B**

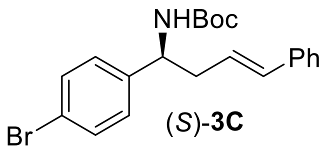

(*S*)-**3C**

Data File E:\DATA\WL\WL-18-88\WL-18088ABCD-RAC 2017-12-21 19-56-34\WL-18-88.D
Sample Name: WL-18-88A-RAC

===

Acq. Operator : SYSTEM Seq. Line : 1
Acq. Instrument : 1260 Location : 7
Injection Date : 12/21/2017 7:58:03 PM Inj : 1
Inj Volume : 5.000 µl
Acq. Method : E:\DATA\WL\WL-18-88\WL-18088ABCD-RAC 2017-12-21 19-56-34\ODH-90-10-1.0ML-ALL-254NM-30MIN.M
Last changed : 12/21/2017 7:56:48 PM by SYSTEM
(modified after loading)
Analysis Method : E:\DATA\WL\WL-18-88\WL-18088ABCD-RAC 2017-12-21 19-56-34\ODH-90-10-1.0ML-ALL-254NM-30MIN.M (Sequence Method)
Last changed : 5/2/2018 9:00:14 PM by SYSTEM
(modified after loading)
Additional Info : Peak(s) manually integrated

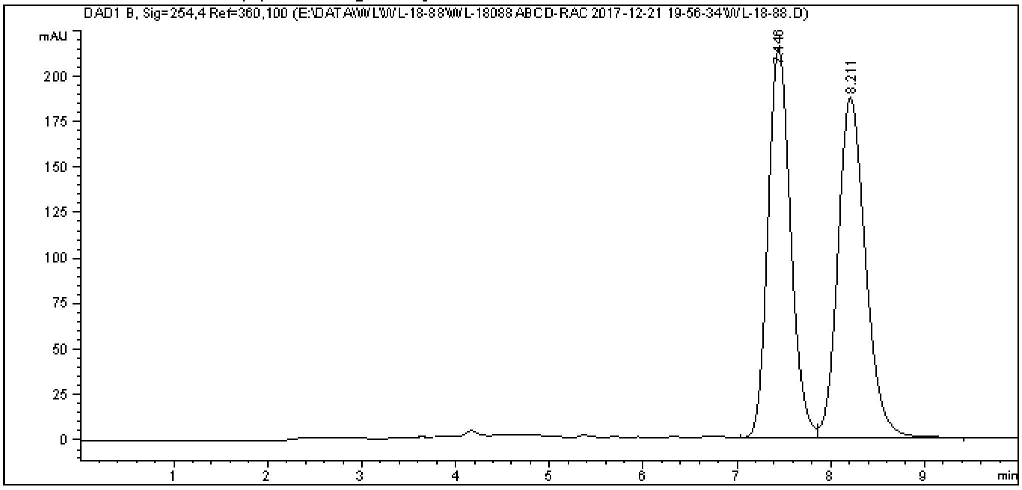

===

Area Percent Report

===

Sorted By : Signal
Multiplier : 1.0000
Dilution : 1.0000
Do not use Multiplier & Dilution Factor with ISTDs

Signal 1: DAD1 B, Sig=254,4 Ref=360,100

Peak #	RetTime [min]	Type	Width [min]	Area [mAU*s]	Height [mAU]	Area %
1	7.446	BV	0.2603	3592.83154	213.57677	48.3717
2	8.211	VB	0.3176	3834.71729	186.72264	51.6283
Totals :				7427.54883	400.29941	

===

1260 5/2/2018 9:00:16 PM SYSTEM Page 1 of 2

Supplementary Figure 172. HPLC spectrum of (*rac*)-**3C**

Data File E:\DATA\WL\WL-18-88\123T 2017-12-29 13-29-08\WL-18-88.D
Sample Name: 1

===

Acq. Operator : SYSTEM Seq. Line : 1
Acq. Instrument : 1260 Location : 1
Injection Date : 12/29/2017 1:30:33 PM Inj : 1
Inj Volume : 5.000 µl
Acq. Method : E:\DATA\WL\WL-18-88\123t 2017-12-29 13-29-08\ODH-90-10-1.0ML-ALL-254NM-30MIN.M
Last changed : 12/29/2017 1:30:51 PM by SYSTEM
(modified after loading)
Analysis Method : E:\DATA\WL\WL-18-88\123t 2017-12-29 13-29-08\ODH-90-10-1.0ML-ALL-254NM-30MIN.M (Sequence Method)
Last changed : 5/2/2018 9:05:28 PM by SYSTEM
(modified after loading)
Additional Info : Peak(s) manually integrated

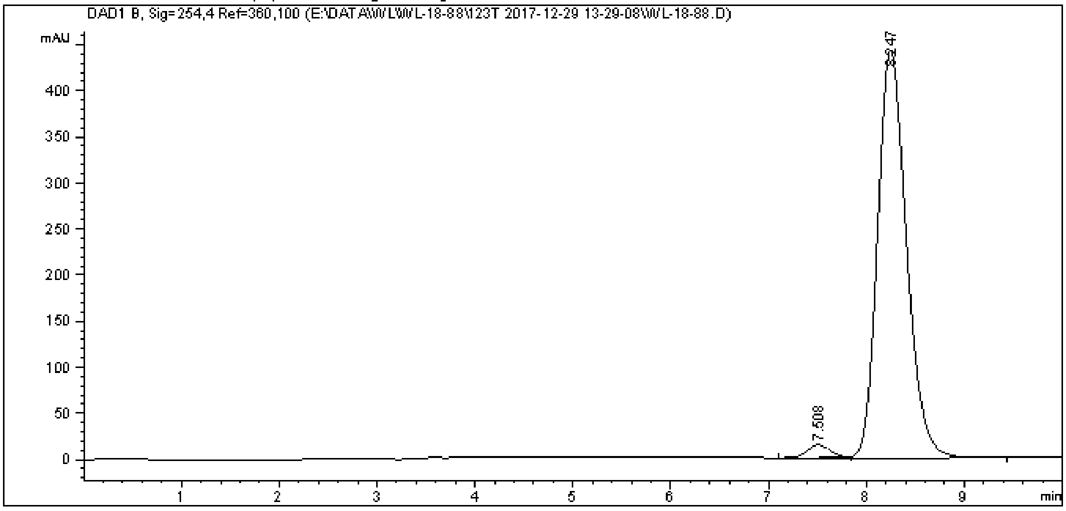

===

Area Percent Report

===

Sorted By : Signal
Multiplier : 1.0000
Dilution : 1.0000
Do not use Multiplier & Dilution Factor with ISTDs

Signal 1: DAD1 B, Sig=254,4 Ref=360,100

Peak #	RetTime [min]	Type	Width [min]	Area [mAU*s]	Height [mAU]	Area %
1	7.508	BV E	0.2511	231.87175	14.08520	2.4971
2	8.247	VB R	0.3152	9053.68945	441.62375	97.5029

Totals : 9285.56120 455.70895

===

1260 5/2/2018 9:05:33 PM SYSTEM Page 1 of 2

Supplementary Figure 173. HPLC spectrum of (*S*)-**3C**

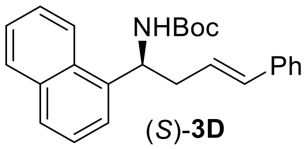

(*S*)-**3D**

Data File E:\DATA\ZQ\ZQ-10-47\ZQ-10-47 2018-01-05 16-00-07\cc-3-67-opt.D
Sample Name: ZQ-10-47-I-nap-rac

===

Acq. Operator : SYSTEM Seq. Line : 1
Acq. Instrument : 1260 Location : 73
Injection Date : 1/5/2018 4:01:34 PM Inj : 1
Inj Volume : 5.000 µl
Acq. Method : E:\DATA\ZQ\ZQ-10-47\ZQ-10-47 2018-01-05 16-00-07\AD-90-10-254NM-20MIN.M
Last changed : 1/5/2018 4:00:07 PM by SYSTEM
Analysis Method : E:\DATA\ZQ\ZQ-10-47\ZQ-10-47 2018-01-05 16-00-07\AD-90-10-254NM-20MIN.M (Sequence Method)
Last changed : 4/11/2018 2:11:58 PM by SYSTEM
(modified after loading)
Additional Info : Peak(s) manually integrated

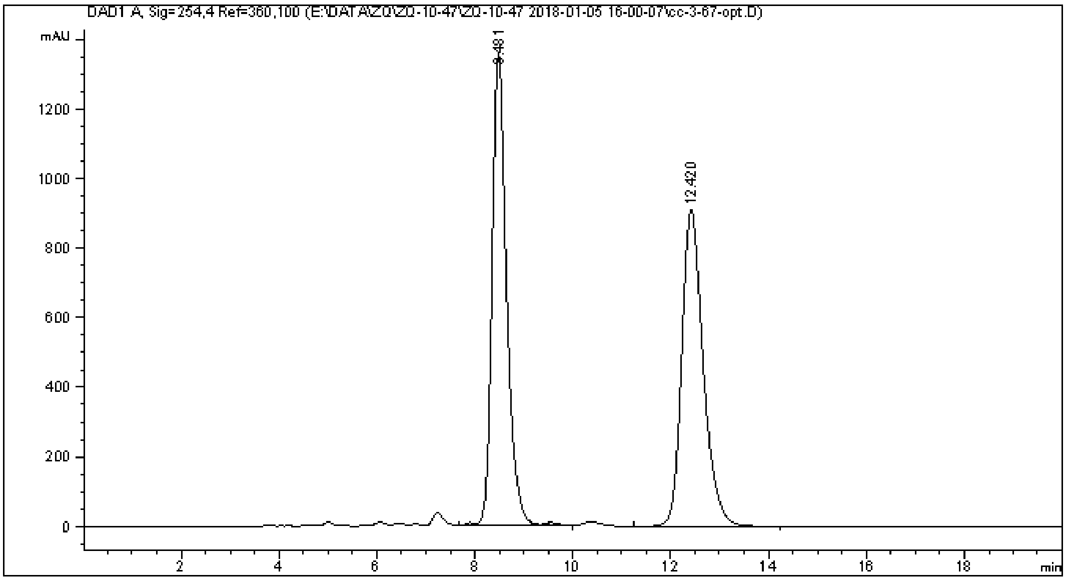

===
Area Percent Report
===

Sorted By : Signal
Multiplier : 1.0000
Dilution : 1.0000
Do not use Multiplier & Dilution Factor with ISTDs

Signal 1: DAD1 A, Sig=254,4 Ref=360,100

Peak #	RetTime [min]	Type	Width [min]	Area [mAU*s]	Height [mAU]	Area %
1	8.481	VV R	0.3086	2.81836e4	1359.59595	50.3671
2	12.420	BB	0.4601	2.77728e4	909.83008	49.6329

Totals : 5.59564e4 2269.42603

1260 4/11/2018 2:12:02 PM SYSTEM Page 1 of 2

Supplementary Figure 174. HPLC spectrum of (*rac*)-**3D**

Data File E:\DATA\ZQ\ZQ-10-47\ZQ-10-47 2018-01-05 16-39-34\cc-3-67-opt.D
Sample Name: ZQ-10-47-I-nap-opt

===

Acq. Operator : SYSTEM Seq. Line : 1
Acq. Instrument : 1260 Location : 75
Injection Date : 1/5/2018 4:41:08 PM Inj : 1
Inj Volume : 5.000 µl
Acq. Method : E:\DATA\ZQ\ZQ-10-47\ZQ-10-47 2018-01-05 16-39-34\AD-90-10-254NM-20MIN.M
Last changed : 1/5/2018 4:39:34 PM by SYSTEM
Analysis Method : E:\DATA\ZQ\ZQ-10-47\ZQ-10-47 2018-01-05 16-39-34\AD-90-10-254NM-20MIN.M (Sequence Method)
Last changed : 4/11/2018 2:14:06 PM by SYSTEM
(modified after loading)
Additional Info : Peak(s) manually integrated

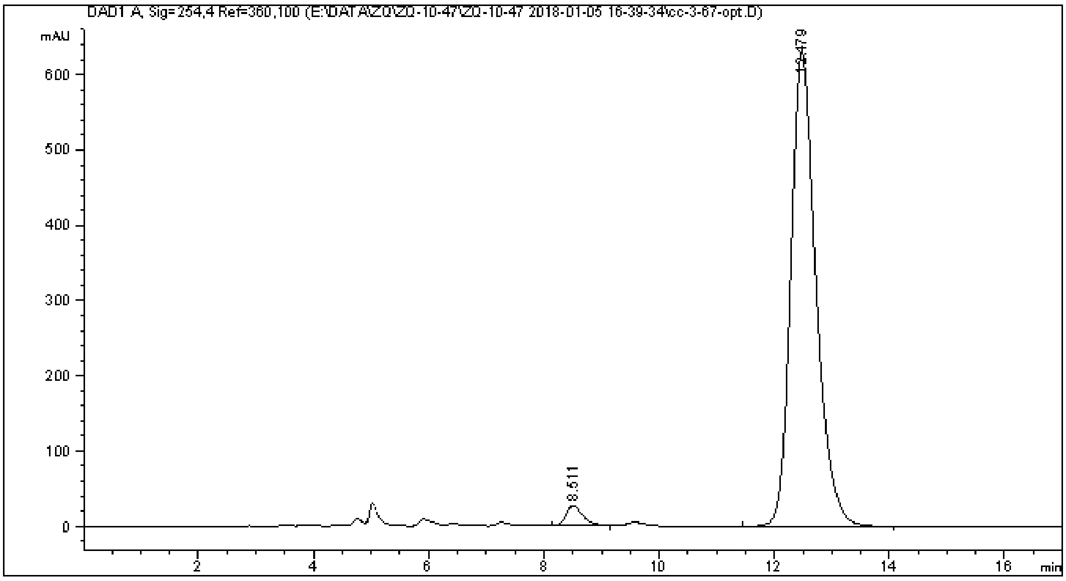

===

Area Percent Report

===

Sorted By : Signal
Multiplier : 1.0000
Dilution : 1.0000
Do not use Multiplier & Dilution Factor with ISTDs

Signal 1: DAD1 A, Sig=254,4 Ref=360,100

Peak #	RetTime [min]	Type	Width [min]	Area [mAU*s]	Height [mAU]	Area %
1	8.511	BB	0.2918	514.60846	26.47277	2.6269
2	12.479	BB	0.4588	1.90751e4	629.01294	97.3731

Totals : 1.95897e4 655.48571

1260 4/11/2018 2:14:10 PM SYSTEM Page 1 of 2

Supplementary Figure 175. HPLC spectrum of (*S*)-**3D**

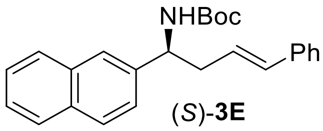

(*S*)-**3E**

```
Data File E:\DATA\WL\WL-18-88\2-NAP 2018-05-05 15-45-06\WL-18-88.D
Sample Name: 2-Nap-rac

    =====================================================================
    Acq. Operator   : SYSTEM                        Seq. Line :    1
    Acq. Instrument : 1260                           Location :   36
    Injection Date  : 5/5/2018 3:46:33 PM                 Inj :    1
                                                Inj Volume : 5.000 µl
    Acq. Method     : E:\DATA\WL\WL-18-88\2-Nap 2018-05-05 15-45-06\ADH-90-10-1.0ML-ALL-210NM-
                      30MIN.M
    Last changed    : 5/5/2018 3:46:14 PM by SYSTEM
                      (modified after loading)
    Analysis Method : E:\DATA\WL\WL-18-88\2-Nap 2018-05-05 15-45-06\ADH-90-10-1.0ML-ALL-210NM-
                      30MIN.M (Sequence Method)
    Last changed    : 5/5/2018 8:45:52 PM by SYSTEM
                      (modified after loading)
    Additional Info : Peak(s) manually integrated
```

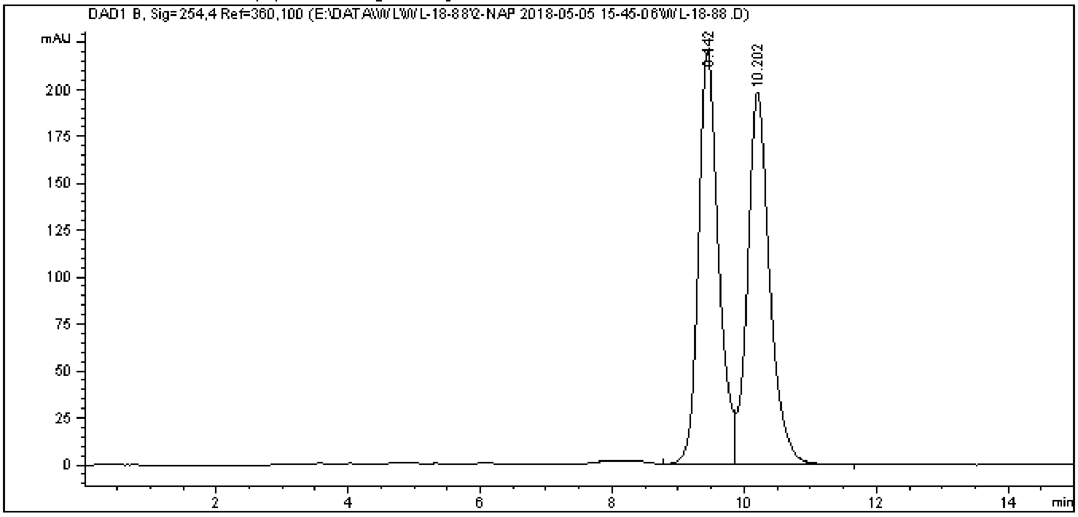


```
    =====================================================================
                             Area Percent Report
    =====================================================================

    Sorted By             :      Signal
    Multiplier            :      1.0000
    Dilution              :      1.0000
    Do not use Multiplier & Dilution Factor with ISTDs


    Signal 1: DAD1 B, Sig=254,4 Ref=360,100

    Peak RetTime Type  Width     Area      Height     Area
      #   [min]        [min]   [mAU*s]     [mAU]        %
    ----|-------|----|-------|----------|----------|--------|
       1   9.442 BV    0.3195 4646.07959  219.87427  50.1449
       2  10.202 VB    0.3487 4619.23145  198.31346  49.8551

    Totals :                  9265.31104  418.18773


    =====================================================================
```


Supplementary Figure 176. HPLC spectrum of (*rac*)-**3a**

Data File E:\DATA\WL\WL-18-88\2-Nap 2018-05-05 15-45-06\WL-18-881.D
Sample Name: 2-Nap-opt

===

Acq. Operator : SYSTEM Seq. Line : 2
Acq. Instrument : 1260 Location : 37
Injection Date : 5/5/2018 4:03:15 PM Inj : 1
Inj Volume : 5.000 µl
Acq. Method : E:\DATA\WL\WL-18-88\2-Nap 2018-05-05 15-45-06\ADH-90-10-1.0ML-ALL-210NM-30MIN.M
Last changed : 5/5/2018 3:46:14 PM by SYSTEM
Analysis Method : E:\DATA\WL\WL-18-88\2-Nap 2018-05-05 15-45-06\ADH-90-10-1.0ML-ALL-210NM-30MIN.M (Sequence Method)
Last changed : 5/5/2018 8:45:52 PM by SYSTEM
(modified after loading)
Additional Info : Peak(s) manually integrated

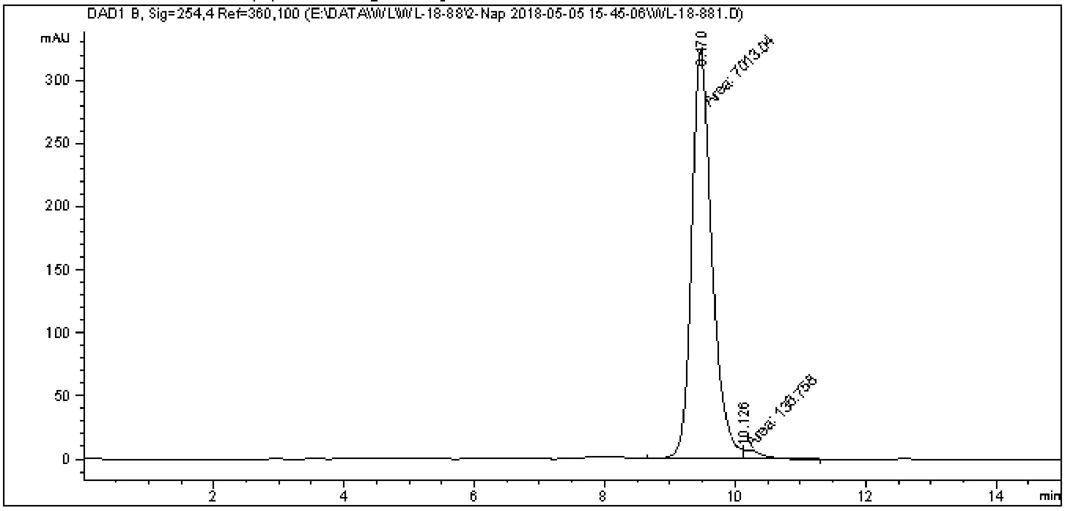

===

Area Percent Report

===

Sorted By : Signal
Multiplier : 1.0000
Dilution : 1.0000
Do not use Multiplier & Dilution Factor with ISTDs

Signal 1: DAD1 B, Sig=254,4 Ref=360,100

Peak #	RetTime [min]	Type	Width [min]	Area [mAU*s]	Height [mAU]	Area %
1	9.470	MF	0.3620	7013.03809	322.89252	98.0872
2	10.126	FM	0.3105	136.75774	7.34056	1.9128

Totals : 7149.79582 330.23308

===

*** End of Report ***

1260 5/5/2018 8:46:28 PM SYSTEM Page 1 of 1

Supplementary Figure 177. HPLC spectrum of (*S*)-**3E**

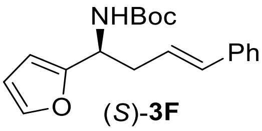

(*S*)-**3F**

Data File E:\DATA\WL\WL-18-88\WL-18-88KLM-RAC 2018-01-06 23-01-18\WL-18-88.D
Sample Name: WL-18-88K

===

Acq. Operator : SYSTEM Seq. Line : 1
Acq. Instrument : 1260 Location : 41
Injection Date : 1/6/2018 11:02:39 PM Inj : 1
Inj Volume : 5.000 µl
Acq. Method : E:\DATA\WL\WL-18-88\WL-18-88KLM-RAC 2018-01-06 23-01-18\ODH-90-10-1.0ML-ALL -254NM-30MIN.M
Last changed : 1/6/2018 11:27:57 PM by SYSTEM (modified after loading)
Analysis Method : E:\DATA\WL\WL-18-88\WL-18-88KLM-RAC 2018-01-06 23-01-18\ODH-90-10-1.0ML-ALL -254NM-30MIN.M (Sequence Method)
Last changed : 5/2/2018 9:24:09 PM by SYSTEM (modified after loading)
Additional Info : Peak(s) manually integrated

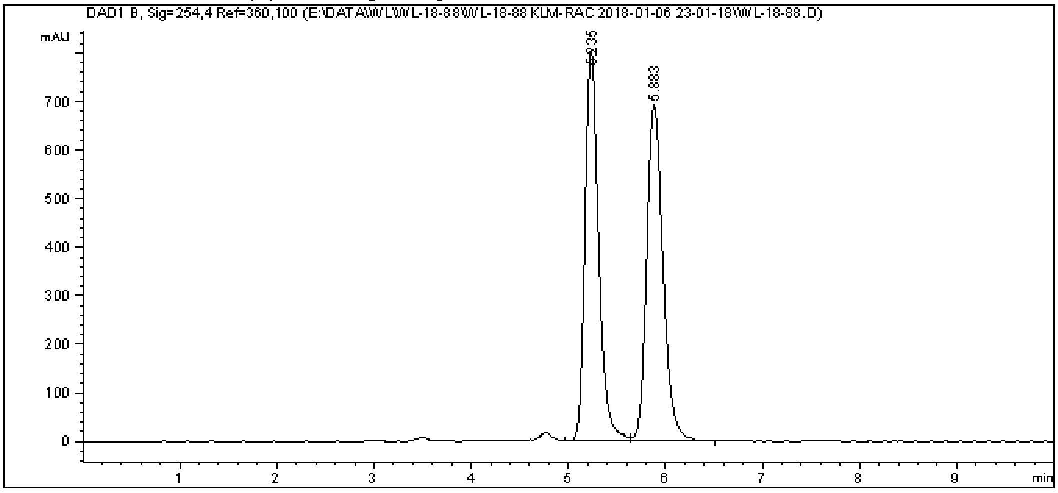

===

Area Percent Report

===

Sorted By : Signal
Multiplier : 1.0000
Dilution : 1.0000
Do not use Multiplier & Dilution Factor with ISTDs

Signal 1: DAD1 B, Sig=254,4 Ref=360,100

Peak #	RetTime [min]	Type	Width [min]	Area [mAU*s]	Height [mAU]	Area %
1	5.235	BV	0.1489	7860.16943	805.24335	49.3522
2	5.883	VV R	0.1792	8066.52637	690.88824	50.6478

Totals : 1.59267e4 1496.13159

===

1260 5/2/2018 9:24:12 PM SYSTEM Page 1 of 2

Supplementary Figure 178. HPLC spectrum of (*rac*)-**3F**

Data File E:\DATA\WL\WL-18-88\WL-18-88KLM-RAC 2018-01-06 23-01-18\WL-18-881.D
Sample Name: WL-18-88L

===

Acq. Operator : SYSTEM Seq. Line : 2
Acq. Instrument : 1260 Location : 43
Injection Date : 1/6/2018 11:30:01 PM Inj : 1
Inj Volume : 5.000 µl
Acq. Method : E:\DATA\WL\WL-18-88\WL-18-88KLM-RAC 2018-01-06 23-01-18\ODH-90-10-1.0ML-ALL -254NM-30MIN.M
Last changed : 1/6/2018 11:48:26 PM by SYSTEM (modified after loading)
Analysis Method : E:\DATA\WL\WL-18-88\WL-18-88KLM-RAC 2018-01-06 23-01-18\ODH-90-10-1.0ML-ALL -254NM-30MIN.M (Sequence Method)
Last changed : 5/2/2018 9:26:35 PM by SYSTEM (modified after loading)
Additional Info : Peak(s) manually integrated

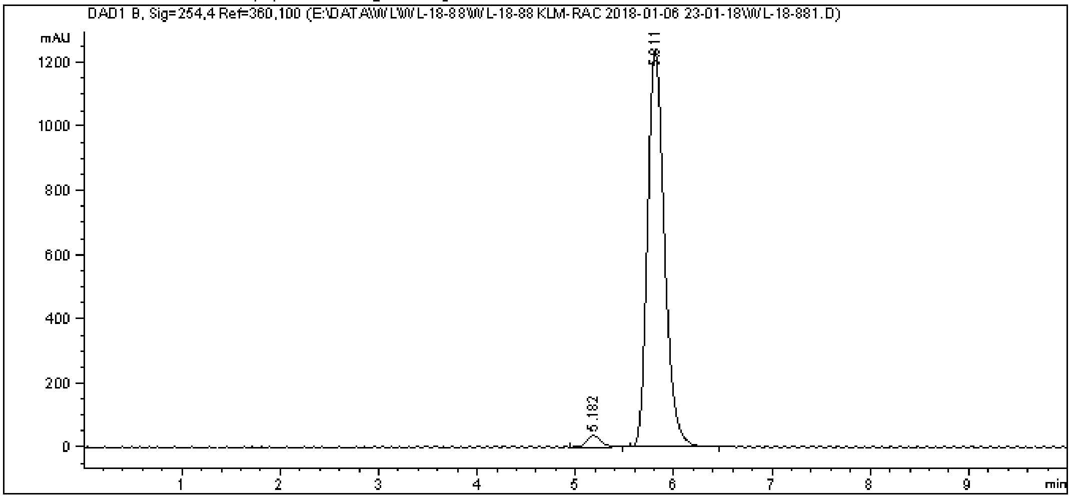

===

Area Percent Report

===

Sorted By : Signal
Multiplier : 1.0000
Dilution : 1.0000
Do not use Multiplier & Dilution Factor with ISTDs

Signal 1: DAD1 B, Sig=254,4 Ref=360,100

Peak #	RetTime [min]	Type	Width [min]	Area [mAU*s]	Height [mAU]	Area %
1	5.182	VV R	0.1422	382.11160	36.87556	2.5190
2	5.811	BV R	0.1845	1.47870e4	1236.54138	97.4810

Totals : 1.51691e4 1273.41695

===

1260 5/2/2018 9:26:37 PM SYSTEM Page 1 of 2

Supplementary Figure 179. HPLC spectrum of (*S*)-**3F**

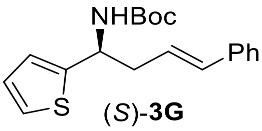

(*S*)-**3G**

Data File E:\DATA\ZQ\ZQ-10-47\ZQ-10-47 2018-01-09 12-09-18\WL-18-88M-RAC.D
Sample Name: ZQ-10-47-thenyl-rac

===

Acq. Operator : SYSTEM   Seq. Line : 1
Acq. Instrument : 1260   Location : 71
Injection Date : 1/9/2018 12:10:50 PM   Inj : 1
Inj Volume : 5.000 µl
Acq. Method : E:\DATA\ZQ\ZQ-10-47\ZQ-10-47 2018-01-09 12-09-18\AD-90-10-254NM-30MIN.M
Last changed : 1/9/2018 12:09:18 PM by SYSTEM
Analysis Method : E:\DATA\ZQ\ZQ-10-47\ZQ-10-47 2018-01-09 12-09-18\AD-90-10-254NM-30MIN.M (Sequence Method)
Last changed : 4/11/2018 2:33:06 PM by SYSTEM
(modified after loading)
Additional Info : Peak(s) manually integrated

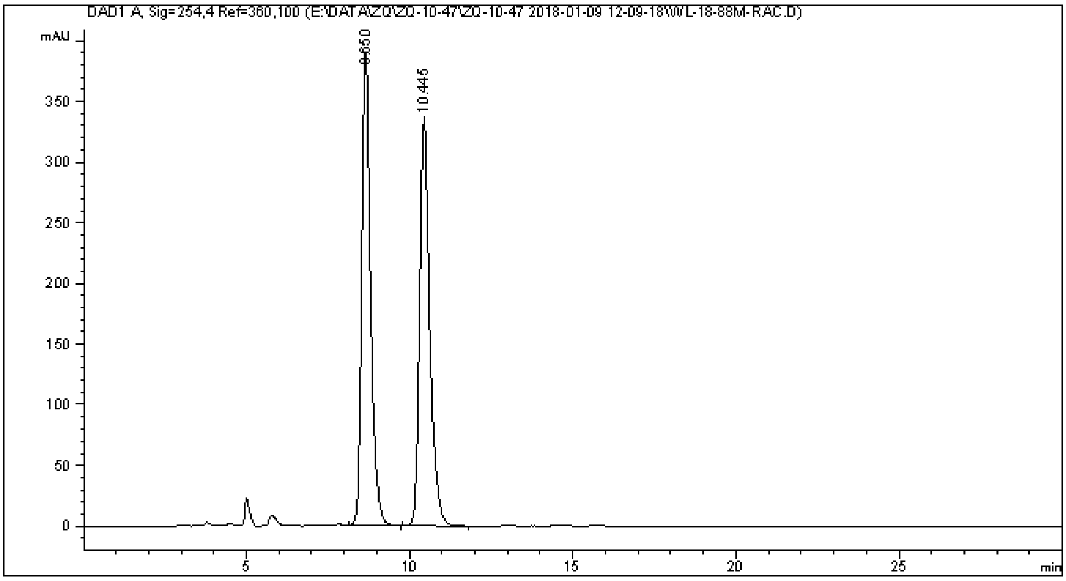

===

Area Percent Report

===

Sorted By : Signal
Multiplier : 1.0000
Dilution : 1.0000
Do not use Multiplier & Dilution Factor with ISTDs

Signal 1: DAD1 A, Sig=254,4 Ref=360,100

Peak #	RetTime [min]	Type	Width [min]	Area [mAU*s]	Height [mAU]	Area %
1	8.650	BB	0.2832	7384.75391	389.43732	50.3112
2	10.445	BB	0.3228	7293.39795	336.64133	49.6888

Totals : 1.46782e4 726.07864

1260 4/11/2018 2:33:09 PM SYSTEM   Page 1 of 2

Supplementary Figure 180. HPLC spectrum of (*rac*)-**3G**

Data File E:\DATA\ZQ\ZQ-10-47\ZQ-10-47 2018-01-09 12-09-18\WL-18-88M-RAC5.D
Sample Name: ZQ-10-47-thenyl1-opt

===
Acq. Operator : SYSTEM Seq. Line : 6
Acq. Instrument : 1260 Location : 76
Injection Date : 1/9/2018 2:58:14 PM Inj : 1
Inj Volume : 5.000 µl
Acq. Method : E:\DATA\ZQ\ZQ-10-47\ZQ-10-47 2018-01-09 12-09-18\AD-90-10-254NM-20MIN.M
Last changed : 1/9/2018 2:23:36 PM by SYSTEM
Analysis Method : E:\DATA\ZQ\ZQ-10-47\ZQ-10-47 2018-01-09 12-09-18\AD-90-10-254NM-20MIN.M (Sequence Method)
Last changed : 4/11/2018 2:34:11 PM by SYSTEM
(modified after loading)
Additional Info : Peak(s) manually integrated

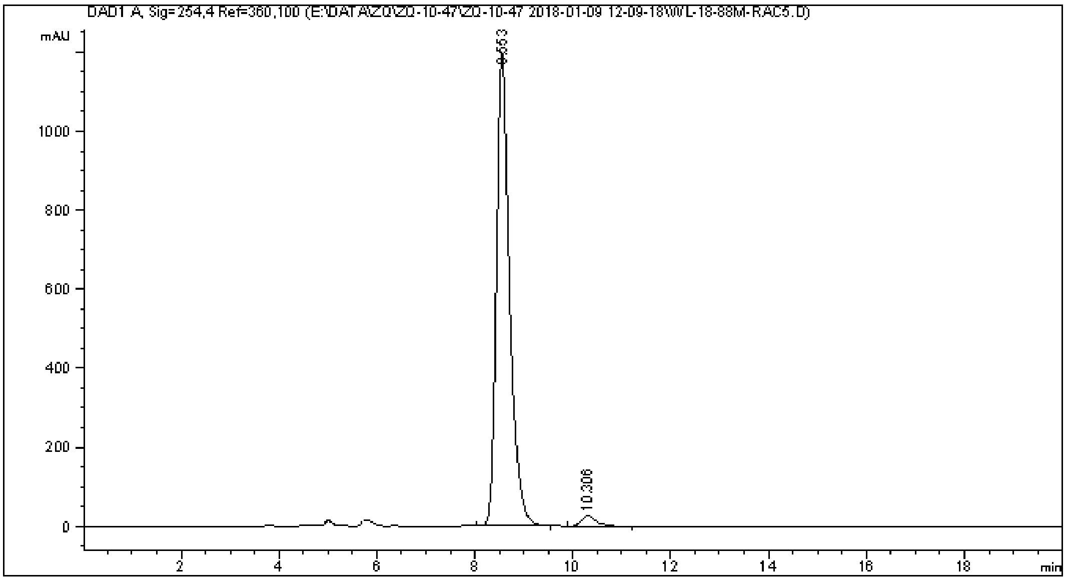

===
Area Percent Report
===

Sorted By : Signal
Multiplier : 1.0000
Dilution : 1.0000
Do not use Multiplier & Dilution Factor with ISTDs

Signal 1: DAD1 A, Sig=254,4 Ref=360,100

Peak #	RetTime [min]	Type	Width [min]	Area [mAU*s]	Height [mAU]	Area %
1	8.553	BB	0.2815	2.25298e4	1197.55994	97.6181
2	10.306	BB	0.3083	549.72864	26.36016	2.3819

Totals : 2.30795e4 1223.92009

Supplementary Figure 181. HPLC spectrum of (*S*)-**3G**

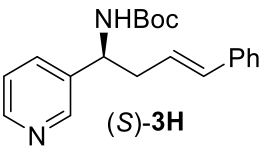

(*S*)-**3H**

Data File E:\DATA\WL\WL-18-88\WL-18-88I-RAC-2 2018-01-05 23-46-31\WL-18-885.D
Sample Name: WL-18-88I-RAC

===

Acq. Operator : SYSTEM Seq. Line : 6
Acq. Instrument : 1260 Location : 92
Injection Date : 1/6/2018 2:16:00 AM Inj : 1
Inj Volume : 5.000 µl
Acq. Method : E:\DATA\WL\WL-18-88\WL-18-88I-RAC-2 2018-01-05 23-46-31\OJH-90-10-1.0ML-ALL-254NM-30MIN.M
Last changed : 1/6/2018 1:10:44 AM by SYSTEM
Analysis Method : E:\DATA\WL\WL-18-88\WL-18-88I-RAC-2 2018-01-05 23-46-31\OJH-90-10-1.0ML-ALL-254NM-30MIN.M (Sequence Method)
Last changed : 5/2/2018 9:22:06 PM by SYSTEM
(modified after loading)
Additional Info : Peak(s) manually integrated

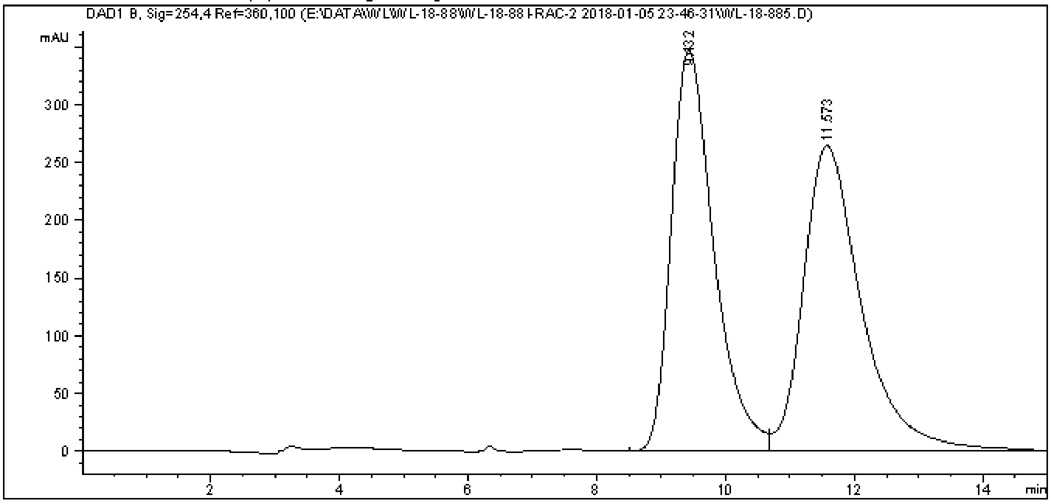

===

Area Percent Report

===

Sorted By : Signal
Multiplier : 1.0000
Dilution : 1.0000
Do not use Multiplier & Dilution Factor with ISTDs

Signal 1: DAD1 B, Sig=254,4 Ref=360,100

Peak #	RetTime [min]	Type	Width [min]	Area [mAU*s]	Height [mAU]	Area %
1	9.432	BV	0.7008	1.60118e4	346.69446	48.9014
2	11.573	VB	0.9279	1.67312e4	264.90259	51.0986

Totals : 3.27430e4 611.59705

===

*** End of Report ***

1260 5/2/2018 9:22:09 PM SYSTEM Page 1 of 1

Supplementary Figure 182. HPLC spectrum of (*rac*)-**3H**

Data File E:\DATA\WL\WL-18-88\WL-18-88I-OPT 2018-01-06 12-45-29\WL-18-88.D
Sample Name: WL-18-88I-opt

===

Acq. Operator : SYSTEM Seq. Line : 1
Acq. Instrument : 1260 Location : 93
Injection Date : 1/6/2018 12:46:53 PM Inj : 1
Inj Volume : 5.000 µl
Acq. Method : E:\DATA\WL\WL-18-88\WL-18-88I-opt 2018-01-06 12-45-29\OJH-90-10-1.0ML-ALL-254NM-30MIN.M
Last changed : 1/6/2018 12:45:29 PM by SYSTEM
Analysis Method : E:\DATA\WL\WL-18-88\WL-18-88I-opt 2018-01-06 12-45-29\OJH-90-10-1.0ML-ALL-254NM-30MIN.M (Sequence Method)
Last changed : 5/2/2018 9:22:35 PM by SYSTEM
(modified after loading)
Additional Info : Peak(s) manually integrated

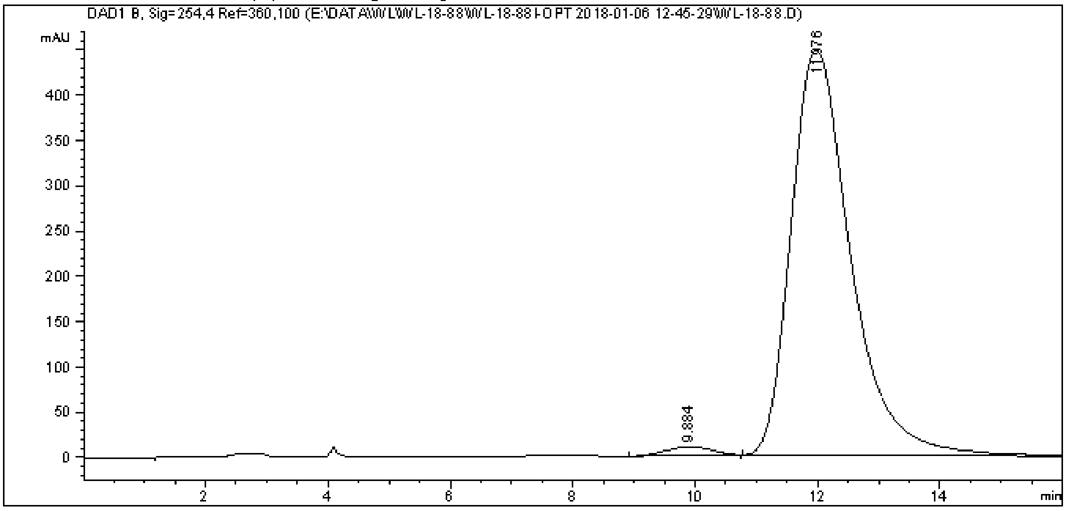

===

Area Percent Report

===

Sorted By : Signal
Multiplier : 1.0000
Dilution : 1.0000
Do not use Multiplier & Dilution Factor with ISTDs

Signal 1: DAD1 B, Sig=254,4 Ref=360,100

Peak #	RetTime [min]	Type	Width [min]	Area [mAU*s]	Height [mAU]	Area %
1	9.884	BB	0.6208	492.61050	9.45538	1.5968
2	11.976	BB	0.9965	3.03569e4	446.31415	98.4032

Totals : 3.08496e4 455.76953

===

*** End of Report ***

1260 5/2/2018 9:22:53 PM SYSTEM Page 1 of 1

Supplementary Figure 183. HPLC spectrum of (*S*)-**3H**

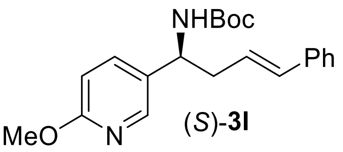

(*S*)-**3l**

Data File E:\DATA\ZQ\ZQ-10-47\ZQ-10-47 2018-01-09 21-05-51\WL-18-88M-RAC.D
Sample Name: ZQ-10-47-6-OMeP

===

Acq. Operator : SYSTEM   Seq. Line : 1
Acq. Instrument : 1260   Location : 72
Injection Date : 1/9/2018 9:07:07 PM   Inj : 1
                                          Inj Volume : 3.000 µl
Acq. Method : E:\DATA\ZQ\ZQ-10-47\ZQ-10-47 2018-01-09 21-05-51\IA-80-20-254NM-30MIN.M
Last changed : 1/9/2018 9:30:42 PM by SYSTEM
(modified after loading)
Analysis Method : E:\DATA\ZQ\ZQ-10-47\ZQ-10-47 2018-01-09 21-05-51\IA-80-20-254NM-30MIN.M (Sequence Method)
Last changed : 4/11/2018 2:35:55 PM by SYSTEM
(modified after loading)
Additional Info : Peak(s) manually integrated

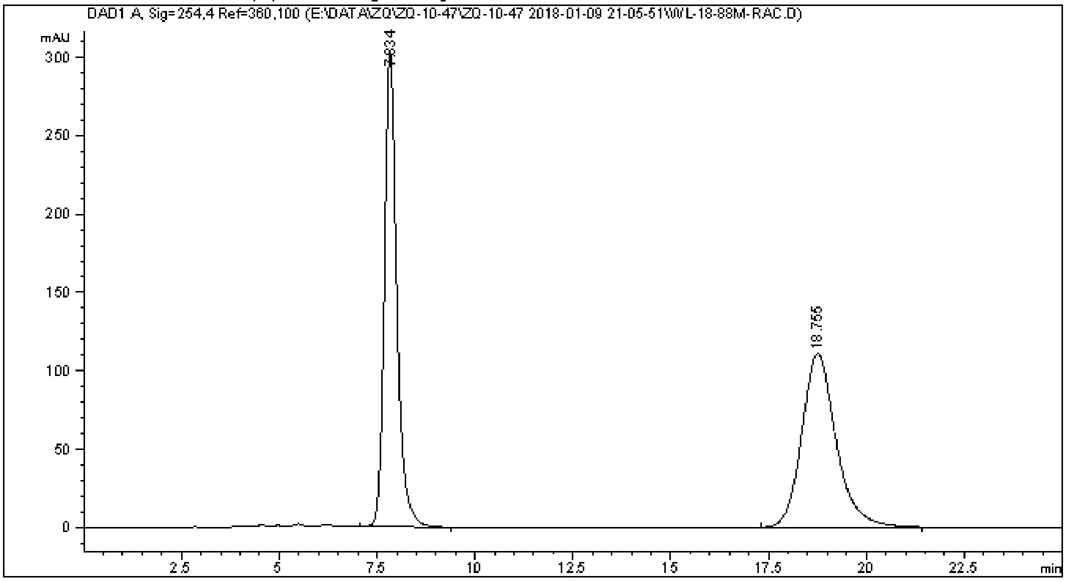

===
Area Percent Report
===

Sorted By : Signal
Multiplier : 1.0000
Dilution : 1.0000
Do not use Multiplier & Dilution Factor with ISTDs

Signal 1: DAD1 A, Sig=254,4 Ref=360,100

Peak #	RetTime [min]	Type	Width [min]	Area [mAU*s]	Height [mAU]	Area %
1	7.834	BB	0.3287	6663.08691	301.65628	49.7001
2	18.755	BB	0.8488	6743.51270	110.84497	50.2999

Totals : 1.34066e4 412.50125

Supplementary Figure 184. HPLC spectrum of (*rac*)-**3l**

Data File E:\DATA\ZQ\ZQ-10-47\ZQ-10-47 2018-01-09 21-33-31\WL-18-88M-RAC.D
Sample Name: ZQ-10-47-6-OMeP-opt

===

Acq. Operator : SYSTEM Seq. Line : 1
Acq. Instrument : 1260 Location : 77
Injection Date : 1/9/2018 9:34:58 PM Inj : 1
Inj Volume : 3.000 µl
Acq. Method : E:\DATA\ZQ\ZQ-10-47\ZQ-10-47 2018-01-09 21-33-31\IA-80-20-254NM-30MIN.M
Last changed : 1/9/2018 9:54:18 PM by SYSTEM
(modified after loading)
Analysis Method : E:\DATA\ZQ\ZQ-10-47\ZQ-10-47 2018-01-09 21-33-31\IA-80-20-254NM-30MIN.M (Sequence Method)
Last changed : 4/11/2018 2:38:53 PM by SYSTEM
(modified after loading)

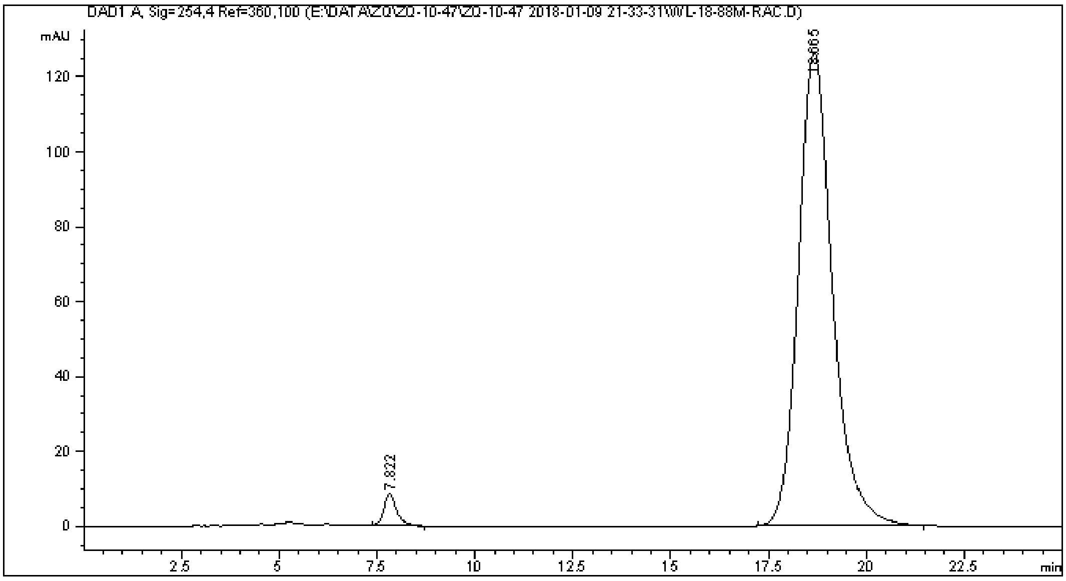

===

Area Percent Report

===

Sorted By : Signal
Multiplier : 1.0000
Dilution : 1.0000
Do not use Multiplier & Dilution Factor with ISTDs

Signal 1: DAD1 A, Sig=254,4 Ref=360,100

Peak #	RetTime [min]	Type	Width [min]	Area [mAU*s]	Height [mAU]	Area %
1	7.822	BB	0.3128	185.08739	8.37467	2.3578
2	18.665	BB	0.8696	7664.75488	126.38108	97.6422

Totals : 7849.84227 134.75575

1260 4/11/2018 2:38:57 PM SYSTEM Page 1 of 2

Supplementary Figure 185. HPLC spectrum of (*S*)-**3I**

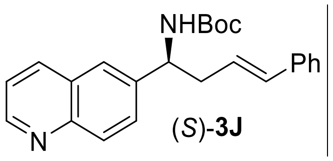

(*S*)-**3J**

Data File E:\DATA\WL\WL-18-88\WL-18-88QR-RAC 2018-01-17 20-37-26\WL-18-881.D
Sample Name: WL-18-88R-RAC

===
Acq. Operator : SYSTEM Seq. Line : 2
Acq. Instrument : 1260 Location : 4
Injection Date : 1/17/2018 8:52:22 PM Inj : 1
Inj Volume : 5.000 µl
Acq. Method : E:\DATA\WL\WL-18-88\WL-18-88QR-RAC 2018-01-17 20-37-26\ODH-75-25-1.0ML-ALL-254NM-30MIN.M
Last changed : 1/17/2018 9:01:09 PM by SYSTEM
(modified after loading)
Analysis Method : E:\DATA\WL\WL-18-88\WL-18-88QR-RAC 2018-01-17 20-37-26\ODH-75-25-1.0ML-ALL-254NM-30MIN.M (Sequence Method)
Last changed : 5/2/2018 9:39:00 PM by SYSTEM
(modified after loading)
Additional Info : Peak(s) manually integrated

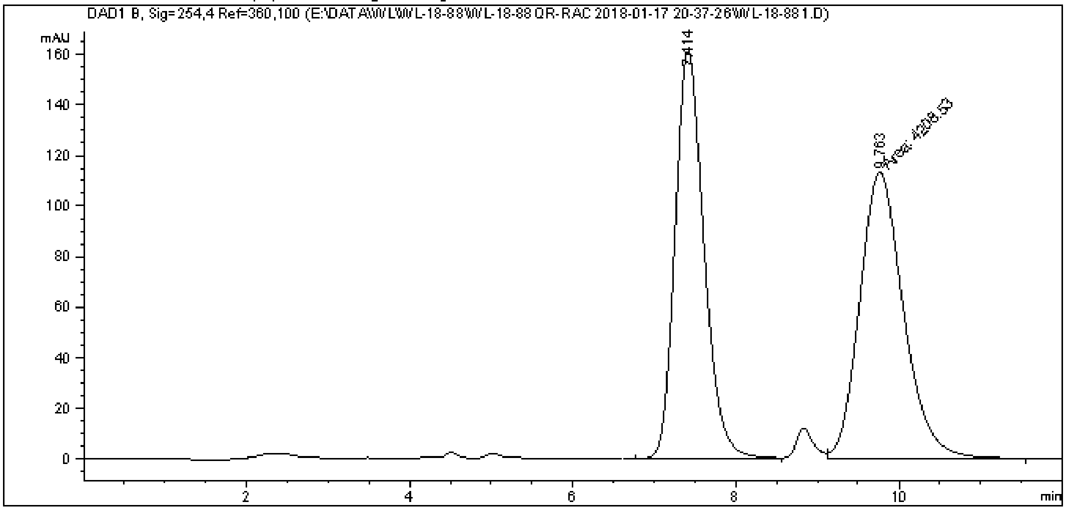

===
Area Percent Report
===

Sorted By : Signal
Multiplier : 1.0000
Dilution : 1.0000
Do not use Multiplier & Dilution Factor with ISTDs

Signal 1: DAD1 B, Sig=254,4 Ref=360,100

Peak #	RetTime [min]	Type	Width [min]	Area [mAU*s]	Height [mAU]	Area %
1	7.414	BB	0.3785	3959.72852	161.27330	48.4770
2	9.763	FM	0.6200	4208.52734	113.13475	51.5230

Totals : 8168.25586 274.40805

===

1260 5/2/2018 9:39:04 PM SYSTEM Page 1 of 2

Supplementary Figure 186. HPLC spectrum of (*rac*)-**3J**

Data File E:\DATA\WL\WL-18-88\WL-18-88QR-RAC 2018-01-17 20-37-26\WL-18-883.D
Sample Name: WL-18-88R-OPT

===

Acq. Operator : SYSTEM Seq. Line : 4
Acq. Instrument : 1260 Location : 5
Injection Date : 1/17/2018 9:25:23 PM Inj : 1
Inj Volume : 5.000 µl
Acq. Method : E:\DATA\WL\WL-18-88\WL-18-88QR-RAC 2018-01-17 20-37-26\ODH-75-25-1.0ML-ALL-254NM-30MIN.M
Last changed : 1/17/2018 9:01:09 PM by SYSTEM
Analysis Method : E:\DATA\WL\WL-18-88\WL-18-88QR-RAC 2018-01-17 20-37-26\ODH-75-25-1.0ML-ALL-254NM-30MIN.M (Sequence Method)
Last changed : 5/2/2018 9:39:00 PM by SYSTEM
(modified after loading)
Additional Info : Peak(s) manually integrated

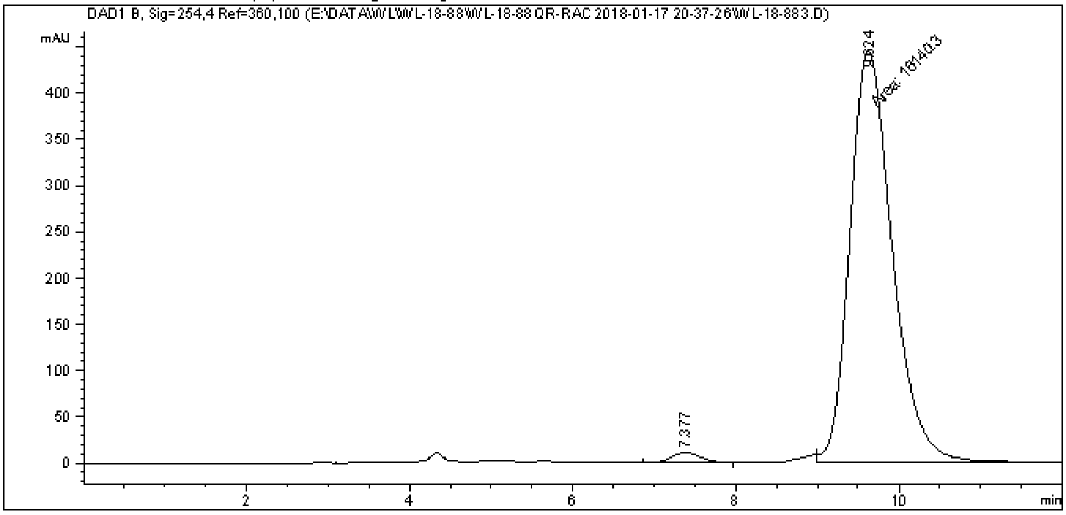

===

Area Percent Report

===

Sorted By : Signal
Multiplier : 1.0000
Dilution : 1.0000
Do not use Multiplier & Dilution Factor with ISTDs

Signal 1: DAD1 B, Sig=254,4 Ref=360,100

Peak #	RetTime [min]	Type	Width [min]	Area [mAU*s]	Height [mAU]	Area %
1	7.377	BB	0.3507	250.45882	10.79424	1.5280
2	9.624	FM	0.6054	1.61403e4	444.36020	98.4720

Totals : 1.63908e4 455.15444

===

*** End of Report ***

1260 5/2/2018 9:39:37 PM SYSTEM Page 1 of 1

Supplementary Figure 187. HPLC spectrum of (*S*)-**3J**

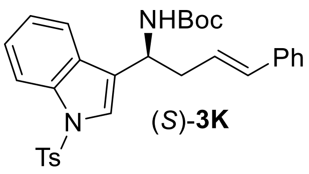

(*S*)-**3K**

Data File E:\DATA\WL\WL-18-88\WL-18-88QR-RAC 2018-01-17 20-37-26\WL-18-88.D
Sample Name: WL-18-88Q-RAC

===
Acq. Operator : SYSTEM Seq. Line : 1
Acq. Instrument : 1260 Location : 2
Injection Date : 1/17/2018 8:38:51 PM Inj : 1
Inj Volume : 5.000 µl
Acq. Method : E:\DATA\WL\WL-18-88\WL-18-88QR-RAC 2018-01-17 20-37-26\ODH-75-25-1.0ML-ALL-254NM-30MIN.M
Last changed : 1/17/2018 8:48:22 PM by SYSTEM
(modified after loading)
Analysis Method : E:\DATA\WL\WL-18-88\WL-18-88QR-RAC 2018-01-17 20-37-26\ODH-75-25-1.0ML-ALL-254NM-30MIN.M (Sequence Method)
Last changed : 5/2/2018 9:38:23 PM by SYSTEM
(modified after loading)
Additional Info : Peak(s) manually integrated

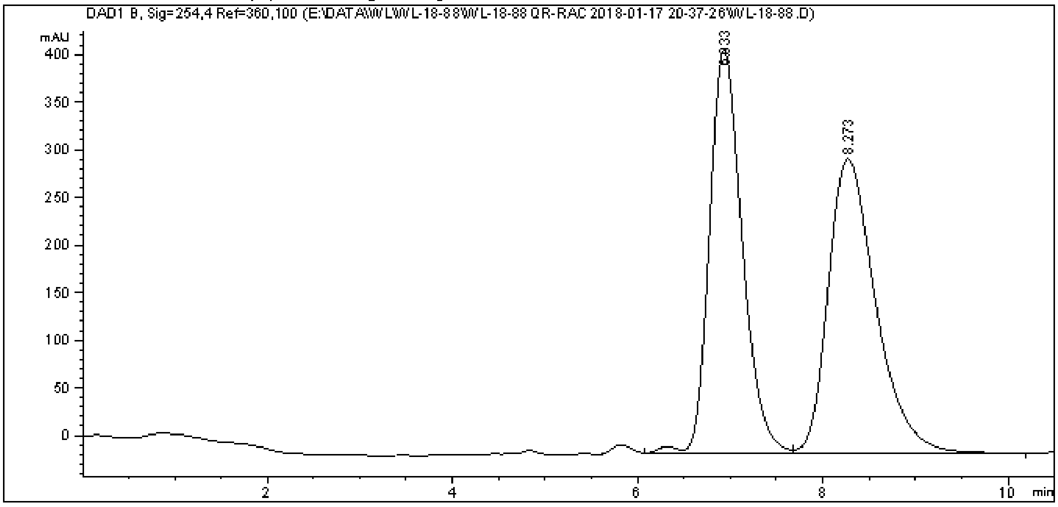

===
Area Percent Report
===

Sorted By : Signal
Multiplier : 1.0000
Dilution : 1.0000
Do not use Multiplier & Dilution Factor with ISTDs

Signal 1: DAD1 B, Sig=254,4 Ref=360,100

Peak #	RetTime [min]	Type	Width [min]	Area [mAU*s]	Height [mAU]	Area %
1	6.933	VV R	0.3765	1.04295e4	422.61874	48.4837
2	8.273	VB	0.5450	1.10819e4	309.30817	51.5163
Totals :				2.15114e4	731.92691	

===

Supplementary Figure 188. HPLC spectrum of (*rac*)-**3K**

Data File E:\DATA\WL\WL-18-88\WL-18-88QR-RAC 2018-01-17 20-37-26\WL-18-882.D
Sample Name: WL-18-88Q-OPT

===

Acq. Operator : SYSTEM Seq. Line : 3
Acq. Instrument : 1260 Location : 3
Injection Date : 1/17/2018 9:08:48 PM Inj : 1
Inj Volume : 5.000 µl
Acq. Method : E:\DATA\WL\WL-18-88\WL-18-88QR-RAC 2018-01-17 20-37-26\ODH-75-25-1.0ML-ALL-254NM-30MIN.M
Last changed : 1/17/2018 9:01:09 PM by SYSTEM
Analysis Method : E:\DATA\WL\WL-18-88\WL-18-88QR-RAC 2018-01-17 20-37-26\ODH-75-25-1.0ML-ALL-254NM-30MIN.M (Sequence Method)
Last changed : 5/2/2018 9:38:23 PM by SYSTEM
(modified after loading)
Additional Info : Peak(s) manually integrated

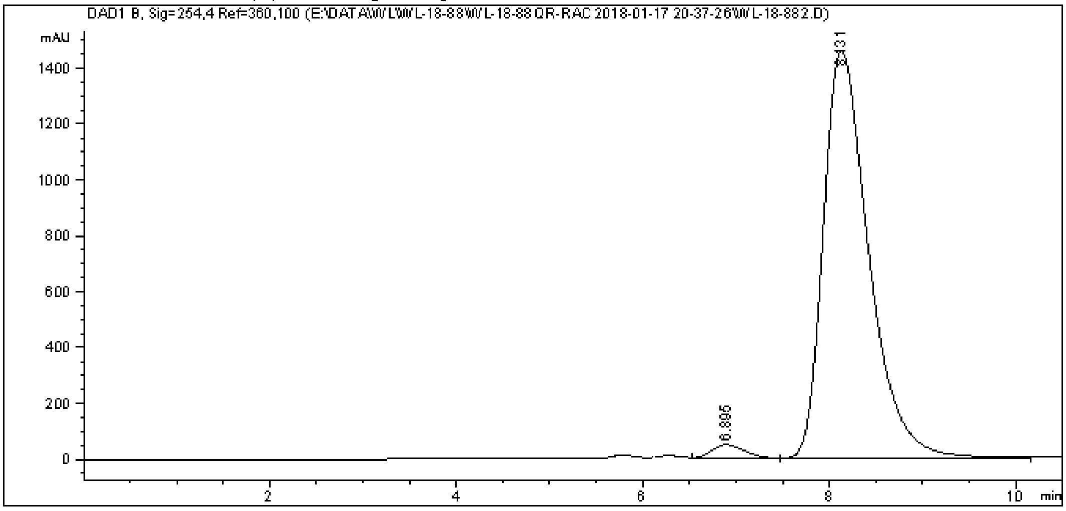

===

Area Percent Report

===

Sorted By : Signal
Multiplier : 1.0000
Dilution : 1.0000
Do not use Multiplier & Dilution Factor with ISTDs

Signal 1: DAD1 B, Sig=254,4 Ref=360,100

Peak #	RetTime [min]	Type	Width [min]	Area [mAU*s]	Height [mAU]	Area %
1	6.895	VB	0.3668	1185.02563	48.88416	2.3510
2	8.131	BB	0.5158	4.92196e4	1458.23877	97.6490

Totals : 5.04046e4 1507.12293

===

*** End of Report ***

1260 5/2/2018 9:38:41 PM SYSTEM Page 1 of 1

Supplementary Figure 189. HPLC spectrum of (*S*)-**3K**

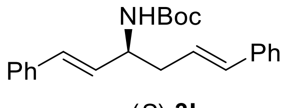

(*S*)-**3L**

Data File E:\DATA\WL\WL-18-88\WL-18-88KLM-RAC 2018-01-06 23-01-18\WL-18-884.D
Sample Name: WL-18-88L-OPT

===

Acq. Operator : SYSTEM Seq. Line : 5
Acq. Instrument : 1260 Location : 44
Injection Date : 1/7/2018 12:34:23 AM Inj : 1
Inj Volume : 5.000 µl
Acq. Method : E:\DATA\WL\WL-18-88\WL-18-88KLM-RAC 2018-01-06 23-01-18\ODH-90-10-1.0ML-ALL-254NM-30MIN.M
Last changed : 1/6/2018 11:48:26 PM by SYSTEM
Analysis Method : E:\DATA\WL\WL-18-88\WL-18-88KLM-RAC 2018-01-06 23-01-18\ODH-90-10-1.0ML-ALL-254NM-30MIN.M (Sequence Method)
Last changed : 5/2/2018 9:26:00 PM by SYSTEM
(modified after loading)
Additional Info : Peak(s) manually integrated

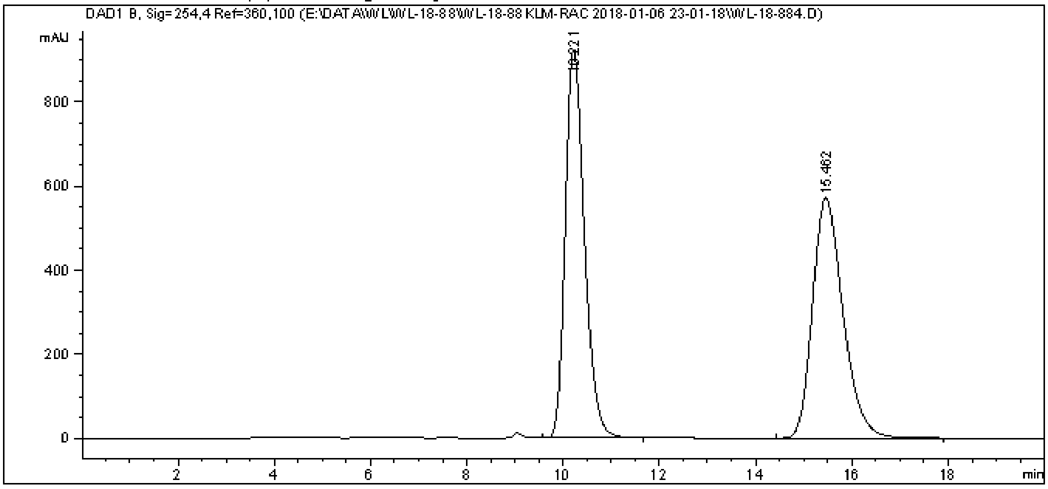

===

Area Percent Report

===

Sorted By : Signal
Multiplier : 1.0000
Dilution : 1.0000
Do not use Multiplier & Dilution Factor with ISTDs

Signal 1: DAD1 B, Sig=254,4 Ref=360,100

Peak #	RetTime [min]	Type	Width [min]	Area [mAU*s]	Height [mAU]	Area %
1	10.221	BB	0.4225	2.54936e4	922.33771	50.3099
2	15.462	BB	0.6786	2.51796e4	570.84875	49.6901

Totals : 5.06731e4 1493.18646

===

*** End of Report ***

1260 5/2/2018 9:26:02 PM SYSTEM Page 1 of 1

Supplementary Figure 190. HPLC spectrum of (*rac*)-**3L**

Data File E:\DATA\WL\WL-18-88\WL-18-88KLM-RAC 2018-01-06 23-01-18\WL-18-883.D
Sample Name: WL-18-88K-OPT

```
=====================================================================
Acq. Operator   : SYSTEM                          Seq. Line :    4
Acq. Instrument : 1260                             Location :   42
Injection Date  : 1/7/2018 12:12:59 AM                   Inj :    1
                                                Inj Volume : 5.000 µl
Acq. Method     : E:\DATA\WL\WL-18-88\WL-18-88KLM-RAC 2018-01-06 23-01-18\ODH-90-10-1.0ML-ALL
                  -254NM-30MIN.M
Last changed    : 1/6/2018 11:48:26 PM by SYSTEM
Analysis Method : E:\DATA\WL\WL-18-88\WL-18-88KLM-RAC 2018-01-06 23-01-18\ODH-90-10-1.0ML-ALL
                  -254NM-30MIN.M (Sequence Method)
Last changed    : 5/2/2018 9:26:00 PM by SYSTEM
                  (modified after loading)
Additional Info : Peak(s) manually integrated
```

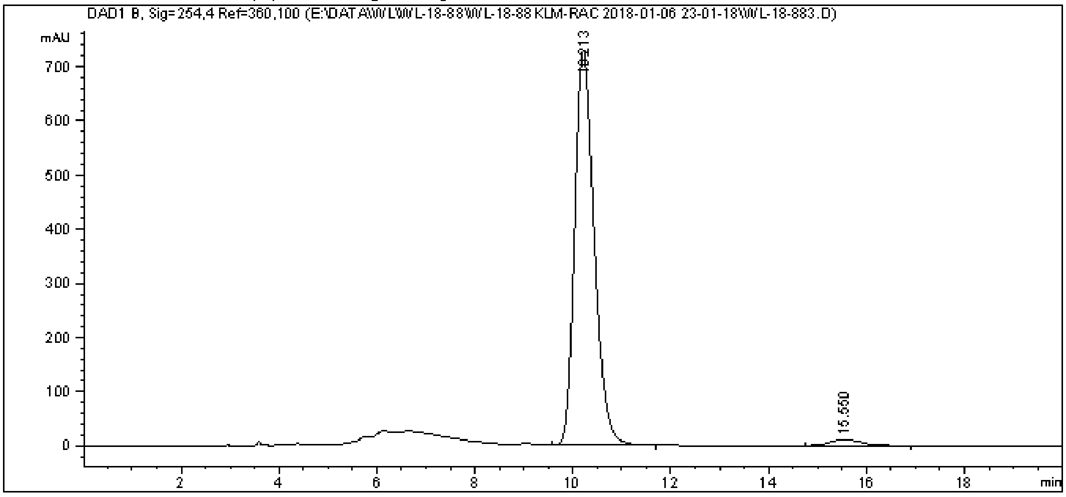


```
=====================================================================
                         Area Percent Report
=====================================================================

Sorted By             :      Signal
Multiplier            :      1.0000
Dilution              :      1.0000
Do not use Multiplier & Dilution Factor with ISTDs


Signal 1: DAD1 B, Sig=254,4 Ref=360,100
```

Peak #	RetTime [min]	Type	Width [min]	Area [mAU*s]	Height [mAU]	Area %
1	10.213	BB	0.4231	2.01898e4	728.95013	97.5671
2	15.550	BB	0.5313	503.45343	11.18180	2.4329

Totals : 2.06932e4 740.13194

```
=====================================================================
                          *** End of Report ***
```

1260 5/2/2018 9:26:20 PM SYSTEM Page 1 of 1

Supplementary Figure 191. HPLC spectrum of (*S*)-**3L**

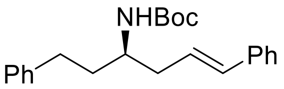

(*R*)-**3M**

Data File E:\DATA\WL\WL-18-88\WL-18-88NO-RAC 2018-01-11 20-10-01\WL-18-88.D
Sample Name: WL-18-88N-RAC

===

Acq. Operator : SYSTEM   Seq. Line : 1
Acq. Instrument : 1260   Location : 6
Injection Date : 1/11/2018 8:11:33 PM   Inj : 1
Inj Volume : 5.000 µl
Acq. Method : E:\DATA\WL\WL-18-88\WL-18-88NO-RAC 2018-01-11 20-10-01\ODH-90-10-1.0ML-ALL-254NM-30MIN.M
Last changed : 1/11/2018 8:23:24 PM by SYSTEM
(modified after loading)
Analysis Method : E:\DATA\WL\WL-18-88\WL-18-88NO-RAC 2018-01-11 20-10-01\ODH-90-10-1.0ML-ALL-254NM-30MIN.M (Sequence Method)
Last changed : 5/2/2018 9:29:47 PM by SYSTEM
(modified after loading)
Additional Info : Peak(s) manually integrated

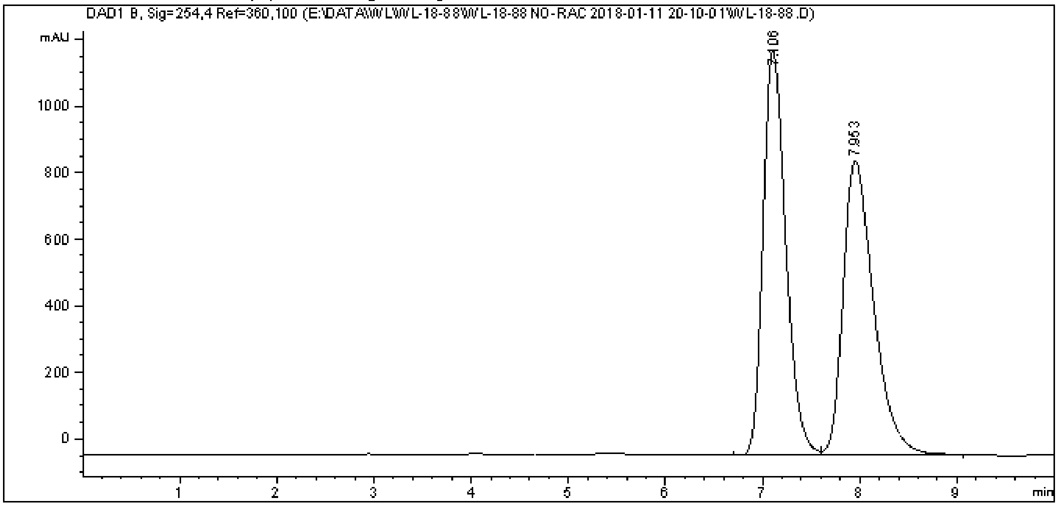

===

Area Percent Report

===

Sorted By : Signal
Multiplier : 1.0000
Dilution : 1.0000
Do not use Multiplier & Dilution Factor with ISTDs

Signal 1: DAD1 B, Sig=254,4 Ref=360,100

Peak #	RetTime [min]	Type	Width [min]	Area [mAU*s]	Height [mAU]	Area %
1	7.106	BV	0.2494	1.97548e4	1216.67871	50.4966
2	7.953	VB	0.3347	1.93663e4	883.64673	49.5034

Totals : 3.91211e4 2100.32544

===

1260 5/2/2018 9:29:50 PM SYSTEM   Page 1 of 2

Supplementary Figure 192. HPLC spectrum of (*rac*)-**3M**

Data File E:\DATA\WL\WL-18-88\WL-18-88NO-OPT 2018-01-12 11-56-55\WL-18-88.D
Sample Name: WL-18-88N-OPT

===

Acq. Operator : SYSTEM Seq. Line : 1
Acq. Instrument : 1260 Location : 11
Injection Date : 1/12/2018 11:58:17 AM Inj : 1
Inj Volume : 5.000 µl
Acq. Method : E:\DATA\WL\WL-18-88\WL-18-88NO-OPT 2018-01-12 11-56-55\ODH-90-10-1.0ML-ALL-254NM-30MIN.M
Last changed : 1/12/2018 11:57:17 AM by SYSTEM
(modified after loading)
Analysis Method : E:\DATA\WL\WL-18-88\WL-18-88NO-OPT 2018-01-12 11-56-55\ODH-90-10-1.0ML-ALL-254NM-30MIN.M (Sequence Method)
Last changed : 5/2/2018 9:30:37 PM by SYSTEM
(modified after loading)
Additional Info : Peak(s) manually integrated

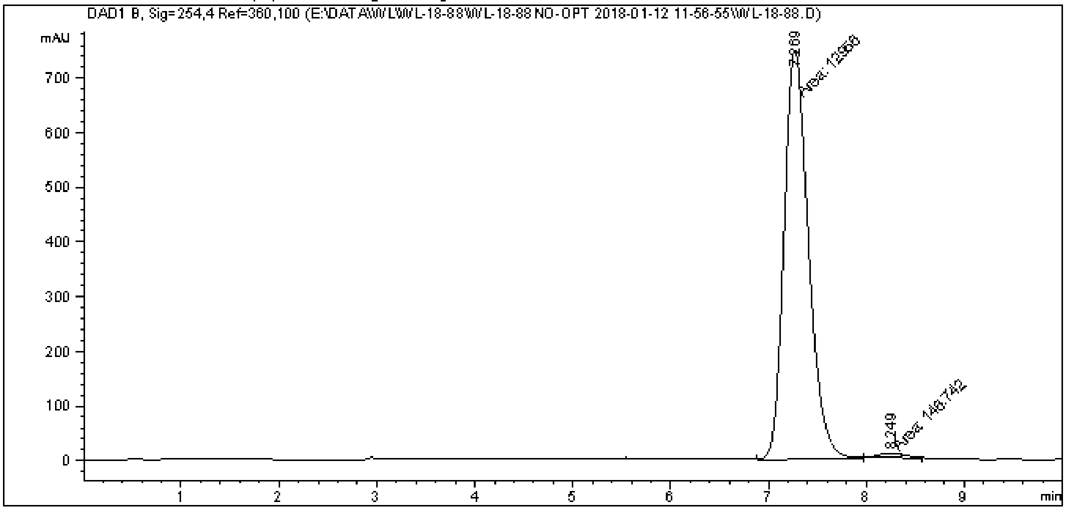

===

Area Percent Report

===

Sorted By : Signal
Multiplier : 1.0000
Dilution : 1.0000
Do not use Multiplier & Dilution Factor with ISTDs

Signal 1: DAD1 B, Sig=254,4 Ref=360,100

Peak #	RetTime [min]	Type	Width [min]	Area [mAU*s]	Height [mAU]	Area %
1	7.269	MM	0.2890	1.29560e4	747.25684	98.8801
2	8.249	MM	0.3357	146.74161	7.28488	1.1199

Totals : 1.31027e4 754.54171

===

1260 5/2/2018 9:31:37 PM SYSTEM Page 1 of 2

Supplementary Figure 193. HPLC spectrum of (*S*)-**3M**

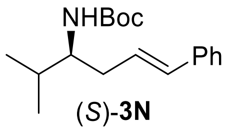

(*S*)-**3N**

Data File E:\DATA\ZQ\ZQ-10-59\ZQ-10-59 2018-01-18 14-58-07\SLM-8-372.D
Sample Name: ZQ-10-60-iBu-rac

===
Acq. Operator : SYSTEM Seq. Line : 3
Acq. Instrument : 1260 Location : 75
Injection Date : 1/18/2018 3:48:31 PM Inj : 1
Inj Volume : 5.000 µl
Acq. Method : E:\DATA\ZQ\ZQ-10-59\ZQ-10-59 2018-01-18 14-58-07\OD-90-10-254NM-30MIN.M
Last changed : 1/18/2018 4:09:08 PM by SYSTEM
(modified after loading)
Analysis Method : E:\DATA\ZQ\ZQ-10-59\ZQ-10-59 2018-01-18 14-58-07\OD-90-10-254NM-30MIN.M (
Sequence Method)
Last changed : 4/11/2018 2:59:48 PM by SYSTEM
(modified after loading)
Additional Info : Peak(s) manually integrated

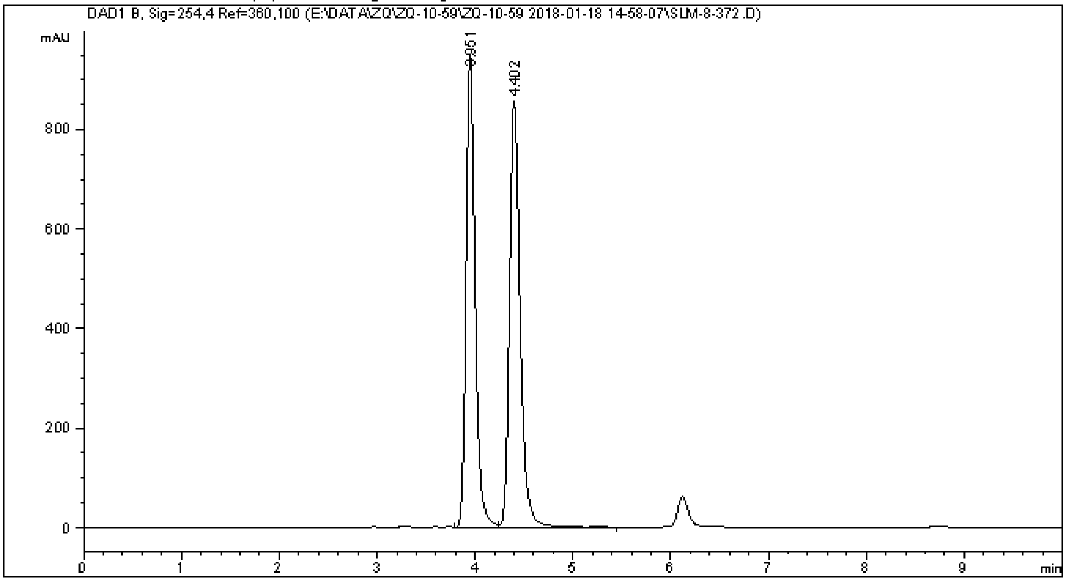

===
Area Percent Report
===

Sorted By : Signal
Multiplier : 1.0000
Dilution : 1.0000
Do not use Multiplier & Dilution Factor with ISTDs

Signal 1: DAD1 B, Sig=254,4 Ref=360,100

Peak #	RetTime [min]	Type	Width [min]	Area [mAU*s]	Height [mAU]	Area %
1	3.951	BV	0.0978	6096.83936	951.31592	48.5299
2	4.402	VV R	0.1139	6466.22998	856.44165	51.4701

Totals : 1.25631e4 1807.75757

Supplementary Figure 194. HPLC spectrum of (*rac*)-**3N**

Data File E:\DATA\ZQ\ZQ-10-59\ZQ-10-59 2018-01-19 17-10-26\CC-3-92-2-OPT1.D
Sample Name: ZQ-10-60-opt

```
=====================================================================
Acq. Operator   : SYSTEM                        Seq. Line :   2
Acq. Instrument : 1260                           Location :   80
Injection Date  : 1/19/2018 5:23:30 PM                Inj :   1
                                               Inj Volume : 5.000 µl
Acq. Method     : E:\DATA\ZQ\ZQ-10-59\ZQ-10-59 2018-01-19 17-10-26\OD-90-10-254NM-10MIN.M
Last changed    : 1/19/2018 5:10:26 PM by SYSTEM
Analysis Method : E:\DATA\ZQ\ZQ-10-59\ZQ-10-59 2018-01-19 17-10-26\OD-90-10-254NM-10MIN.M (
                  Sequence Method)
Last changed    : 4/11/2018 3:01:31 PM by SYSTEM
                  (modified after loading)
Additional Info : Peak(s) manually integrated
```

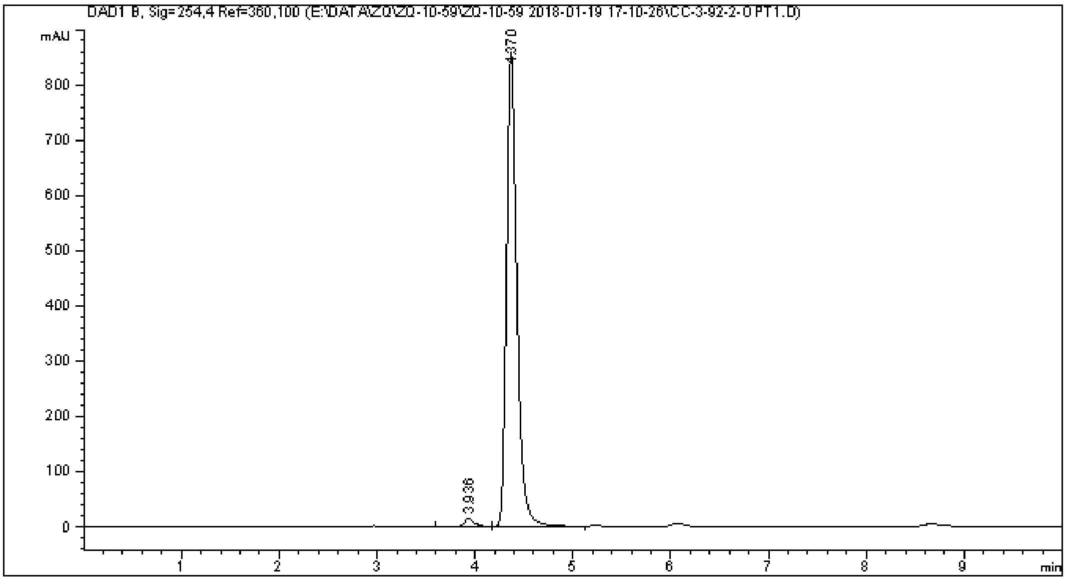


```
=====================================================================
                         Area Percent Report
=====================================================================

Sorted By             :      Signal
Multiplier            :      1.0000
Dilution              :      1.0000
Do not use Multiplier & Dilution Factor with ISTDs


Signal 1: DAD1 B, Sig=254,4 Ref=360,100

Peak RetTime Type  Width     Area      Height     Area
  #   [min]        [min]   [mAU*s]     [mAU]        %
----|-------|----|-------|----------|----------|--------|
   1   3.936 VV R  0.1071   99.49068   13.80729   1.5495
   2   4.370 VB    0.1120 6321.31885  857.83911  98.4505

Totals :                  6420.80953  871.64640
```


Supplementary Figure 195. HPLC spectrum of (*S*)-**3N**

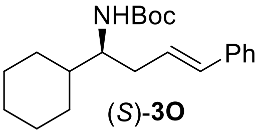

(*S*)-**3O**

```
Data File E:\DATA\WL\WL-18-88\WL-18-88NO-RAC 2018-01-11 20-10-01\WL-18-881.D
Sample Name: WL-18-880-RAC

=====================================================================
Acq. Operator   : SYSTEM                        Seq. Line :   2
Acq. Instrument : 1260                           Location :   7
Injection Date  : 1/11/2018 8:24:58 PM                Inj :   1
                                               Inj Volume : 5.000 µl
Acq. Method     : E:\DATA\WL\WL-18-88\WL-18-88NO-RAC 2018-01-11 20-10-01\ODH-90-10-1.0ML-ALL-
                  254NM-30MIN.M
Last changed    : 1/11/2018 8:26:38 PM by SYSTEM
                  (modified after loading)
Analysis Method : E:\DATA\WL\WL-18-88\WL-18-88NO-RAC 2018-01-11 20-10-01\ODH-90-10-1.0ML-ALL-
                  254NM-30MIN.M (Sequence Method)
Last changed    : 5/2/2018 9:29:47 PM by SYSTEM
                  (modified after loading)
Additional Info : Peak(s) manually integrated
```

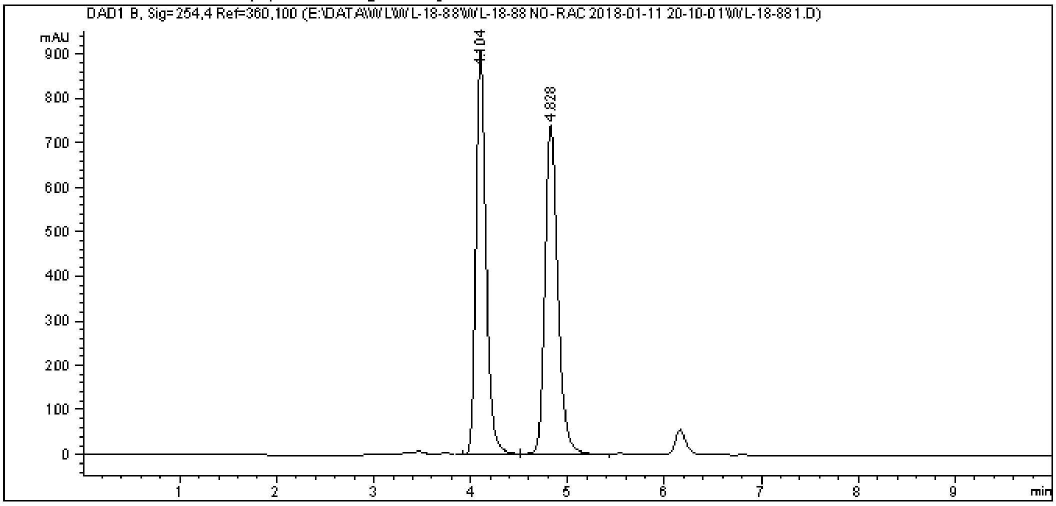


```
=====================================================================
                         Area Percent Report
=====================================================================

Sorted By             :      Signal
Multiplier            :      1.0000
Dilution              :      1.0000
Do not use Multiplier & Dilution Factor with ISTDs


Signal 1: DAD1 B, Sig=254,4 Ref=360,100

Peak RetTime Type  Width     Area      Height     Area
  #   [min]        [min]   [mAU*s]     [mAU]       %
----|-------|----|-------|----------|----------|--------|
   1   4.104 BV    0.1122 6630.14355  907.94366  48.9407
   2   4.828 VV    0.1432 6917.14941  739.19159  51.0593

Totals :                  1.35473e4  1647.13525


=====================================================================
```


Supplementary Figure 196. HPLC spectrum of (*rac*)-**3O**

Data File E:\DATA\WL\WL-18-88\WL-18-88NO-OPT 2018-01-12 11-56-55\WL-18-881.D
Sample Name: WL-18-880-OPT

===

Acq. Operator : SYSTEM Seq. Line : 2
Acq. Instrument : 1260 Location : 12
Injection Date : 1/12/2018 12:09:36 PM Inj : 1
Inj Volume : 5.000 µl
Acq. Method : E:\DATA\WL\WL-18-88\WL-18-88NO-OPT 2018-01-12 11-56-55\ODH-90-10-1.0ML-ALL-254NM-30MIN.M
Last changed : 1/12/2018 11:57:17 AM by SYSTEM
Analysis Method : E:\DATA\WL\WL-18-88\WL-18-88NO-OPT 2018-01-12 11-56-55\ODH-90-10-1.0ML-ALL-254NM-30MIN.M (Sequence Method)
Last changed : 5/2/2018 9:32:02 PM by SYSTEM
Additional Info : Peak(s) manually integrated

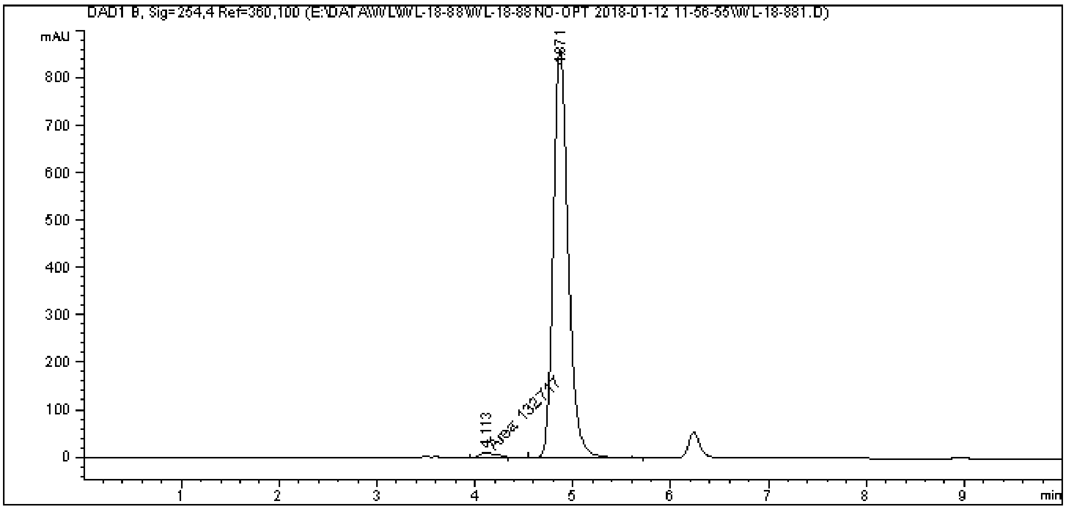

===

Area Percent Report

===

Sorted By : Signal
Multiplier : 1.0000
Dilution : 1.0000
Do not use Multiplier & Dilution Factor with ISTDs

Signal 1: DAD1 B, Sig=254,4 Ref=360,100

Peak #	RetTime [min]	Type	Width [min]	Area [mAU*s]	Height [mAU]	Area %
1	4.113	MM	0.1958	132.71750	11.29862	1.4327
2	4.871	BV R	0.1628	9130.42480	859.33972	98.5673

Totals : 9263.14230 870.63834

===

*** End of Report ***

1260 5/2/2018 9:32:34 PM SYSTEM Page 1 of 1

Supplementary Figure 197. HPLC spectrum of (*S*)-**3O**

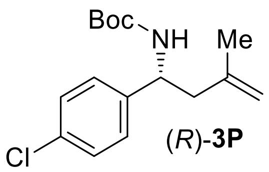

(*R*)-**3P**

```
Data File E:\DATA\WL\WL-19-12\WL-19-12-RAC-AS 2018-09-12 19-19-03\WL-19-12.D
Sample Name: WL-19-12-RAC

    =====================================================================
    Acq. Operator   : SYSTEM                         Seq. Line :   1
    Acq. Instrument : 1260                            Location :   5
    Injection Date  : 9/12/2018 7:20:30 PM                 Inj :   1
                                                    Inj Volume : 5.000 µl
    Acq. Method     : E:\DATA\WL\WL-19-12\WL-19-12-RAC-AS 2018-09-12 19-19-03\ASH-97-3-1.0ML-ALL-
                      254NM-30MIN.M
    Last changed    : 9/12/2018 7:37:33 PM by SYSTEM
                      (modified after loading)
    Analysis Method : E:\DATA\WL\WL-19-12\WL-19-12-RAC-AS 2018-09-12 19-19-03\ASH-97-3-1.0ML-ALL-
                      254NM-30MIN.M (Sequence Method)
    Last changed    : 11/7/2018 3:58:20 PM by SYSTEM
                      (modified after loading)
    Additional Info : Peak(s) manually integrated
```

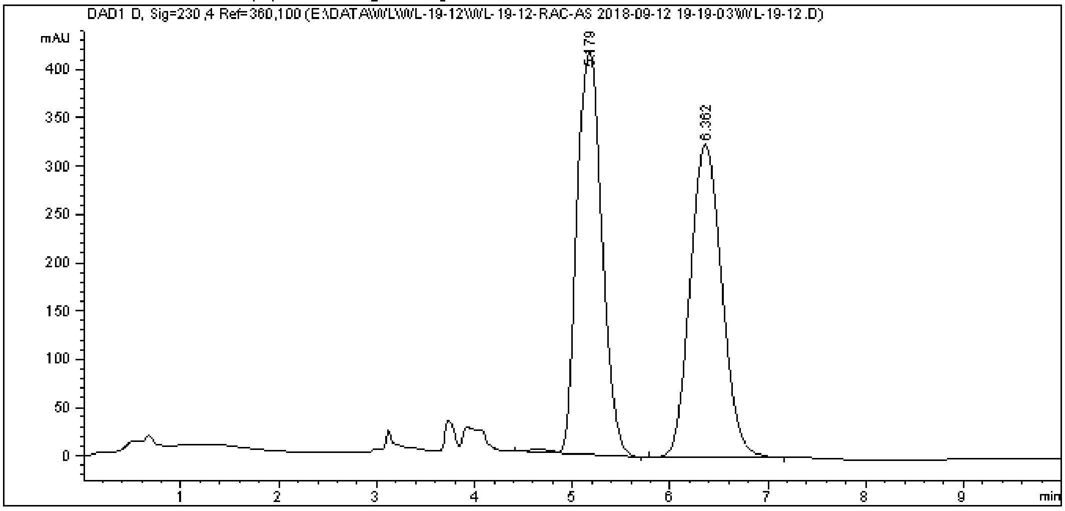


```
    =====================================================================
                             Area Percent Report
    =====================================================================

    Sorted By             :      Signal
    Multiplier            :      1.0000
    Dilution              :      1.0000
    Do not use Multiplier & Dilution Factor with ISTDs


    Signal 1: DAD1 D, Sig=230,4 Ref=360,100

    Peak RetTime Type  Width     Area      Height     Area
      #   [min]        [min]   [mAU*s]     [mAU]        %
    ----|-------|----|-------|----------|----------|--------|
       1   5.179 VB R  0.2844 7473.89600  417.75284  50.6149
       2   6.362 BB    0.3569 7292.29883  323.57196  49.3851

    Totals :                  1.47662e4   741.32480


    =====================================================================
```


Supplementary Figure 198. HPLC spectrum of (*rac*)-**3P**

Data File E:\DATA\WL\WL-19-34\WL-19-34-RAC 2018-09-21 12-41-26\WL-19-343.D
Sample Name: WL-19-26-OPT

===

Acq. Operator : SYSTEM Seq. Line : 4
Acq. Instrument : 1260 Location : 73
Injection Date : 9/21/2018 1:40:40 PM Inj : 1
Inj Volume : 5.000 µl
Acq. Method : E:\DATA\WL\WL-19-34\WL-19-34-rac 2018-09-21 12-41-26\ASH-97-3-1.0ML-ALL-254NM-30MIN.M
Last changed : 9/21/2018 1:36:57 PM by SYSTEM
Analysis Method : E:\DATA\WL\WL-19-34\WL-19-34-rac 2018-09-21 12-41-26\ASH-97-3-1.0ML-ALL-254NM-30MIN.M (Sequence Method)
Last changed : 11/7/2018 4:15:14 PM by SYSTEM
(modified after loading)
Additional Info : Peak(s) manually integrated

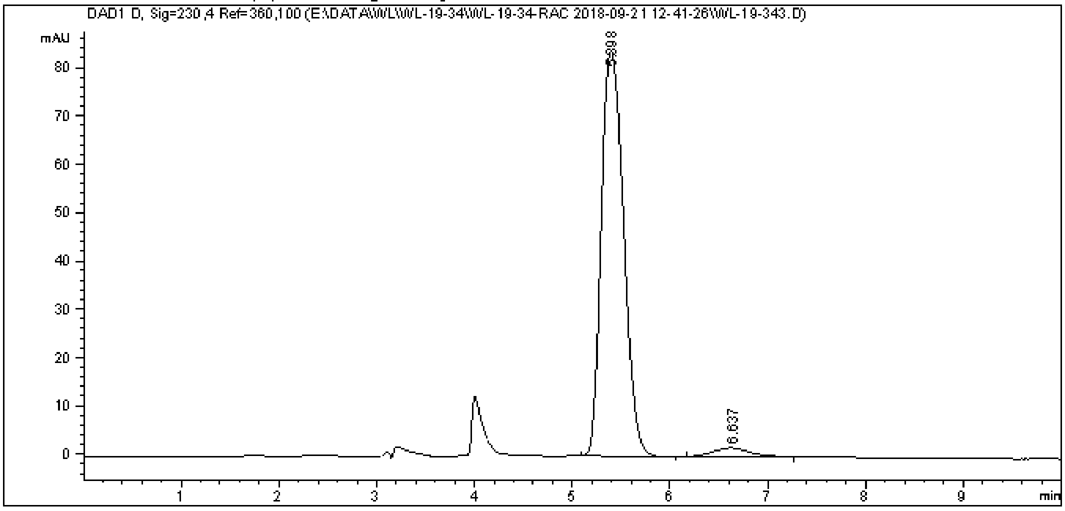

===

Area Percent Report

===

Sorted By : Signal
Multiplier : 1.0000
Dilution : 1.0000
Do not use Multiplier & Dilution Factor with ISTDs

Signal 1: DAD1 D, Sig=230,4 Ref=360,100

Peak #	RetTime [min]	Type	Width [min]	Area [mAU*s]	Height [mAU]	Area %
1	5.398	BB	0.2545	1331.41675	83.73792	96.4926
2	6.637	BB	0.3207	48.39525	1.79404	3.5074

Totals : 1379.81200 85.53196

===

*** End of Report ***

1260 11/7/2018 4:15:18 PM SYSTEM Page 1 of 1

Supplementary Figure 199. HPLC spectrum of (*R*)-**3P**

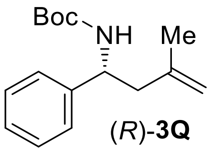

(*R*)-**3Q**

Data File E:\DATA\WL\WL-19-31\WL-19-31-OPT 2018-09-26 13-17-53\WL-19-26.D
Sample Name: WL-19-31-RAC

===

Acq. Operator : SYSTEM Seq. Line : 1
Acq. Instrument : 1260 Location : 51
Injection Date : 9/26/2018 1:19:20 PM Inj : 1
Inj Volume : 5.000 µl
Acq. Method : E:\DATA\WL\WL-19-31\WL-19-31-OPT 2018-09-26 13-17-53\ASH-97-3-1.0ML-ALL-254NM-30MIN.M
Last changed : 9/26/2018 1:18:33 PM by SYSTEM
(modified after loading)
Analysis Method : E:\DATA\WL\WL-19-31\WL-19-31-OPT 2018-09-26 13-17-53\ASH-97-3-1.0ML-ALL-254NM-30MIN.M (Sequence Method)
Last changed : 11/7/2018 4:11:40 PM by SYSTEM
(modified after loading)
Additional Info : Peak(s) manually integrated

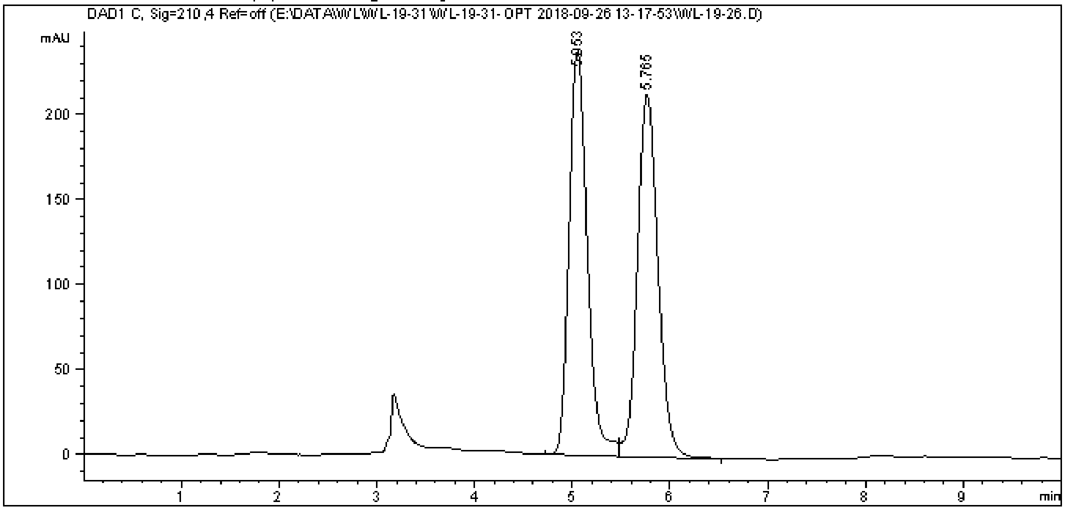

===

Area Percent Report

===

Sorted By : Signal
Multiplier : 1.0000
Dilution : 1.0000
Do not use Multiplier & Dilution Factor with ISTDs

Signal 1: DAD1 C, Sig=210,4 Ref=off

Peak #	RetTime [min]	Type	Width [min]	Area [mAU*s]	Height [mAU]	Area %
1	5.053	BV	0.2006	3088.18921	237.67070	49.8501
2	5.765	VB	0.2270	3106.76514	213.18971	50.1499

Totals : 6194.95435 450.86041

===

1260 11/7/2018 4:11:45 PM SYSTEM Page 1 of 2

Supplementary Figure 200. HPLC spectrum of (*rac*)-**3Q**

```
Data File E:\DATA\WL\WL-19-31\WL-19-31-OPT 2018-09-26 13-17-53\WL-19-261.D
Sample Name: WL-19-31-OPT

    =====================================================================
    Acq. Operator   : SYSTEM                        Seq. Line :   2
    Acq. Instrument : 1260                           Location :   52
    Injection Date  : 9/26/2018 1:35:46 PM                Inj :   1
                                                   Inj Volume : 5.000 µl
    Acq. Method     : E:\DATA\WL\WL-19-31\WL-19-31-OPT 2018-09-26 13-17-53\ASH-97-3-1.0ML-ALL-
                      254NM-30MIN.M
    Last changed    : 9/26/2018 1:18:33 PM by SYSTEM
    Analysis Method : E:\DATA\WL\WL-19-31\WL-19-31-OPT 2018-09-26 13-17-53\ASH-97-3-1.0ML-ALL-
                      254NM-30MIN.M (Sequence Method)
    Last changed    : 11/7/2018 4:11:40 PM by SYSTEM
                      (modified after loading)
    Additional Info : Peak(s) manually integrated
```

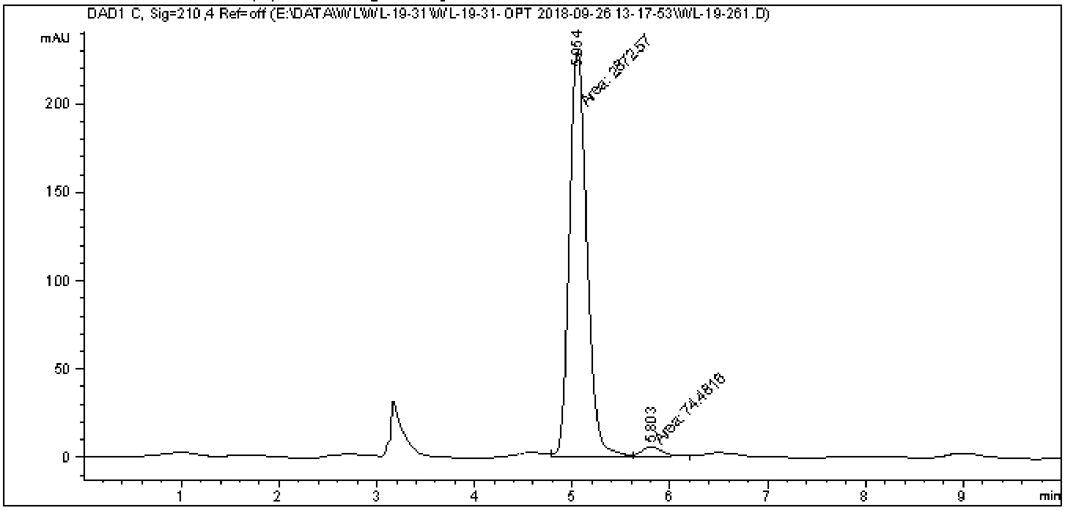


```
    =====================================================================
                             Area Percent Report
    =====================================================================

    Sorted By             :      Signal
    Multiplier            :      1.0000
    Dilution              :      1.0000
    Do not use Multiplier & Dilution Factor with ISTDs


    Signal 1: DAD1 C, Sig=210,4 Ref=off

    Peak RetTime Type  Width     Area      Height     Area
      #   [min]        [min]   [mAU*s]     [mAU]        %
    ----|-------|----|-------|----------|----------|--------|
       1   5.054 MF    0.2084 2872.57300  229.72197  97.4727
       2   5.803 FM    0.2397   74.48161    5.17850   2.5273

    Totals :                  2947.05460  234.90047


    =====================================================================
                             *** End of Report ***
```


Supplementary Figure 201. HPLC spectrum of (*R*)-**3Q**

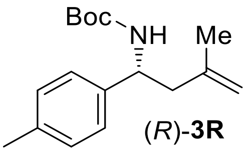

(*R*)-**3R**

```
Data File E:\DATA\WL\WL-19-35\WL-19-35-RAC 2018-09-21 12-02-35\WL-19-35.D
Sample Name: WL-19-35-RAC

    ===================================================================
    Acq. Operator   : SYSTEM                        Seq. Line :    1
    Acq. Instrument : 1260                           Location :   23
    Injection Date  : 9/21/2018 12:04:02 PM               Inj :    1
                                                   Inj Volume : 5.000 µl
    Acq. Method     : E:\DATA\WL\WL-19-35\WL-19-35-rac 2018-09-21 12-02-35\ASH-97-3-1.0ML-ALL-
                      254NM-30MIN.M
    Last changed    : 9/21/2018 12:02:35 PM by SYSTEM
    Analysis Method : E:\DATA\WL\WL-19-35\WL-19-35-rac 2018-09-21 12-02-35\ASH-97-3-1.0ML-ALL-
                      254NM-30MIN.M (Sequence Method)
    Last changed    : 11/7/2018 4:18:23 PM by SYSTEM
                      (modified after loading)
    Additional Info : Peak(s) manually integrated
```

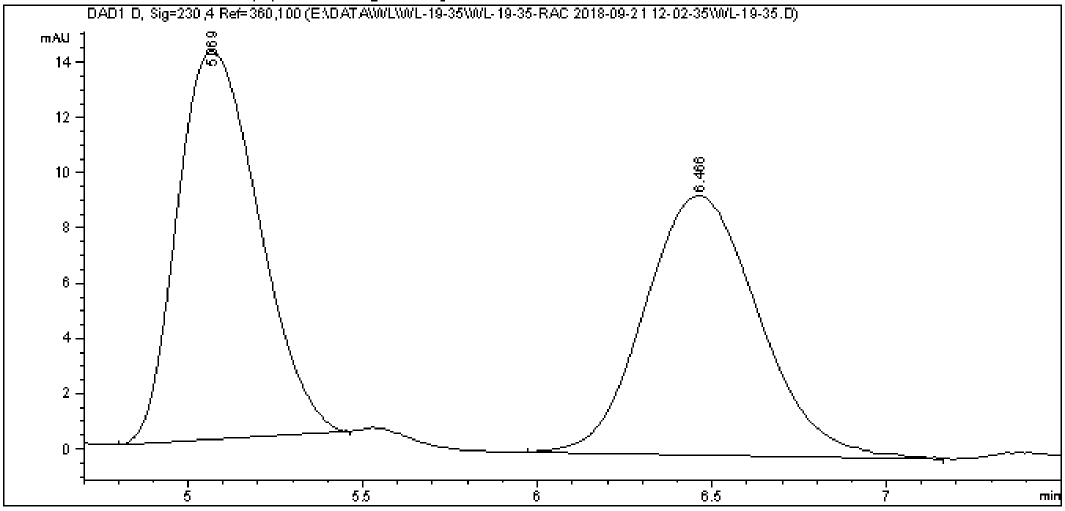


```
    ===================================================================
                             Area Percent Report
    ===================================================================

    Sorted By             :      Signal
    Multiplier            :      1.0000
    Dilution              :      1.0000
    Do not use Multiplier & Dilution Factor with ISTDs


    Signal 1: DAD1 D, Sig=230,4 Ref=360,100

    Peak RetTime Type  Width     Area      Height     Area
      #   [min]        [min]   [mAU*s]     [mAU]        %
    ----|-------|----|-------|----------|----------|--------|
       1   5.069 BB    0.2513  228.18507   14.06123  51.7326
       2   6.466 BB    0.2948  212.90016    9.37952  48.2674

    Totals :                   441.08524   23.44075


    ===================================================================
                             *** End of Report ***
```

1260 11/7/2018 4:18:29 PM SYSTEM Page 1 of 1

Supplementary Figure 202. HPLC spectrum of (*rac*)-**3R**

Data File E:\DATA\WL\WL-19-34\WL-19-34-rac 2018-09-21 12-41-26\WL-19-342.D
Sample Name: WL-19-35-OPT

===

Acq. Operator : SYSTEM Seq. Line : 3
Acq. Instrument : 1260 Location : 72
Injection Date : 9/21/2018 1:29:20 PM Inj : 1
Inj Volume : 5.000 µl
Acq. Method : E:\DATA\WL\WL-19-34\WL-19-34-rac 2018-09-21 12-41-26\ASH-97-3-1.0ML-ALL-254NM-30MIN.M
Last changed : 9/21/2018 1:36:57 PM by SYSTEM
(modified after loading)
Analysis Method : E:\DATA\WL\WL-19-34\WL-19-34-rac 2018-09-21 12-41-26\ASH-97-3-1.0ML-ALL-254NM-30MIN.M (Sequence Method)
Last changed : 11/7/2018 4:19:09 PM by SYSTEM
(modified after loading)
Additional Info : Peak(s) manually integrated

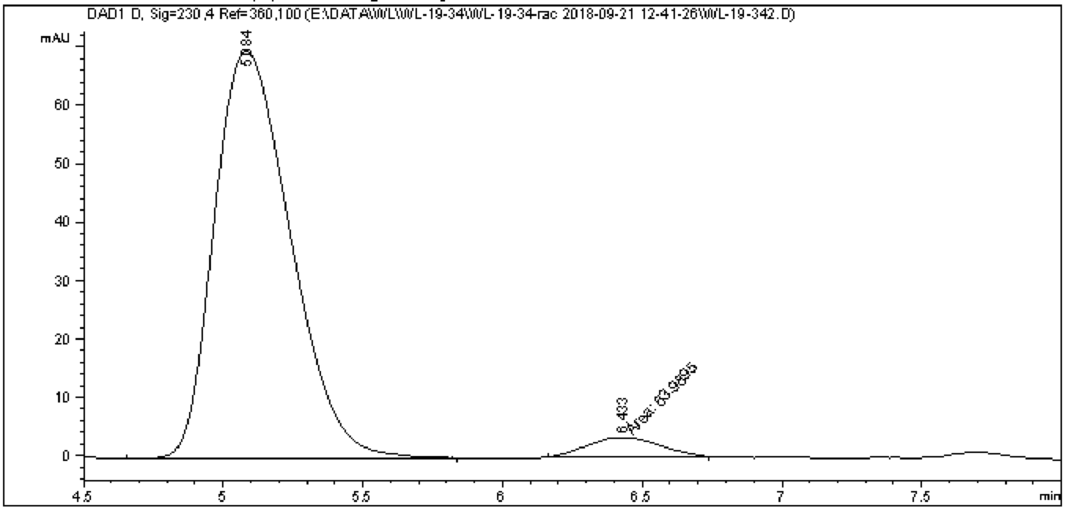

===

Area Percent Report

===

Sorted By : Signal
Multiplier : 1.0000
Dilution : 1.0000
Do not use Multiplier & Dilution Factor with ISTDs

Signal 1: DAD1 D, Sig=230,4 Ref=360,100

Peak #	RetTime [min]	Type	Width [min]	Area [mAU*s]	Height [mAU]	Area %
1	5.084	BB	0.2972	1322.84546	69.77694	95.3859
2	6.433	MM	0.3089	63.98948	3.45255	4.6141

Totals : 1386.83494 73.22949

===

1260 11/7/2018 4:20:15 PM SYSTEM Page 1 of 2

Supplementary Figure 203. HPLC spectrum of (*R*)-**3R**

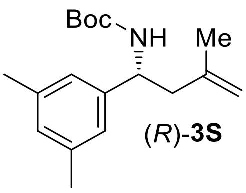

(*R*)-**3S**

Data File E:\DATA\WL\WL-19-43\WL-19-43B-AS 2018-10-08 18-16-57\WL-19-43.D
Sample Name: WL-19-43B-RAC

```
=====================================================================
Acq. Operator   : SYSTEM                         Seq. Line :   1
Acq. Instrument : 1260                            Location :   13
Injection Date  : 10/8/2018 6:18:27 PM                 Inj :   1
                                                Inj Volume : 5.000 µl
Acq. Method     : E:\DATA\WL\WL-19-43\WL-19-43B-AS 2018-10-08 18-16-57\ASH-97-3-1.0ML-ALL-
                  254NM-30MIN.M
Last changed    : 10/8/2018 6:17:34 PM by SYSTEM
                  (modified after loading)
Analysis Method : E:\DATA\WL\WL-19-43\WL-19-43B-AS 2018-10-08 18-16-57\ASH-97-3-1.0ML-ALL-
                  254NM-30MIN.M (Sequence Method)
Last changed    : 11/7/2018 4:57:22 PM by SYSTEM
                  (modified after loading)
Additional Info : Peak(s) manually integrated
```

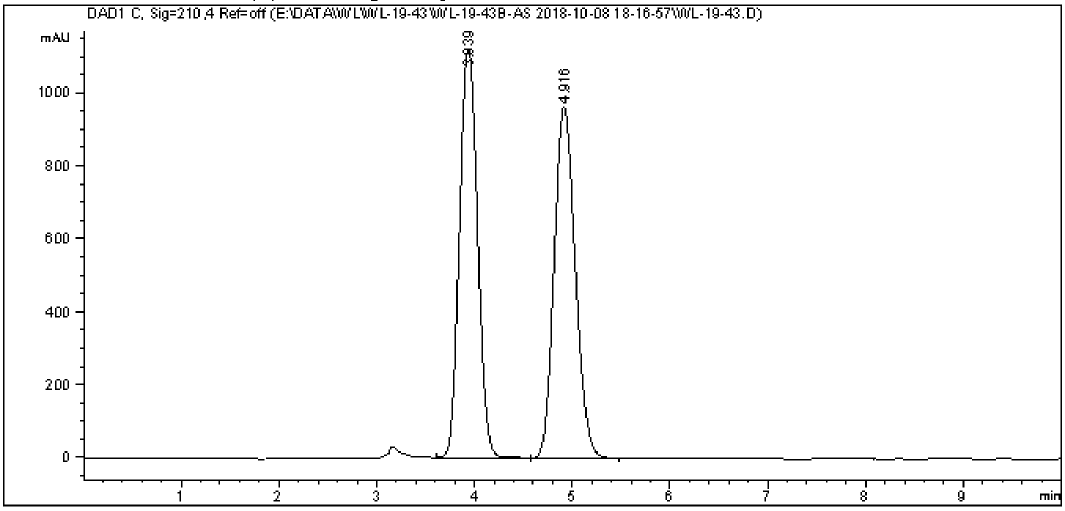


```
=====================================================================
                         Area Percent Report
=====================================================================

Sorted By             :      Signal
Multiplier            :      1.0000
Dilution              :      1.0000
Do not use Multiplier & Dilution Factor with ISTDs


Signal 1: DAD1 C, Sig=210,4 Ref=off
```

Peak #	RetTime [min]	Type	Width [min]	Area [mAU*s]	Height [mAU]	Area %
1	3.939	BB	0.2058	1.44342e4	1116.96216	49.9201
2	4.916	BB	0.2356	1.44804e4	962.26581	50.0799

Totals : 2.89147e4 2079.22797

===

Supplementary Figure 204. HPLC spectrum of (*rac*)-**3S**

Data File E:\DATA\WL\WL-19-43\WL-19-43B-OPT-AS 2018-10-06 11-10-03\WL-19-43.D
Sample Name: WL-19-43B-OPT-AS

===

Acq. Operator : SYSTEM Seq. Line : 1
Acq. Instrument : 1260 Location : 5
Injection Date : 10/6/2018 11:11:30 AM Inj : 1
Inj Volume : 5.000 µl
Acq. Method : E:\DATA\WL\WL-19-43\WL-19-43B-OPT-AS 2018-10-06 11-10-03\ASH-97-3-1.0ML-ALL-254NM-30MIN.M
Last changed : 10/6/2018 11:10:03 AM by SYSTEM
Analysis Method : E:\DATA\WL\WL-19-43\WL-19-43B-OPT-AS 2018-10-06 11-10-03\ASH-97-3-1.0ML-ALL-254NM-30MIN.M (Sequence Method)
Last changed : 11/7/2018 4:54:56 PM by SYSTEM
(modified after loading)
Additional Info : Peak(s) manually integrated

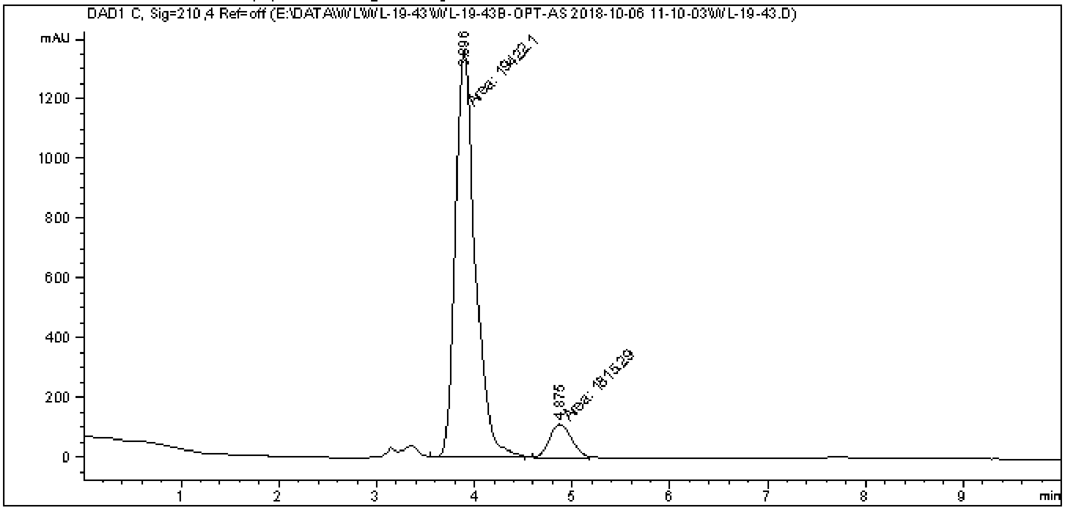

===

Area Percent Report

===

Sorted By : Signal
Multiplier : 1.0000
Dilution : 1.0000
Do not use Multiplier & Dilution Factor with ISTDs

Signal 1: DAD1 C, Sig=210,4 Ref=off

Peak #	RetTime [min]	Type	Width [min]	Area [mAU*s]	Height [mAU]	Area %
1	3.896	MM	0.2377	1.94221e4	1361.97290	91.4524
2	4.875	MM	0.2686	1815.28625	112.62121	8.5476

Totals : 2.12374e4 1474.59411

===

*** End of Report ***

1260 11/7/2018 4:55:01 PM SYSTEM Page 1 of 1

Supplementary Figure 205. HPLC spectrum of (*R*)-**3S**

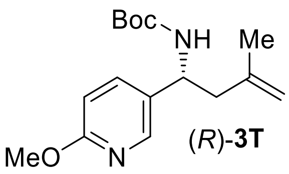

(*R*)-**3T**

Data File E:\DATA\WL\WL-19-42\WL-19-42 2018-09-30 12-03-08\WL-19-422.D
Sample Name: WL-19-42A-RAC

===
Acq. Operator : SYSTEM Seq. Line : 3
Acq. Instrument : 1260 Location : 96
Injection Date : 9/30/2018 1:07:31 PM Inj : 1
Inj Volume : 5.000 µl
Acq. Method : E:\DATA\WL\WL-19-42\WL-19-42 2018-09-30 12-03-08\ASH-97-3-1.0ML-ALL-254NM-30MIN.M
Last changed : 9/30/2018 12:03:08 PM by SYSTEM
Analysis Method : E:\DATA\WL\WL-19-42\WL-19-42 2018-09-30 12-03-08\ASH-97-3-1.0ML-ALL-254NM-30MIN.M (Sequence Method)
Last changed : 11/7/2018 4:23:23 PM by SYSTEM
(modified after loading)
Additional Info : Peak(s) manually integrated

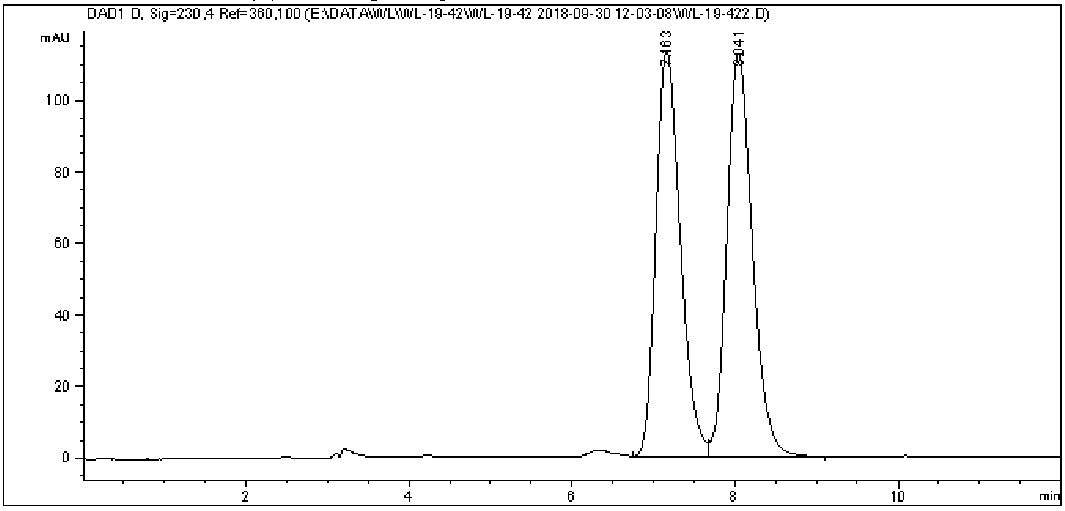

===
Area Percent Report
===

Sorted By : Signal
Multiplier : 1.0000
Dilution : 1.0000
Do not use Multiplier & Dilution Factor with ISTDs

Signal 1: DAD1 D, Sig=230,4 Ref=360,100

Peak #	RetTime [min]	Type	Width [min]	Area [mAU*s]	Height [mAU]	Area %
1	7.163	BV	0.3190	2359.81836	113.30417	48.9266
2	8.041	VB	0.3363	2463.35864	112.57076	51.0734

Totals : 4823.17700 225.87493

===
*** End of Report ***

1260 11/7/2018 4:23:27 PM SYSTEM Page 1 of 1

Supplementary Figure 206. HPLC spectrum of (*rac*)-**3T**

Data File E:\DATA\WL\WL-19-42\WL-19-42 2018-09-30 12-03-08\WL-19-423.D
Sample Name: WL-19-42A-OPT

===

Acq. Operator : SYSTEM Seq. Line : 4
Acq. Instrument : 1260 Location : 97
Injection Date : 9/30/2018 1:39:01 PM Inj : 1
Inj Volume : 5.000 µl
Acq. Method : E:\DATA\WL\WL-19-42\WL-19-42 2018-09-30 12-03-08\ASH-97-3-1.0ML-ALL-254NM-30MIN.M
Last changed : 9/30/2018 12:03:08 PM by SYSTEM
Analysis Method : E:\DATA\WL\WL-19-42\WL-19-42 2018-09-30 12-03-08\ASH-97-3-1.0ML-ALL-254NM-30MIN.M (Sequence Method)
Last changed : 11/7/2018 4:23:23 PM by SYSTEM
(modified after loading)
Additional Info : Peak(s) manually integrated

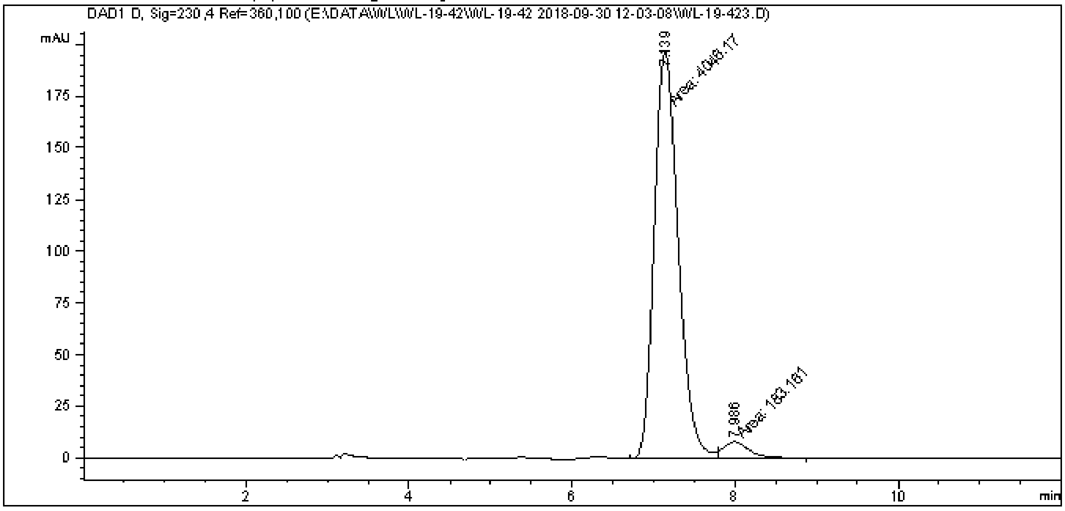

===

Area Percent Report

===

Sorted By : Signal
Multiplier : 1.0000
Dilution : 1.0000
Do not use Multiplier & Dilution Factor with ISTDs

Signal 1: DAD1 D, Sig=230,4 Ref=360,100

Peak #	RetTime [min]	Type	Width [min]	Area [mAU*s]	Height [mAU]	Area %
1	7.139	MF	0.3418	4046.16626	197.32574	95.6693
2	7.986	FM	0.3752	183.16145	8.13528	4.3307

Totals : 4229.32771 205.46102

===

*** End of Report ***

1260 11/7/2018 4:24:53 PM SYSTEM Page 1 of 1

Supplementary Figure 207. HPLC spectrum of (*S*)-**3T**

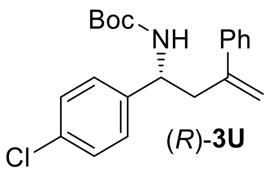

(*R*)-**3U**

Data File E:\DATA\WL\WL-19-34\WL-19-34-rac 2018-09-21 12-41-26\WL-19-34.D
Sample Name: WL-19-34-RAC

===

Acq. Operator : SYSTEM Seq. Line : 1
Acq. Instrument : 1260 Location : 24
Injection Date : 9/21/2018 12:42:54 PM Inj : 1
Inj Volume : 5.000 µl
Acq. Method : E:\DATA\WL\WL-19-34\WL-19-34-rac 2018-09-21 12-41-26\ASH-97-3-1.0ML-ALL-254NM-30MIN.M
Last changed : 9/21/2018 1:11:16 PM by SYSTEM
(modified after loading)
Analysis Method : E:\DATA\WL\WL-19-34\WL-19-34-rac 2018-09-21 12-41-26\ASH-97-3-1.0ML-ALL-254NM-30MIN.M (Sequence Method)
Last changed : 11/7/2018 4:15:14 PM by SYSTEM
(modified after loading)
Additional Info : Peak(s) manually integrated

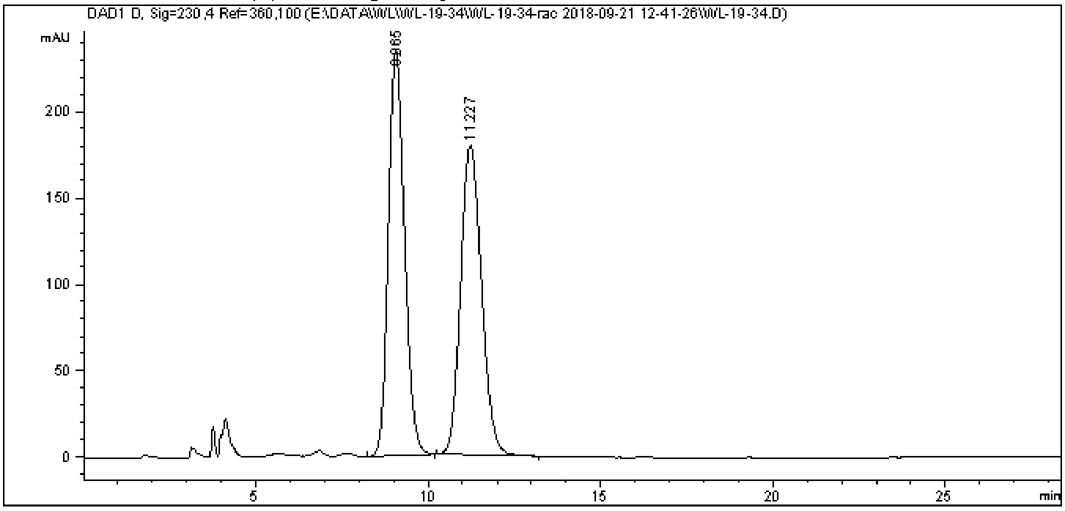

===
Area Percent Report
===

Sorted By : Signal
Multiplier : 1.0000
Dilution : 1.0000
Do not use Multiplier & Dilution Factor with ISTDs

Signal 1: DAD1 D, Sig=230,4 Ref=360,100

Peak #	RetTime [min]	Type	Width [min]	Area [mAU*s]	Height [mAU]	Area %
1	9.065	BB	0.5043	7629.70313	234.07097	50.2799
2	11.227	BB	0.6390	7544.77002	178.91655	49.7201

Totals : 1.51745e4 412.98752

===

1260 11/7/2018 4:15:53 PM SYSTEM Page 1 of 2

Supplementary Figure 208. HPLC spectrum of (*rac*)-**3U**

Data File E:\DATA\WL\WL-19-34\WL-19-34-rac 2018-09-21 12-41-26\WL-19-341.D
Sample Name: WL-19-34-OPT

===

```
Acq. Operator   : SYSTEM                        Seq. Line :    2
Acq. Instrument : 1260                           Location :   71
Injection Date  : 9/21/2018 1:12:48 PM                Inj :    1
                                               Inj Volume : 5.000 µl
Acq. Method     : E:\DATA\WL\WL-19-34\WL-19-34-rac 2018-09-21 12-41-26\ASH-97-3-1.0ML-ALL-
                  254NM-30MIN.M
Last changed    : 9/21/2018 1:11:16 PM by SYSTEM
Analysis Method : E:\DATA\WL\WL-19-34\WL-19-34-rac 2018-09-21 12-41-26\ASH-97-3-1.0ML-ALL-
                  254NM-30MIN.M (Sequence Method)
Last changed    : 11/7/2018 4:15:14 PM by SYSTEM
                  (modified after loading)
Additional Info : Peak(s) manually integrated
```

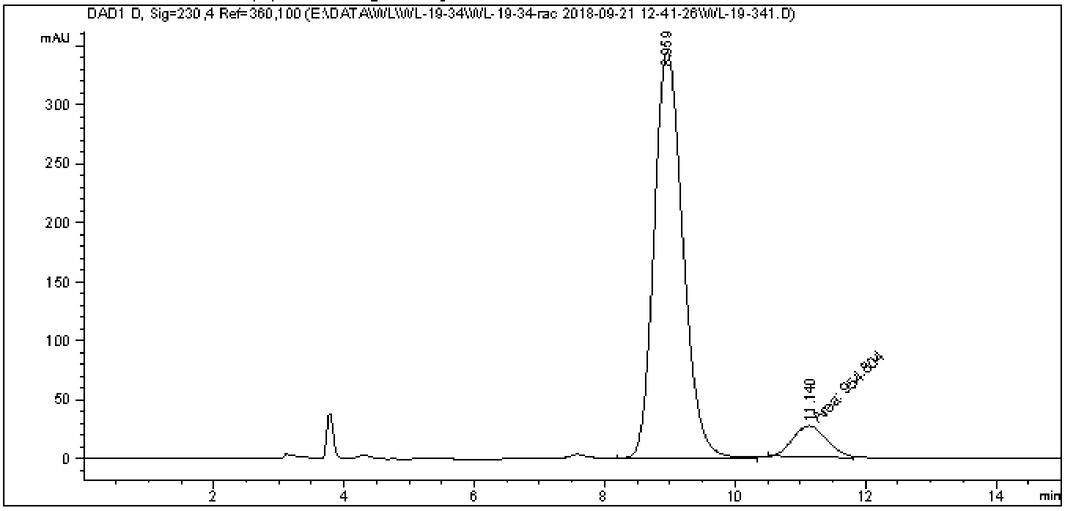

===
Area Percent Report
===

Sorted By : Signal
Multiplier : 1.0000
Dilution : 1.0000
Do not use Multiplier & Dilution Factor with ISTDs

Signal 1: DAD1 D, Sig=230,4 Ref=360,100

Peak #	RetTime [min]	Type	Width [min]	Area [mAU*s]	Height [mAU]	Area %
1	8.959	BB	0.4801	1.05946e4	345.14307	91.7329
2	11.140	MM	0.6074	954.80353	26.19711	8.2671

Totals : 1.15494e4 371.34017

===
*** End of Report ***

1260 11/7/2018 4:16:42 PM SYSTEM Page 1 of 1

Supplementary Figure 209. HPLC spectrum of (*R*)-**3U**

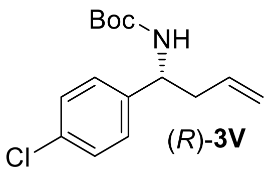

(*R*)-**3V**

Data File E:\DATA\WL\WL-19-12\WL-19-12-STEP-1-RAC-AD 2018-09-18 13-28-29\WL-19-12.D
Sample Name: WL-19-12-STEP-1-RAC

===

Acq. Operator : SYSTEM Seq. Line : 1
Acq. Instrument : 1260 Location : 34
Injection Date : 9/18/2018 1:30:02 PM Inj : 1
Inj Volume : 5.000 µl
Acq. Method : E:\DATA\WL\WL-19-12\WL-19-12-STEP-1-RAC-AD 2018-09-18 13-28-29\ADH-97-3-1.0ML-ALL-254NM-30MIN1.M
Last changed : 9/18/2018 1:28:42 PM by SYSTEM
(modified after loading)
Analysis Method : E:\DATA\WL\WL-19-12\WL-19-12-STEP-1-RAC-AD 2018-09-18 13-28-29\ADH-97-3-1.0ML-ALL-254NM-30MIN1.M (Sequence Method)
Last changed : 11/7/2018 9:04:18 PM by SYSTEM
(modified after loading)
Additional Info : Peak(s) manually integrated

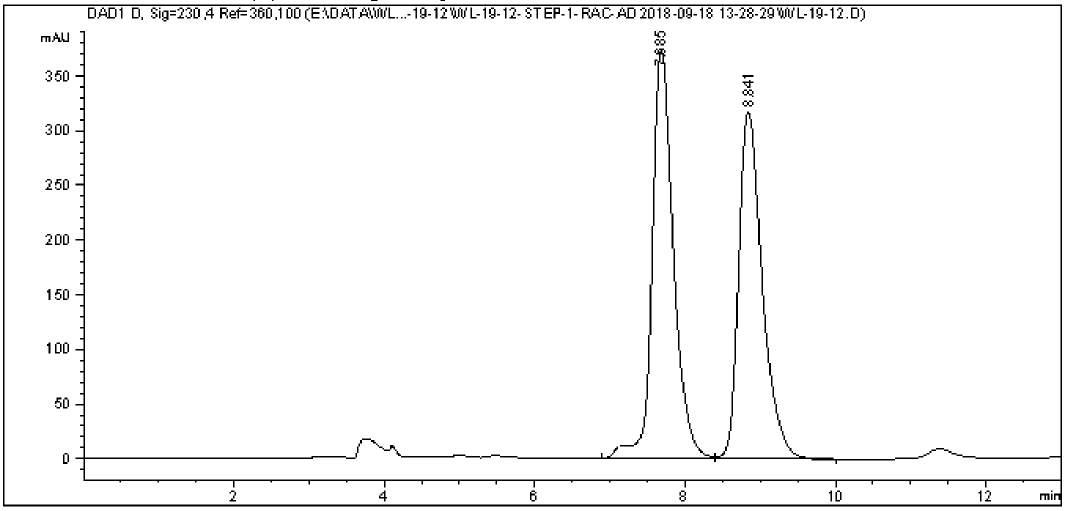

===

Area Percent Report

===

Sorted By : Signal
Multiplier : 1.0000
Dilution : 1.0000
Do not use Multiplier & Dilution Factor with ISTDs

Signal 1: DAD1 D, Sig=230,4 Ref=360,100

Peak #	RetTime [min]	Type	Width [min]	Area [mAU*s]	Height [mAU]	Area %
1	7.685	VV R	0.2963	7386.27588	372.67676	51.6380
2	8.841	VB	0.3337	6917.67676	316.80811	48.3620

Totals : 1.43040e4 689.48486

===

1260 11/7/2018 9:04:22 PM SYSTEM Page 1 of 2

Supplementary Figure 210. HPLC spectrum of (*rac*)-**3V**

Data File E:\DATA\WL\WL-19-12\WL-19-12-STEP-1-OPT 2018-09-18 15-10-30\WL-19-12.D
Sample Name: WL-19-12-STEP-1-OPT-1

```
=====================================================================
Acq. Operator   : SYSTEM                          Seq. Line :    1
Acq. Instrument : 1260                             Location :   35
Injection Date  : 9/18/2018 3:12:04 PM                  Inj :    1
                                               Inj Volume : 5.000 µl
Acq. Method     : E:\DATA\WL\WL-19-12\WL-19-12-STEP-1-OPT 2018-09-18 15-10-30\ADH-97-3-1.0ML-
                  ALL-254NM-30MIN1.M
Last changed    : 9/18/2018 3:10:30 PM by SYSTEM
Analysis Method : E:\DATA\WL\WL-19-12\WL-19-12-STEP-1-OPT 2018-09-18 15-10-30\ADH-97-3-1.0ML-
                  ALL-254NM-30MIN1.M (Sequence Method)
Last changed    : 11/7/2018 4:04:53 PM by SYSTEM
                  (modified after loading)
Additional Info : Peak(s) manually integrated
```

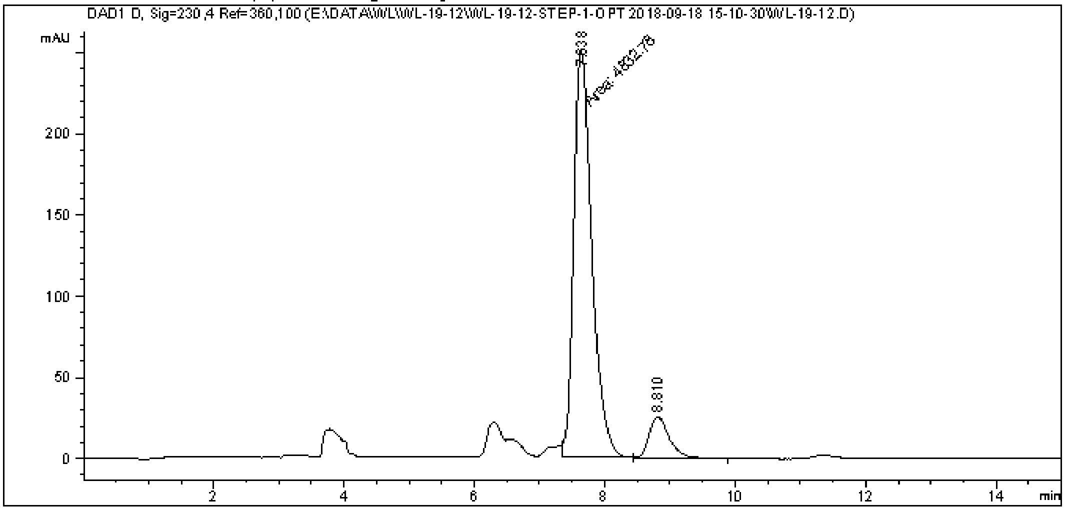


```
=====================================================================
                         Area Percent Report
=====================================================================

Sorted By             :      Signal
Multiplier            :      1.0000
Dilution              :      1.0000
Do not use Multiplier & Dilution Factor with ISTDs


Signal 1: DAD1 D, Sig=230,4 Ref=360,100

Peak RetTime Type  Width     Area      Height     Area
  #   [min]        [min]   [mAU*s]     [mAU]        %
----|-------|----|-------|----------|----------|--------|
   1   7.638 FM    0.3218 4832.77686  250.30499  90.0030
   2   8.810 BB    0.3139  536.79840   25.15751   9.9970

Totals :                  5369.57526  275.46250


=====================================================================
                          *** End of Report ***
```

1260 11/7/2018 4:05:38 PM SYSTEM   Page 1 of 1

Supplementary Figure 211. HPLC spectrum of (*R*)-**3V**

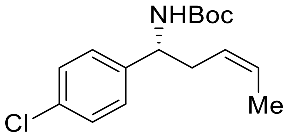

(*R*,*Z*)-**3s**

```
Data File E:\DATA\WL\WL-19-26\WL-19-26 2018-09-21 11-36-10\WL-19-26.D
Sample Name: WL-19-26-RAC

    =====================================================================
    Acq. Operator   : SYSTEM                        Seq. Line :    1
    Acq. Instrument : 1260                           Location :   27
    Injection Date  : 9/21/2018 11:37:34 AM               Inj :    1
                                                   Inj Volume : 5.000 µl
    Acq. Method     : E:\DATA\WL\WL-19-26\WL-19-26 2018-09-21 11-36-10\ASH-97-3-1.0ML-ALL-254NM-
                      30MIN.M
    Last changed    : 9/21/2018 11:48:01 AM by SYSTEM
                      (modified after loading)
    Analysis Method : E:\DATA\WL\WL-19-26\WL-19-26 2018-09-21 11-36-10\ASH-97-3-1.0ML-ALL-254NM-
                      30MIN.M (Sequence Method)
    Last changed    : 11/7/2018 4:09:12 PM by SYSTEM
                      (modified after loading)
    Additional Info : Peak(s) manually integrated
```

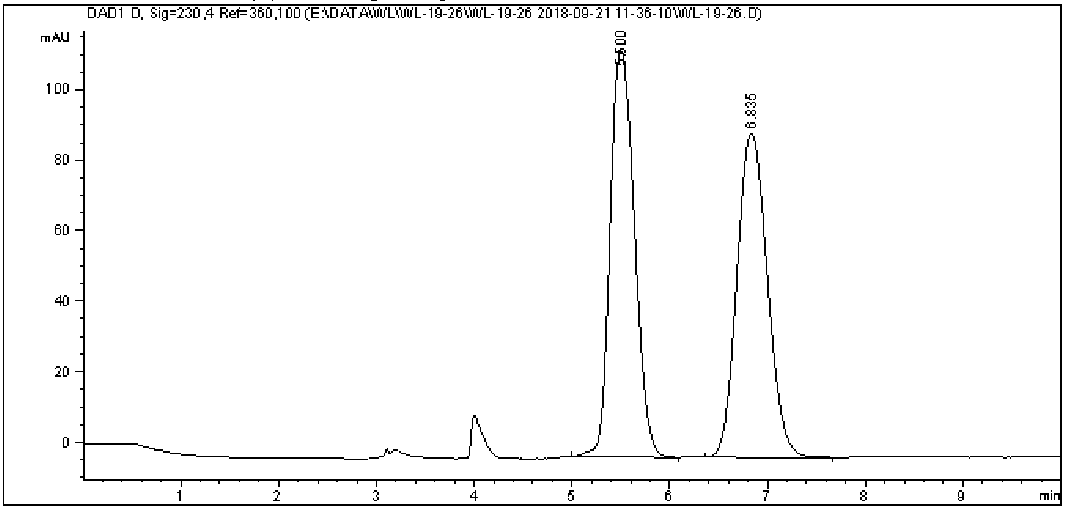


```
    =====================================================================
                             Area Percent Report
    =====================================================================

    Sorted By             :      Signal
    Multiplier            :      1.0000
    Dilution              :      1.0000
    Do not use Multiplier & Dilution Factor with ISTDs


    Signal 1: DAD1 D, Sig=230,4 Ref=360,100

    Peak RetTime Type  Width     Area      Height     Area
      #   [min]        [min]   [mAU*s]     [mAU]        %
    ----|-------|----|-------|----------|----------|--------|
       1   5.500 BB    0.2768 1991.79980  115.15438  50.1522
       2   6.835 BB    0.3364 1979.70947   91.86042  49.8478

    Totals :                  3971.50928  207.01480


    =====================================================================
```


Supplementary Figure 212. HPLC spectrum of (*rac*,*Z*)-**3s**

Data File E:\DATA\WL\WL-19-12\WL-19-12-OPT 2018-09-12 21-48-24\WL-19-12.D
Sample Name: WL-19-12-opt

===

Acq. Operator : SYSTEM Seq. Line : 1
Acq. Instrument : 1260 Location : 7
Injection Date : 9/12/2018 9:49:52 PM Inj : 1
Inj Volume : 5.000 µl
Acq. Method : E:\DATA\WL\WL-19-12\WL-19-12-opt 2018-09-12 21-48-24\ASH-97-3-1.0ML-ALL-254NM-30MIN.M
Last changed : 9/12/2018 9:48:39 PM by SYSTEM
(modified after loading)
Analysis Method : E:\DATA\WL\WL-19-12\WL-19-12-opt 2018-09-12 21-48-24\ASH-97-3-1.0ML-ALL-254NM-30MIN.M (Sequence Method)
Last changed : 11/7/2018 4:01:06 PM by SYSTEM
(modified after loading)
Additional Info : Peak(s) manually integrated

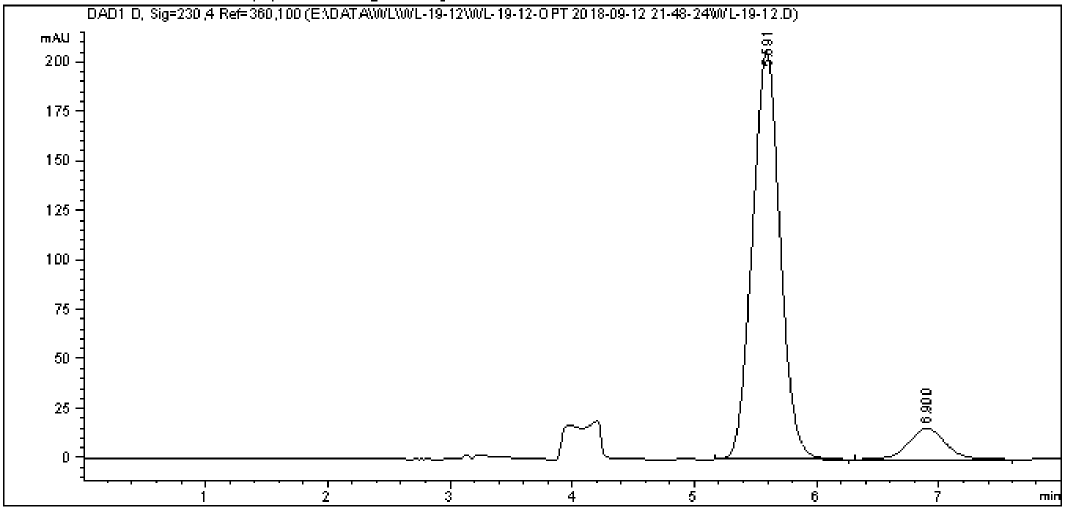

===

Area Percent Report

===

Sorted By : Signal
Multiplier : 1.0000
Dilution : 1.0000
Do not use Multiplier & Dilution Factor with ISTDs

Signal 1: DAD1 D, Sig=230,4 Ref=360,100

Peak #	RetTime [min]	Type	Width [min]	Area [mAU*s]	Height [mAU]	Area %
1	5.591	BB	0.2402	3254.70142	206.19867	90.8990
2	6.900	BB	0.3119	325.86774	15.58762	9.1010

Totals : 3580.56915 221.78628

===

1260 11/7/2018 4:01:09 PM SYSTEM Page 1 of 2

Supplementary Figure 213. HPLC spectrum of (*R*,*Z*)-**3s**

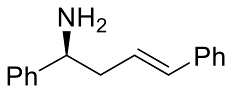

(*S*)-**5**

Data File E:\DATA\WL...ION\PRIMARY-AMINE-RAC-4 2018-04-06 16-28-49\paper7-transformation.D
Sample Name: PRIMARY-AMINE-RAC

===

Acq. Operator : SYSTEM Seq. Line : 1
Acq. Instrument : 1260 Location : 14
Injection Date : 4/6/2018 4:30:16 PM Inj : 1
Inj Volume : 5.000 µl
Acq. Method : E:\DATA\WL\paper7-transformation\PRIMARY-AMINE-RAC-4 2018-04-06 16-28-49
\ODH-60-40-1.0ML-ALL-254NM-30MIN.M
Last changed : 4/6/2018 4:28:50 PM by SYSTEM
Analysis Method : E:\DATA\WL\paper7-transformation\PRIMARY-AMINE-RAC-4 2018-04-06 16-28-49
\ODH-60-40-1.0ML-ALL-254NM-30MIN.M (Sequence Method)
Last changed : 5/2/2018 9:51:42 PM by SYSTEM
(modified after loading)
Additional Info : Peak(s) manually integrated

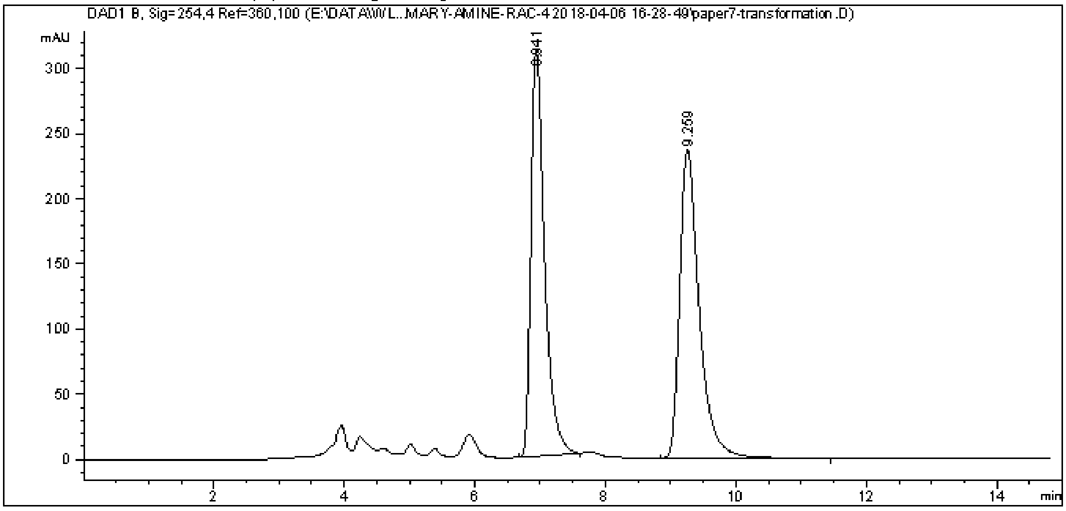

===

Area Percent Report

===

Sorted By : Signal
Multiplier : 1.0000
Dilution : 1.0000
Do not use Multiplier & Dilution Factor with ISTDs

Signal 1: DAD1 B, Sig=254,4 Ref=360,100

Peak #	RetTime [min]	Type	Width [min]	Area [mAU*s]	Height [mAU]	Area %
1	6.941	BB	0.2186	4526.76807	311.35965	47.9363
2	9.259	BB	0.3130	4916.52832	236.09406	52.0637

Totals : 9443.29639 547.45370

===

*** End of Report ***

1260 5/2/2018 9:51:45 PM SYSTEM Page 1 of 1

Supplementary Figure 214. HPLC spectrum of (*rac*)-**5**

Data File E:\DATA\WL...TION\PRIMARY-AMINE-RAC 2018-03-23 10-08-14\paper7-transformation1.D
Sample Name: primary-amine-opt

===

Acq. Operator : SYSTEM Seq. Line : 2
Acq. Instrument : 1260 Location : 16
Injection Date : 3/23/2018 10:27:14 AM Inj : 1
Inj Volume : 5.000 µl
Acq. Method : E:\DATA\WL\paper7-transformation\Primary-amine-RAC 2018-03-23 10-08-14\ODH-60-40-1.0ML-ALL-254NM-30MIN.M
Last changed : 3/23/2018 10:25:31 AM by SYSTEM
Analysis Method : E:\DATA\WL\paper7-transformation\Primary-amine-RAC 2018-03-23 10-08-14\ODH-60-40-1.0ML-ALL-254NM-30MIN.M (Sequence Method)
Last changed : 5/2/2018 9:52:24 PM by SYSTEM
(modified after loading)
Additional Info : Peak(s) manually integrated

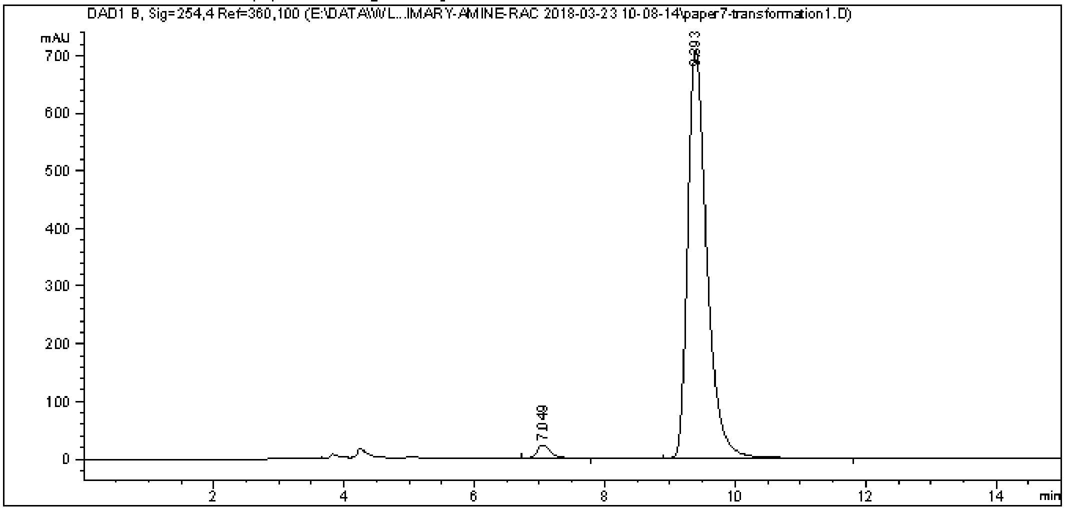

===

Area Percent Report

===

Sorted By : Signal
Multiplier : 1.0000
Dilution : 1.0000
Do not use Multiplier & Dilution Factor with ISTDs

Signal 1: DAD1 B, Sig=254,4 Ref=360,100

Peak #	RetTime [min]	Type	Width [min]	Area [mAU*s]	Height [mAU]	Area %
1	7.049	BB	0.2254	342.36392	22.90691	2.3071
2	9.393	BB	0.3103	1.44972e4	706.75562	97.6929

Totals : 1.48395e4 729.66252

===

*** End of Report ***

1260 5/2/2018 9:52:26 PM SYSTEM Page 1 of 1

Supplementary Figure 215. HPLC spectrum of (*S*)-**5**

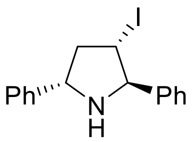

(2*R*,3*S*,5*S*)-**12**

Data File E:\DATA\WL...ATION\IDO-AMINATION-RAC 2018-03-06 15-13-31\paper7-transformation.D
Sample Name: ido-amination-rac

```
=====================================================================
Acq. Operator   : SYSTEM                          Seq. Line :   1
Acq. Instrument : 1260                             Location :   1
Injection Date  : 3/6/2018 3:14:59 PM                   Inj :   1
                                               Inj Volume : 5.000 µl
Acq. Method     : E:\DATA\WL\paper7-transformation\ido-amination-rac 2018-03-06 15-13-31\IAH-
                  97-3-1.0ML-ALL-210NM-30MIN.M
Last changed    : 3/6/2018 3:13:31 PM by SYSTEM
Analysis Method : E:\DATA\WL\paper7-transformation\ido-amination-rac 2018-03-06 15-13-31\IAH-
                  97-3-1.0ML-ALL-210NM-30MIN.M (Sequence Method)
Last changed    : 5/2/2018 9:49:35 PM by SYSTEM
                  (modified after loading)
Additional Info : Peak(s) manually integrated
```

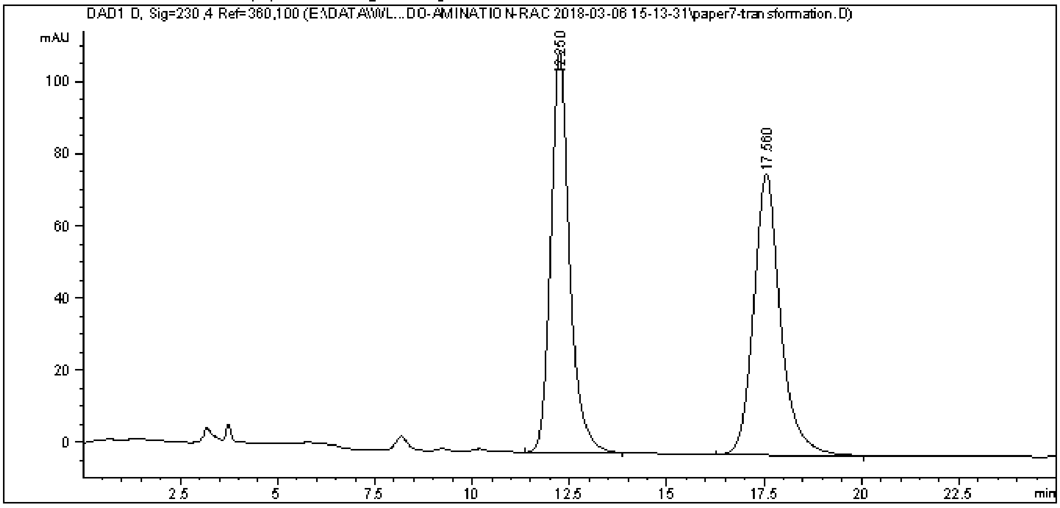


```
=====================================================================
                     Area Percent Report
=====================================================================

Sorted By             :      Signal
Multiplier            :      1.0000
Dilution              :      1.0000
Do not use Multiplier & Dilution Factor with ISTDs


Signal 1: DAD1 D, Sig=230,4 Ref=360,100

Peak RetTime Type  Width     Area      Height     Area
  #   [min]        [min]   [mAU*s]     [mAU]        %
----|-------|----|-------|----------|----------|--------|
   1  12.250 BB    0.4991 3728.93530  111.23530  49.9872
   2  17.560 BB    0.7150 3730.84351   77.59152  50.0128

Totals :                  7459.77881  188.82682


=====================================================================
                       *** End of Report ***
```

1260 5/2/2018 9:49:38 PM SYSTEM   Page 1 of 1

Supplementary Figure 216. HPLC spectrum of (*rac*)-**12**

Data File E:\DATA\WL...ATION\IDO-AMINATION-OPT 2018-03-06 15-53-53\paper7-transformation.D
Sample Name: ido-amination-opt

```
=====================================================================
Acq. Operator   : SYSTEM                        Seq. Line :   1
Acq. Instrument : 1260                           Location :   2
Injection Date  : 3/6/2018 3:55:10 PM                 Inj :   1
                                               Inj Volume : 5.000 µl
Acq. Method     : E:\DATA\WL\paper7-transformation\ido-amination-opt 2018-03-06 15-53-53\IAH-
                  97-3-1.0ML-ALL-210NM-30MIN.M
Last changed    : 3/6/2018 3:53:53 PM by SYSTEM
Analysis Method : E:\DATA\WL\paper7-transformation\ido-amination-opt 2018-03-06 15-53-53\IAH-
                  97-3-1.0ML-ALL-210NM-30MIN.M (Sequence Method)
Last changed    : 5/2/2018 9:50:14 PM by SYSTEM
                  (modified after loading)
Additional Info : Peak(s) manually integrated
```

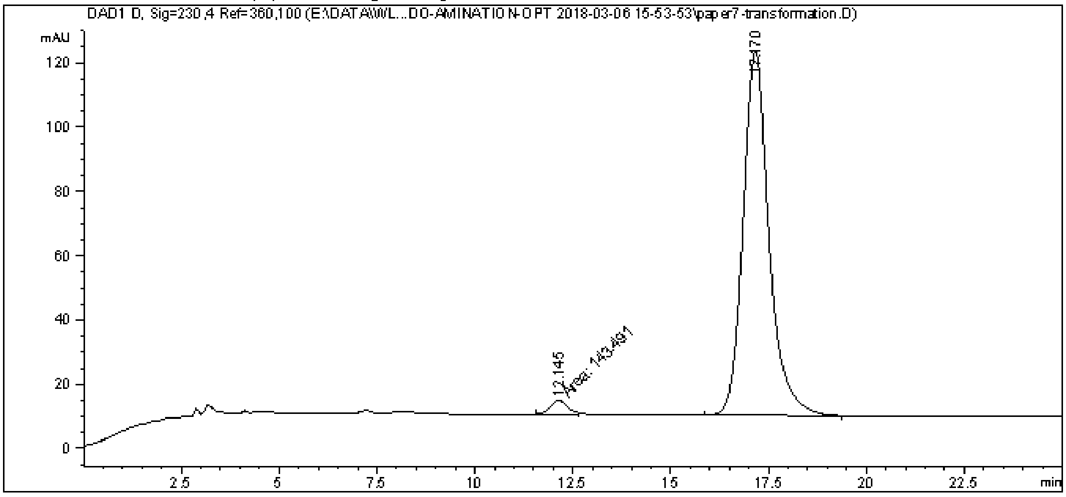


```
=====================================================================
                         Area Percent Report
=====================================================================

Sorted By             :      Signal
Multiplier            :      1.0000
Dilution              :      1.0000
Do not use Multiplier & Dilution Factor with ISTDs


Signal 1: DAD1 D, Sig=230,4 Ref=360,100
```

Peak #	RetTime [min]	Type	Width [min]	Area [mAU*s]	Height [mAU]	Area %
1	12.145	MM	0.5328	143.49069	4.48890	2.6724
2	17.170	BB	0.6906	5225.91455	113.61776	97.3276

Totals : 5369.40524 118.10666

```
=====================================================================
                          *** End of Report ***
```

1260 5/2/2018 9:50:17 PM SYSTEM   Page 1 of 1

Supplementary Figure 217. HPLC spectrum of (2*R*,3*S*,5*S*)-**12**

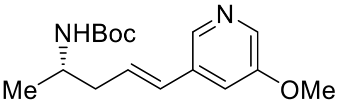

(*S*)-**10**

Data File E:\DATA\WL\WL-18-88\WL-18-88P 2018-01-18 16-38-03\WL-18-88.D
Sample Name: WL-18-88P-RAC

===

Acq. Operator : SYSTEM   Seq. Line : 1
Acq. Instrument : 1260   Location : 94
Injection Date : 1/18/2018 4:39:32 PM   Inj : 1
Inj Volume : 5.000 µl
Acq. Method : E:\DATA\WL\WL-18-88\WL-18-88P 2018-01-18 16-38-03\ODH-75-25-1.0ML-ALL-254NM-30MIN.M
Last changed : 1/18/2018 4:45:01 PM by SYSTEM
(modified after loading)
Analysis Method : E:\DATA\WL\WL-18-88\WL-18-88P 2018-01-18 16-38-03\ODH-75-25-1.0ML-ALL-254NM-30MIN.M (Sequence Method)
Last changed : 5/2/2018 9:34:45 PM by SYSTEM
(modified after loading)
Additional Info : Peak(s) manually integrated

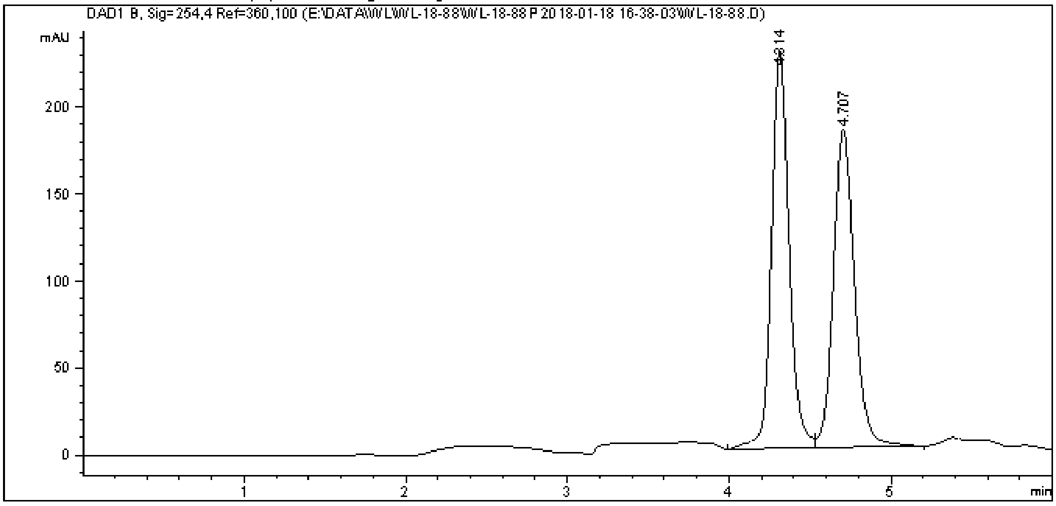

===

Area Percent Report

===

Sorted By : Signal
Multiplier : 1.0000
Dilution : 1.0000
Do not use Multiplier & Dilution Factor with ISTDs

Signal 1: DAD1 B, Sig=254,4 Ref=360,100

Peak #	RetTime [min]	Type	Width [min]	Area [mAU*s]	Height [mAU]	Area %
1	4.314	BV	0.1154	1734.40332	228.92056	51.2174
2	4.707	VB	0.1386	1651.95422	182.46173	48.7826

Totals : 3386.35754 411.38229

===

1260 5/2/2018 9:34:47 PM SYSTEM   Page 1 of 2

Supplementary Figure 218. HPLC spectrum of (*rac*)-**10**

Data File E:\DATA\WL\WL-18-88\WL-18-88P-OPT 2018-02-07 12-05-08\WL-18-881.D
Sample Name: WL-18-88P-OPT

===

Acq. Operator : SYSTEM Seq. Line : 2
Acq. Instrument : 1260 Location : 86
Injection Date : 2/7/2018 12:27:48 PM Inj : 1
Inj Volume : 5.000 µl
Acq. Method : E:\DATA\WL\WL-18-88\WL-18-88P-OPT 2018-02-07 12-05-08\ODH-75-25-1.0ML-ALL-254NM-30MIN.M
Last changed : 2/7/2018 12:05:21 PM by SYSTEM
Analysis Method : E:\DATA\WL\WL-18-88\WL-18-88P-OPT 2018-02-07 12-05-08\ODH-75-25-1.0ML-ALL-254NM-30MIN.M (Sequence Method)
Last changed : 5/2/2018 9:36:19 PM by SYSTEM
(modified after loading)
Additional Info : Peak(s) manually integrated

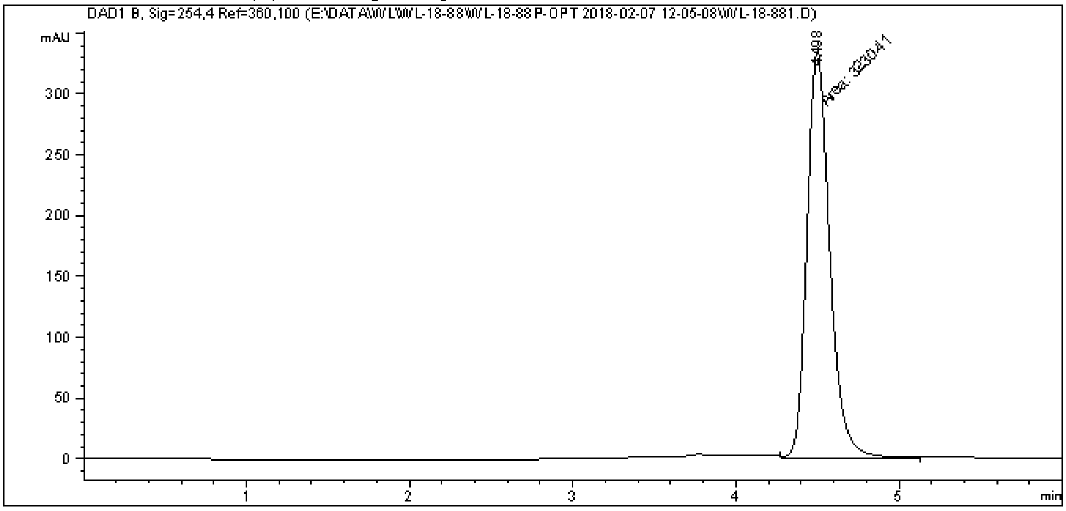

===

Area Percent Report

===

Sorted By : Signal
Multiplier : 1.0000
Dilution : 1.0000
Do not use Multiplier & Dilution Factor with ISTDs

Signal 1: DAD1 B, Sig=254,4 Ref=360,100

Peak #	RetTime [min]	Type	Width [min]	Area [mAU*s]	Height [mAU]	Area %
1	4.498	FM	0.1609	3230.41260	334.54758	100.0000

Totals : 3230.41260 334.54758

===

*** End of Report ***

1260 5/2/2018 9:36:22 PM SYSTEM Page 1 of 1

Supplementary Figure 219. HPLC spectrum of (*S*)-**10**

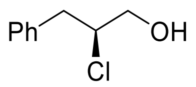

(*S*)-**14**

```
Data File E:\DATA\WL...MATION\CHLORO-AMINATION 2018-03-22 20-34-22\paper7-tranformation2.D
Sample Name: chloro-amination-rac

    =====================================================================
    Acq. Operator   : SYSTEM                         Seq. Line :   3
    Acq. Instrument : 1260                            Location :   5
    Injection Date  : 3/22/2018 9:13:46 PM                 Inj :   1
                                                    Inj Volume : 5.000 µl
    Acq. Method     : E:\DATA\WL\paper7-transformation\Chloro-Amination 2018-03-22 20-34-22\ODH-
                      97-3-1.0ML-ALL-210NM-30MIN.M
    Last changed    : 3/22/2018 9:10:16 PM by SYSTEM
    Analysis Method : E:\DATA\WL\paper7-transformation\Chloro-Amination 2018-03-22 20-34-22\ODH-
                      97-3-1.0ML-ALL-210NM-30MIN.M (Sequence Method)
    Last changed    : 5/2/2018 9:44:23 PM by SYSTEM
                      (modified after loading)
    Additional Info : Peak(s) manually integrated
```

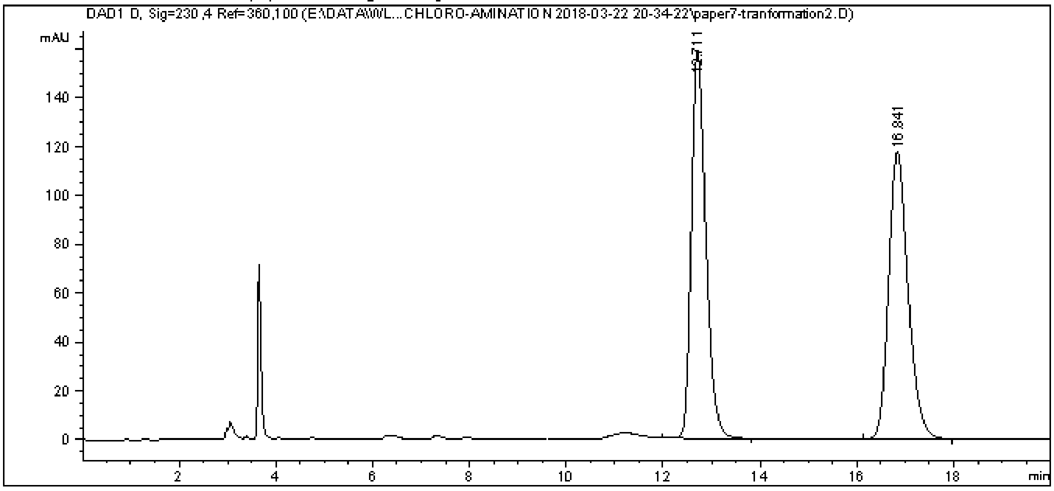


```
    =====================================================================
                             Area Percent Report
    =====================================================================

    Sorted By             :      Signal
    Multiplier            :      1.0000
    Dilution              :      1.0000
    Do not use Multiplier & Dilution Factor with ISTDs


    Signal 1: DAD1 D, Sig=230,4 Ref=360,100

    Peak RetTime Type  Width     Area      Height     Area
      #   [min]        [min]   [mAU*s]     [mAU]        %
    ----|-------|----|-------|----------|----------|--------|
       1  12.711 BB    0.3138 3297.97046  159.12433  50.0992
       2  16.841 BB    0.4252 3284.91504  117.48693  49.9008

    Totals :                  6582.88550  276.61126


    =====================================================================
                             *** End of Report ***

1260 5/2/2018 9:44:26 PM SYSTEM                                              Page 1 of 1
```

Supplementary Figure 220. HPLC spectrum of (*rac*)-**14**

Data File E:\DATA\WL...N\CHLORO-AMINATION-OPT 2018-03-23 11-43-20\paper7-transformation1.D
Sample Name: Chloro-Amination-OPT

===

Acq. Operator : SYSTEM Seq. Line : 2
Acq. Instrument : 1260 Location : 11
Injection Date : 3/23/2018 12:05:48 PM Inj : 1
Inj Volume : 5.000 µl
Acq. Method : E:\DATA\WL\paper7-transformation\Chloro-Amination-OPT 2018-03-23 11-43-20\ODH-97-3-1.0ML-ALL-210NM-30MIN.M
Last changed : 3/23/2018 11:43:29 AM by SYSTEM
Analysis Method : E:\DATA\WL\paper7-transformation\Chloro-Amination-OPT 2018-03-23 11-43-20\ODH-97-3-1.0ML-ALL-210NM-30MIN.M (Sequence Method)
Last changed : 5/2/2018 9:45:29 PM by SYSTEM
(modified after loading)
Additional Info : Peak(s) manually integrated

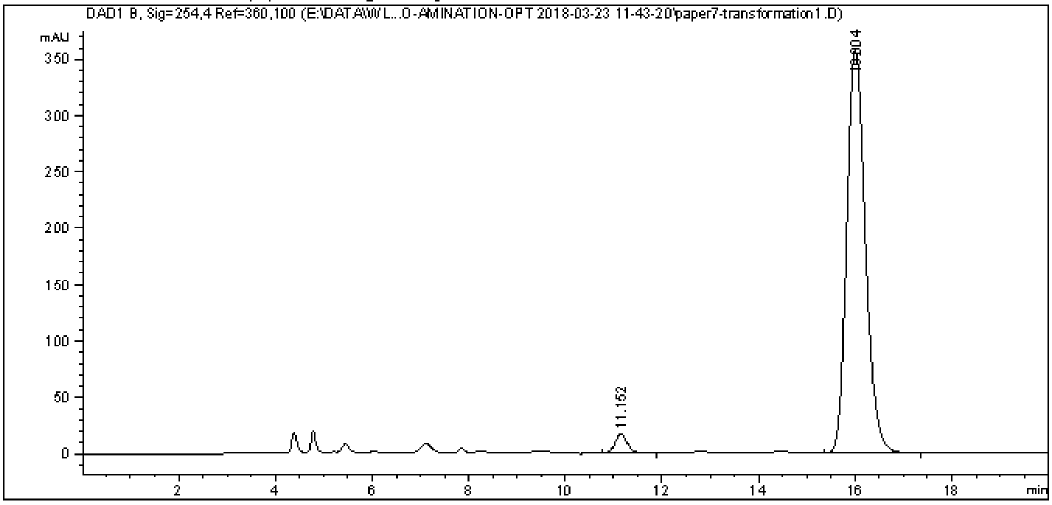

===

Area Percent Report

===

Sorted By : Signal
Multiplier : 1.0000
Dilution : 1.0000
Do not use Multiplier & Dilution Factor with ISTDs

Signal 1: DAD1 B, Sig=254,4 Ref=360,100

Peak #	RetTime [min]	Type	Width [min]	Area [mAU*s]	Height [mAU]	Area %
1	11.152	BB	0.2714	305.51883	17.18824	3.0985
2	16.004	BB	0.4121	9554.77051	357.11279	96.9015

Totals : 9860.28934 374.30103

===

*** End of Report ***

Supplementary Figure 221. HPLC spectrum of (*S*)-**14**

Supplementary References

1. Wang, C.-J.; Liang, G.; Xue, Z.-Y.; Gao, F. Highly Enantioselective 1,3-Dipolar Cycloaddition of Azomethine Ylides Catalyzed by Copper(I)/TF-BiphamPhos Complexes. *J. Am. Chem. Soc.* **130**, 17250 (2008).

2. Stanley, L. M.; Hartwig, J. F. Iridium-Catalyzed Regio- and Enantioselective N-Allylation of Indoles. *Angew. Chem., Int. Ed.* **48**, 7841 (2009).

3 Richards, C. J.; Mulvaney, A. W.; Synthesis of phosphinoferrocenyloxazolines. New ligands for asymmetric catalysis. *Tetrahedron: Asymmetry* **7**, 1419 (1996).

4 Smith, C. R.; Mans, D. J.; Rajanbabu, T. V. (*R*)-2,2'-BINAPHTHOYL-(*S*,*S*)-DI(1-PHENYLETHYL) AMINOPHOSPHINE. SCALABLE PROTOCOLS FOR THE SYNTHESES OF PHOSPHORAMIDITE (FERINGA) LIGANDS. *Org. Synth.* **85**, 238 (2008).

5 Delaye, P.-O., Vasse, J.-L., Szymoniak, J. Switching Regioselectivity in the Allylation of Imines by N-Side Chain Tuning. *Org. Lett.* **14**, 3004 (2012).

6 Román, J. G. Soderquist, J. A. Asymmetric Synthesis of 2°- and 3°-Carbinols via *B*-Methallyl-10-(TMS and Ph)-9-borabicyclo[3.3.2]decanes. *J. Org. Chem.* **72**, 9772 (2007).

7 Kiener, C. A.; Shu, C.; Incarvito, C.; Hartwig, J. H. Identification of an Activated Catalyst in the Iridium-Catalyzed Allylic Amination and Etherification. Increased Rates, Scope, and Selectivity. *J. Am. Chem. Soc.* **125**, 14272 (2003).
